# Electronic Finetuning of 8‐Methoxy Psoralens by Palladium‐Catalyzed Coupling: Acidochromicity and Solvatochromicity

**DOI:** 10.1002/chem.201905676

**Published:** 2020-05-29

**Authors:** Sarah R. Geenen, Lysander Presser, Torsten Hölzel, Christian Ganter, Thomas J. J. Müller

**Affiliations:** ^1^ Institut für Organische Chemie und Makromolekulare Chemie Heinrich-Heine-Universität Düsseldorf Universitätsstraße 1 40225 Düsseldorf Germany; ^2^ Institut für Anorganische Chemie und Strukturchemie I Heinrich-Heine-Universität Düsseldorf Universitätsstraße 1 40225 Düsseldorf Germany

**Keywords:** acidochromism, cross-coupling reactions, density functional calculations, donor–acceptor dyes, solvatochromism, UV/Vis spectroscopy

## Abstract

Differently 5‐substituted 8‐methoxypsoralens can be synthesized by an efficient synthetic route with various cross‐coupling methodologies, such as Suzuki, Sonogashira and Heck reaction. Compared to previously synthesized psoralens, thereby promising daylight absorbing compounds as potentially active agents against certain skin diseases can be readily accessed. Extensive investigations of all synthesized psoralen derivatives reveal fluorescence in the solid state as well as several distinctly emissive derivatives in solution. Donor‐substituted psoralens exhibit remarkable photophysical properties, such as high fluorescence quantum yields and pronounced emission solvatochromicity and acidochromicity, which were scrutinized by Lippert–Mataga and Stern–Volmer plots. The results indicate that the compounds exceed the limit of visible light, a significant factor for potential applications as an active agent. In addition, (TD)DFT calculations were performed to elucidate the underlying electronic structure and to assign experimentally obtained data.

## Graphical abstract

**A diverse and colorful** library of novel psoralens can be obtained by cross‐coupling reactions of 5‐bromo‐8‐methoxypsoralen, which was prepared by a novel multistep synthesis. Photophysical properties, such as emission solvatochromicity and acidochromicity make this class of functional chromophores particularly attractive.

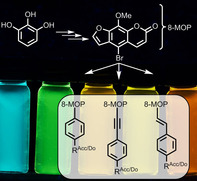

## Introduction

The development of biologically active small molecules has reached increasing importance for applications in medicine,^1^ biology^2^ and biochemistry,^3^ in particular, in the fields of diagnosis and therapy^4^ of certain diseases. As a consequence exploration of novel pharmacophores and structures remains an ongoing major challenge in synthetic chemistry.^5^ In particular, photophysical properties might significantly affect the effectivity of some active ingredients. Controlling excited state properties by diversity oriented synthetic strategies, such as multicomponent processes,^6^ is becoming increasingly important.

**Figure 1:**
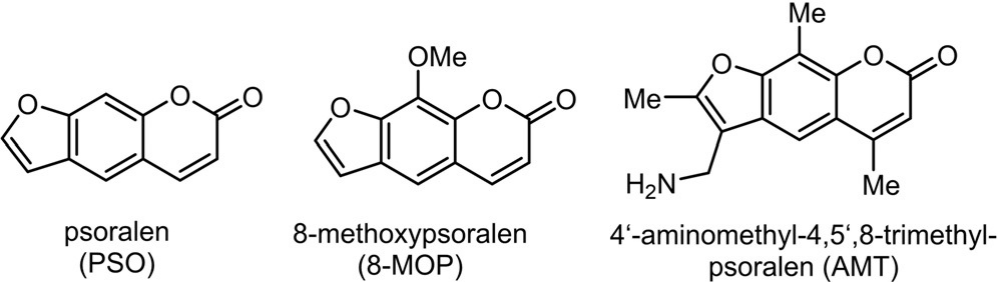
Selected psoralen compounds.

Psoralen (Figure 1) is a privileged pharmacophore with photosensitizing character that interacts^7^ with human DNA and can be used to treat many different types of skin diseases.^8^ The psoralen derivative 8‐methoxypsoralen (8‐MOP) can be used, for example, for the treatment of vitiligo^9^ or T‐cell lymphoma^10^ and promotes the healing of psoriasis by a photochemotherapeutic approach.^11^

The PUVA mechanism (psoralen+UVA radiation) assumes a key role in this process.^12^ Previous studies suggest that a double [2+2] cycloaddition occurs between the furan and the pyrone moieties of psoralen and the DNA.^13^ This crosslinking of the DNA structure induces apoptosis, which prevents the cell from reproducing. Recent studies also indicate that a photo‐induced electron transfer competes with the cycloaddition reaction.7a, 14

**Scheme 1:**
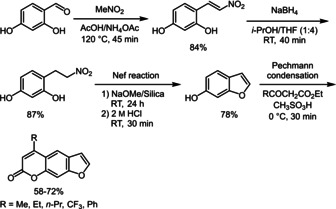
State‐of‐the‐art on the ring‐forming psoralen synthesis.

For further advancing previous investigations and for establishing coherence between various psoralens, it is necessary to establish efficient routes to novel electronically tunable psoralens. Most of the previously known synthetic routes of psoralens start from coumarin, umbelliferon or benzofuran (Scheme 1).^15^

Here, we report a diversity‐oriented route of 8‐MOP derivatives starting from pyrogallol by applying cross‐coupling methodologies to 5‐bromo‐8‐MOP for accessing donor–acceptor substituted systems in which the psoralen core acts as a donor. Furthermore, photophysical properties are studied by absorption and emission spectroscopy, as well as observed solvatochromism and halochromism is reported.

## Results and Discussion

### Synthesis

**Scheme 2:**
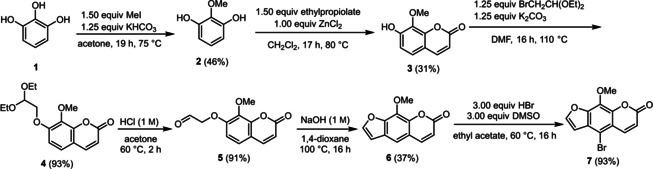
Synthesis of 5‐bromo‐8‐methoxypsoralen (**7**).

Usually, psoralen derivatives are synthesized starting from psoralen or coumarin analogues.15b, 16 For providing a faster and more versatile access, we established a new synthetic route. As an easily affordable starting material pyrogallol (**1**) was chosen. Methylation^17^ followed by hydroarylation^18^ with ethyl propiolate furnished 8‐methoxyumbelliferon (**3**) (Scheme 2). Interestingly, until today compound **3** has only been prepared from more complex starting materials in more sophisticated syntheses.^19^ Application of the hydroarylation on 2‐methoxyresorcinol (**2**) according to Costa et al.18a including solvent change and stoichiometry of ethyl propiolate provided another umbelliferon derivate. The third step of the six‐step synthetic route was achieved by the Williamson ether synthesis^20^ with very good yields (Scheme 2). Subsequent acetal cleavage with hydrochloric acid gave umbelliferon derivative **5**. Other acids^21^ as well as basic acetal cleavage with sodium hydroxide only led to low yields. The subsequent cyclization to 8‐methoxypsoralen (**6**) was carried out according to Nupponen et al.^21^ The halogen functionality required for coupling reactions is finally introduced by bromination with hydrobromic acid in DMSO. This bromination method^22^ is easier to handle than direct bromination with elemental bromine. Furthermore, the 8‐methoxypsoralen (**6**) is efficiently and selectively brominated in 5‐position.

**Scheme 3:**
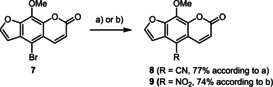
Synthesis of 5‐cyano‐8‐methoxypsoralen (**8**) and 5‐nitro‐8‐methoxypsoralen (**9**). a) 4.00 mol % Zn(OAc)_2_, 4.00 mol % Zn, 1.20 equivalents Zn(CN)_2,_ 0.20 mol % Pd_2_(dba)_3,_ 0.48 mol % dppf, DMF/H_2_O (1:0.001), 16 h, 100 °C. b) glacial acetic acid/HNO_3_ (2:1), 1 h, 0 °C–RT.

5‐Cyano‐ and 5‐nitrosubstituted 8‐methoxy psoralens were prepared by selective displacement. After failed Beller cyanation,^23^ cyanation using zinc cyanide^24^ was attempted and the cyano product **8** was obtained in 77 % yield (Scheme 3). The intended product **8** has so far only been prepared based on other psoralen intermediates, for example, 5‐formyl‐8‐methoxypsoralen.^25^ In addition, 5‐nitro‐8‐methoxypsoralen (**9**) was prepared under nitration conditions according to Yue et al.^26^

**Scheme 4:**
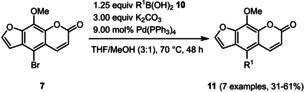
Suzuki synthesis of 5‐(hetero)aryl substituted 8‐methoxypsoralens **11**.

Starting from compound **7**, several functionalization reactions such as Suzuki, Sonogashira and Heck coupling could be established for variation of the aryl substituent at position 5. Using Suzuki coupling, various acceptors and donors were introduced under conditions shown in Scheme 4. With Pd(PPh_3_)_4_ as the standard catalyst and potassium carbonate as the base, seven different novel 5‐substituted 8‐methoxypsoralens (**11 a**–**g**) have been synthesized.

Specific deviations from standard conditions had to be implemented for coupling of the pyridine derivative (Table 1, entry 4). 4‐Pyridinylboronic acid (**10 d**) possesses ligand properties that can inhibit reductive elimination and reduce the amount of the active catalyst in the final step of the Suzuki coupling cycle. This assumption is confirmed by the fact that an increase in catalyst loading led to higher yields. Moreover, in a particular case higher yields were achieved using tri‐*tert*‐butylphosphonium tetrafluoroborate as a ligand and Pd(dba)_2_ as a catalyst with potassium hydroxide as a base (Table 1, entry 2). However, applying these conditions to the other boronic acids did not lead to increased yields. By using 4‐carboxyphenylboronic acid (**10 g**), it was necessary to switch to completely different conditions (Pd_2_(dba)_3_, SPhos as a ligand, and KF as a base) to reach conversion (Table 1, entry 7).

**Table 1:** Suzuki synthesis of 5‐(hetero)aryl substituted 8‐methoxypsoralens **11**.

Entry	Boronic acid, R^1^B(OH)_2_ **10**		5‐Substituted 8‐methoxypsoralen **11** (yield)^[a]^	
**1**	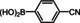	**10 a**	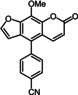	**11 a** (59 %)
**2**	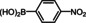	**10 b**	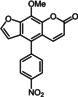	**11 b** (61 %; 99 %^[b]^)
**3**	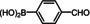	**10 c**	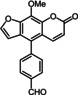	**11 c** (43 %)
**4** ^[c]^	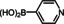	**10 d**	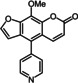	**11 d** (50 %)
**5**	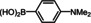	**10 e**	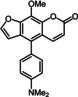	**11 e** (59 %)
**6**	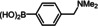	**10 f**	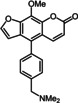	**11 f** (31 %)
**7** ^[d]^	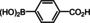	**10 g**	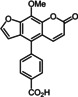	**11 g** (54 %)

[a] Yields after chromatography on silica gel. [b] 1.50 mol % Pd(dba)_2_, 3.00 equivalents KF, 3.00 mol % [(*t*Bu)_3_PH][BF_4_]. [c] 18.0 mol % Pd(PPh_3_)_4_. [d] 1.10 equivalents R^1^B(OH)_2_, 20.0 mol % Pd_2_(dba)_3_, 3.00 equivalents KF, 26.0 mol % SPhos.

**Figure 2:**
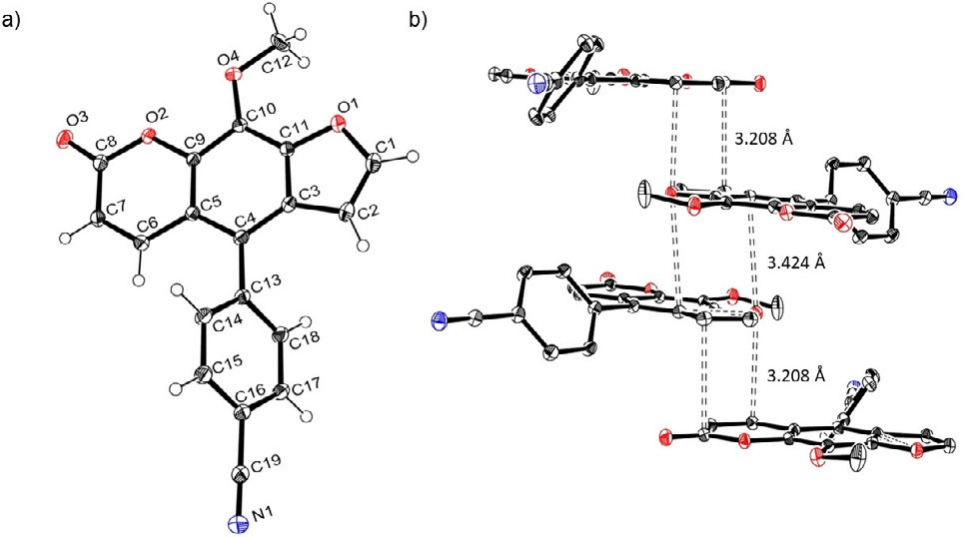
a) X‐ray structure of compound **11 a** (thermal ellipsoids for N, O, and C shown at 40 % probability); b) Crystal packing of compound **11 a** with shortest interplanar distances between furan and pyrone moieties.

Additionally, it was possible to corroborate the structure of 5‐(hetero)aryl substituted 8‐methoxypsoralens **11** by an X‐ray crystal structure analysis of compound **11 a** (Figure 2).^27^ The brownish block‐shaped compound crystallizes with a centrosymmetric arrangement in the monoclinic space group *P*2_1_/*c*. The cyanophenyl moiety is twisted with the psoralen core by an angle of 57.01(4)°. Furthermore, analysis of the crystal packing reveals that the psoralen cores are self‐oriented in a planar fashion. Thereby two furan (3.424 Å) and two pyrone units (3.708 Å) are mutually stacked on top of each other. The plane distance between furan and pyrone (3.208 Å) is even shorter, rationalizing π‐stacking of the molecules.

**Scheme 5:**
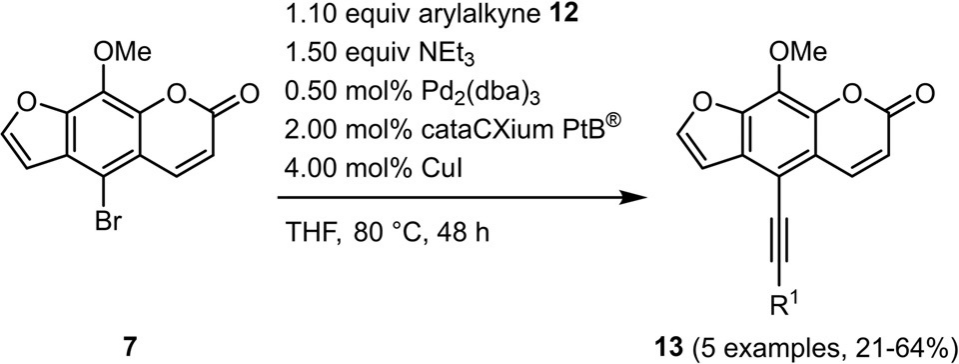
Sonogashira synthesis of 5‐(hetero)aryl alkynyl substituted 8‐methoxypsoralens **13**.

**Scheme 6:**
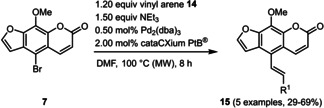
Heck synthesis of 5‐(hetero)aryl vinyl substituted 8‐methoxypsoralens **15**.

Subsequently, various ethynyl and vinyl aryl substrates **12** and **14** were coupled to the 5‐position of 8‐methoxypsoralen under Sonogashira and Heck conditions with the same catalyst and ligand system consisting of Pd_2_(dba)_3_ and cataCXium PtB to give the corresponding 5‐(hetero)aryl alkynyl substituted 8‐methoxypsoralens **13** and 5‐(hetero)aryl vinyl substituted 8‐methoxypsoralens **15** (Scheme 5 and 6). For both series four examples with electron‐withdrawing groups and one example with a dimethylamino group as electron‐donating substituents were synthesized (Table 2 and 3). As previously shown for the Suzuki coupling, double amounts of catalyst and ligand were used for successful transformation of pyridyl derivatives (Table 2 and 3, entries 4). All obtained psoralen derivatives **11**, **13**, and **15** were purified by precipitation or column chromatography and then recrystallized in various solvents. Structures and purity were confirmed by ^1^H, ^13^C NMR, mass spectrometry, high resolution mass spectrometry, HPLC and elemental analysis.

**Table 2:** Sonogashira synthesis of 5‐(hetero)aryl alkynyl substituted 8‐methoxypsoralens **13**.

Entry	Arylalkyne **12**		5‐Substituted 8‐methoxypsoralen **13** (yield)^[a]^	
**1**	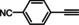	**12 a**	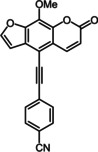	**13 a** (21 %)
**2**	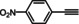	**12 b**	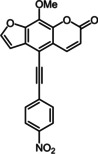	**13 b** (25 %)
**3**	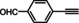	**12 c**	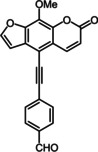	**13 c** (45 %)
**4** ^[b]^	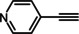	**12 d**	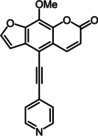	**13 d** (64 %)
**5**	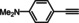	**12 e**	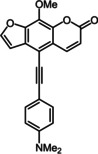	**13 e** (63 %)

[a] Yields after chromatography on silica gel. [b] 1.00 mol % Pd_2_(dba)_3,_ 4.00 mol % cataCXium PtB (2‐(di‐*tert*‐butyl‐phosphino)‐1‐phenyl‐1*H*‐pyrrole).

**Table 3:** Heck synthesis of 5‐(hetero)aryl vinyl substituted 8‐methoxypsoralens **15**.

Entry	Vinyl (hetero)aryene **14**		5‐Substituted 8‐methoxypsoralen **15** (yield)^[a]^	
**1**	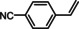	**14 a**	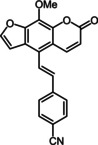	**15 a** (39 %)
**2**	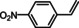	**14 b**	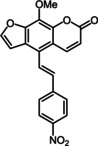	**15 b** (69 %)
**3**	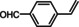	**14 c**	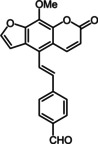	**15 c** (69 %)
**4** ^[b]^	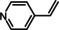	**14 d**	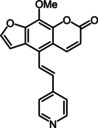	**15 d** (29 %)
**5**	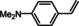	**14 e**	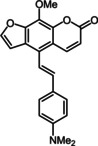	**15 e** (57 %)

[a] Yields after chromatography on silica gel. [b] 1.00 mol % Pd_2_(dba)_3_, 4.00 mol % cataCXium PtB (2‐(di‐*tert*‐butyl‐phosphino)‐1‐phenyl‐1*H*‐pyrrole).

**Figure 3:**
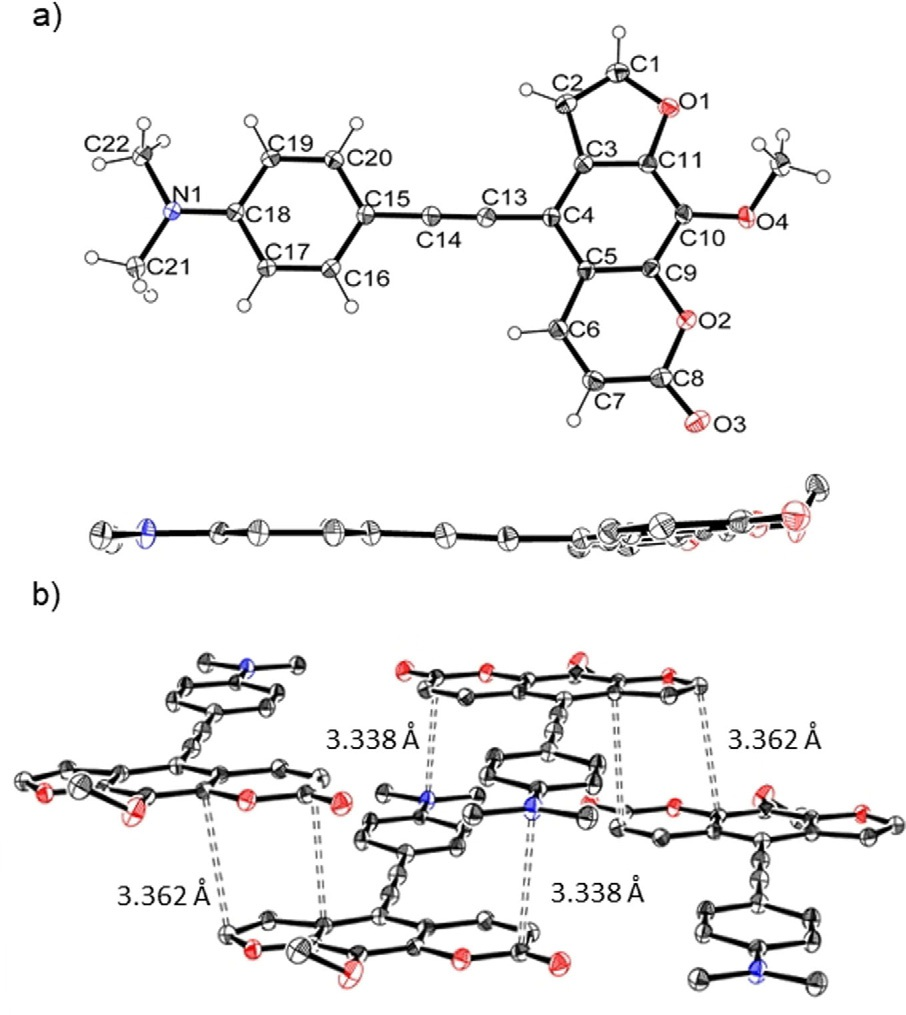
a) X‐ray structure of compound **13 e** (thermal ellipsoids for N, O, and C shown at 40 % probability), top: top‐view, bottom: side‐on‐view; b) Crystal packing of compound **13 e** with shortest interplanar distances between furan and pyrone moieties and between pyrone and *N*,*N*‐dimethyl aniline moieties.

An X‐ray crystal structure analysis of alkyne‐linked compound **13 e** was obtained.^27^, ^28^ The yellow acicular crystals with the monoclinic space group *P*2_1_/*c* crystallize planar due to the rigid character of the ethynyl bridge (Figure 3). The centrosymmetric arrangement, supported by the short interplanar distances between the molecules (<3.4 Å), enables a close interaction of the molecules in the crystalline solid state. These interactions cause a pronounced π‐stacking, which appears to be relevant for the observed solid state luminescence (vide infra).

### Photophysical properties

Most of the synthesized psoralen derivatives **8**, **9**, **11**, **13**, and **15** are novel chromophores and have not been photophysically investigated so far. A peculiar aspect is that psoralen by its furo (donor) and α‐pyrone (acceptor) anellation represents a donor—acceptor chromophore per se, which acts electronically amphiphilic. This means 8‐MOP can adopt a donor and acceptor function depending on the electronic nature of the 5‐substituent. Based on a library of 5‐substituted 8‐methoxypsoralens systematic studies of the absorption and emission properties were conducted. The relative fluorescence quantum yield *Φ*_F_ was determined with Coumarin 30 as a standard.^28^

All 5‐acceptor‐8‐methoxypsoralens **8**, **9**, and **11** exhibit a shoulder as the longest wavelength absorption between 355 and 412 nm, with molar absorption coefficients, *ϵ*, between 2700 and 6700 Lmol^−1^ cm^−1^ (Table 4). The longest wavelength bands of cyano and nitro psoralens **8** and **9** are more bathochromically shifted than those of aryl‐substituted psoralens **11**, plausibly rationalized by the expected twist between the aryl moiety and the psoralen core, resulting in a weaker orbital overlap.

**Table 4:** Selected photophysical properties of 5‐substituted 8‐methoxy‐psoralens **8**, **9** and **11**.

Compound	*λ*_max,abs_ [nm]^[a]^ (*ϵ* [m ^−1^ cm^−1^])	*λ*_max,em_ [nm]^[b]^ (*Φ*_F_ [a.u.])	Stokes shift Δ$\tilde{\nu }$ [cm^−1^]^[c]^
**8**	355 (6700), 380 (3300sh)	–	–
**9**	394 (7400), 412 (5200sh)	–	–
**11 a**	310 (16 400), 359 (3600sh)	–	–
**11 b**	309 (16 100), 371 (6700sh)	–	–
**11 c**	313 (18 300), 365 (3900sh)	–	–
**11 d**	307 (15 200), 355 (3100sh)	–	–
**11 e**	327 (8000sh), 380 (5100)	557 (0.27)	8400
**11 f**	310 (5600), 360 (1000sh)	–	–
**11 g**	311 (15 600), 359 (2700sh)	–	–

[a] Recorded in CH_2_Cl_2,_ *c*(**8**), *c*(**9**), *c*(**11**)*=*10^−5^ m at *T=*293 K. [b] Recorded in CH_2_Cl_2_, *c*(**8**), *c*(**9**), *c*(**11**)*=*10^−7^ m at *T=*293 K, relative quantum yields were determined with Coumarin 30 as a standard in acetonitrile (*Φ*_F_=0.67^28^). [c] Δ$\tilde{\nu }$ =$\;\frac{1}{\lambda_{max,\;abs}}-\;\frac{1}{\lambda_{max,\;em}}$

**Figure 4:**
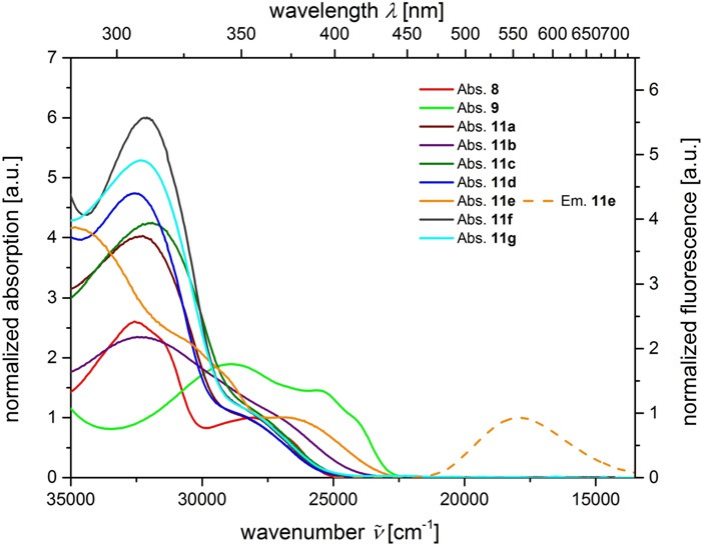
Normalized UV/Vis absorption (recorded in CH_2_Cl_2_, *T=*293 K,. *c*(**8**), *c*(**9**), *c*(**11**)*=*10^−5^ m, bold lines) and emission bands (recorded in CH_2_Cl_2_, *T=*293 K, *c*(**8**), *c*(**9**), *c*(**11**)*=*10^−7^ m, dashed lines) of compounds **8**, **9** and **11**.

Only the donor‐substituted psoralen **11 e** has a distinct longest wavelength band at *λ*_max,abs_=380 nm (*ϵ*=5100 Lmol^−1^ cm^−1^, Table 4, entry 4, Figure 4). Furthermore, only this compound fluoresces in dichloromethane with a substantial fluorescence quantum yield of 27 %.

**Figure 5:**
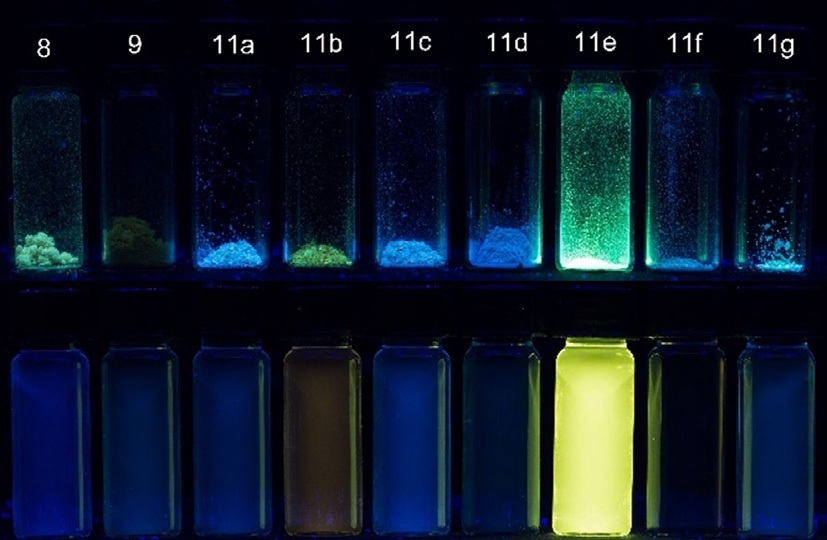
Fluorescence of psoralen derivatives **8**, **9** and **11** in solid state (upper row) and in dichloromethane (lower row, *c*(**8**), *c*(**9**), *c*(**11**)*=*10^−7^ m, hand‐held UV‐Lamp, *λ_exc_=*365 nm).

All other 8‐methoxypsoralens **8**, **9**, and **11** only fluoresce in the solid state, however, only very weakly in solution (Figure 5). Strongly acceptor‐ (**9**, **11 b**) and donor‐substituted derivatives (**11 e**) possess redshifted absorptions that correlate to HOMO–LUMO transitions as supported by TD–DFT calculations (vide infra and for further details, see Supporting Information).

**Figure 6:**
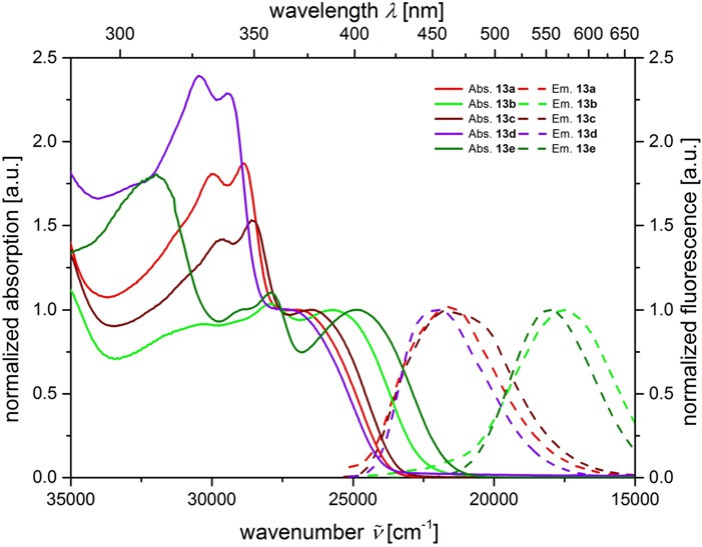
Normalized UV/Vis absorption (recorded in CH_2_Cl_2_, *T=*293 K, *c*(**13**)*=*10^−5^ m, bold lines) and emission bands (recorded in CH_2_Cl_2_, *T=*293 K, *c*(**13**)*=*10^−7^ m, dashed lines) of compounds **13**.

Ethynyl substituted psoralens **13** differ from compounds **11** by pronounced redshifted maxima of the longest wavelength absorption bands in UV/Vis spectra (Table 5, Figure 6). Also the molar extinction coefficients are substantially higher (between 8800 and 20 000 Lmol^−1^ cm^−1^). Compounds **13 b**, with the strongest acceptor, and **13 e**, with the strongest donor, exhibit the largest bathochromic shift probably due to a charge transfer state. Interestingly, the latter is the first psoralen derivative absorbing light in the visible.

**Table 5:** Selected photophysical properties of 5‐substituted 8‐methoxy‐psoralens **13**.

Compound	*λ*_max,abs_ [nm]^[a]^ (*ϵ* [m ^−1^ cm^−1^])	*λ*_max,em_ [nm]^[b]^ (*Φ*_F_ [a.u.])	Stokes shift Δ$\tilde{\nu }$ [cm^−1^]^[c]^
**13 a**	346 (24 800), 374 (13 100)	474 (0.07)	5600
**13 b**	358 (19 200), 388 (18 400)	565 (0.03)	8100
**13 c**	350 (29 700), 377 (19 300)	464 (0.09)	5000
**13 d**	341 (20 000), 368 (8800)	465 (0.09)	5900
**13 e**	359 (22 100), 403 (20 000)	553 (0.28)	6700

[a] Recorded in CH_2_Cl_2,_ *c*(**13**)*=*10^−5^ m at *T=*293 K. [b] Recorded in CH_2_Cl_2_, *c*(**13**)*=*10^−7^ m at *T=*293 K, relative quantum yields were determined with Coumarin 30 as a standard in acetonitrile (*Φ*_F_=0.67). [c] Δ$\tilde{\nu }$ =$\;\frac{1}{\lambda_{max,\;abs}}-\;\frac{1}{\lambda_{max,\;em}}$

**Figure 7:**
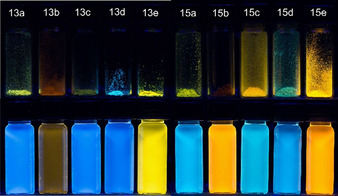
Fluorescence of psoralen derivatives **13** (left) and **15** (right) in solid state (upper row) and dichloromethane (lower row, *c*(**13**), *c*(**15**)*=*10^−7^ m, hand‐held UV‐Lamp, *λ_exc_=*365 nm).

Compared to compounds **8**, **9**, and **11** the relative fluorescence quantum yields of compounds **13** in solution increase significantly with values between 3 and 28 % (Table 5, Figure 7). The nitro‐substituted psoralen **13 b** fluoresces the least, which is due to its competitive deactivation of the electronic excitation energy by predissociation.^29^

**Figure 8:**
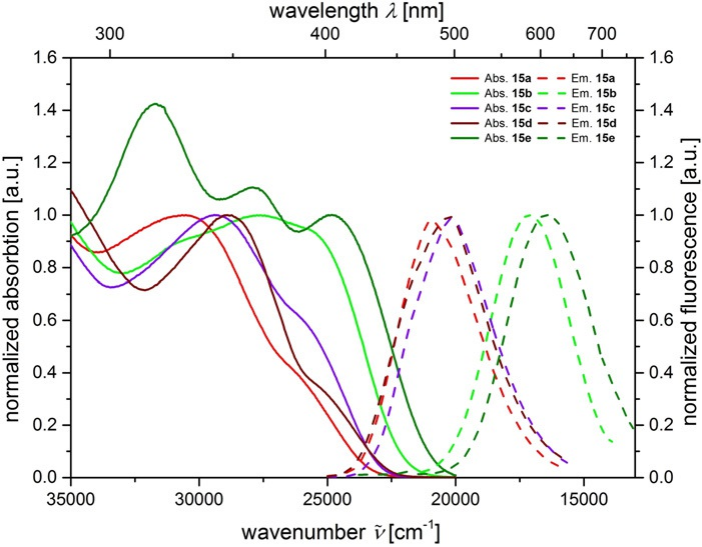
Normalized UV/Vis absorption (recorded in CH_2_Cl_2_, *T=*293 K, *c=*10^−5^ m, bold lines) and emission bands (recorded in CH_2_Cl_2_, *T=*293 K, *c=*10^−7^ m, dashed lines) of compounds **15**.

The vinyl‐substituted psoralen derivatives **15** possess most redshifted longest wavelength absorption bands in these psoralen series (Table 6, Figure 8). Additionally, the longest wavelength absorption bands of compounds **15 a**–**d** can only be recognized as weak shoulders (Figure 8).

**Table 6:** Selected photophysical properties of 5‐substituted 8‐methoxy‐psoralens **15**.

Compound	*λ*_max,abs_ [nm]^[a]^ (*ϵ* [m ^−1^ cm^−1^])	*λ*_max,em_ [nm]^[b]^ (*Φ*_F_ [a.u.])	Stokes shift Δ$\tilde{\nu }$ [cm^−1^]^[c]^
**15 a**	329 (13 400), 385 (9300sh)	477 (0.09)	5000
**15 b**	362 (21 200), 396 (20 600sh)	583 (0.13)	8100
**15 c**	342 (24 400), 386 (14 700sh)	498 (0.04)	5800
**15 d**	327 (21 700), 378 (7200sh)	497 (0.05)	6300
**15 e**	358 (18 100), 403 (16 300)	553 (0.13)	6700

[a] Recorded in CH_2_Cl_2,_ *c*(**15**)*=*10^−5^ m at *T=*293 K. [b] Recorded in CH_2_Cl_2_, *c*(**15**)*=*10^−7^ m at *T=*293 K, relative quantum yields were determined with Coumarin 30 as a standard in acetonitrile (*Φ*_F_=0.67^28^). [c] Δ$\tilde{\nu }$ =$\;\frac{1}{\lambda_{max,\;abs}}-\;\frac{1}{\lambda_{max,\;em}}$

**Figure 9:**
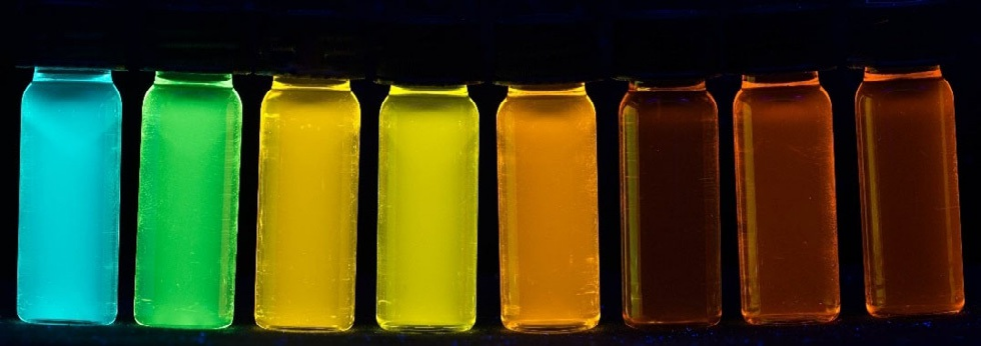
Fluorescence of compound **15 e** in solvents of different polarity (from left to right: cyclohexane, toluene, chloroform, ethyl acetate, dichloromethane, dimethyl sulfoxide, *N*,*N*‐dimethylformamide, acetonitrile; *λ_exc_=*365 nm).

Despite the mostly dissociative nature of the nitro group, compound **15 b** unexpectedly fluoresces with a substantial fluorescence quantum yield of 13 % (Table 6, entry 2). All psoralen compounds **15** particularly fluoresce in the solid state. As in the series **11** and **13** dimethylamino‐substituted psoralens reveal pronounced positive emission solvatochromicity. Most remarkably these compounds cover a spectral range from blue emission in cyclohexane to orange‐red emission in acetonitrile (Figure 9). The solvatochromicity of compound **15 e** was studied in more detail. Therefore, absorption and emission spectra were recorded in solvents of different polarity. The absorption solvatochromicity in the range from 392 to 413 nm turns out to be weak. In comparison, the emission solvatochromicity with a bathochromic shift of 499 to 684 nm is very strong (for details, see Supporting Information).

This peculiar behavior originates from the change of dipole moment of the molecule upon excitation by UV light and the concomitant relaxation of surrounding solvent molecules.^30^ Quantitative calculation of this dipole moment change can be performed with the Lippert–Mataga model.^31^ Initially, the orientation polarizability Δ*f* of different solvents is determined according to the following equation [Eq. (1)]:

(1) $$\Delta f=\;\frac{\epsilon_{r}-1}{2\epsilon_{r}+1}-\frac{n^{2}-1}{2n^{2}+1}$$

*ϵ_r_* describes the relative permittivity and *n* the refractive index of the respective solvent.^30^ Subsequently the orientation polarizability Δ*f* can be plotted against the Stokes shift Δ$\tilde{\nu }$ The regression correlates with an excellent goodness of fit (*r* ^*2*^=0.98, for further details, see Supporting Information).

The Stokes shift can be described using the Lippert–Mataga equation [Eq. (2)] by the change of the dipole moment from the ground to the excited state.

(2) $${\tilde{\nu }}_{a}-{\tilde{\nu }}_{f}=\;\frac{2\Delta f}{4\pi \epsilon_{o}hca^{3}}{\left(\mu_{E}-\mu_{G}\right)}^{2}+const$$

The parameters $\tilde{\nu }$_a_ and $\tilde{\nu }$_f_ define the absorption and emission maxima (in cm^−1^). *ϵ_o_* is the vacuum permittivity constant (8.8542⋅10^−12^ AsV^−1^m^−1^) and *h* is the Planck's constant (6.6256⋅10^−34^ Js). Furthermore, *c* describes the speed of light (2.9979⋅10^10^ cms^−1^) and *a* the radius of the solvent cavity which occupies the investigated molecule. Finally, *μ_E_* and *μ_G_* refer to the dipole moment in the ground and excited state. The parameter *a* could be determined by assuming a spherical dipole using DFT calculations in the optimized ground state. This Onsager radius *a* is 5.83 Å (5.83⋅10^−10^ m). With the determined parameters and constants, for compound **13 e** a change of dipole moment Δ*μ* of 13 D (4.28⋅10^−29^ Cm) results. For the other donor‐substituted psoralens **11 e** and **15 e**, the change in the dipole moment Δ*μ* values amount to 12 D (3.88⋅10^−29^ Cm) and 19 D (6.43⋅10^−29^ Cm), respectively. The differences in the change of dipole moment indicate the increase in charge transfer character with extension of the π‐system.

**Figure 10:**
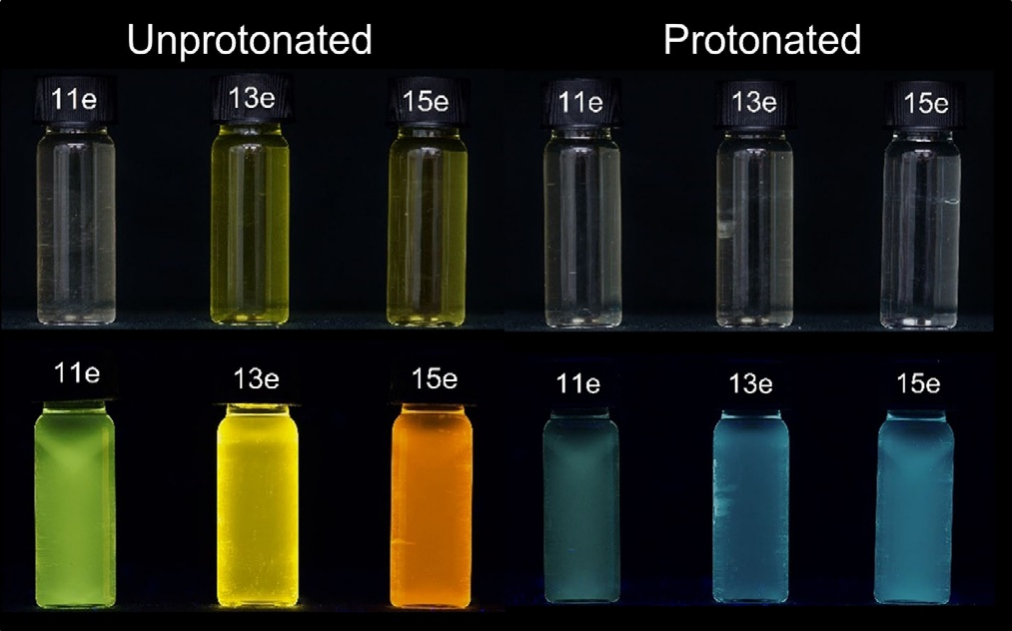
Psoralen derivates **11 e**, **13 e** and **15 e** in dichloromethane (left) and with dichloromethane and trifluoracetic acid (right) at daylight (top row, *c=*10^−5^ m) and under the hand‐held UV lamp (top bottom, *c=*10^−5^ m, *λ_exc_=*365 nm).

Protonation of chromophores **11 e**, **13 e** and **15 e** in dichloromethane reveals another photophysical effect. The protonation of the compounds significantly changes the absorption and emission behavior (Figure 10). Upon addition of trifluoroacetic acid the solutions’ yellowish color disappears with concomitant fluorescence quenching. Upon addition of triethylamine this acidochromicity can be reversed and luminescence returns.

**Figure 11:**
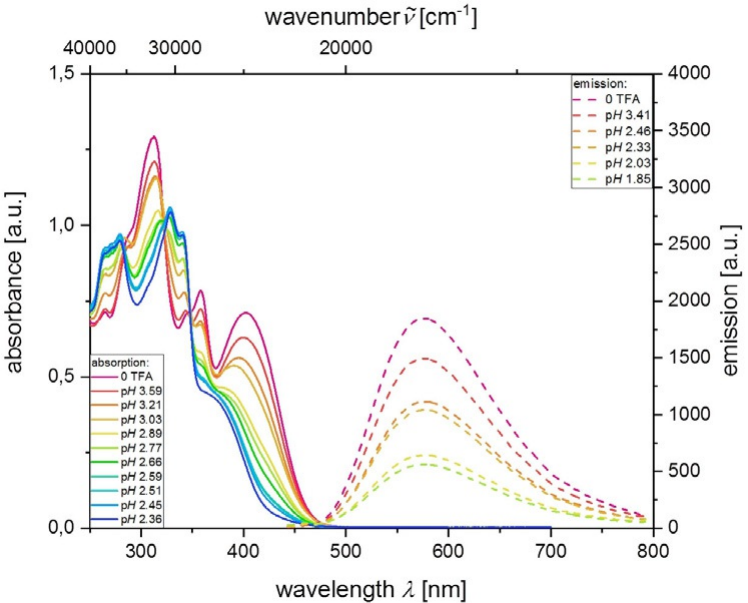
Absorption and emission spectra of **13 e** in the presence of increasing amounts of trifluoracetic acid (recorded in CH_2_Cl_2,_ *c*(**13 e**)=2.78⋅10^−4^ m (absorption), *c=*1.11⋅10^−4^ m (emission; *λ_exc_=*403 nm) *T=*293 K).

Thereby, it was also possible to determine the p*K*_a_ value of the chromophores **11 e**, **13 e** and **15 e**. Assuming complete dissociation of trifluoroacetic acid in dichloromethane the p*K*_a_ values were determined by recording the absorption spectra at different p*H* values. For compound **13 e** a hypsochromic shift of the absorption maximum at 403 nm to a shoulder at 371 nm was monitored (Figure 11). For **13 e‐H^+^** a p*K*_a_ value of 2.81 could be determined (for experimental details, see Supporting Information). Likewise the aryl derivative **11 e** gives a p*K*_a_ of 3.05, whereas for the styryl derivative **15 e** a p*K*_a_ of 3.28 can be determined.

In addition, monitoring the fluorescence quenching by trifluoroacetic acid the p*K*_a_ values of the chromophores **11 e**, **13 e** and **15 e** were alternatively determined from the resulting Stern–Volmer^32^ plots revealing linear correlations of the fluorescence intensities *F_0_*/*F* with the concentration of the trifluoroacetic acid solution *c*(TFA) (for details, see Supporting Information). The determined Stern–Volmer constant *K_sv_* of compound **13 e** is 155.93 Lmol^−1^, corresponding to a p*K*_a_ value of 2.15, which is in good agreement with the p*K*_a_ value determined by absorption spectroscopy. The p*K*_a_ values by Stern–Volmer plots of compounds **11 e‐H^+^** and **15 e‐H^+^** were also determined to 3.31 and 3.45, respectively, corresponding very well with the previously determined values by absorption spectroscopy. The obtained values are typical of *para*‐substituted amines,^33^ therefore it can be assumed that the protonation occurs at the dimethylamino nitrogen atom, which is additionally supported by NMR spectra of the unprotonated and protonated species (for further details, see Supporting Information).

The comparison of the three *p*‐dimethylamino phenyl derivatives **11 e**, **13 e**, and **15 e**, giving the highest fluorescence quantum yields in dichloromethane within all three consanguineous series, reveals that the emission maxima lie in a very narrow margin between 553 and 557 nm. This accounts for a very similar electronic structure of the vibrationally relaxed excited state. For the alkynyl derivative **13 e** the solid state spectrum was detected at 557 nm, that is, at a very similar energy. In addition the chromophores **11 e**, **13 e**, and **15 e** were embedded in PMMA (polymethylmethacrylate) films at 1 wt % and their emission maxima appear at 500 (**11 e**), 523 (**13 e**), and 547 nm (**15 e**), that is, hypsochromically shifted in comparison to the solution emission maxima. This slight blue shift can be rationalized by the polarity effect of the PMMA matrix (for spectra, see Supporting Information).

### Calculated electronic structure

For gaining an insight in the electronic structure of these T‐shaped 8‐methoxy psoralen chromophores, in which the psoralen moiety and the 5‐substituents adopt rectangular orientations, TD–DFT calculations were performed for the chromophores **11 a**, **11 e**, **13 a**, **13 e**, **15 a** and **15 e**. The geometry of the electronic ground state structures was optimized using Gaussian 09,^34^, ^35^ with the PBE1PBE^35^ functional and the Pople 6–311G(d,p)^36^ base set. Since all photophysical measurements were carried out in dichloromethane solutions, the polarizable continuum model (PCM) with dichloromethane as a solvent was used.^37^ Geometry optimization shows that the torsional angle between the aryl moiety and the psoralen core lies between 55 and 57° for all molecules synthesized by Suzuki coupling. This is in good agreement with the torsional angles extracted from crystal structure analyses. Molecules synthesized by Sonogashira coupling are essentially coplanar due to the ethynyl bridge. The Heck derivatives possess torsional angles of the styryl substituents between 33 to 34°.

Starting from the geometry optimized structures, the lowest energy electronic transitions of chromophores **11 a**, **11 e**, **13 a**, **13 e**, **15 a** and **15 e** were calculated on the TD–DFT level of theory with the Pople 6–21G basis set (Table 7).^38^ The comparison considers in each series the cyano‐substituted (acceptor) and the dimethylamino‐substituted (donor) derivatives. The calculations confirm that the experimentally assessed longest wavelength absorption bands (maxima and shoulders) can be clearly assigned to HOMO–LUMO transitions.

**Table 7:** TD‐DFT calculations (PBE1PBE/6‐21G) of the UV/Vis absorption maxima of **11 a** <**11 e** <**13 a** <**13 e** <**15 a** and **15 e** using PCM with dichloromethane as solvent.

	*λ*_max,abs_ [nm]^[a]^ (*ϵ* [m ^−1^ cm^−1^])	*λ*_max,calcd_ [nm]	Dominant contributions	Oscillator strength
**11 a**	359 (3600sh)	355	HOMO→LUMO (96 %)	0.2066
	310 (16 400)	313	HOMO→LUMO+1 (93 %)	0.1376
**11 e**	380 (5100)	400	HOMO→LUMO (99 %)	0.2105
	327 (8000sh)	319	HOMO−1→LUMO (91 %)	0.0147
**13 a**	374 (13 100sh)	397	HOMO→LUMO (97 %)	0.8154
	346 (24 800)	329	HOMO→LUMO+1 (74 %)	0.3676
**13 e**	403 (20 000)	442	HOMO→LUMO (98 %)	0.6549
	359 (22 100)	337	HOMO−1→LUMO (66 %)	0.1754
**15 a**	385 (9300sh)	403	HOMO→LUMO (98 %)	0.7491
	329 (13 400)	337	HOMO→LUMO+1 (87 %)	0.2324
**15 e**	403 (16 300)	445	HOMO→LUMO (99 %)	0.6721
	358 (18 100)	337	HOMO→LUMO+1 (50 %)	0.2719

8‐MOP as an electronic amphiphile can adopt either donor or acceptor functionality depending on the remote substituent's electronic nature. This can be clearly visualized by the molecules’ FMOs, reflecting the Franck–Condon transition of the longest wavelength absorption band.

**Figure 12:**
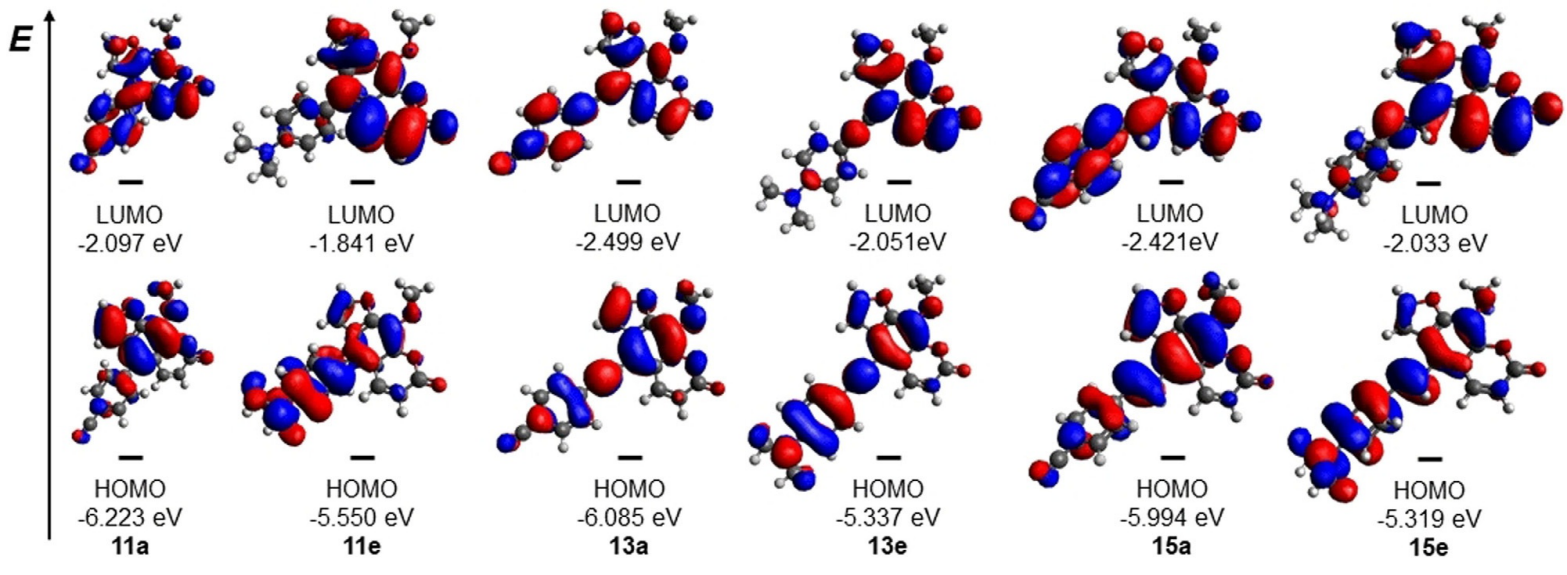
Selected Kohn–Sham FMOs of psoralens **11 a** <**11 e** <**13 a** <**13 e** <**15 a** and **15 e** (with PBE1PBE/6‐311G(d,p) and PCM with dichloromethane as solvent).

The calculated frontier molecule orbitals (FMO) indicate that the coefficient densities in HOMOs predominantly reside on the 5‐substituents. The LUMOs, however, predominantly localize coefficient density on the psoralen units (Figure 12). The dominance of the HOMO–LUMO transitions clearly rationalize the charge transfer character of these dominant low energy absorption bands, as well as the pronounced emission solvatochromicity. In addition the T‐shape of two constituting subchromophores, biaryl, tolane, and stilbene, and psoralen enables the design of rectangular excited state coupled chromophores with considerable alteration of dipole moment orientation. Furthermore the tunability of absorption and emission characteristics makes these novel chromophores interesting candidates for photo‐induced DNA‐crosslinking.

## Conclusions

A novel route from pyrogallol to 5‐bromo‐8‐methoxypsoralen was established. Several new chromophores with functional donor and acceptor groups were synthesized by different cross‐coupling methodologies, such as Suzuki, Sonogashira or Heck reactions. These psoralen series absorb at wavelengths around 400 nm and possess highly interesting emission properties with relative fluorescence quantum yields of up to 28 %. Besides pronounced positive emission solvatochromicity reversible fluorescence quenching by acidochromicity can be assessed. Experimentally the highly polar nature of the excited state was supported by determining the change of dipole moments according to the Lippert–Mataga model. The acidochromicity and protic emission quenching was quantitatively investigated by determining p*K_a_* values of these psoralen chromophores by absorption photometry and by Stern–Volmer plots. TD–DFT calculations using the PBE1PBE functional can successfully applied to elucidate the nature of the longest wavelength absorption bands.

With the embedded multifunctionality these novel psoralen derivatives are promising candidates for PUVA therapy at lower energies. Their bathochromic absorption does not require the use of ultraviolet light, potentially also daylight suffices. In addition, these sensitivity to polar and protic environments encourage to scout for applications in biophysical analytics as well as theranostic agents.^39^

## Experimental Section

All experimental details, such as preparations, typical procedures, and all ^1^H and ^13^C NMR spectra, absorption and emission spectra, solvatochromicity and acidochromicity studies as well as crystal structures and quantum chemical calculations are included in the Supporting Information.

## Conflict of interest

The authors declare no conflict of interest.

## Supporting information

As a service to our authors and readers, this journal provides supporting information supplied by the authors. Such materials are peer reviewed and may be re‐organized for online delivery, but are not copy‐edited or typeset. Technical support issues arising from supporting information (other than missing files) should be addressed to the authors.

Supplementary
