# Supplementary material for: Electronic Finetuning of 8‐Methoxy Psoralens by Palladium‐Catalyzed Coupling: Acidochromicity and Solvatochromicity
Source: Chemistry. 2020 May 29;26(36):8064–75. doi: 10.1002/chem.201905676 (PMC7383860; doi:10.1002/chem.201905676)
Supplement: Supplementary file 1 — Supplementary [file CHEM-26-8064-s001.pdf]

Chemistry–A European Journal

Supporting Information

Electronic Finetuning of 8-Methoxy Psoralens by Palladium-Catalyzed Coupling: Acidochromicity and Solvatochromicity

Sarah R. Geenen,[a] Lysander Presser,[a] Torsten Hölzel,[b] Christian Ganter,[b] and Thomas J. J. Müller*[a]

Supporting Information

Table of Contents

1. Synthesis of starting materials 5
1.1. 2-Methoxybenzene-1,3-diol[1] (**2**) 5
1.2. 7-Hydroxy-8-methoxycoumarin (**3**) 6
1.3. 7-(2,2-Diethoxyethoxy)-8-methoxy-2*H*-chromen-2-one (**4**) 6
1.4. 2-((8-Methoxy-2-oxo-2*H*-chromen-7-yl)oxy)acetaldehyde (**5**) 7
1.5. 8-Methoxypsoralen[2] (**6**) 8
1.6. 5-Bromo-8-methoxypsoralen (**7**) 8
2. Synthesis of 5-substituted 8-methoxypsoralens 9
2.1. 5-Cyano-8-methoxypsoralen (**8**) 9
2.2. 5-Nitro-8-methoxypsoralen[3] (**9**) 10
2.1. General procedure: Suzuki coupling 11
2.1.1. 4-(9-Methoxy-7-oxo-7*H*-furo[3,2-*g*]chromen-4-yl)benzonitrile (**11a**) 12
2.1.2. 9-Methoxy-4-(4-nitrophenyl)-7*H*-furo[3,2-*g*]chromen-7-one (**11b**) 13
2.1.3. 4-(9-Methoxy-7-oxo-7*H*-furo[3,2-*g]*chromen-4-yl)benzaldehyde (**11c**) 14
2.1.4. 9-Methoxy-4-(pyridin-4-yl)-7*H*-furo[3,2-*g*]chromen-7-one (**11d**) 15
2.1.5. 4-(4-(Dimethylamino)phenyl)-9-methoxy-7*H*-furo[3,2-*g*]chromen-7-one (**11e**) 15
2.1.6. 4-(4-((Dimethylamino)methyl)phenyl)-9-methoxy-7*H*-furo[3,2-*g*]chromen-7-one (**11f**) 16
2.1.7. 4-(9-Methoxy-7-oxo-7*H*-furo[3,2-*g*]chromen-4-yl)benzoic acid (**11g**) 17
2.2. General procedure: Sonogashira coupling 18
2.2.1. 4-((9-Methoxy-7-oxo-7*H*-furo[3,2-*g*]chromen-4-yl)ethynyl)benzonitrile (**13a**) 18
2.2.2. 9-Methoxy-4-((4-nitrophenyl)ethynyl)-7*H*-furo[3,2-*g*]chromen-7-one (**13b**) 19
2.2.3 4-((9-Methoxy-7-oxo-7*H*-furo[3,2-*g*]chromen-4-yl)ethynyl)benzaldehyde (**13c**) 20
2.2.4. 9-Methoxy-4-(pyridin-4-ylethynyl)-7*H*-furo[3,2-*g*]chromen-7-one (**13d**) 21
2.2.5 4-((4-(Dimethylamino)phenyl)ethynyl)-9-methoxy-7*H*-furo[3,2-*g*]chromen-7-one (**13e**) 21
2.3. General procedure: Heck coupling 23
2.3.1. (*E*)-4-(2-(9-Methoxy-7-oxo-7*H*-furo[3,2-*g*]chromen-4-yl)vinyl)benzonitrile (**15a**) 23
2.3.2. (*E*)-9-Methoxy-4-(4-nitrostyryl)-7*H*-furo[3,2-*g*]chromen-7-one (**15b**) 24
2.3.3. (*E*)-4-(2-(9-Methoxy-7-oxo-7*H*-furo[3,2-*g*]chromen-4-yl)vinyl)benzaldehyde (**15c**) 25
2.3.4. (*E*)-9-Methoxy-4-(2-(pyridin-4-yl)vinyl)-7*H*-furo[3,2-*g*]chromen-7-one (**15d**) 25
2.3.5. (*E*)-4-(4-(Dimethylamino)styryl)-9-methoxy-7*H*-furo[3,2-*g*]chromen-7-one (**15e**) 26
3. NMR spectra 28
3.1. NMR spectra of starting materials 28
3.1.1. 2-Methoxybenzene-1,3-diol (**2**) 28
3.1.2. 7-Hydroxy-8-methoxycoumarin (**3**) 29
3.1.3. 7-(2,2-Diethoxyethoxy)-8-methoxy-2*H*-chromen-2-one (**4**) 30
3.1.4. 2-((8-Methoxy-2-oxo-2*H*-chromen-7-yl)oxy)acetaldehyde (**5**) 31

3.1.5. 8-Methoxypsoralen (**6**) ... 32
3.1.6. 5-Bromo-8-methoxypsoralen (**7**) ... 33
3.2. NMR spectra of 5-substituted 8-methoxypsoralens ... 34
3.2.1. 5-Cyano-8-methoxypsoralen (**8**) ... 34
3.2.2. 5-Nitro-8-methoxypsoralen (**9**) ... 35
3.2.3. 4-(9-Methoxy-7-oxo-7*H*-furo[3,2-*g*]chromen-4-yl)benzonitrile (**11a**) ... 36
3.2.4. 9-Methoxy-4-(4-nitrophenyl)-7*H*-furo[3,2-*g*]chromen-7-one (**11b**) ... 37
3.2.5. 4-(9-Methoxy-7-oxo-7*H*-furo[3,2-*g]*chromen-4-yl)benzaldehyde (**11c**) ... 38
3.2.6. 9-Methoxy-4-(pyridin-4-yl)-7*H*-furo[3,2-*g*]chromen-7-one (**11d**) ... 39
3.2.7. 4-(4-(Dimethylamino)phenyl)-9-methoxy-7*H*-furo[3,2-*g*]chromen-7-one (**11e**) ... 40
3.2.8 4-(4-((Dimethylamino)methyl)phenyl)-9-methoxy-7*H*-furo[3,2-*g*]chromen-7-one (**11f**) ... 41
3.2.9. 4-(9-Methoxy-7-oxo-7H-furo[3,2-*g*]chromen-4-yl)benzoic acid (**11g**) ... 42
3.2.10. 4-((9-Methoxy-7-oxo-7*H*-furo[3,2-*g*]chromen-4-yl)ethynyl)benzonitrile (**13a**) ... 43
3.2.11. 9-Methoxy-4-((4-nitrophenyl)ethynyl)-7*H*-furo[3,2-*g*]chromen-7-one (**13b**) ... 44
3.2.12. 4-((9-Methoxy-7-oxo-7*H*-furo[3,2-*g*]chromen-4-yl)ethynyl)benzaldehyde (**13c**) ... 45
3.2.13. 9-Methoxy-4-(pyridin-4-ylethynyl)-7*H*-furo[3,2-*g*]chromen-7-one (**13d**) ... 46
3.2.14. 4-((4-(Dimethylamino)phenyl)ethynyl)-9-methoxy-7*H*-furo[3,2-*g*]chromen-7-one (**13e**) ... 47
3.2.15. (*E*)-4-(2-(9-methoxy-7-oxo-7*H*-furo[3,2-*g*]chromen-4-yl)vinyl)benzonitrile (**15a**) ... 49
3.2.16. (*E*)-9-methoxy-4-(4-nitrostyryl)-7*H*-furo[3,2-*g*]chromen-7-one (**15b**) ... 50
3.2.17. (*E*)-4-(2-(9-methoxy-7-oxo-7*H*-furo[3,2-*g*]chromen-4-yl)vinyl)benzaldehyde (**15c**) ... 51
3.2.18. (*E*)-9-methoxy-4-(2-(pyridin-4-yl)vinyl)-7*H*-furo[3,2-*g*]chromen-7-one (**15d**) ... 52
3.2.19. (*E*)-4-(4-(Dimethylamino)styryl)-9-methoxy-7*H*-furo[3,2-*g*]chromen-7-one (**15e**) ... 53
4. Absorption and emission spectra ... 54
4.1. Absorption spectra of compound **8** ... 54
4.2. Absorption spectra of compound **9** ... 54
4.3. Absorption spectra of compound **11a** ... 55
4.4. Absorption spectra of compound **11b** ... 55
4.5. Absorption spectra of compound **11c** ... 56
4.6. Absorption spectra of compound **11d** ... 56
4.6. Absorption and emission spectra of compound **11e** ... 57
4.7. Absorption spectra of compound **11f** ... 57
4.8. Absorption spectra of compound **11g** ... 58
4.9. Absorption and emission spectra of compound **13a** ... 58
4.10. Absorption and emission spectra of compound **13b** ... 59
4.11. Absorption and emission spectra of compound **13c** ... 59
4.12. Absorption and emission spectra of compound **13d** ... 60
4.13. Absorption and emission spectra of compound **13e** ... 60
4.14. Absorption and emission spectra of compound **15a** ... 61
4.15. Absorption and emission spectra of compound **15b** ... 61
4.16. Absorption and emission spectra of compound **15c** ... 62

4.17. Absorption and emission spectra of compound **15d** 62
4.18. Absorption and emission spectra of compound **15e** 63
4.19. Emission spectra of PMMA-films of compounds **11e**, **13e**, **15e** 63
and solid state emission of compound **13e** 63
5. Solvatochromicity 64
5.1. Solvatochromicity of compound **11e** 64
5.2. Solvatochromicity of compound **13e** 65
5.3. Solvatochromicity of compound **15e** 67
6. Acidochromicity 69
6.1. Absorption spectra of non-protonated and protonated compounds 69
6.1.1. Absorption spectra of compound **11e** 69
6.1.2. Absorption spectra of compound **13e** 69
6.1.3. Absorption spectra of compound **15e** 70
6.2. Determination of pK_a value 70
6.2.1. Titration Experiment (Absorption) of compound **11e** 71
6.2.2. Titration Experiment (Absorption) of compound **13e** 72
6.2.3. Titration Experiment (Absorption) of compound **15e** 73
6.2.4. Titration Experiment (Fluorescence Quenching) of compound **11e** 74
6.2.5. Titration Experiment (Fluorescence Quenching) of compound **13e** 76
6.2.6. Titration Experiment (Fluorescence Quenching) of compound **15e** 77
7. X-ray 79
7.1. X-ray structural data of compound **11a** 79
7.2. X-ray structural data of compound **13e** 81
8. Computed xyz-coordinates and computed UV/Vis spectra of TD-DFT calculated structures 83
8.1. XYZ-coordinates of the S0 state of compound **8** (PBE1PBE/6-311G(d,p)) and TD-DFT calculation (PBE1PBE/6-21G, SCRF(IEFPCM, DCM)) 83
8.2. XYZ-coordinates of the S0 state of compound **9** (PBE1PBE/6-311G(d,p)) and TD-DFT calculation (PBE1PBE/6-21G, SCRF(IEFPCM, DCM) 87
8.3. XYZ-coordinates of the S0 state of compound **11a** (PBE1PBE/6-311G(d,p)) and TD-DFT calculation (PBE1PBE/6-21G, SCRF(IEFPCM, DCM) 92
8.4. XYZ-coordinates of the S0 state of compound **11b** (PBE1PBE/6-311G(d,p)) and TD-DFT calculation (PBE1PBE/6-21G, SCRF(IEFPCM, DCM) 99
8.5. XYZ-coordinates of the S0 state of compound **11c** (PBE1PBE/6-311G(d,p)) and TD-DFT calculation (PBE1PBE/6-21G, SCRF(IEFPCM, DCM) 105
8.6. XYZ-coordinates of the S0 state of compound **11d** (PBE1PBE/6-311G(d,p)) and TD-DFT calculation (PBE1PBE/6-21G, SCRF(IEFPCM, DCM) 110
8.7. XYZ-coordinates of the S0 state of compound **11e** (PBE1PBE/6-311G(d,p)) and TD-DFT calculation (PBE1PBE/6-21G, SCRF(IEFPCM, DCM) 116
8.8. XYZ-coordinates of the S0 state of compound **11f** (PBE1PBE/6-311G(d,p)) and TD-DFT calculation (PBE1PBE/6-21G, SCRF(IEFPCM, DCM) 121
8.9. XYZ-coordinates of the S0 state of compound **11g** (PBE1PBE/6-311G(d,p)) and TD-DFT calculation (PBE1PBE/6-21G, SCRF(IEFPCM, DCM) 128
8.10. XYZ-coordinates of the S0 state of compound **13a** (PBE1PBE/6-311G(d,p)) and TD-DFT calculation (PBE1PBE/6-21G, SCRF(IEFPCM, DCM) 134

8.11. XYZ-coordinates of the S0 state of compound **13b** (PBE1PBE/6-311G(d,p)) and TD-DFT calculation (PBE1PBE/6-21G, SCRF(IEFPCM, DCM).. 139

8.12. XYZ-coordinates of the S0 state of compound **13c** (PBE1PBE/6-311G(d,p)) and TD-DFT calculation (PBE1PBE/6-21G, SCRF(IEFPCM, DCM).. 144

8.13. XYZ-coordinates of the S0 state of compound **13d** (PBE1PBE/6-311G(d,p)) and TD-DFT calculation (PBE1PBE/6-21G, SCRF(IEFPCM, DCM).. 149

8.14. XYZ-coordinates of the S0 state of compound **13e** (PBE1PBE/6-311G(d,p)) and TD-DFT calculation (PBE1PBE/6-21G, SCRF(IEFPCM, DCM).. 154

8.15. XYZ-coordinates of the S0 state of compound **15a** (PBE1PBE/6-311G(d,p)) and TD-DFT calculation (PBE1PBE/6-21G, SCRF(IEFPCM, DCM).. 161

8.16. XYZ-coordinates of the S0 state of compound **15b** (PBE1PBE/6-311G(d,p)) and TD-DFT calculation (PBE1PBE/6-21G, SCRF(IEFPCM, DCM).. 166

8.17. XYZ-coordinates of the S0 state of compound **15c** (PBE1PBE/6-311G(d,p)) and TD-DFT calculation (PBE1PBE/6-21G, SCRF(IEFPCM, DCM).. 172

8.18. XYZ-coordinates of the S0 state of compound **15d** (PBE1PBE/6-311G(d,p)) and TD-DFT calculation (PBE1PBE/6-21G, SCRF(IEFPCM, DCM).. 178

8.19. XYZ-coordinates of the S0 state of compound **15e** (PBE1PBE/6-311G(d,p)) and TD-DFT calculation (PBE1PBE/6-21G, SCRF(IEFPCM, DCM).. 184

8.20. Kohn-Sham FMOs of the DFT calculated structures **11a**, **11e**, **13a**, **13e**, **15a**, **15e**, and **5-Ph-8-MOP**.. 189

9. Literature.. 190

1. Synthesis of starting materials

General considerations

All reactions were carried out under exclusion of air by Schlenk technique. Dry solvents 1,4-dioxane, THF and CH_2Cl_2 were taken from the drying system (MB-SPS 800 from M.Braun). All other chemicals that were not self-made were purchased and continued to be used without purification. Reaction controls were carried out by TLC, using silica-gel-coated finished aluminum films from Macherey and Nagel (60 UV_{254}). The detection was carried out using UV light of the wavelengths 254 and 365 nm. The purification of the crude products was carried out by column chromatography. In this case, silica gel 60 (particle size 0.04-0.063 mm) from Macherey and Nagel and the flash technique under a pressure of 1.5 bar or the chromatograph Biotage SP1 were used. For this purpose, the crude products were adsorbed on Celite® 545 from Carl Roth GmbH. The assignments of C_{quat}, CH_3, CH_2 and CH were determined using DEPT-135 spectra. The deuterated solvents used were acetone-d_6, DMF-d_7, $CDCl_3$ and CD_2Cl_2. The intensities of the IR absorption bands are abbreviated to vs (very strong), s (strong), m (medium) and w (weak).

1.1. 2-Methoxybenzene-1,3-diol[1] (2)

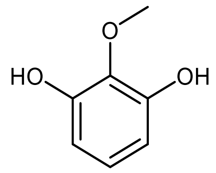

Pyrogallol (**1**, 2.52 g, 20.0 mmol) was solved in acetone (60 mL). The reaction mixture was stirred at 75 °C for 10 min. Subsequently methyl iodide (1.87 mL, 30.0 mmol) was added dropwise. After complete addition the reaction mixture was stirred at 75 °C for 19 h. After cooling, the reaction mixture was filtered off and the solvent was removed under reduced pressure. The brown residue was dissolved in diethyl ether and filtered again. The filtrate was absorbed on Celite® and purified by column chromatography (*n*-hexane/ethyl acetate 9:1). The viscous oil was sublimed at 80 °C in vacuo and gave colorless crystals of 2-methoxybenzene-1,3-diol. Yield: 1.29 g (9.21 mmol, 46%) colorless crystals.

R_f = 0.65 (*n*-hexane/ethyl acetate, 1:1). Mp 85 °C. ^{1}H NMR: (300 MHz, $CDCl_3$) δ = 6.87 (t, J = 8.5, 7.9 Hz, 1 H), 6.52 (d, J = 8.2 Hz, 2 H), 5.53 (s, 2 H), 3.88 (s, 3 H). ^{13}C NMR: (75 MHz, $CDCl_3$) δ = 149.1 (C_{quat}), 134.8 (C_{quat}), 125.0 (CH), 108.4 (CH), 61.3 (CH_3). MS (EI) (m/z (%)): 140 ($[C_7H_8O_3]^+$, 100), 125 ($[C_6H_5O_3]^+$, 92), 97 ($[C_5H_5O_2]^+$, 46), 51 ($[C_4H_4]^+$, 15). Anal. Calcd. for $C_7H_8O_3$ [140.14]: C 59.99, H 5.75. Found: C 59.98, H 6.04. IR: $\tilde{\nu}$ [cm^{-1}] = 3325 (m), 3001 (w), 2943 (w), 2835 (w), 1589 (w), 1574 (w), 1493 (s), 1467 (w), 1360 (m),

1260 (w), 1167 (s), 1153 (s), 1059 (m), 1018 (m), 988 (s), 947 (w), 845 (w), 812 (w), 766 (m), 731 (vs), 708 (m), 689 (m).

1.2. 7-Hydroxy-8-methoxycoumarin (3)

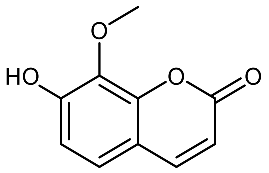

2-Methoxybenzene-1,3-diol (**2**, 2.80 g, 20.0 mmol) was dissolved in DCM (2 mL). Zinc chloride (2.72 g, 20.0 mmol) was added and heated to 80 °C. Over a period of 12 h, ethyl propiolate (3.05 mL, 30.0 mmol) was added dropwise by syringe pump and stirred for further 12 h. After cooling, a 5% HCl solution (25 mL) was slowly added dropwise. The solution was extracted several times with ethyl acetate (5 x 20 mL). The ethyl acetate layers were combined and dried with anhydrous sodium sulfate. The product was then adsorbed on Celite® and purified by column chromatography (petroleum ether/ethyl acetate, 2:1). The yellow solid was recrystallized in ethanol. Yield: 1.22 g (6.35 mmol, 32%) yellow solid.

R_f = 0.60 (*n*-hexane/ethyl acetate, 1:1). Mp 157 °C. ^{1}H NMR: (600 MHz, $CDCl_3$) δ = 7.63 (d, J = 9.5 Hz, 1 H), 7.11 (d, J = 8.5 Hz, 1 H), 6.90 (d, J = 8.5 Hz, 1 H), 6.43 (s, 1 H), 6.24 (d, J = 9.5 Hz, 1 H), 4.11 (s, 3 H). ^{13}C NMR: (150 MHz, $CDCl_3$) δ = 160.3 (C_{quat}), 152.0 (C_{quat}), 147.1 (C_{quat}), 144.2 (CH), 133.6 (C_{quat}), 123.2 (CH), 113.1 (C_{quat}), 112.5 (CH), 112.1 (CH), 61.7 (CH_3). MS (EI) (m/z (%)): 192 ($[C_{10}H_8O_4]^+$, 100), 177 ($[C_9H_5O_4]^+$, 23), 164 ($[C_9H_8O_3]^+$, 23), 149 ($[C_9H_9O_2]^+$, 27). Anal. Calcd. for $C_{10}H_8O_4$ [192.17]: C 62.50, H 4.20. Found: C 62.50, H 4.50. IR: $\tilde{\nu}$ [cm^{-1}] = 3321 (m), 3080 (w), 2941 (w), 1692 (s), 1602 (s), 1558 (m), 1504 (m), 1456 (m), 1433 (s), 1412 (w), 1342 (m), 1128 (w), 1244 (m), 1206 (m), 1190 (m), 1165 (s), 1148 (s), 1124 (s), 1067 (s), 1034 (s), 1005 (w), 966 (s), 841 (s), 827 (m), 775 (m), 718 (m), 623 (s), 664 (m), 631 (m).

1.3. 7-(2,2-Diethoxyethoxy)-8-methoxy-2*H*-chromen-2-one (4)

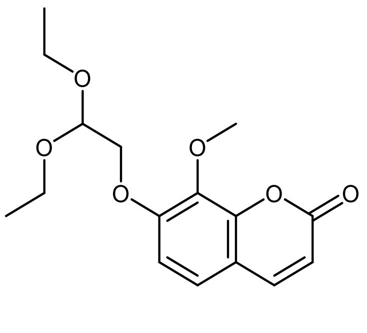

7-Hydroxy-8-methoxycoumarin (**3**, 192 mg, 1.00 mmol) and potassium carbonate (172 mg, 1.25 mmol) were placed in a Schlenk tube. Then DMF (1 mL) and bromoacetaldehyde diethyl acetal (0.191 mL, 1.25 mmol) were added and degassed with nitrogen for 5 min. The

reaction mixture was stirred at 110 °C for 20 h. After the reaction had cooled, the reaction mixture was admixed with ethyl acetate (25 mL) and washed with saturated NaCl solution (1 × 10 mL) and H_2O (2 × 10 mL). The organic phase was dried with anhydrous sodium sulfate and adsorbed on Celite® and purified by column chromatography (petroleum ether/ethyl acetate, 1:1). Yield: 276 mg (0.896 mmol, 90%) beige solid.

R_f = 0.67 (*n*-hexane/ethyl acetate, 1:1). Mp 92 °C. ^{1}H NMR: (300 MHz, $CDCl_3$) δ = 7.60 (d, *J* = 9.5 Hz, 1 H), 7.13 (d, *J* = 8.7 Hz, 1 H), 6.87 (d, *J* = 8.7 Hz, 1 H), 6.24 (d, *J* = 9.5 Hz, 1 H), 4.87 (t, *J* = 5.2 Hz, 1 H), 4.09 (d, *J* = 5.3 Hz, 2 H), 3.96 (d, *J* = 0.5 Hz, 3 H), 3.77 (m, 2 H), 3.64 (m, 2 H), 1.22 (t, *J* = 7.0 Hz, 6 H). ^{13}C NMR: (75 MHz, $CDCl_3$) δ = 160.54 (C_{quat}), 154.74 (C_{quat}), 148.15 (C_{quat}), 143.58 (CH), 136.61 (C_{quat}), 122.69 (CH), 114.03 (C_{quat}), 113.67 (CH), 110.19 (CH), 100.52 (CH), 69.95 (CH_2), 63.16 (CH_2), 61.34 (CH_3), 15.33 (CH_3). ESI-MS: calcd. for $(C_{16}H_{20}O_6+H)^+$ m/z = 309.13 (100%); Found: 309.4 (100%). Anal. Calcd. for $C_{13}H_{10}O_4$ [230.06]: C 62.33, H 6.54. Found: C 62.08, H 6.84. IR: $\tilde{\nu}$ [cm^{-1}] = 2934 (w), 1709 (vs), 1605 (s), 1562 (w), 1501 (m), 1452 (m), 1404 (w), 1375 (w), 1350 (w), 1298 (s), 1263 (m), 1238 (w), 1209 (m), 1171 (m), 1134 (s), 1101 (s), 1063 (vs), 997 (w), 962 (m), 941 (w), 895 (w), 839 (s), 777 (m), 729 (m), 696 (m), 631 (w), 619 (m).

1.4. 2-((8-Methoxy-2-oxo-2*H*-chromen-7-yl)oxy)acetaldehyde (5)

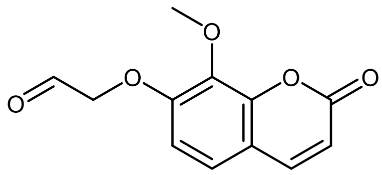

7-(2,2-Diethoxyethoxy)-8-methoxy-2*H*-chromen-2-one (**4**, 308 mg, 1.00 mmol) was dissolved in acetone (0.75 mL) in a Schlenk tube and HCl (1 M, 1.50 mL) was added. The reaction mixture was heated to 60 °C for 2 h and then cooled at -8 °C overnight. The colorless precipitated solid was filtered in vacuo and dried under vacuo for 8 h. Yield: 215 mg (0.919 mmol, 92%) colorless solid.

R_f = 0.16 (*n*-hexane/ethyl acetate, 1:1). Mp 89 °C. ^{1}H NMR: (300 MHz, $CDCl_3$) δ = 9.84 (d, *J* = 0.4 Hz, 1 H), 7.89 (dd, *J* = 9.6, 0.3 Hz, 1 H), 7.35 (d, *J* = 8.7 Hz, 1 H), 7.00 (d, *J* = 8.7 Hz, 1 H), 6.26 (dd, *J* = 9.5, 0.3 Hz, 1 H), 5.01 (s, 2 H), 3.97 (s, 3 H). ^{13}C NMR: (75 MHz, $CDCl_3$) δ = 198.20 (CH), 160.42 (C_{quat}), 154.95 (C_{quat}), 149.17 (C_{quat}), 144.72 (CH), 137.20 (C_{quat}), 123.97 (CH), 115.37 (C_{quat}), 114.44 (CH), 111.22 (CH), 74.46 (CH_2), 61.42 (CH_3). MS (EI) (m/z (%)): 234 ($[C_{12}H_{10}O_5]^+$, 100), 206 ($[C_{11}H_9O_4]^+$, 58), 190 ($[C_{10}H_7O_4]^+$, 56). HR-MS (ESI): calcd. for $(C_{12}H_{10}O_5+H)^+$ m/z = 235.2145. Found: 235.2161 (100%). IR: $\tilde{\nu}$ [cm^{-1}] = 3347 (m), 1701 (s), 1603 (s), 1558 (m), 1508 (m), 1452 (w), 1427 (w), 1404 (w), 1300 (s), 1263 (s), 1213 (w), 1178 (m), 1138 (s), 1088 (s), 1045 (s), 970 (m), 908 (m), 849 (s), 775 (w), 694 (s).

1.5. 8-Methoxypsoralen[2] (6)

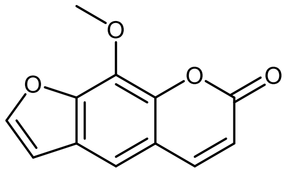

2-((8-Methoxy-2-oxo-2*H*-chromen-7-yl)oxy)acetaldehyde (**5**, 608 mg, 2.60 mmol) was dissolved in 1,4-dioxane (3 mL) and added dropwise by syringe pump over a period of 12 h to a NaOH solution (1 M, 50 mL) at 100 °C. After cooling, phosphoric acid (85%, 3.50 mL) was slowly added dropwise. The aqueous phase was washed with ethyl acetate (3 x 100 mL) and the organic phases were combined and dried with anhydrous sodium sulfate. The mixture was then adsorbed on Celite® and purified by column chromatography (petroleum ether/ethyl acetate, 1:1). After recrystallization a colorless solid could be obtained. Yield: 212 mg (0.981 mmol, 38%) colorless solid.

R_f = 0.57 (*n*-hexane/ethyl acetate, 1:1). Mp 145 °C. ^{1}H NMR: (300 MHz, $CDCl_3$) δ = 7.75 (d, *J* = 9.6 Hz, 1 H), 7.68 (d, *J* = 2.2 Hz, 1 H), 7.34 (s, 1 H), 6.81 (d, *J* = 2.2 Hz, 1 H), 6.35 (d, *J* = 9.6 Hz, 1 H), 4.28 (s, 3 H). ^{13}C NMR: (75 MHz, $CDCl_3$) δ = 159.44 (C_{quat}), 146.64 (C_{quat}), 145.62 (CH), 143.33 (CH), 141.95 (C_{quat}), 131.75 (C_{quat}), 125.11 (C_{quat}), 115.45 (C_{quat}), 113.69 (CH), 111.90 (CH), 105.71 (CH), 60.30 (CH_3). MS (EI) (m/z (%)): 216 ($[C_{12}H_8O_4]^+$, 100), 201 ($[C_{11}H_5O_4]^+$, 28). Anal. Calcd. for $C_{12}H_8O_4$ [216.04]: C 66.67, H 3.73. Found: C 66.65, H 3.71. IR: $\tilde{\nu}$[cm^{-1}] = 3109 (w), 2953 (w), 1699 (s), 1620 (w), 1582 (s), 1504 (w), 1456 (m), 1431 (m), 1398 (s), 1335 (m), 1296 (m), 1267 (w), 1219 (m), 1175 (w), 1146 (m), 1130 (m), 1098 (s), 1086 (s), 1059 (w), 1026 (m), 997 (m), 949 (s), 895 (w), 872 (s), 856 (s), 837 (m), 812 (s), 789 (m), 746 (s), 725 (w), 704 (w), 671 (m).

1.6. 5-Bromo-8-methoxypsoralen (7)

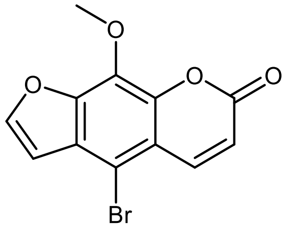

8-Methoxypsoralen (**6**, 973 mg, 4.50 mmol) was dissolved in DMSO (959 µL, 13.5 mmol) and ethyl acetate (18 mL) and heated to 60 °C. HBr (48%, 2.28 g, 13.5 mmol) was added and stirred at the same temperature for 16 h. After cooling the reaction mixture, the ethyl acetate layer was dried with anhydrous sodium sulfate and adsorbed on Celite® and purified by column chromatography (petroleum ether/ethyl acetate, 2:1). After recrystallization with ethyl

acetate thus obtaining the product in form of colorless crystals. Yield: 1.23 mg (4.17 mmol, 93%) colorless crystals.

R_f = 0.55 (*n*-hexane/ethyl acetate, 2:1). Mp 183 °C. ^{1}H NMR: (600 MHz, $CDCl_3$) δ = 8.12 (d, *J* = 9.8 Hz, 1 H), 7.72 (d, *J* = 2.2 Hz, 1 H), 6.87 (d, *J* = 2.2 Hz, 1 H), 6.44 (d, *J* = 9.8 Hz, 1 H), 4.28 (s, 3 H). ^{13}C NMR: (150 MHz, $CDCl_3$) δ = 159.75 (C_{quat}), 146.91 (CH), 146.66 (C_{quat}), 143.74 (C_{quat}), 142.61 (CH), 132.44 (C_{quat}), 127.98 (C_{quat}), 115.85 (CH), 115.68 (C_{quat}), 107.46 (CH), 105.55 (C_{quat}), 61.44 (CH_3). MS (EI) (m/z (%)): 294 ($[C_{12}H_7{}^{79}BrO_4]^+$, 100), 296 ($[C_{12}H_7{}^{81}BrO_4]^+$, 90), 215 ($[C_{12}H_7O_4]^+$, 61). Anal. Calcd. for $C_{12}H_7BrO_4$ [293.95]: C 48.84, H 2.39. Found: C 48.77, H 2.22. IR: $\tilde{\nu}$ [cm^{-1}] =3134 (w), 2947 (w), 2843 (w), 1721 (s), 1626 (w), 1589 (s), 1456 (m), 1415 (m), 1375 (m), 1329 (m), 1294 (w), 1261 (w), 1206 (w), 1148 (s), 1124 (w), 1105 (m), 1024 (m), 995 (m), 959 (m), 887 (m), 826 (m), 785 (w), 773 (w), 750 (s), 725 (w), 683 (w), 652 (w).

2. Synthesis of 5-substituted 8-methoxypsoralens

2.1. 5-Cyano-8-methoxypsoralen (8)

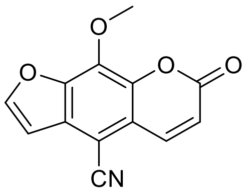

5-Bromo-8-methoxypsoralen (**7**, 147 mg, 0.500 mmol), $Zn(OAc)_2$ (3.61 mg, 19.7 µmol), zinc powder (1.32 mg, 20.2 µmol), $Pd_2(dba)_3$ (90.0 µg, 1.00 µmol), 1,1'-bis(diphenylphosphino)ferrocene (1.40 mg,2.40 µmol) were placed in a seked screw-cap Schlenk tube and dissolved in DMF (0.5 mL) and water (5 µL). Thereafter, the reaction mixture was degassed with nitrogen for 5 min and stirred at 100 °C for 20 minutes. The cooled reaction mixture was then poured into a $NH_4Cl/NH_3/H_2O$ solution (4:1:5.50 mL) and the precipitated beige solid was filtered off and recrystallized in acetone. Yield: 93.2 mg (0.386 mmol, 77%) beige solid.

R_f = 0.43 (*n*-hexane/ethyl acetate, 2:1). Mp 248 °C. ^{1}H NMR: (300 MHz, $CDCl_3$) δ = 8.08 (d, *J* = 9.8 Hz, 1 H), 7.84 (d, *J* = 2.2 Hz, 1 H), 7.05 (d, *J* = 2.3 Hz, 1 H), 6.60 (d, *J* = 9.8 Hz, 1 H), 4.46 (s, 3 H). ^{13}C NMR: (75 MHz, $CDCl_3$) δ = 158.84 (C_{quat}), 148.90 (CH), 145.20 (C_{quat}), 142.25 (C_{quat}), 140.29 (CH), 136.96 (C_{quat}), 130.39 (C_{quat}), 118.79 (C_{quat}), 118.03 (CH), 115.00 (C_{quat}), 105.99 (CH), 92.55 (C_{quat}), 61.35 (CH_3). MS (EI) (m/z (%)): 241 ($[C_{13}H_7NO_4]^+$, 100)). Anal. Calcd. for $C_{13}H_7NO_4$ [241.20]: C 64.54, H 2.95, N 5.60. Found: C 64.74, H 2.93, N 5.81. IR: $\tilde{\nu}$ [cm^{-1}] = 3115 (w), 2222 (m), 1724 (vs), 1626 (w), 1580 (vs), 1539 (w), 1473 (m), 1427 (s), 1383 (s), 1348 (m), 1305 (s), 1309 (s), 1217 (s), 1202 (m), 1169 (s), 1132 (vs), 1095 (s),

1047 (s), 1024 (s), 945 (w), 889 (w), 881 (w), 860 (m), 837 (s), 810 (m), 795 (s), 761 (s), 725 (w), 673 (m).

2.2. 5-Nitro-8-methoxypsoralen[3] (9)

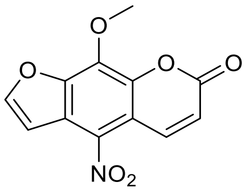

8-Methoxypsoralen (**7**, 135 mg, 0.625 mmol) was dissolved in glacial acetic acid at room temperature (1.35 mL) and HNO_3 (65%, 1.08 mL) were added dropwise. The yellow solution was stirred for 1 h at room temperature and then filtered off. The yellow residue was washed with water and dried. Yield: 136 mg (0.521 mmol, 84%) yellow solid.

R_f = 0.81 (*n*-hexane/ethyl acetate, 1:1). Mp 233 °C. ^{1}H NMR: (300 MHz, $CDCl_3$) δ = 8.79 (d, *J* = 10.2 Hz, 1 H), 7.87 (d, *J* = 2.2 Hz, 1 H), 7.43 (d, *J* = 2.2 Hz, 1 H), 6.64 (d, *J* = 10.2 Hz, 1 H), 4.50 (s, 3 H). ^{13}C NMR: (75 MHz, $CDCl_3$) δ = 158.21 (C_{quat}), 149.45 (CH), 144.85 (C_{quat}), 142.07 (C_{quat}), 139.24 (CH), 137.61 (C_{quat}), 129.70 (C_{quat}), 124.55 (C_{quat}), 118.62 (CH), 112.25 (C_{quat}), 107.42 (CH), 61.50 (CH_3). MS (EI) (m/z (%)): 261 ($[C_{12}H_7NO_6]^+$, 100). Anal. Calcd. for $C_{12}H_7NO_6$ [261.03]: C 55.18, H 2.70, N 5.36. Found: C 55.10, H 2.47, N 5.19. IR: $\tilde{\nu}$ [cm^{-1}] = 3667 (w), 3132 (w), 2968 (m), 1736 (vs), 1618 (w), 1570 (s), 1539 (w), 1497 (s), 1445 (w), 1409 (w), 1348 (w), 1315 (s), 1282 (s), 1265 (s), 1217 (w), 1188 (w), 1161 (s), 1138 (s), 1121 (s), 1096 (m), 1051 (m), 1043 (m), 1020 (s), 935 (m), 889 (w), 874 (m), 822 (w), 787 (s), 764 (m), 740 (w), 671 (w), 656 (w), 627 (m).

2.1. General procedure: Suzuki coupling

Procedure A:

5-Bromo-8-methoxypsoralen (**7**) was placed in dry THF under nitrogen atmosphere in a screw-cap Schlenk tube. The boronic acid, tetrakis(triphenylphosphine)palladium(0), potassium carbonate and methanol were added and the solution was then degassed with nitrogen for 5 min. The reaction mixture was stirred at 70 °C for 48 h. After cooling, water was added (10 mL) and the aqueous phase extracted with ethyl acetate (3 x 30 mL). The organic layers were combined and dried with anhydrous sodium sulfate. The mixture was adsorbed on Celite® and purified by column chromatography (petroleum ether/ethyl acetate).

Table 1. Experimental details of the Suzuki coupling of 5-substituted 8-methoxypsoralens **11**.

entry	5-Br-8-MOP (**7**)	boronic acid **10**	$Pd(PPh_3)_4$	K_2CO_3	THF/MeOH	product **11** (yield)
1	222 mg (0.752 mmol)	**10a**, 138 mg (0.939 mmol)	78.1 mg (67.7 µmol)	311 mg (2.25 mmol)	6 mL/2 mL	**11a**,140 mg (0.441 mmol) 59%
2	194 mg (0.660 mmol)	**10b**, 136 mg (0.815 mmol)	69.0 mg (59.7 µmol)	273 mg (1.98 mmol)	6 mL/2 mL	**11b**, 136 mg (0.403 mmol) 61%
3	295 mg (1.00 mmol)	**10c**, 187 mg (1.25 mmol)	104 mg (90.0 µmol)	415 mg (3.00 mmol)	6 mL/2 mL	**11c**, 138 mg (0.430 mmol) 43%
4	193 mg (0.654 mmol)	**10d**, 112 mg (0.818 mmol)	205 mg (0.177 mmol)	271 mg (1.96 mmol)	6 mL/2 mL	**11d**, 96.1 mg (0.328 mmol) 50%
5	295 mg (1.00 mmol)	**10e**, 190 mg (1.25 mmol)	104 mg (90.0 µmol)	414 mg (3.00 mmol)	8 mL/2 mL	**11e**, 199 mg (0.593 mmol) 59%
6	590 mg (2.00 mmol)	**10f**, 447 mg (2.45 mmol)	208 mg (0.180 mmol)	828 mg (6.05 mmol)	8 mL/4 mL	**11f**, 218 mg (0.624 mmol) 31%

Procedure B:

5-Bromo-8-methoxypsoralen (**7**) was placed in dry THF (4 mL/mmol) under nitrogen atmosphere in a screw-cap Schlenk tube. The boronic acid, bis(dibenzylideneacetone)palladium(0), tri-*tert*-butylphosphonium tetrafluoroborate and potassium fluoride were added and the solution was then degassed with nitrogen for 5 min. The reaction mixture was stirred at room temperature for 21 h. After cooling, water was added (10 mL) and the aqueous phase extracted with ethyl acetate (3 x 30 mL). The organic layers were combined and dried with anhydrous sodium sulfate. The mixture was adsorbed on Celite® and purified by column chromatography (petroleum ether/ethyl acetate).

Table 2. Experimental details of the Suzuki coupling of 5-substituted 8-methoxypsoralen **11b**.

entry	5-Br-8-MOP (**7**)	boronic acid **10**	$Pd(dba)_2$	KF	$[(t\text{-}Bu)_3PH]BF_4$	product **11** (yield)
1	235 mg, (0.796 mmol)	**10b**, 145 mg, (0.876 mmol)	10.9 mg, (11.9 µmol)	138 mg, (2.39 mmol)	6.92 mg, (23.9 µmol)	**11b**, 268 mg, (79.5 µmol) 99%

Procedure C:

5-Bromo-8-methoxypsoralen (**7**) was placed in dry THF (4 mL/mmol) under nitrogen atmosphere in a screw-cap Schlenk tube. The boronic acid, tris(dibenzylideneacetone) dipalladium(0), SPhos and potassium fluoride were added and the solution was then degassed with nitrogen for 5 min. The reaction mixture was stirred at room temperature for 24 h. After cooling, water was added (10 mL) and the aqueous phase extracted with ethyl acetate
(3 x 30 mL). The organic layers were combined and dried with anhydrous sodium sulfate. The mixture was adsorbed on Celite® and purified by column chromatography (petroleum ether/ethyl acetate).

Table 3. Experimental details of the Suzuki coupling of 5-substituted 8-methoxypsoralens **11g**.

entry	5-Br-8-MOP (**7**)	boronic acid **10**	Pd_2dba_3	KF	SPhos	product **11** (yield)
1	443 mg (1.50 mmol)	**10g**, 274 mg (1.65 mmol)	173 mg (0.301 mmol)	260 mg (4.49 mmol)	164 mg (40.0 µmol)	**11g**, 272 mg (0.810 mmol) 54%

2.1.1. 4-(9-Methoxy-7-oxo-7*H*-furo[3,2-*g*]chromen-4-yl)benzonitrile (11a)

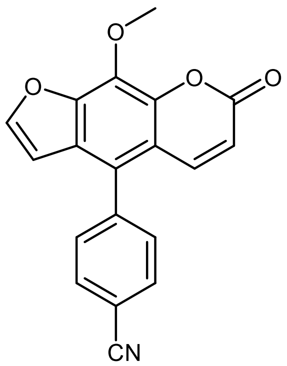

The column chromatographic purification was carried out with petroleum ether/ethyl acetate (4:1). The yellow solid was recrystallized in ethanol. Yield: 140 mg (0.441 mmol, 59%) yellowish crystals.

R_f = 0.44 (*n*-hexane/ethyl acetate, 2:1). Dec. 250 °C. ^{1}H NMR: (600 MHz, $CDCl_3$) δ = 7.88 – 7.81 (m, 2 H), 7.71 (d, J = 2.3 Hz, 2 H), 7.66 (d, J = 9.9 Hz, 1 H), 7.57 – 7.49 (m, 1 H), 6.60 (d, J = 2.3 Hz, 1 H), 6.35 (d, J = 9.9 Hz, 1 H), 4.35 (s, 3 H). ^{13}C NMR: (150 MHz,

CDCl$_3$) δ = 159.80 (C_{quat}), 147.21 (CH), 146.82 (C_{quat}), 143.58 (C_{quat}), 141.37 (CH), 140.48 (C_{quat}), 132.96 (C_{quat}), 132.60 (CH), 131.16 (CH), 125.79 (C_{quat}), 123.96 (C_{quat}), 118.33 (C_{quat}), 115.25 (CH), 114.12 (C_{quat}), 112.31 (C_{quat}), 105.91 (CH), 61.44 (CH_3). MS (EI) (m/z (%)): 317 ($[C_{19}H_{11}NO_4]^+$, 100), 302 ($[C_{18}H_8NO_{44}]^+$, 33). Anal. Calcd. for $C_{19}H_{11}NO_4$ [317.30]: C 71.92, H 3.49, N 4.41. Found: C 71.67, H 3.31, N 4.36. IR: $\tilde{\nu}$ [cm^{-1}] = 3148 (w), 2230 (m), 1713 (vs), 1472 (m), 1422 (m), 1368 (m), 1315 (m), 1273 (w), 1207 (m), 1175 (w), 1157 (s), 1140 (m), 1096 (s), 1088 (s), 1040 (s), 1013 (s), 955 (w), 901 (w), 883 (m), 873 (m), 835 (s), 822 (s), 754 (s), 723 (w), 684 (w), 646 (w), 629 (s), 621 (w).

2.1.2. 9-Methoxy-4-(4-nitrophenyl)-7*H*-furo[3,2-*g*]chromen-7-one (11b)

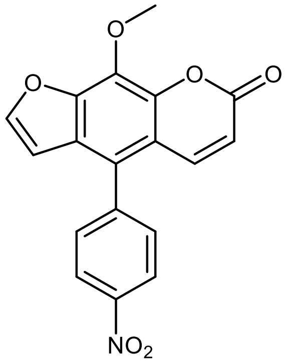

Procedure A:

The column chromatographic purification was carried out with petroleum ether/ethyl acetate (4:1 to 1:9). The yellow solid was recrystallized in ethanol. Yield: 136 mg (0.403 mmol, 61%) yellowish solid.

Procedure B:

The column chromatographic purification was carried out with petroleum ether/ethyl acetate (3:1 to 1:9). The yellow solid was recrystallized in ethanol. Yield: 268 mg (0.795 mmol, 99%) yellowish solid

R_f = 0.24 (*n*-hexane/ethyl acetate, 3:1). Mp 219 °C. ^{1}H NMR: (600 MHz, CDCl$_3$) δ = 8.47 – 8.36 (m, 2 H), 7.79 – 7.52 (m, 4 H), 6.61 (d, *J* = 2.3 Hz, 1 H), 6.36 (d, *J* = 9.9 Hz, 1 H), 4.36 (s, 3 H). ^{13}C NMR: (150 MHz, CDCl$_3$) δ = 159.76 (C_{quat}), 147.69 (C_{quat}), 147.31 (CH), 146.78 (C_{quat}), 143.57 (C_{quat}), 142.41 (C_{quat}), 141.28 (CH), 133.09 (C_{quat}), 131.35 (CH), 125.85 (C_{quat}), 124.07 (CH), 123.49 (C_{quat}), 115.37 (CH), 114.17 (C_{quat}), 105.88 (CH), 61.44 (CH_3). MS (EI) (m/z (%)): 337 ($[C_{18}H_{11}NO_6]^+$, 100). Anal. Calcd. for $C_{18}H_{11}NO_6$ [337.06]: C 64.10, H 3.29, N 4.15. Found: C 63.92, H 3.16, N 4.14. IR: $\tilde{\nu}$ [cm^{-1}] = 3115 (w), 2360 (w), 1732 (vs), 1582 (s), 1543 (w), 1510 (s), 1450 (w), 1421 (s), 1344 (m), 1310 (m), 1273 (w), 1209 (s), 1186 (w),

1153 (s), 1093 (m), 1043 (m), 1026 (m), 1011 (m), 989 (w), 955 (w), 905 (w), 870 (m), 827 (s), 810 (m), 797 (m), 781 (w), 750 (s), 721 (w), 700 (m), 650 (w), 604 (m).

2.1.3. 4-(9-Methoxy-7-oxo-7*H*-furo[3,2-*g]*chromen-4-yl)benzaldehyde (11c)

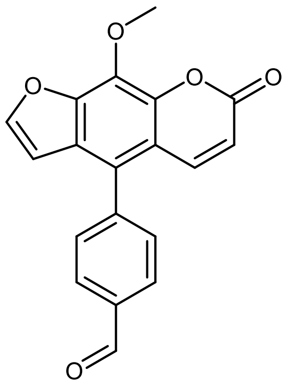

Procedure A:

The column chromatographic purification was carried out with petroleum ether/ethyl acetate (1:1). The yellow solid was recrystallized in ethanol. Yield: 138 mg (0.430 mmol, 43%) yellowish solid.

R_f = 0.80 (*n*-hexane/ethyl acetate, 1:5). Mp 219 °C. ^{1}H NMR: (300 MHz, $CDCl_3$) δ = 10.14 (s, 1 H), 8.10 – 8.02 (m, 2 H), 7.77 – 7.67 (m, 2 H), 7.58 (d, *J* = 8.1 Hz, 2 H), 6.63 (d, *J* = 2.3 Hz, 1 H), 6.34 (d, *J* = 9.9 Hz, 1 H), 4.35 (s, 3 H). ^{13}C NMR: (75 MHz, $CDCl_3$) δ = 191.53 (CH), 159.94 (C_{quat}), 147.04 (CH), 146.89 (C_{quat}), 143.63 (C_{quat}), 141.86 (C_{quat}), 141.75 (CH), 135.95 (C_{quat}), 132.77 (C_{quat}), 131.12 (CH), 130.06 (CH), 125.81 (C_{quat}), 124.84 (C_{quat}), 115.00 (CH), 114.17 (C_{quat}), 106.14 (CH), 61.44 (CH_3). MS (EI) (m/z (%)): 320 ($[C_{19}H_{12}O_5]^+$, 100), 305 ($[C_{18}H_9O_5]^+$, 26), 277 ($[C_{18}H_{13}O_3]^+$, 17). Anal. Calcd. for $C_{19}H_{12}O_5$ [320.3]: C 71.25, H 3.78. Found: C 70.95 , H 3.75. IR: $\tilde{\nu}$ [cm^{-1}] = 2359 (w), 1730 (m), 1697 (m), 1587 (m), 1468 (w), 1423 (w), 1362 (w), 1302 (w), 1207 (m), 1148 (s), 1088 (m), 1034 (m), 997 (w), 959 (w), 833 (m), 797 (w), 764 (s), 729 (w), 691 (w).

2.1.4. 9-Methoxy-4-(pyridin-4-yl)-7*H*-furo[3,2-*g*]chromen-7-one (11d)

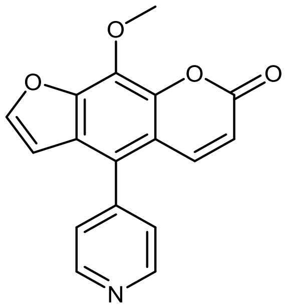

Procedure A:

The column chromatographic purification was carried out with petroleum ether/ethyl acetate (3:1 to 0:1). The yellow solid was recrystallized in ethanol. Yield: 96.1 mg (0.328 mmol, 50%) yellowish solid.

R_f = 0.33 (*n*-hexane/ethyl acetate, 1:5). Dec. 254 °C. ^{1}H NMR: (300 MHz, $CDCl_3$) δ = 8.83 – 8.76 (m, 2 H), 7.77 – 7.68 (m, 2 H), 7.38 – 7.30 (m, 2 H), 6.64 (d, *J* = 2.3 Hz, 1 H), 6.36 (d, *J* = 9.9 Hz, 1 H), 4.35 (s, 3 H). ^{13}C NMR: (75 MHz, $CDCl_3$) δ = 159.82 (C_{quat}), 150.35 (CH), 147.19 (CH), 146.85 (C_{quat}), 143.72 (C_{quat}), 143.57 (C_{quat}), 141.37 (CH), 133.00 (C_{quat}), 125.65 (C_{quat}), 125.16 (CH), 123.09 (C_{quat}), 115.28 (CH), 113.98 (C_{quat}), 105.95 (CH), 61.43 (CH_3). MS (EI) (m/z (%)): 293 ($[C_{17}H_{11}NO_4]^+$, 100), 278 ($[C_{16}H_8NO_4]^+$, 28), 250 ($[C_{16}H_{12}NO_2]^+$, 25). Anal. Calcd. for $C_{17}H_{11}NO_4$ [293.28]: C 69.62, H 3.78, N 4.78. Found: C 69.68, H 3.95, N 4.49. IR: $\tilde{\nu}$ [cm^{-1}] = 3138 (w), 3065 (w), 1717 (vs), 1585 (s), 1468 (w), 1422 (m), 1373 (m), 1329 (w), 1310 (m), 1275 (w), 1205 (w), 1153 (s), 1092 (s), 1034 (m), 1009 (m), 993 (w), 957 (w), 883 (w), 868 (w), 835 (s), 824 (w), 762 (s), 685 (s), 646 (w), 608 (m).

2.1.5. 4-(4-(Dimethylamino)phenyl)-9-methoxy-7*H*-furo[3,2-*g*]chromen-7-one (11e)

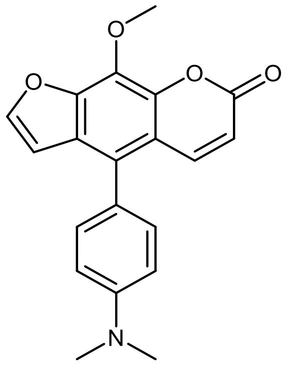

Procedure A:

The column chromatographic purification was carried out with petroleum ether/ethyl acetate (3:1). The yellow solid was recrystallized in ethanol. Yield: 199 mg (0.593 mmol, 59%) yellow solid.

R$_f$ = 0.50 (*n*-hexane/ethyl acetate, 3:1). Mp 217 °C. ^{1}H NMR: (600 MHz, acetone-d$_6$) δ = 7.97 – 7.90 (m, 2 H), 7.29 (d, *J* = 8.8 Hz, 2 H), 6.93 (d, *J* = 8.7 Hz, 2 H), 6.80 (d, *J* = 2.2 Hz, 1 H), 6.30 (d, *J* = 9.9 Hz, 1 H), 4.24 (s, 3 H), 3.05 (s, 6 H). ^{13}C NMR: (150 MHz, acetone-d$_6$) δ = 160.48 (C$_{quat}$), 151.52 (C$_{quat}$), 148.37 (C$_{quat}$), 48.01 (CH), 145.18 (C$_{quat}$), 143.92 (CH), 132.37 (C$_{quat}$), 132.07 (CH), 129.19 (C$_{quat}$), 126.63 (C$_{quat}$), 123.66 (C$_{quat}$), 115.19 (C$_{quat}$), 114.61 (CH), 113.25 (CH), 107.69 (CH), 61.75 (CH$_3$), 40.55 CH$_3$). ESI-MS: calc. for $(C_{20}H_{17}NO_4+H)^+$ m/z = 336.4 (100%). Found: 336.3 (100%). HR-MS (ESI): calcd. for $(C_{20}H_{17}NO_4+H)^+$ m/z = 336.1230. Found: 336.1234 (100%). HPLC (6.9 min): 99%. IR: $\tilde{\nu}$ [cm^{-1}] =1724 (s), 1607 (m), 1585 (s), 1526 (m), 1468 (m), 1449 (m), 1414 (m), 1362 (s), 1310 (m), 1265 (w), 1234 (w), 1196 (m), 1142 (s), 1086 (s), 1024 (m), 999 (m), 939 (m), 901 (w), 878 (w), 858 (m), 824 (s), 799 (w), 754 (s), 725 (m).

2.1.6. 4-(4-((Dimethylamino)methyl)phenyl)-9-methoxy-7*H*-furo[3,2-*g*]chromen-7-one (11f)

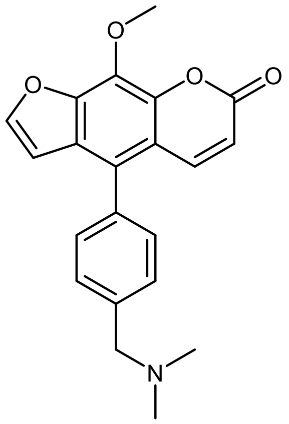

Procedure A:

The column chromatographic purification was carried out with petroleum ether/ethyl acetate (3:1 to 0:1). The yellow solid was recrystallized in hexane. Yield: 218 mg (0.624 mmol, 31%) yellowish crystals.

R$_f$ = 0.10 (*n*-hexane/ethyl acetate, 1:1). Mp 111 °C. ^{1}H NMR: (300 MHz, CDCl$_3$) δ = 7.79 (d, *J* = 9.9 Hz, 1 H), 7.65 (d, *J* = 2.2 Hz, 1 H), 7.46 (d, *J* = 8.0 Hz, 2 H), 7.34 – 7.30 (m, 2 H), 6.65 (d, *J* = 2.2 Hz, 1 H), 6.27 (d, *J* = 9.8 Hz, 1 H), 4.30 (s, 3 H), 3.51 (s, 2 H), 2.31 (s, 6 H). ^{13}C NMR (75 MHz, CDCl$_3$) δ = 160.34 (C$_{quat}$), 147.05 (C$_{quat}$), 146.50 (CH), 143.69 (C$_{quat}$), 142.68 (CH), 139.22 (C$_{quat}$), 134.20 (C$_{quat}$), 132.04 (C$_{quat}$), 130.25 (CH), 129.42 (CH), 126.78 (C$_{quat}$), 125.86 (C$_{quat}$), 114.25 (C$_{quat}$), 114.16 (CH), 106.60 (CH), 64.01 (CH$_2$), 61.42 (CH$_3$), 45.52 (CH$_3$). ESI-MS: calc. for $(C_{21}H_{19}NO_4+H)^+$ m/z = 350.4 (100%). Found: 350 (100%). Anal. Calcd. for $C_{21}H_{19}NO_4$ [349.39]: C 72.19, H 5.48, N 4.01. Found: C 71.97, H 5.51, N 3.90. IR: $\tilde{\nu}$ [cm^{-1}] = 2939 (w), 2765 (w), 1728 (s), 1589 (w), 1468 (m), 1412 (w), 1362 (m),

1308 (m), 1204 (w), 1175 (w), 1152 (s), 1090 (s), 1026 (m), 1003 (w), 961 (w), 901 (w), 870 (w), 837 (m), 802 (w), 758 (s), 727 (w), 673 (m).

2.1.7. 4-(9-Methoxy-7-oxo-7*H*-furo[3,2-*g*]chromen-4-yl)benzoic acid (11g)

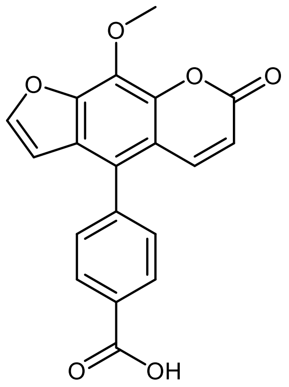

Procedure C:

The column chromatographic purification was carried out with petroleum ether/ethyl acetate (3:1 to 0:1). The yellow solid was recrystallized in acetone. Yield: 272 mg (0.810 mmol, 54%) yellowish solid.

R_f = 0.28 (*n*-hexane/ethyl acetate, 3:1). Dec. 252 °C. ^{1}H NMR: (600 MHz, DMSO-d_6) δ = 13.16 (s, 1 H), 8.19 – 8.08 (m, 3 H), 7.80 (d, *J* = 9.9 Hz, 1 H), 7.56 (d, *J* = 8.0 Hz, 2 H), 6.80 (d, *J* = 2.3 Hz, 1 H), 6.41 (d, *J* = 9.9 Hz, 1 H), 4.23 (s, 3 H). ^{13}C NMR: (150 MHz, DMSO-d_6) δ = 167.51 (C_{quat}), 159.77 (C_{quat}), 148.92 (CH), 146.73 (C_{quat}), 143.56 (C_{quat}), 142.85 (CH), 139.71 (C_{quat}), 132.23 (C_{quat}), 131.10 (C_{quat}), 131.05 (CH), 130.21 (CH), 125.86 (C_{quat}), 125.80 (C_{quat}), 115.13 (CH), 114.30 (C_{quat}), 106.71 (CH), 61.59 (CH_3). HR-MS (ESI): calcd. for $(C_{19}H_{12}O_6+H)^+$ m/z = 337.0707. Found: 337.0709 (100%). HPLC (6.0 min min): 99%. IR: $\tilde{\nu}$ [cm^{-1}] = 2960 (w), 1712 (s), 1585 (s), 1470 (w), 1422 (w), 1306 (w), 1246 (w), 1211 (w), 1119 (w), 1101 (m), 1086 (s), 1034 (w), 1009 (m), 988 (m), 903 (w), 883 (w), 872 (w), 843 (m), 783 (m), 754 (s), 710 (m), 683 (w).

2.2. General procedure: Sonogashira coupling

Procedure A:

5-Bromo-8-methoxypsoralen (**7**) was placed in dry THF under nitrogen atmosphere in a screw-cap Schlenk tube. The arylalkyne, tris(dibenzylideneacetone)dipalladium(0), cataCXium PtB®, triethylamine and copper iodide were added and the solution was then degassed with nitrogen for 5 min. The reaction mixture was stirred at 80 °C for 48 h. After cooling, water was added (10 mL) and the aqueous phase extracted with ethyl acetate (3 x 30 mL). The organic layers were combined and dried with anhydrous sodium sulfate. The mixture was adsorbed on Celite® and purified by column chromatography (petroleum ether/acetone).

Table 4. Experimental details of Sonogashira coupling of 5-substituted 8-methoxypsoralens **13**.

entry	5-Br-8-MOP (**7**)	alkyne **12**	$Pd_2(dba)_3$	cataCXium PtB®	CuI	NEt_3	THF	product **13**
1	246 mg (0.839 mmol)	**12a**, 116 mg (0.913 mmol)	3.74 mg (4.20 µmol)	4.98 mg (17.3 µmol)	6.32 mg (33.2 µmol)	173 µL (1.25 mmol)	3 mL	**13a**, 61.0 mg (179 µmol) 21%
2	139 mg (0.471 mmol)	**12b**, 76.5 mg (0.520 mmol)	2.16 mg (2.36 µmol)	2.71 mg (9.46 µmol)	3.59 mg (18.9 µmol)	98 µL (0.707 mmol)	3 mL	**13b**, 43.0 mg (119 µmol) 25%
3	295 mg (1.00 mmol)	**12c**, 143 mg (1.10 mmol)	4.58 mg (5.00 µmol)	5.75 mg (20.0 µmol)	7.62 mg (40.0 µmol)	208 µL (1.50 mmol)	3 mL	**13c**, 156 mg (453 µmol) 45%
4	155 mg (0.529 mmol)	**12d**, 91.0 mg (0.583 mmol)	4.85 mg (5.30 µmol)	6.06 mg (21.2 µmol)	4.04 mg (21.2 µmol)	110 µL (0.794mmol)	3 mL	**13d**, 107 mg (337 µmol) 64%
5	528 mg (1.79 mmol)	**12e**, 286 mg (1.97 mmol)	8.19 mg (8.95 µmol)	10.3 mg (35.8 mmol)	13.6 mg (71.6 mmol)	372 µL (2.69 mmol)	3 mL	**13e**, 400 mg (1.13 mmol) 63%

2.2.1. 4-((9-Methoxy-7-oxo-7*H*-furo[3,2-*g*]chromen-4-yl)ethynyl)benzonitrile (13a)

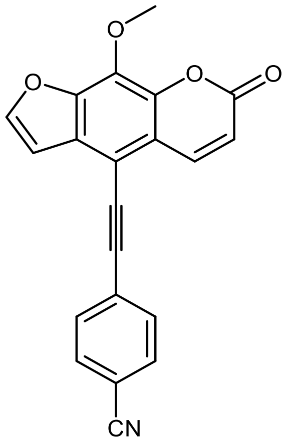

The column chromatographic purification was carried out with petroleum ether/acetone (3:1). The yellow solid was recrystallized in ethanol. Yield: 61.0 mg (0.179 mmol, 21%) yellow solid.

R_f = 0.40 (*n*-hexane/ethyl acetate, 2:1). Mp 251 °C. ^{1}H NMR: (300 MHz, $CDCl_3$) δ = 8.24 (d, *J* = 9.8 Hz, 1 H), 7.77 (d, *J* = 2.2 Hz, 1 H), 7.70 (s, 4 H), 7.02 (d, *J* = 2.2 Hz, 1 H), 6.49 (d, *J* = 9.7 Hz, 1 H), 4.38 (s, 3 H). ^{13}C NMR: (75 MHz, $CDCl_3$) δ = 159.85 (C_{quat}), 147.32 (CH), 146.21 (C_{quat}), 142.95 (C_{quat}), 141.94 (CH), 134.14 (C_{quat}), 132.27 (CH), 132.06 (CH), 129.13 (C_{quat}), 127.31 (C_{quat}), 118.28 (C_{quat}), 117.77 (C_{quat}), 115.76 (CH), 112.23 (C_{quat}), 106.65 (CH), 104.84 (C_{quat}), 95.72 (C_{quat}), 87.39 (C_{quat}), 61.37 (CH_3). ESI-MS: calc. for $(C_{21}H_{11}NO_4+H)^+$ m/z = 342.1 (100%). Found: 342.5 (100%). HR-MS (ESI): calcd. for $(C_{21}H_{11}NO_4+H)^+$ m/z = 342.0761. Found: 342.0764. HPLC (6.3min): 99%. IR: $\tilde{\nu}$ [cm^{-1}] = 3145 (w), 2954 (w), 2359 (w), 2226 (w), 1728 (vs), 1716 (s), 1582 (vs), 1504 (m), 1472 (m), 1423 (m), 1406 (w), 1377 (m), 1315 (m), 1273 (w), 1238 (w), 1207 (m), 1153 (s), 1119 (s), 1088 (w), 1036 (m), 1020 (s), 949 (w), 880 (w), 827 (s), 750 (s), 718 (m), 704 (m), 652 (w).

2.2.2. 9-Methoxy-4-((4-nitrophenyl)ethynyl)-7*H*-furo[3,2-*g*]chromen-7-one (13b)

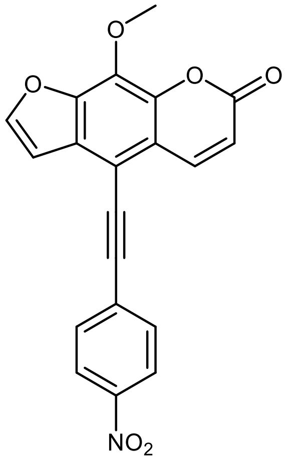

No column chromatographic purification was needed. The cooled solution was washed in hot acetone and the precipitated solid was filtered off. Yield: 43.0 mg (0.119 mmol, 25%) orange solid.

R_f = 0.30 (*n*-hexane/ethyl acetate, 3:1). Dec. 275 °C. ^{1}H NMR: (600 MHz, $CDCl_3$) δ = 8.33 – 8.22 (m, 3 H), 7.81 – 7.70 (m, 3 H), 7.04 (d, *J* = 2.2 Hz, 1 H), 6.51 (d, *J* = 9.7 Hz, 1 H), 4.39 (s, 3 H). ^{13}C NMR: (150 MHz, $CDCl_3$) δ = 159.83 (C_{quat}), 147.40 (CH), 146.20 (C_{quat}), 142.96 (C_{quat}), 141.90 (CH), 134.29 (C_{quat}), 132.30 (CH), 129.25 (C_{quat}), 123.89 (CH), 117.86 (C_{quat}), 115.87 (CH), 106.66 (CH), 104.66 (C_{quat}), 95.51 (C_{quat}), 88.28 (C_{quat}), 61.38 (CH_3).

MS (EI) (m/z (%)): 361 ($[C_{20}H_{11}NO_6]^+$, 100). HR-MS (ESI): calcd. for $(C_{20}H_{11}NO_6+H)^+$ m/z =

362.0659, 363.0693. Found: 362.0657, 363.0691. HPLC (6.0 min): 99%. IR: $\tilde{\nu}$ [cm-1] = 3107 (w), 2955 (w), 2206 (m), 1724 (vs), 1578 (vs), 1512 (m), 1470 (m), 1449 (w), 1423 (m), 1379 (w), 1341 (s), 1319 (m), 1277 (m), 1209 (m), 1155 (m), 1124 (s), 1105 (m), 1088 (w), 1038 (m), 1026 (m), 880 (w), 854 (s), 831 (m), 818 (w), 748 (s), 687 (m).

2.2.3 4-((9-Methoxy-7-oxo-7*H*-furo[3,2-*g*]chromen-4-yl)ethynyl)benzaldehyde (13c)

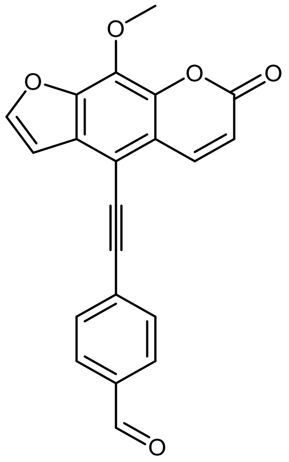

The column chromatographic purification was carried out with petroleum ether/acetone (2:1 to 0:1). The yellow solid was recrystallized in acetone. Yield: 156 mg (0.453 mmol, 45%) beige solid.

R_f = 0.83 (*n*-hexane/ethyl acetate, 2:1). Dec. 224 °C. ^{1}H NMR: (300 MHz, $CDCl_3$) δ = 10.06 (s, 1 H), 8.28 (d, *J* = 9.7 Hz, 1 H), 8.01 – 7.84 (m, 2 H), 7.81 – 7.70 (m, 3 H), 7.04 (d, *J* = 2.2 Hz, 1 H), 6.49 (d, *J* = 9.7 Hz, 1 H), 4.38 (s, 3 H). ^{13}C NMR: (75 MHz, $CDCl_3$) δ = 191.23 (CH), 159.94 (C_{quat}), 147.25 (CH), 146.27 (C_{quat}), 142.98 (C_{quat}), 142.09 (CH), 135.89 (C_{quat}), 134.01 (C_{quat}), 132.11 (CH), 129.74 (CH), 129.09 (C_{quat}), 128.61 (C_{quat}), 117.74 (C_{quat}), 115.65 (CH), 106.72 (CH), 105.26 (C_{quat}), 96.63 (C_{quat}), 86.99 (C_{quat}), 61.37 (CH_3). MS (EI) (m/z (%)): 344 ($[C_{21}H_{12}O_5]^+$, 100), 344 ($[C_{20}H_9O_5]^+$, 34), 301 ($[C_{19}H_9O_4]^+$, 34). HR-MS (ESI): calcd. for $(C_{21}H_{12}O_5+H)^+$ m/z = 345.0757. Found: 345.0756. HPLC (5.9min): 99%. IR: $\tilde{\nu}$ [cm^{-1}] = 2972 (w), 2901 (w), 2197 (w), 1732 (vs), 1669 (s), 1582 (s), 1558 (m), 1472 (m), 1423 (m), 1380 (m), 1308 (m), 1275 (w), 1207 (m), 1157 (s), 1128 (vs), 1090 (w), 1040 (s), 1024 (m), 947 (w), 843 (m), 824 (s), 752 (s), 667 (w), 606 (w).

2.2.4. 9-Methoxy-4-(pyridin-4-ylethynyl)-7*H*-furo[3,2-*g*]chromen-7-one (13d)

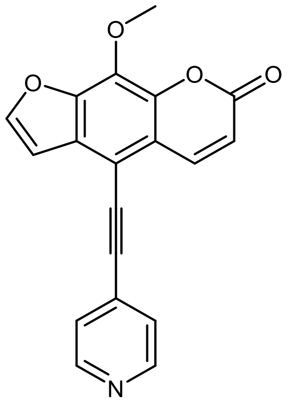

The column chromatographic purification was carried out with petroleum ether/acetone (3:1 to 1:9). Yield: 107 mg (0.337 mmol, 64%) beige solid.

R_f = 0.28 (*n*-hexane/ethyl acetate, 1:5). Dec. 207 °C. ^{1}H NMR: (600 MHz, $CDCl_3$) δ = 8.68 (m, 3 H), 8.24 (d, *J* = 9.7 Hz, 1 H), 7.77 (d, *J* = 2.2 Hz, 1 H), 7.52 – 7.42 (m, 2 H), 7.02 (d, *J* = 2.2 Hz, 1 H), 6.49 (d, *J* = 9.7 Hz, 1 H), 4.38 (s, 3 H). ^{13}C NMR: (150 MHz, $CDCl_3$) δ = 159.86 (C_{quat}), 150.02 (CH), 147.37 (CH), 146.18 (C_{quat}), 142.93 (C_{quat}), 141.94 (CH), 134.22 (C_{quat}), 130.57 (C_{quat}), 129.25 (C_{quat}), 125.39 (CH), 117.86 (C_{quat}), 115.82 (CH), 106.65 (CH), 104.65 (C_{quat}), 94.66 (C_{quat}), 87.54 (C_{quat}), 61.36 (CH_3). MS (EI) (m/z (%)): 317 ([$C_{19}H_{11}NO_4$]$^+$, 100), 302 ([$C_{18}H_8NO_4$]$^+$, 40), 274 ([$C_{17}H_8NO$]$^{3+}$, 37), 246 ([$C_{16}H_{18}NO_2$]$^+$, 17). Anal. Calcd. for $C_{19}H_{11}NO_4$ [317.30]: C 71.92, H 3.49, N 4.41. Found: C 71.98, H 3.64, N 4.30. HR-MS (ESI): calcd. for ($C_{19}H_{11}NO_4$+H)$^+$ m/z = 318.08. Found: 318.0765. IR: $\tilde{\nu}$ [cm^{-1}] =3132 (w), 3053 (w), 2949 (w), 2205 (w), 1713 (vs), 1582 (vs), 1495 (m), 1470 (m), 1423 (m), 1377 (m), 1314 (m), 1277 (m), 1206 (w), 1155 (s), 1125 (s), 1093 (m), 1018 (s), 989 (m), 947 (m), 899 (w), 878 (w), 858 (w), 826 (s), 791 (m), 762 (s), 637 (m), 617 (w).

2.2.5 4-((4-(Dimethylamino)phenyl)ethynyl)-9-methoxy-7*H*-furo[3,2-*g*]chromen-7-one (13e)

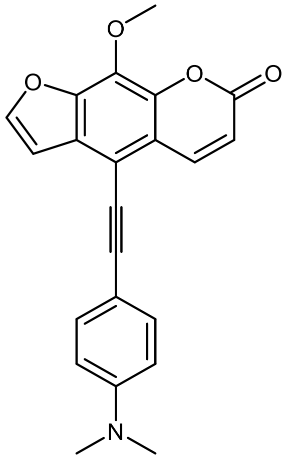

The column chromatographic purification was carried out with petroleum ether/acetone (3:1). The yellow solid was recrystallized in acetonitrile. Yield: 400 mg (1.13 mmol, 63%) yellow needle-shaped crystals.

R_f = 0.61 (*n*-hexane/ethyl acetate, 2:1). Mp 200 °C. ^1H NMR: (300 MHz, $CDCl_3$) δ = 8.31 (d, *J* = 9.7 Hz, 1 H), 7.70 (d, *J* = 2.1 Hz, 1 H), 7.47 (d, *J* = 8.6 Hz, 2 H), 7.03 (d, *J* = 2.1 Hz, 1 H), 6.71 (d, *J* = 8.5 Hz, 2 H), 6.42 (d, *J* = 9.7 Hz, 1 H), 4.32 (s, 3 H), 3.03 (s, 7 H). ^{13}C NMR: (75 MHz, $CDCl_3$) δ = 160.20 (C_{quat}), 150.54 (C_{quat}), 150.34 (C_{quat}), 146.85 (C_{quat}), 146.46 (CH), 143.35 (C_{quat}), 142.72 (C_{quat}), 132.84 (CH), 132.76 (C_{quat}), 128.10 (C_{quat}), 117.10 (C_{quat}), 114.64 (CH), 112.18 (C_{quat}), 111.96 (CH), 106.98 (CH), 77.15 (C_{quat}), 61.33 (CH_3), 40.31 (CH_3), 40.16 (CH_3). MS (EI) (m/z (%)): 359 ($[C_{22}H_{17}NO_4]^+$, 100), 344 ($[C_{20}H_9O_5]^+$, 51), 316 ($[C_{20}H_{12}O_4]^+$, 33). Anal. Calcd. for $C_{22}H_{17}NO_4$ [359.38]: C 73.53, H 4.77, N 3.90. Found: C 73.49, H 4.79, N 3.85. IR: $\tilde{\nu}$ [cm^{-1}] = 2900 (w), 2358 (w), 2195 (w), 1719 (s), 1601 (m), 1585 (s), 1518 (s), 1449 (w), 1368 (s), 1317 (w), 1271 (w), 1233 (w), 1200 (m), 1152 (s), 1119 (s), 1071 (w), 1032 (s), 1015 (m), 1003 (m), 945 (w), 880 (w), 810 (s), 779 (w), 754 (s), 721 (w), 704 (w), 654 (w).

2.3. General procedure: Heck coupling

Procedure A:

5-Bromo-8-methoxypsoralen (**7**) was placed in dry DMF under nitrogen atmosphere in a microwave tube. The arylvinyl, tris(dibenzylideneacetone)dipalladium(0), cataCXium PtB® and triethylamine were added and the solution was then degassed with nitrogen for 5 min. The reaction mixture was stirred at 100 °C for 8 h in the microwave. After cooling, water was added (10 mL) and the aqueous phase extracted with dichloromethane (3 x 30 mL). The organic layers were combined and dried with anhydrous sodium sulfate. The mixture was adsorbed on Celite® and purified by column chromatography (petroleum ether/acetone).

Table 5. Experimental details of the Heck coupling of 5-substituted 8-methoxypsoralens **15**.

entry	5-Br-8-MOP (**7**)	alkyne **14**	$Pd_2(dba)_3$	cataCXium PtB®	NEt_3	DMF	product **15** (yield)
1	295 mg (1.00 mmol)	**14a**, 160 mg (1.20 mmol)	4.58 mg (5.00 µmol)	5.75 mg (20.0 µmol)	208 µL (1.50 mmol)	3 mL	**15a**, 135 mg (393 µmol) 39%
2	115 mg (0.39 mmol)	**14b**, 70 mg (0.468 mmol)	1.75 mg (1.95 µmol)	4.14 mg (7.80 µmol)	81.1 µL (0.59 mmol)	1.5 mL	**15b**, 98.0 mg (270 µmol) 69%
3	295 mg (1.00 mmol)	**14c**, 150 mg (1.20 mmol)	4.58 mg (5.00 µmol)	5.75 mg (20.0 µmol)	208 µL (1.50 mmol)	6 mL	**15c**, 239 mg (690 µmol) 69%
4	295 mg (1.00 mmol)	**14d**, 128 µL (1.20 mmol)	9.16 mg (10.0 µmol)	11.5 mg (40.0 µmol)	208 µL (1.50 mmol)	6 mL	**15d**, 91.7 mg (287 µmol) 29%
5	520 mg (1.76 mmol)	**14e**, 311 mg (2.11 mmol)	8.05 mg (8.80 µmol)	10.1 mg (35.2 mmol)	365 µL (2.64 mmol)	5 mL	**15e**, 369 mg (1.02 mmol) 57%

2.3.1. (*E*)-4-(2-(9-Methoxy-7-oxo-7*H*-furo[3,2-*g*]chromen-4-yl)vinyl)benzonitrile (15a)

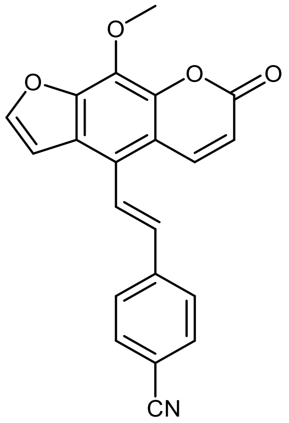

The column chromatographic purification was carried out with petroleum ether/acetone (2:1 to 0:1). The yellow solid was recrystallized in ethanol. Yield: 135 mg (0.393 mmol, 39%) yellow solid.

R_f = 0.36 (*n*-hexane/ethyl acetate, 2:1). Dec. 255 °C. ^{1}H NMR: (300 MHz, CD_2Cl_2/TFA) δ = 8.51 (d, *J* = 9.9 Hz, 1 H), 8.35 – 8.30 (m, 2 H), 7.88 (d, *J* = 2.3 Hz, 1 H), 7.82 – 7.72 (m, 3 H), 7.23 – 7.15 (m, 2 H), 6.67 (d, *J* = 9.8 Hz, 1 H), 4.37 (s, 3 H). ^{13}C NMR: (75 MHz, CD_2Cl_2/TFA) δ = 148.32 (C_{quat}), 148.00 (C_{quat}), 144.79 (CH), 143.17 (C_{quat}), 142.18 (C_{quat}), 135.18 (CH), 133.44 (CH), 132.82 (C_{quat}), 127.68 (CH), 126.60 (CH), 125.82 (CH), 122.69 (C_{quat}), 118.15 (C_{quat}), 114.89 (C_{quat}), 113.16 (CH), 110.28 (C_{quat}), 106.78 (CH), 62.05 (CH_3). MS (EI) (m/z (%)): 343 ([$C_{21}H_{13}NO_4$]$^+$, 100), 300 ([$C_{19}H_{10}O_4$]$^+$, 48). HR-MS (ESI): calcd. for ($C_{21}H_{13}NO_4$+H)$^+$ m/z = 344.0917. Found: 344.0919. HPLC (3.7 min): 99%. IR: $\tilde{\nu}$ [cm^{-1}] = 3122 (w), 2947 (m), 2222 (m), 1717 (vs), 1585 (s), 1506 (m), 1472 (m), 1418 (m), 1373 (m), 1327 (w), 1303 (m), 1206 (m), 1124 (vs), 1096 (w), 1036 (s), 1013 (w), 993 (w), 953 (m), 868 (m), 829 (m), 812 (s), 772 (m), 745 (s), 725 (m), 679 (w), 652 (w), 637 (m), 609 (m).

2.3.2. (*E*)-9-Methoxy-4-(4-nitrostyryl)-7*H*-furo[3,2-*g*]chromen-7-one (15b)

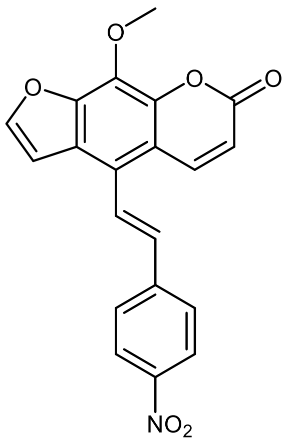

The column chromatographic purification was carried out with dichloromethane. The red solid was washed with acetone and ethanol. Yield: 98.0 mg (0.270 mmol, 69%) red solid.

R_f = 0.83 (*n*-hexane/ethyl acetate, 1:5). Dec. 309 °C. ^{1}H NMR: (300 MHz, CD_2Cl_2/TFA) δ = 8.50 (d, *J* = 9.9 Hz, 1 H), 8.35 – 8.26 (m, 2 H), 7.88 (d, *J* = 2.3 Hz, 1 H), 7.83 – 7.69 (m, 3 H), 7.23 – 7.10 (m, 2 H), 6.67 (d, *J* = 9.8 Hz, 1 H), 4.37 (s, 3 H). ^{13}C NMR: (75 MHz, CD_2Cl_2/TFA) δ = 148.43 (CH), 148.03 (C_{quat}), 147.50 (C_{quat}), 145.06 (CH), 143.72 (C_{quat}), 143.13 (C_{quat}), 134.91 (CH), 132.75 (C_{quat}), 127.79 (CH), 126.76 (C_{quat}), 126.35 (CH), 125.22 (CH), 124.76 (C_{quat}), 122.81 (C_{quat}), 114.94 (C_{quat}), 113.04 (C_{quat}), 106.78 (CH), 62.11 (CH_3). MS (EI) (m/z (%)): 363 ([$C_{20}H_{13}NO_6$]$^+$, 100), 320 ([$C_{19}H_{14}NO_4$]$^+$, 32). HR-MS (ESI): calcd. for ($C_{20}H_{13}NO_6$+H)$^+$ m/z = 364.0816. Found: 364.0818. HPLC (3.7 min): 99%. IR: $\tilde{\nu}$ [cm^{-1}] = 3116 (w), 2953 (w), 2426 (w), 1717 (vs), 1574 (vs), 1504 (s), 1468 (m), 1423 (m), 1373 (w), 1337 (s), 1325 (s), 1298 (m), 1273 (w), 1209 (m), 1167 (m), 1140 (s), 1107 (m), 1094 (m), 1043 (s), 970 (m), 876 (m), 858 (w), 837 (w), 820 (s), 777 (w), 758 (s), 745 (m), 687 (m), 644 (w).

2.3.3. (*E*)-4-(2-(9-Methoxy-7-oxo-7*H*-furo[3,2-*g*]chromen-4-yl)vinyl)benzaldehyde (15c)

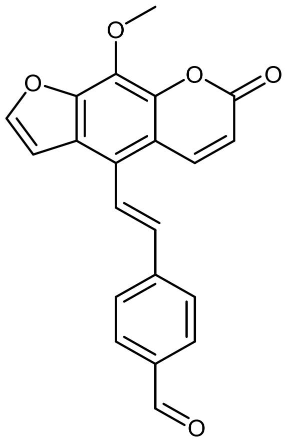

The column chromatographic purification was carried out with petrolether/acetone (2:1). The yellow solid was recrystallized in ethanol. Yield: 239 mg (0.690 mmol, 69%) yellow solid.

R_f = 0.44 (*n*-hexane/ethyl acetate, 2:1). Dec. 237 °C. ^{1}H NMR: (300 MHz, $CDCl_3$/TFA) δ = 9.96 (s, 1 H), 8.42 (d, *J* = 9.9 Hz, 1 H), 8.04 (d, *J* = 8.3 Hz, 2 H), 7.83 (d, *J* = 2.3 Hz, 1 H), 7.80 – 7.69 (m, 3 H), 7.16 – 7.10 (m, 2 H), 6.63 (d, *J* = 9.9 Hz, 1 H), 4.36 (s, 3 H). ^{13}C NMR: (75 MHz, $CDCl_3$/TFA) δ = 195.82 (CH), 164.69 (C_{quat}), 148.18 (C_{quat}), 147.77 (CH), 147.47 (C_{quat}), 143.96 (CH), 142.73 (C_{quat}), 135.18 (CH), 134.61 (C_{quat}), 132.66 (C_{quat}), 131.66 (CH), 127.40 (CH), 127.32 (C_{quat}), 126.14 (C_{quat}), 125.69 (CH), 122.00 (C_{quat}), 114.46 (C_{quat}), 113.10 (CH), 106.46 (CH), 61.64 (CH_3). ESI-MS: calcd. for $(C_{21}H_{14}O_5+H)_+$ m/z = 347.35 (100%). Found: 347.3 (100%). HR-MS (ESI): calcd. for $(C_{21}H_{14}O_6+H)^+$ m/z = 347.0914. Found: 347.0916. HPLC (6.9min): 99%. IR: $\tilde{\nu}$ [cm^{-1}] = 3165 (vw), 3165 (vw), 3030 (vw), 2955 (vw), 2795 (vw), 2718 (vw), 1732 (vs), 1692 (s), 1601 (m), 1574 (vs), 1466 (m), 1422 (m), 1377 (m), 1329 (m), 1302 (s), 1275 (w), 1258 (w), 1217 (m), 1206 (m), 1167 (s), 1146 (vs), 1134 (s), 1091 (m), 1053 (s), 1040 (s), 995 (w), 964 (m), 947 (m), 897 (w), 876 (w), 864 (w), 849 (m), 808 (s), 797 (s), 779 (m), 758 (s), 729 (w), 718 (m), 660 (w), 629 (w).

2.3.4. (*E*)-9-Methoxy-4-(2-(pyridin-4-yl)vinyl)-7*H*-furo[3,2-*g*]chromen-7-one (15d)

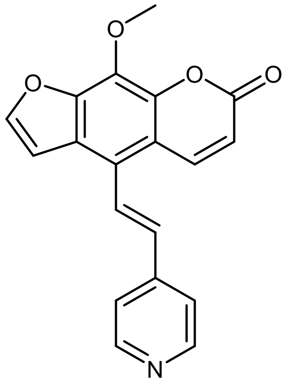

The column chromatographic purification was carried out with petrolether/acetone (2:1 to 0:1). The yellow solid was recrystallized in ethanol. Yield: 91.7 mg (0.287 mmol, 29%) yellow solid.

R_f = 0.32 (*n*-hexane/ethyl acetate, 1:5). Dec. 243 °C. ^{1}H NMR: (300 MHz, DMF-d_7) δ = 8.75 (d, *J* = 10.0 Hz, 1 H), 8.67 – 8.62 (m, 2 H), 8.29 – 8.21 (m, 2 H), 7.86 – 7.82 (m, 2 H), 7.60 (d, *J* = 2.3 Hz, 1 H), 7.35 (d, *J* = 16.4 Hz, 1 H), 6.52 (d, *J* = 10.0 Hz, 1 H), 4.31 (s, 3 H). ^{13}C NMR: (75 MHz, DMF-d_7) δ = 159.54 (C_{quat}), 150.29 (CH), 148.31 (CH), 147.52 (C_{quat}), 144.42 (C_{quat}), 143.80 (C_{quat}), 142.10 (CH), 133.07 (CH), 132.52 (C_{quat}), 126.86 (CH), 124.57 (C_{quat}), 121.89 (CH), 121.41 (C_{quat}), 114.87 (C_{quat}), 114.26 (CH), 107.13 (CH), 61.08 (CH_3). MS (EI) (m/z (%)):319 ($[C_{19}H_{13}NO_4]^+$, 100), 276 ($[C_{18}H_{14}NO_2]^+$, 45). HR-MS (ESI): calcd. for $(C_{19}H_{13}NO_4+H)^+$ m/z = 320.0917. Found: 320.0917. HPLC (5.7 min): 99%. IR: $\tilde{\nu}$[cm^{-1}] = 3051 (w), 2398 (w), 1719 (vs), 1578 (vs), 1497 (w), 1468 (m), 1415 (m), 1375 (m), 1338 (m), 1313 (m), 1278 (w), 1220 (w), 1169 (m), 1144 (vs), 1090 (w), 1045 (s), 1028 (m), 989 (m), 970 (m), 947 (s), 899 (m), 874 (m), 824 (s), 793 (s), 766 (s), 644 (w), 621 (m).

2.3.5. (*E*)-4-(4-(Dimethylamino)styryl)-9-methoxy-7*H*-furo[3,2-*g*]chromen-7-one (15e)

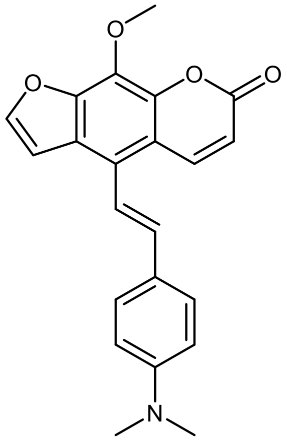

The column chromatographic purification was carried out with petrolether/acetone (3:1). The yellow solid was recrystallized in methanol/ethyl acetate. Yield: 369 mg (1.02 mmol, 57%) yellow solid.

R_f = 0.55 (*n*-hexane/ethyl acetate, 3:1). Mp 183 °C. ^{1}H NMR: (300 MHz, CD_2Cl_2) δ = 8.12 (d, *J* = 9.9 Hz, 1 H), 7.64 (d, *J* = 2.2 Hz, 1 H), 7.42 – 7.31 (m, 2 H), 7.20 (d, *J* = 16.2 Hz, 1 H), 7.01 (d, *J* = 2.3 Hz, 1 H), 6.87 (d, *J* = 16.2 Hz, 1 H), 6.66 (d, *J* = 8.6 Hz, 2 H), 6.22 (d, *J* = 9.9 Hz,

1 H), 4.16 (s, 3 H), 2.92 (s, 6 H). ^{13}C NMR: (75 MHz, CD_2Cl_2) δ = 160.51 (C_{quat}), 151.07 (C_{quat}), 148.16 (C_{quat}), 146.70 (CH), 144.34 (C_{quat}), 141.99 (CH), 136.85 (CH), 131.78 (C_{quat}), 128.16 (CH), 125.22 (C_{quat}), 124.50 (C_{quat}), 117.19 (CH), 114.17 (C_{quat}), 113.87 (CH), 112.57 (CH), 107.14 (CH), 61.74 (CH_3), 40.53 (CH_3). ESI-MS: calcd. for $(C_{22}H_{19}NO_4+H)_+$ m/z =

362.41 (100%). Found: 362.1 (100%). HR-MS (ESI): calcd. for $(C_{22}H_{19}NO_4+H)^+$ m/z = 362.1387. Found: 362.1385. HPLC (7.9 min): 99%. IR: $\tilde{\nu}$ [cm^{-1}] = 2891 (w), 1730 (s), 1601 (m), 1521 (s), 1468 (m), 1445 (m), 1425 (m), 1364 (m), 1303 (m), 1273 (w), 1186 (m), 1165 (m), 1144 (s), 1093 (w), 1049 (s), 1026 (m), 995 (m), 966 (m), 947 (m), 893 (w), 835 (m), 808 (s), 777 (w), 750 (s), 725 (m), 681 (w), 654 (w).

3. NMR spectra

3.1. NMR spectra of starting materials

3.1.1. 2-Methoxybenzene-1,3-diol (2)

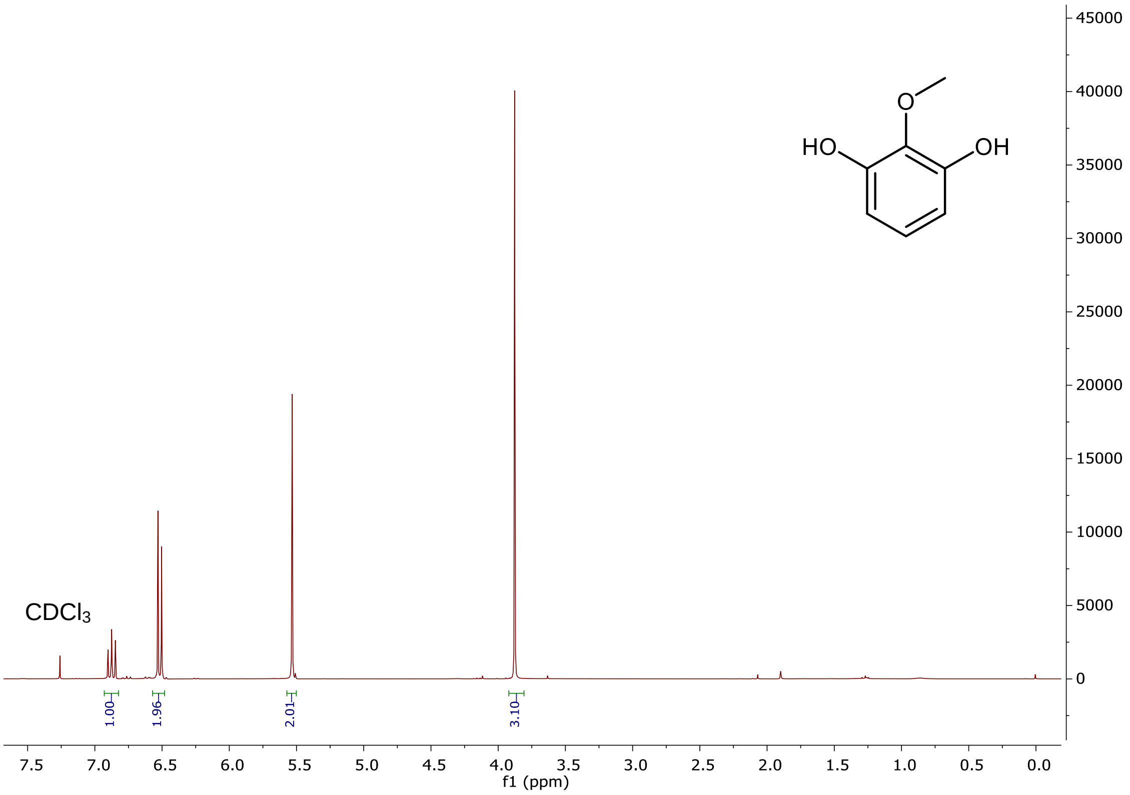

^{1}H NMR spectrum (300 MHz) of **2**; recorded in $CDCl_3$ at 298 K.

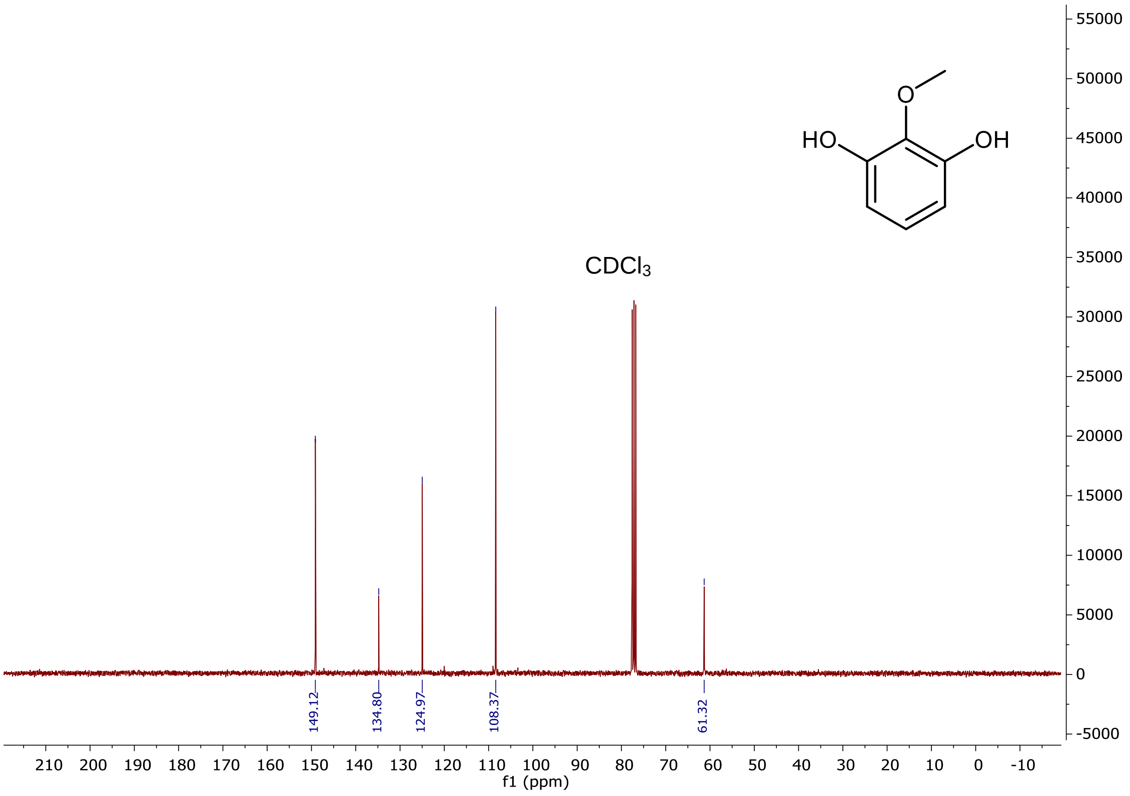

^{13}C NMR spectrum (75 MHz) of **2**; recorded in $CDCl_3$ at 298 K.

3.1.2. 7-Hydroxy-8-methoxycoumarin (3)

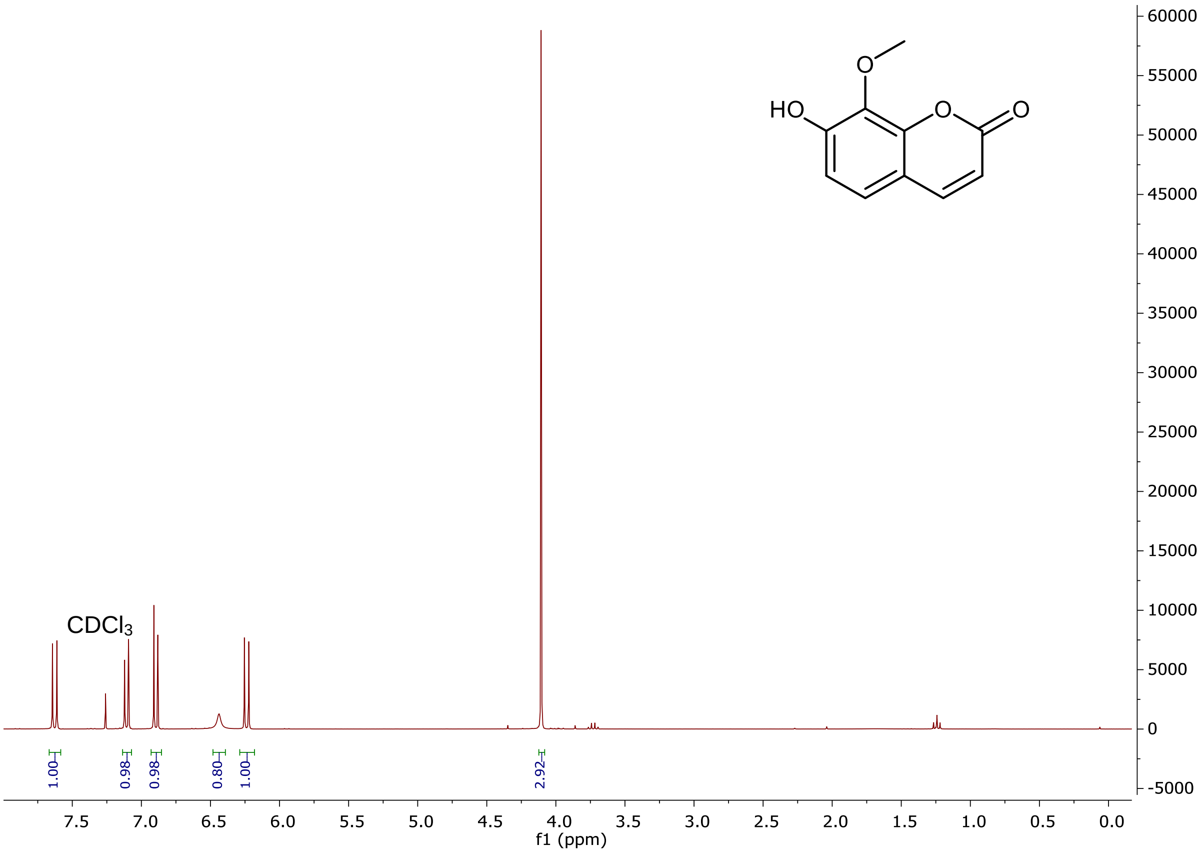

^{1}H NMR spectrum (300 MHz) of **3**; recorded in $CDCl_3$ at 298 K.

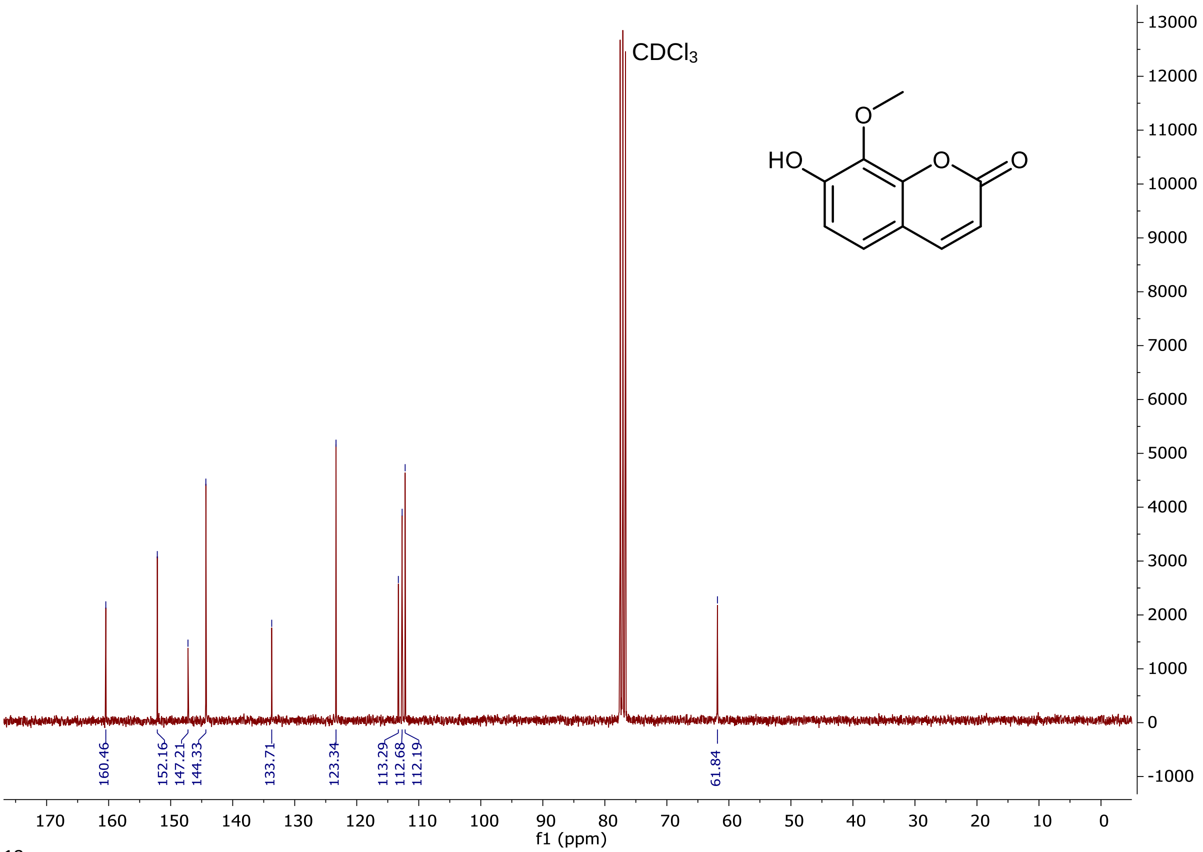

^{13}C NMR spectrum (75 MHz) of **3**; recorded in $CDCl_3$ at 298 K.

3.1.3. 7-(2,2-Diethoxyethoxy)-8-methoxy-2*H*-chromen-2-one (4)

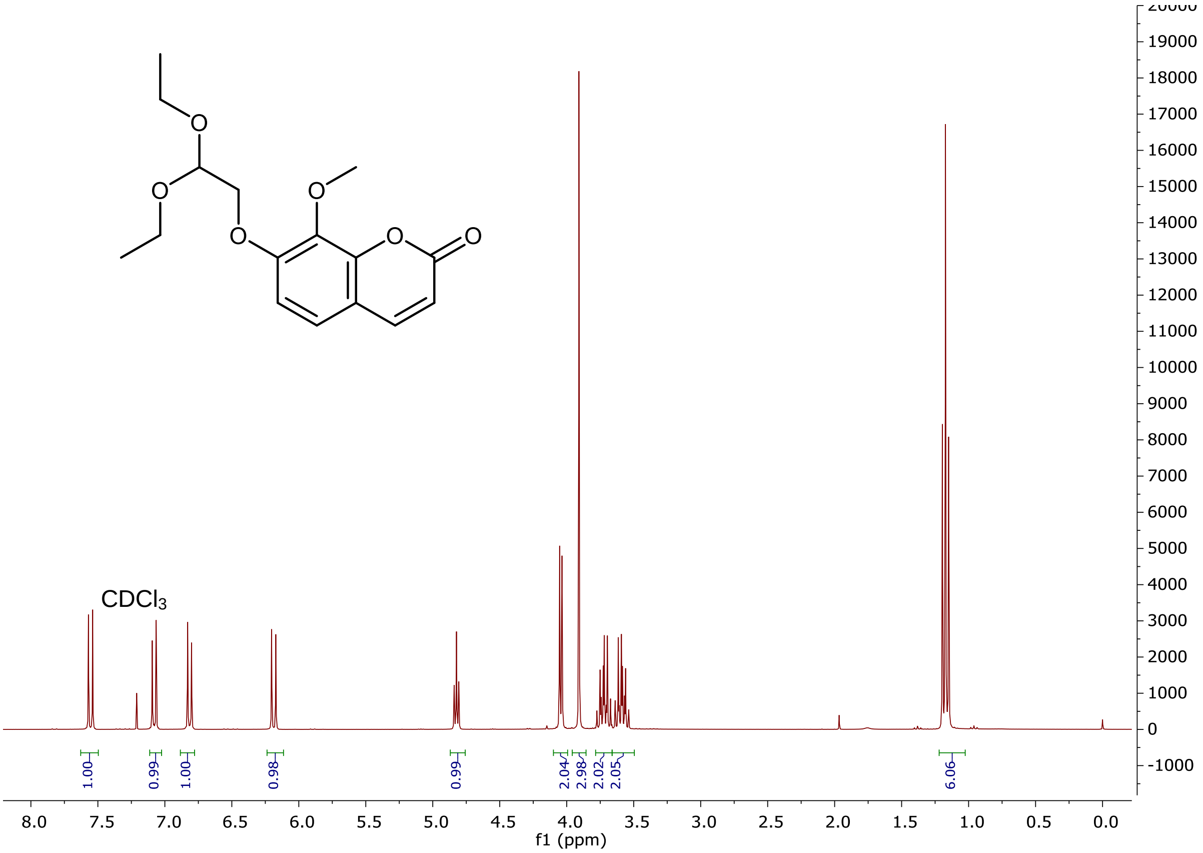

^{1}H NMR spectrum (300 MHz) of **4**; recorded in $CDCl_3$ at 298 K.

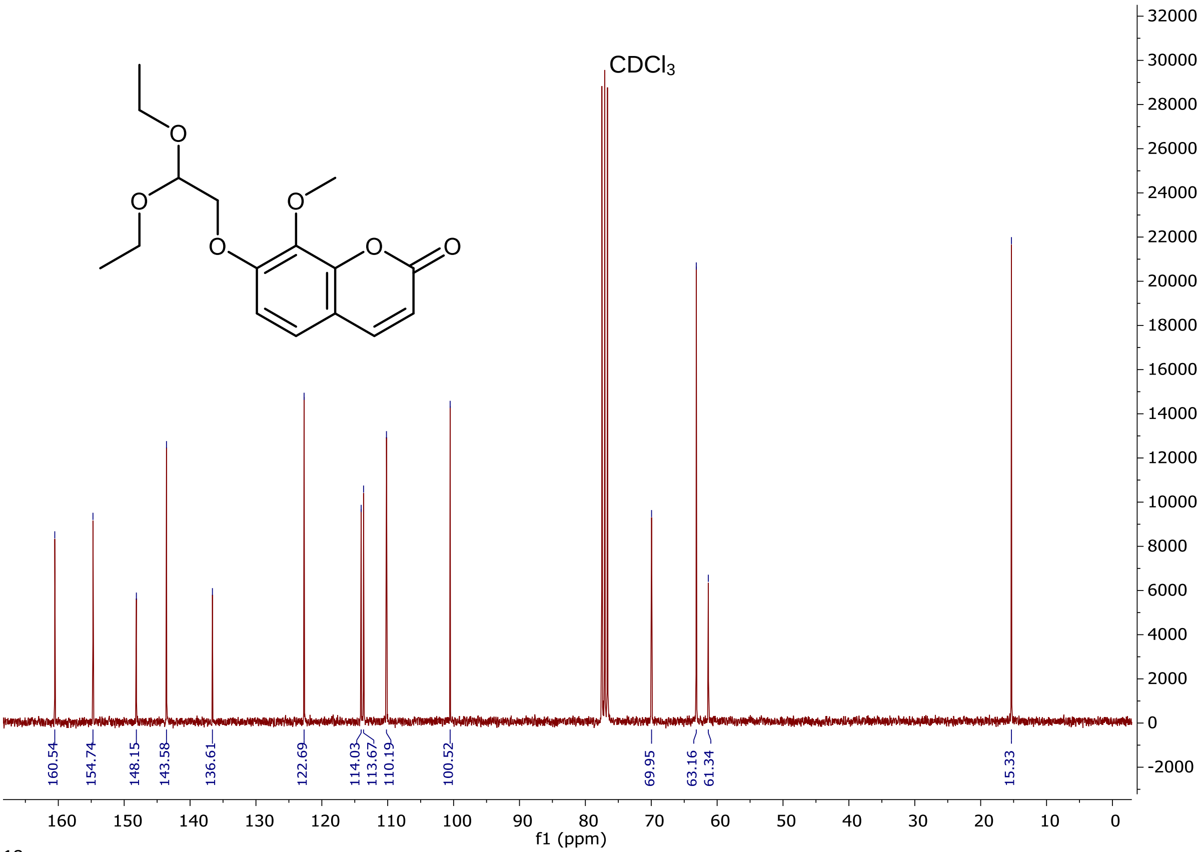

^{13}C NMR spectrum (75 MHz) of **4**; recorded in $CDCl_3$ at 298 K.

3.1.4. 2-((8-Methoxy-2-oxo-2*H*-chromen-7-yl)oxy)acetaldehyde (5)

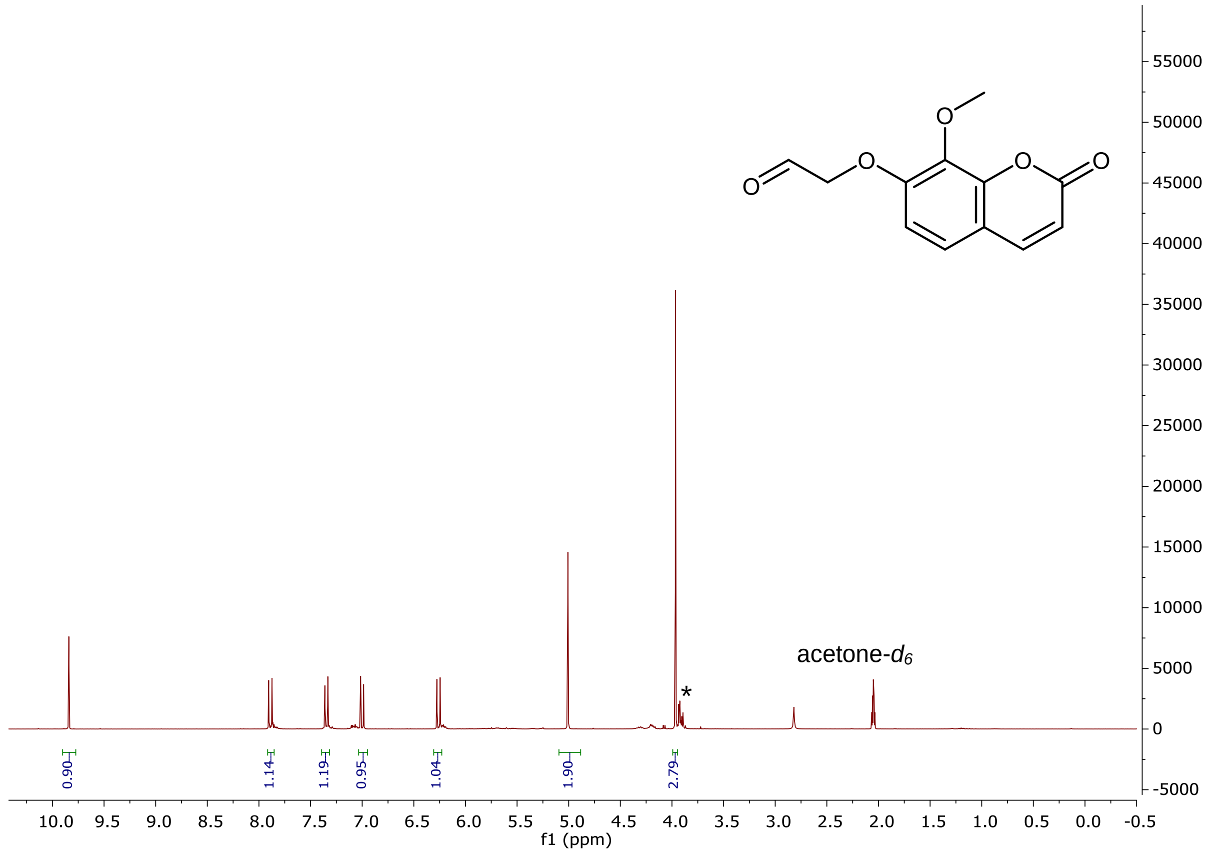

^{1}H NMR spectrum (300 MHz) of **5**; recorded in acetone-d_6 at 298 K. * Minor impurities

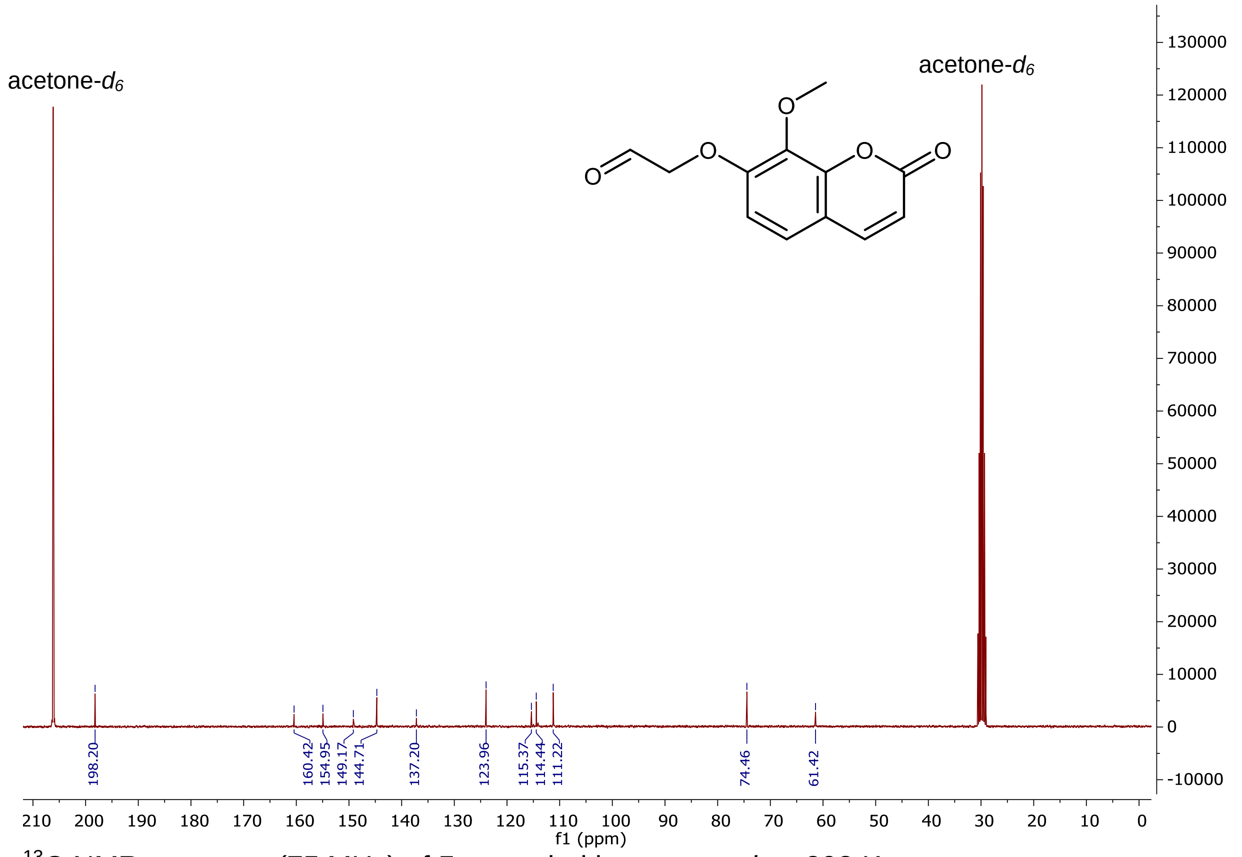

^{13}C NMR spectrum (75 MHz) of **5**; recorded in acetone-d_6 at 298 K.

3.1.5. 8-Methoxypsoralen (6)

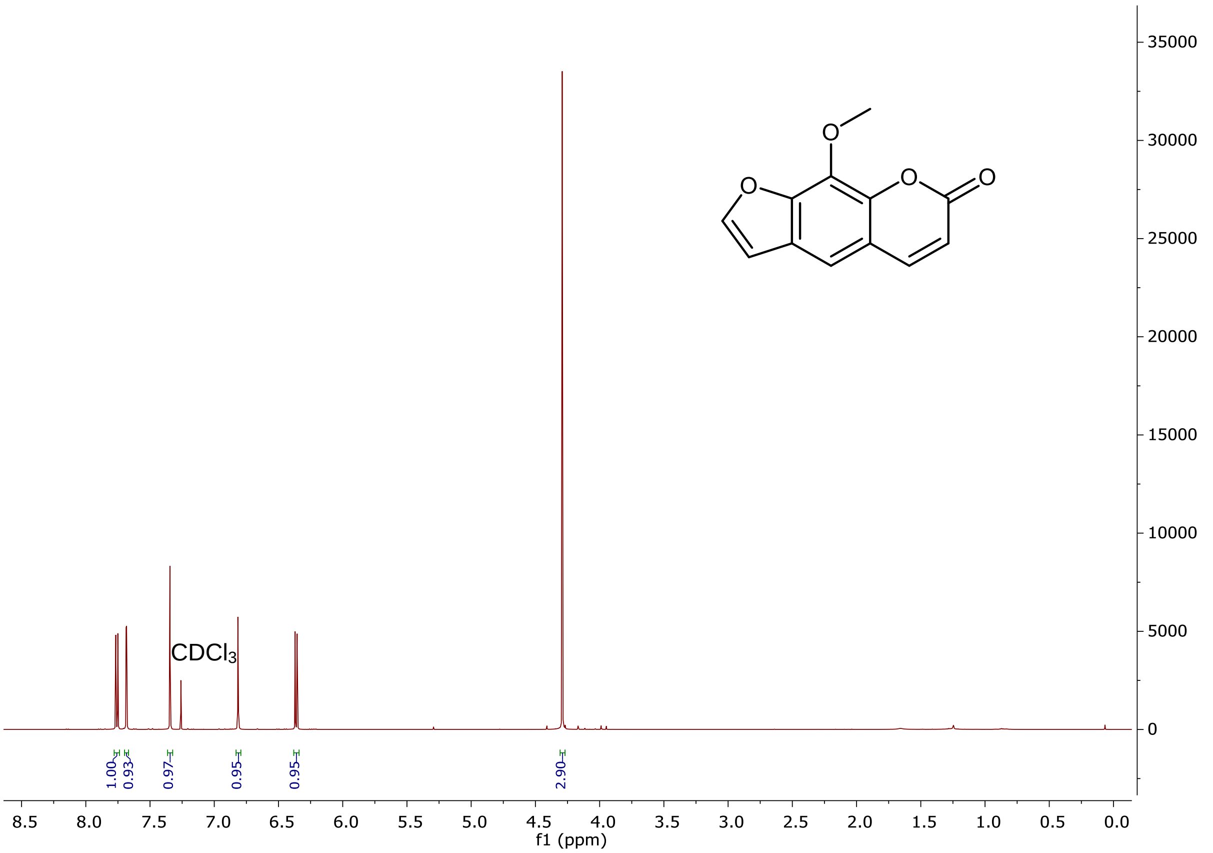

^{1}H NMR spectrum (600 MHz) of **6**; recorded $CDCl_3$ at 298 K.

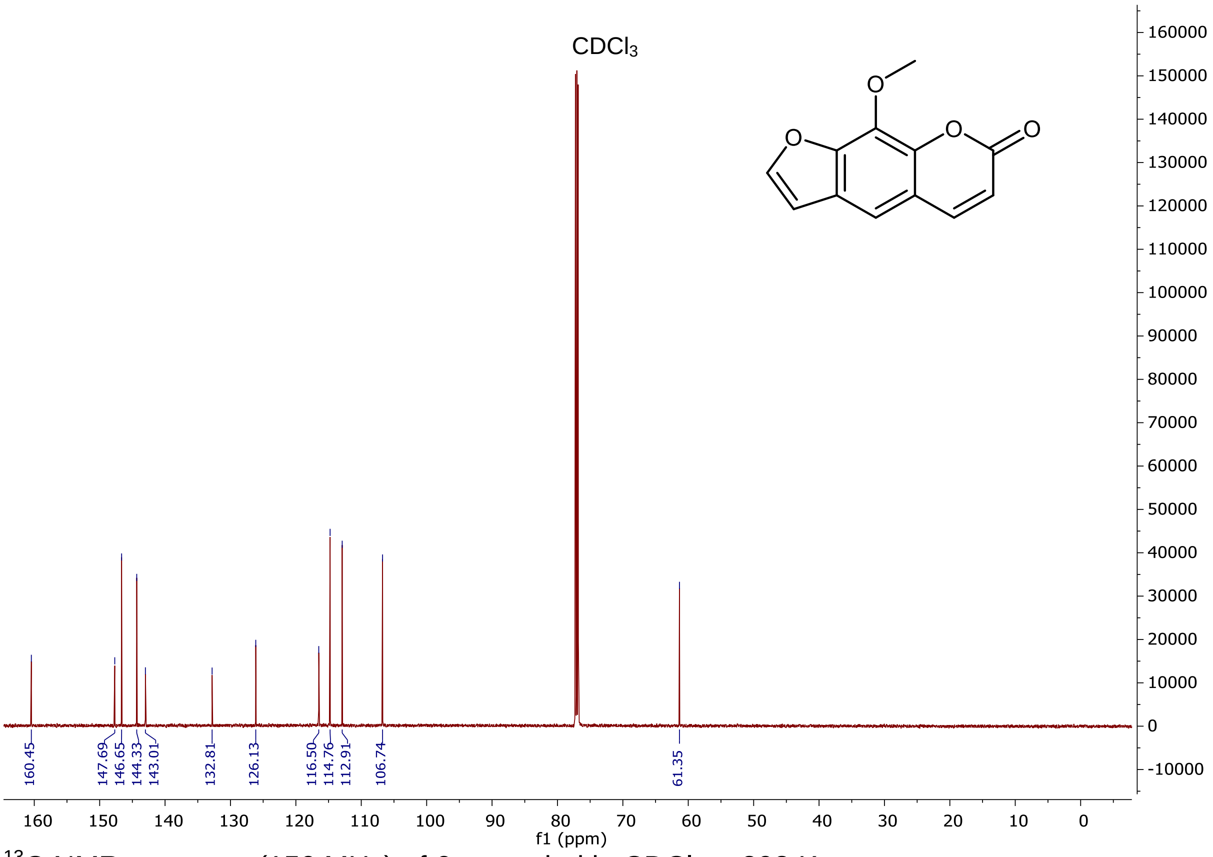

^{13}C NMR spectrum (150 MHz) of **6**; recorded in $CDCl_3$ at 298 K.

3.1.6. 5-Bromo-8-methoxypsoralen (7)

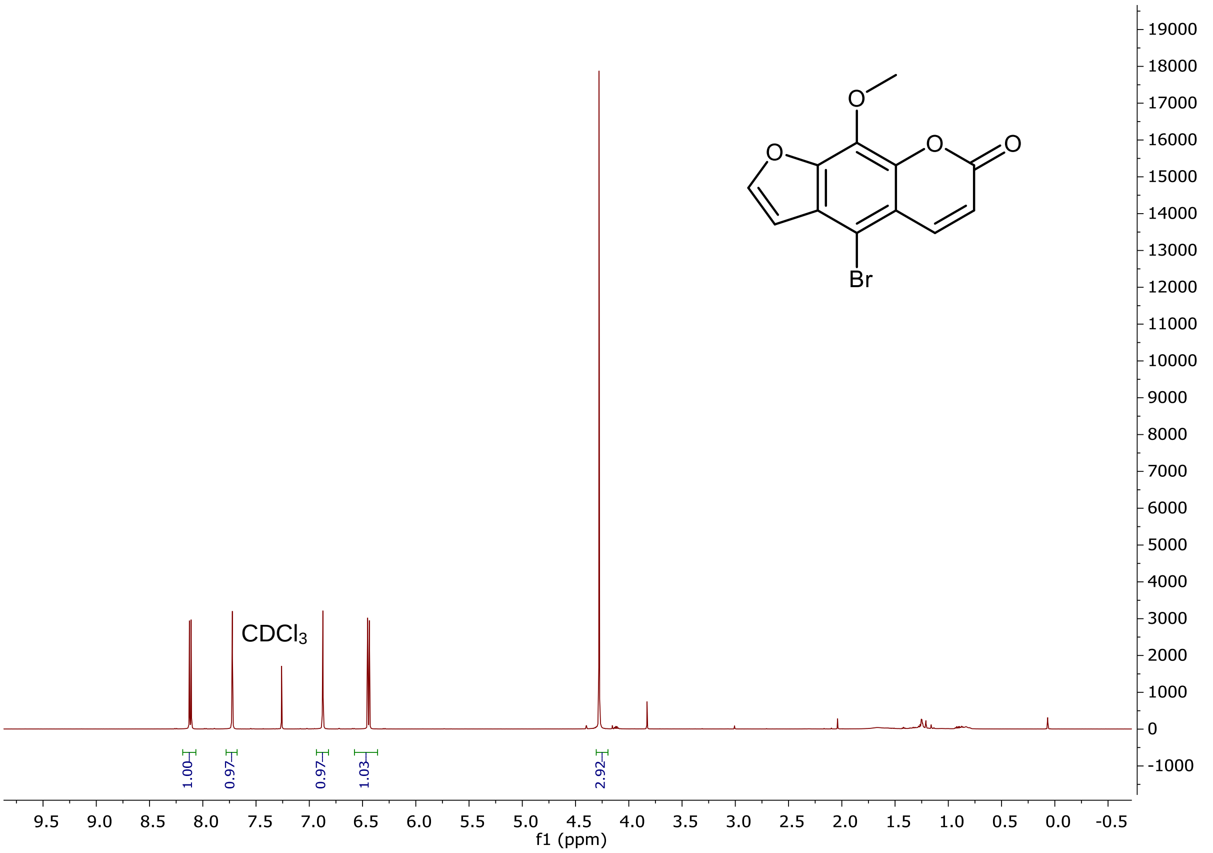

^{1}H NMR spectrum (600 MHz) of **7**; recorded $CDCl_3$ at 298 K.

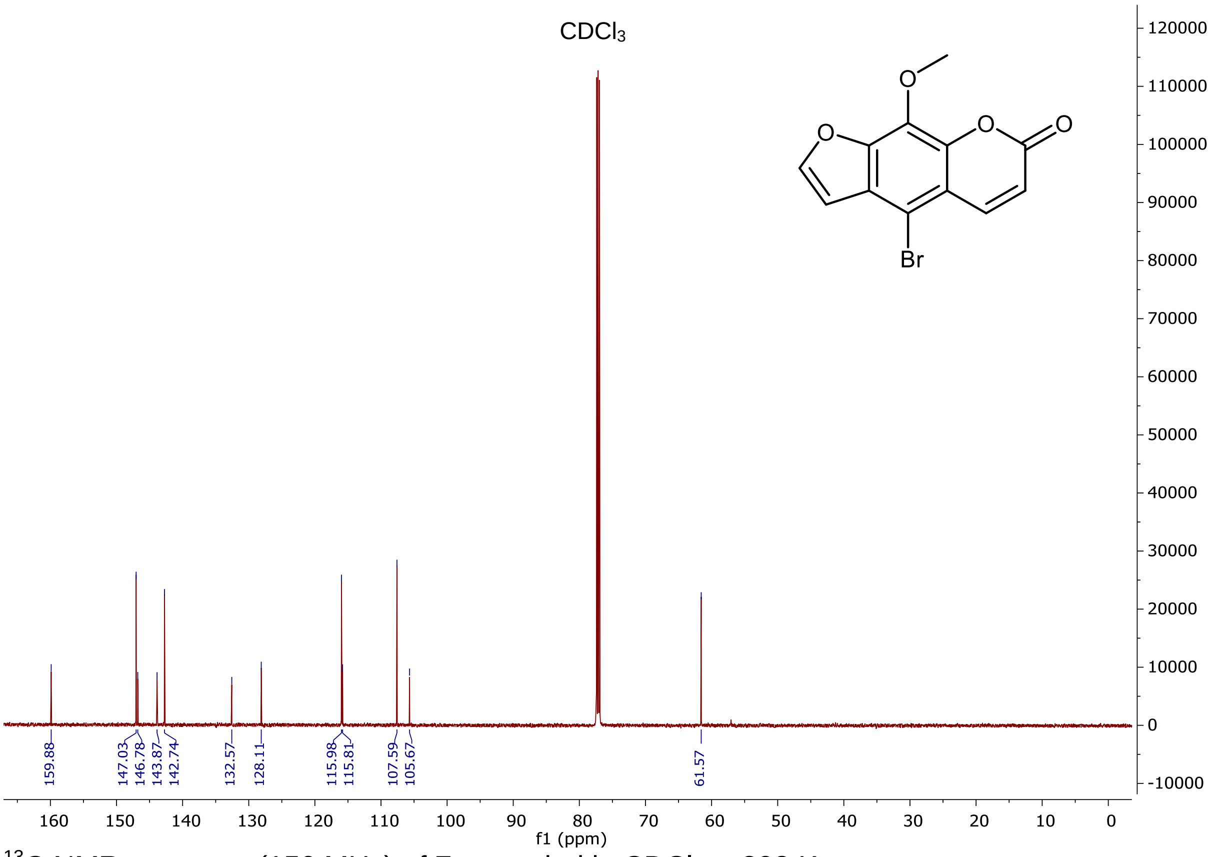

^{13}C NMR spectrum (150 MHz) of **7**; recorded in $CDCl_3$ at 298 K.

3.2. NMR spectra of 5-substituted 8-methoxypsoralens

3.2.1. 5-Cyano-8-methoxypsoralen (8)

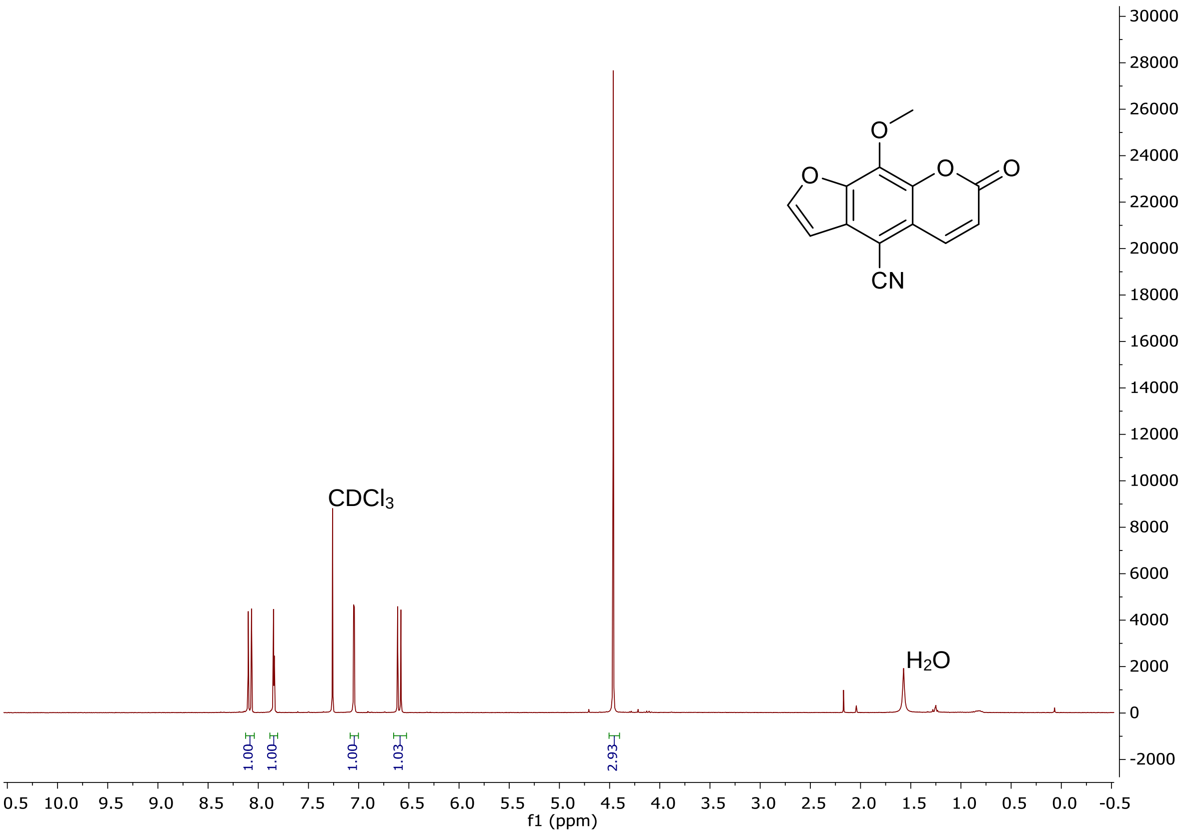

^{1}H NMR spectrum (300 MHz) of **8**; recorded $CDCl_3$ at 298 K.

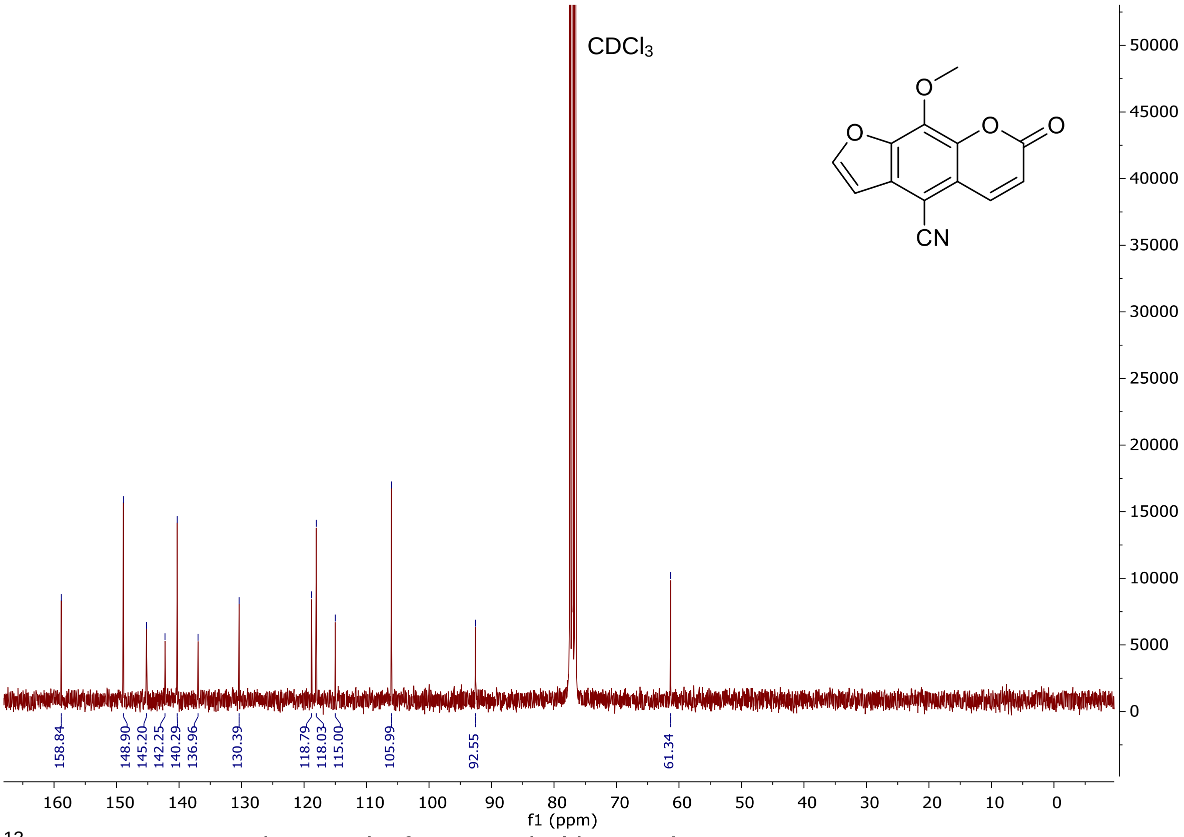

^{13}C NMR spectrum (75 MHz) of **8**; recorded in $CDCl_3$ at 298 K.

3.2.2. 5-Nitro-8-methoxypsoralen (9)

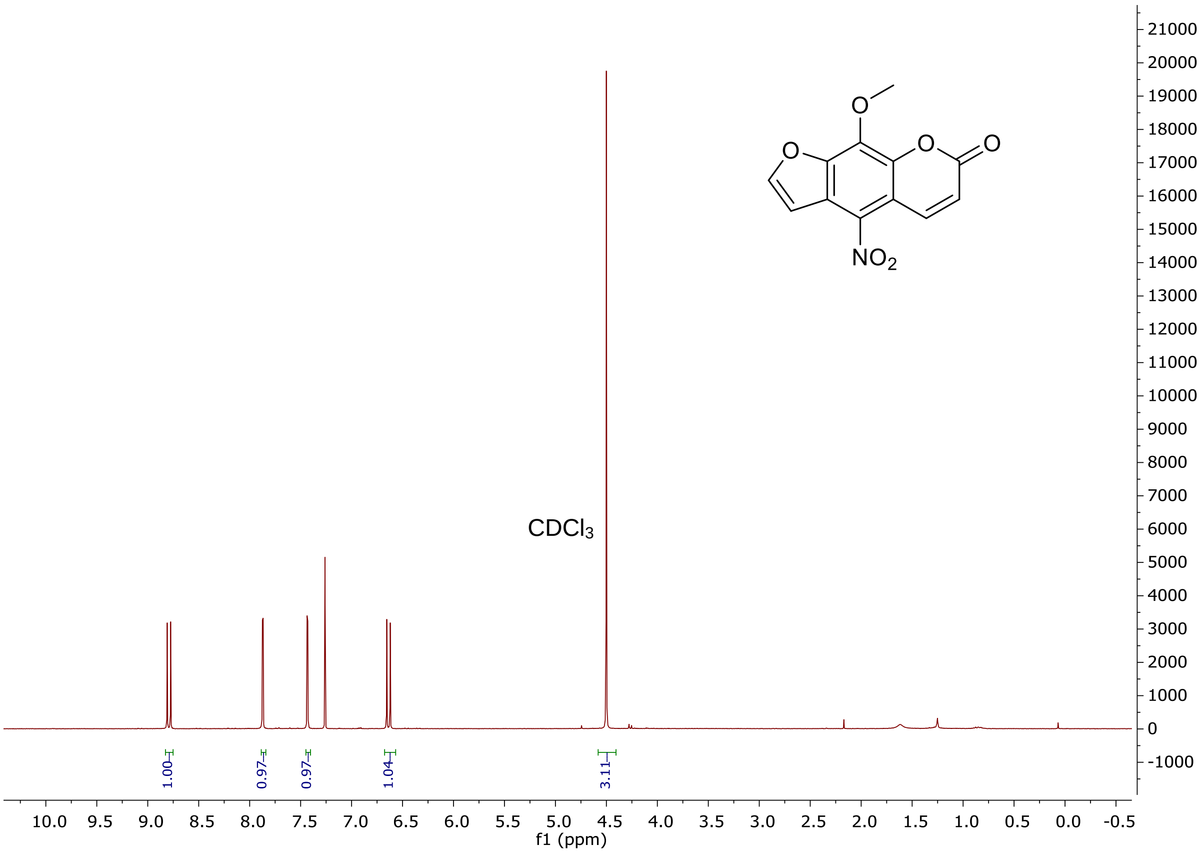

^{1}H NMR spectrum (300 MHz) of **9**; recorded $CDCl_3$ at 298 K.

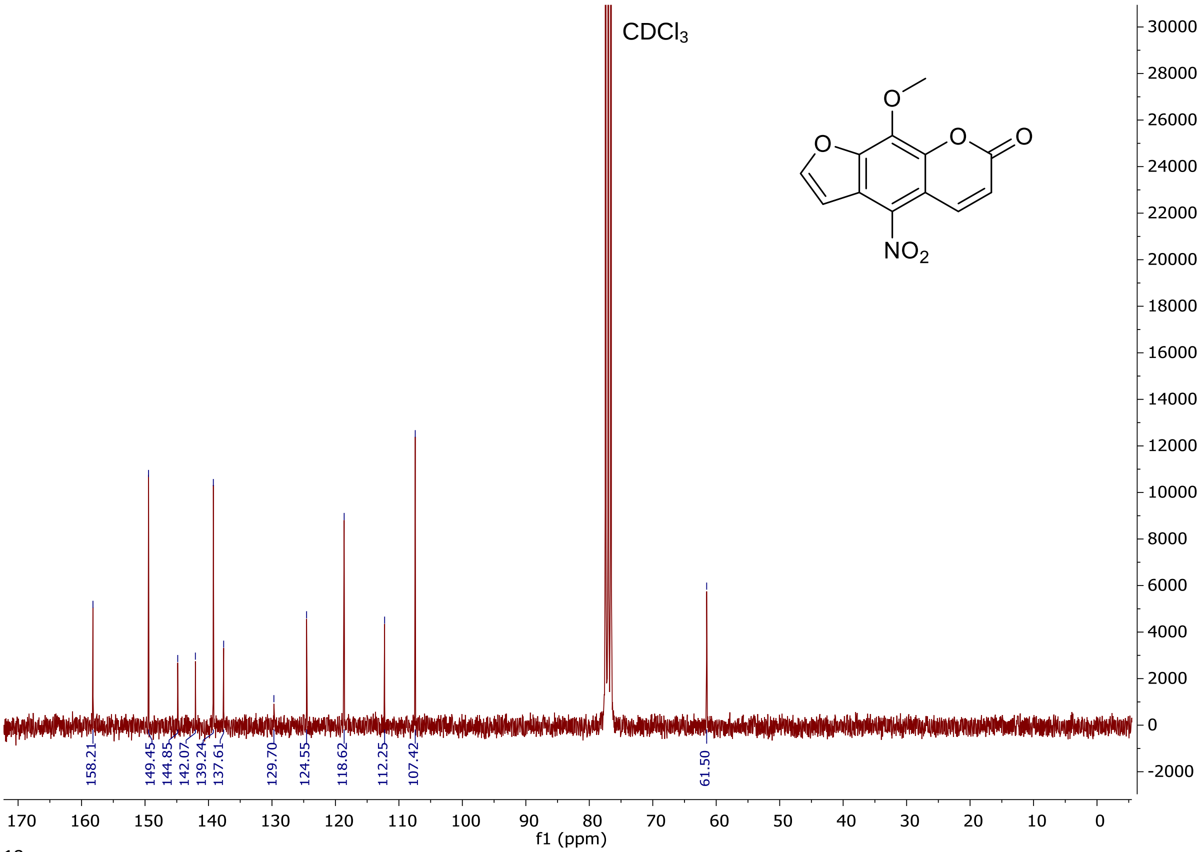

^{13}C NMR spectrum (75 MHz) of **9**; recorded in $CDCl_3$ at 298 K.

3.2.3. 4-(9-Methoxy-7-oxo-7*H*-furo[3,2-*g*]chromen-4-yl)benzonitrile (11a)

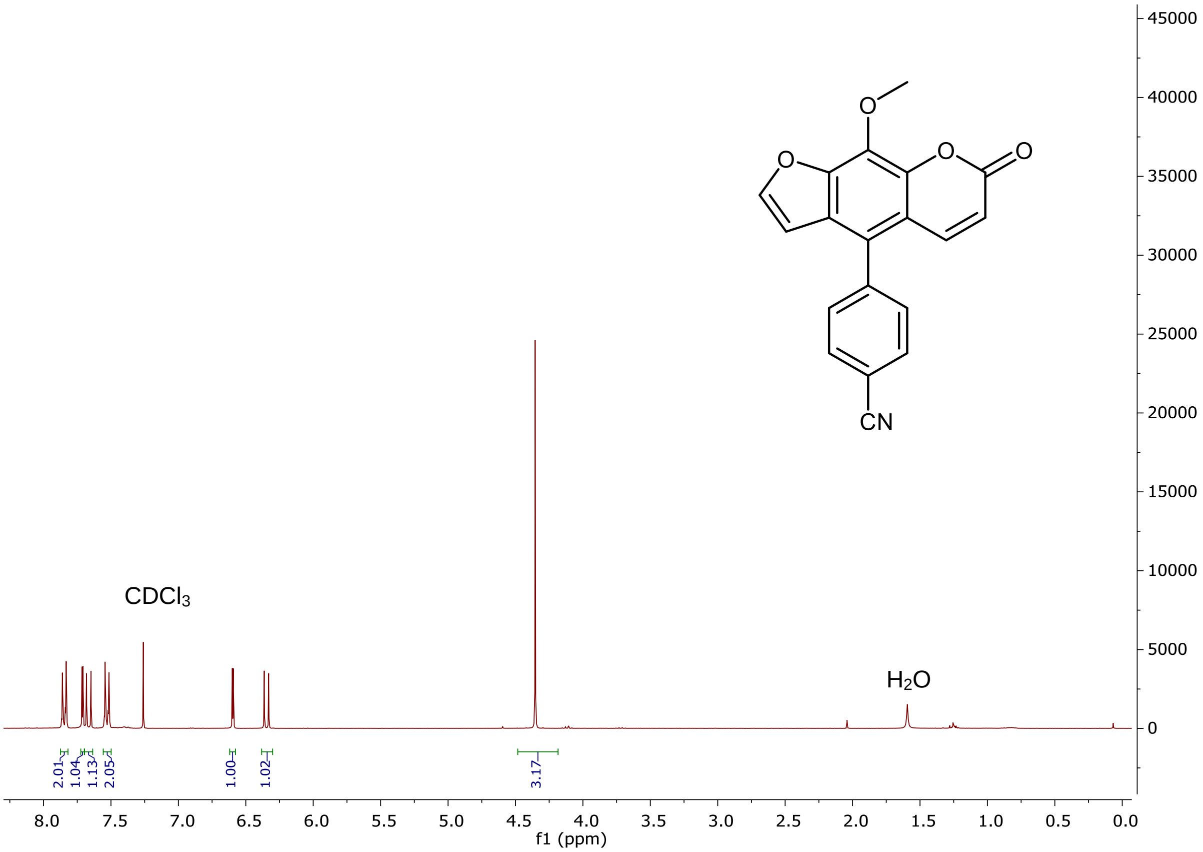

^{1}H NMR spectrum (300 MHz) of **11a**; recorded $CDCl_3$ at 298 K.

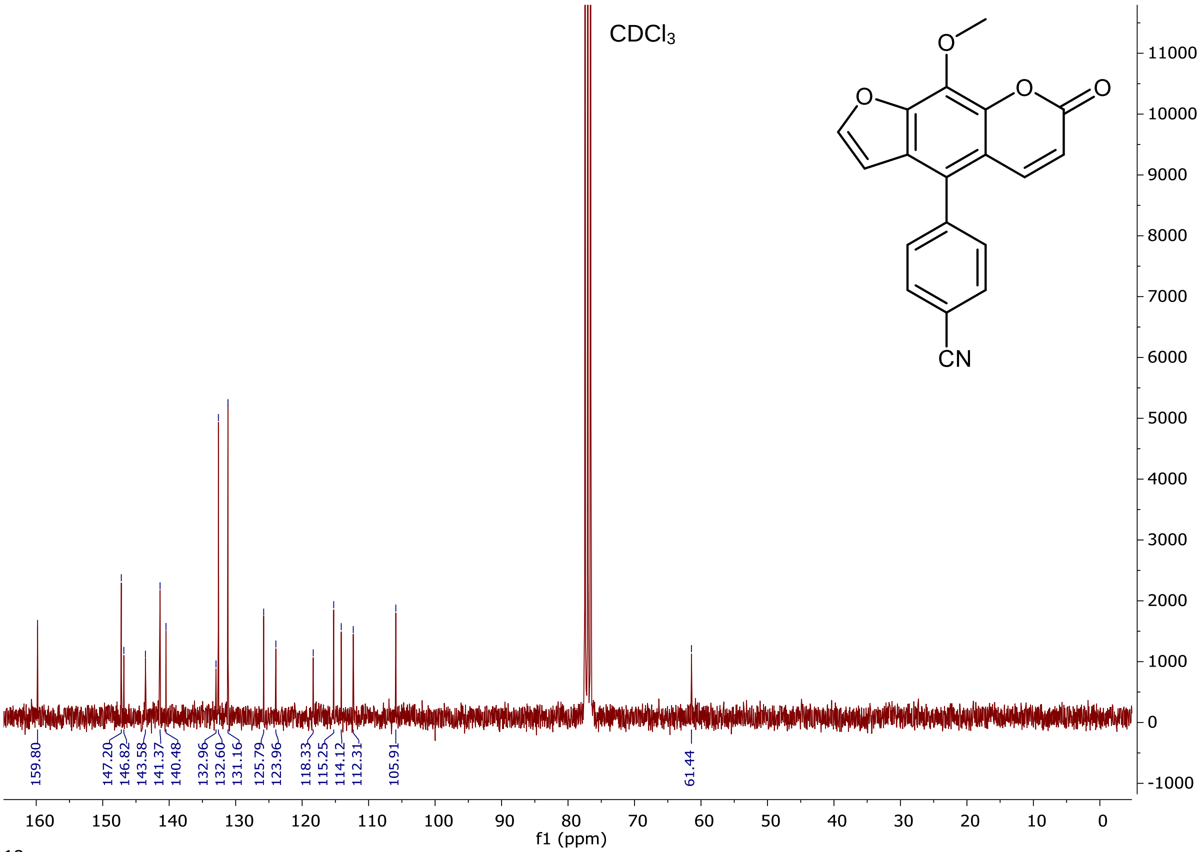

^{13}C NMR spectrum (75 MHz) of **11a**; recorded in $CDCl_3$ at 298 K.

3.2.4. 9-Methoxy-4-(4-nitrophenyl)-7*H*-furo[3,2-*g*]chromen-7-one (11b)

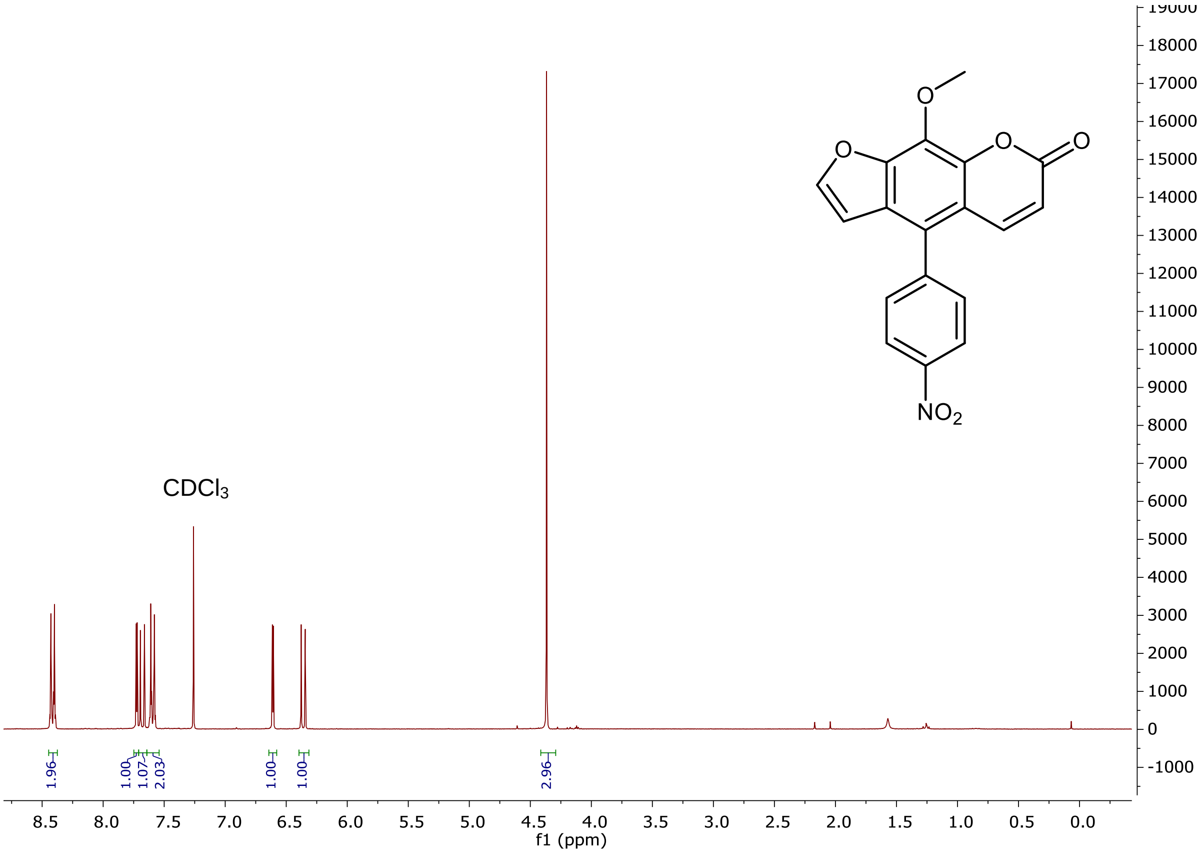

^{1}H NMR spectrum (300 MHz) of **11b**; recorded CDCl3 at 298 K.

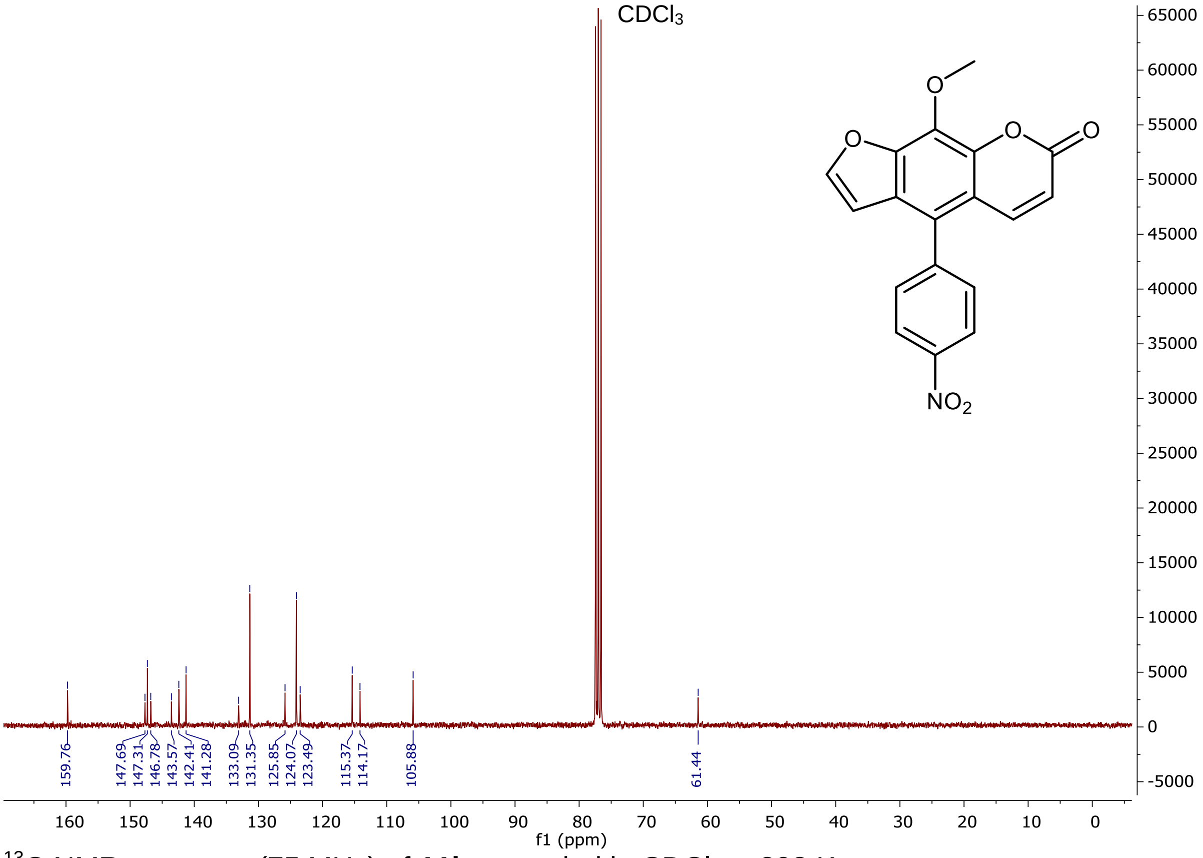

^{13}C NMR spectrum (75 MHz) of **11b**; recorded in CDCl3 at 298 K.

3.2.5. 4-(9-Methoxy-7-oxo-7*H*-furo[3,2-*g*]chromen-4-yl)benzaldehyde (11c)

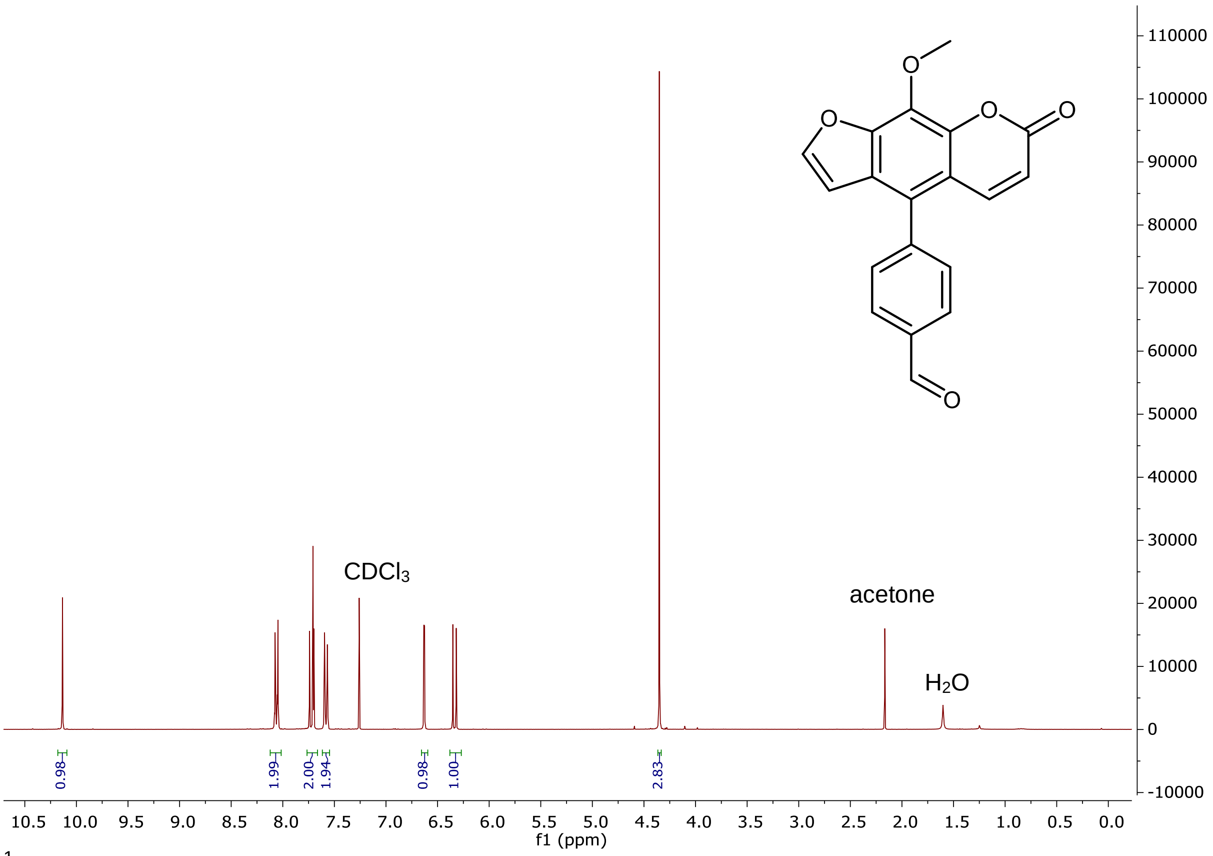

^{1}H NMR spectrum (300 MHz) of **11c**; recorded $CDCl_3$ at 298 K.

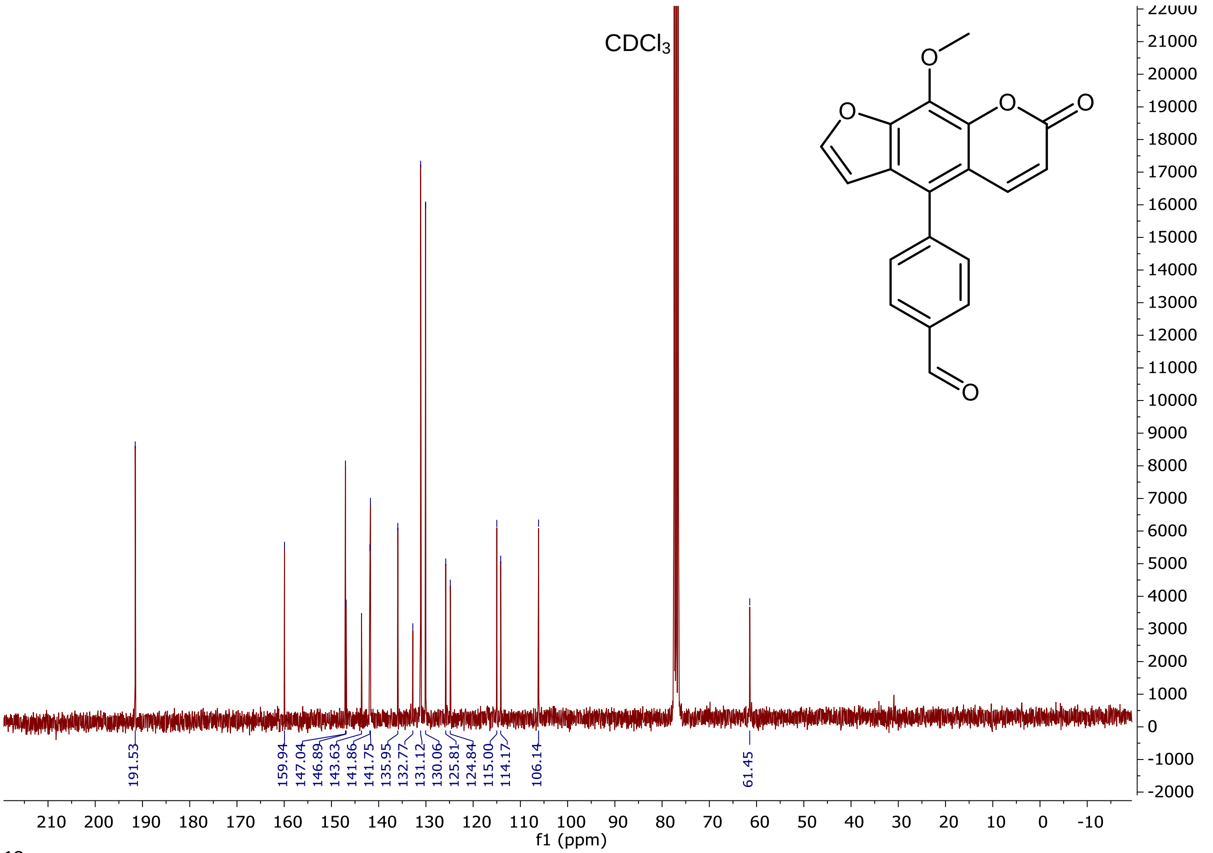

^{13}C NMR spectrum (75 MHz) of **11c**; recorded in $CDCl_3$ at 298 K.

3.2.6. 9-Methoxy-4-(pyridin-4-yl)-7*H*-furo[3,2-*g*]chromen-7-one (11d)

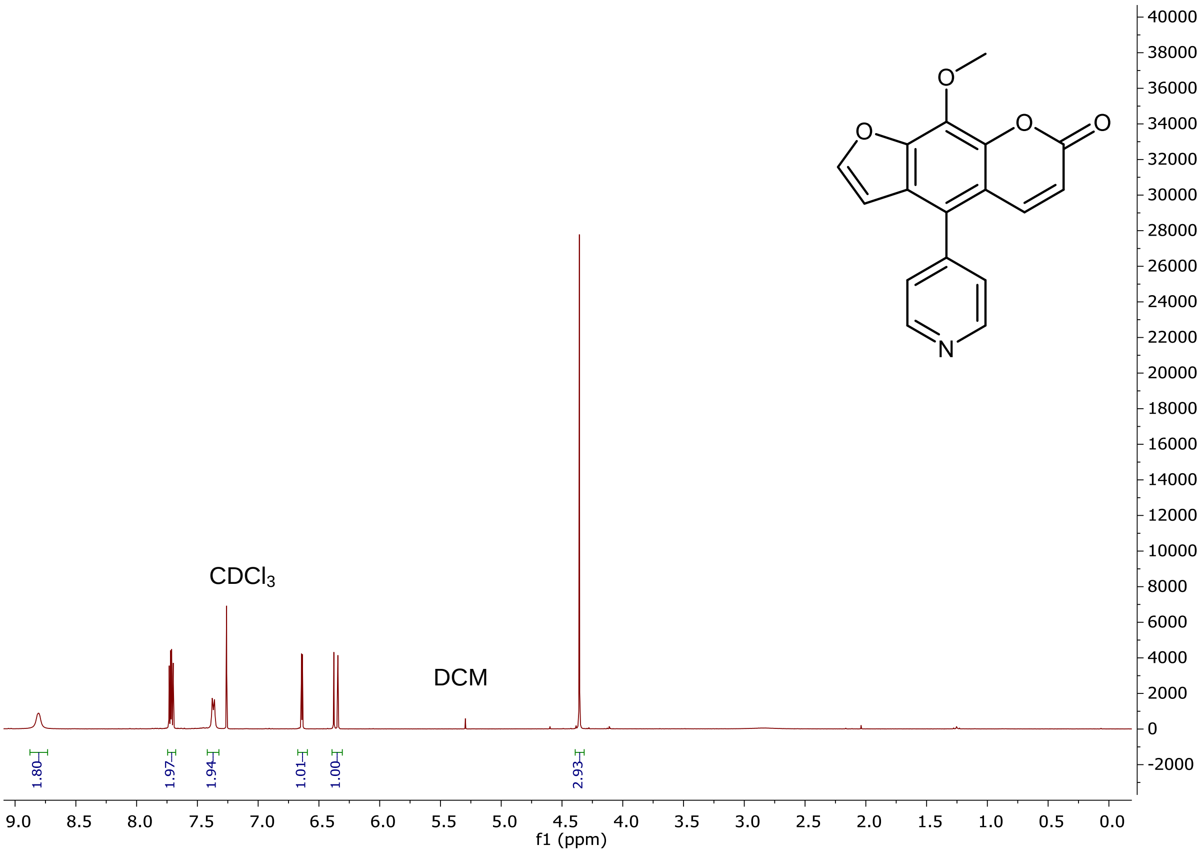

^{1}H NMR spectrum (300 MHz) of **11d**; recorded $CDCl_3$ at 298 K.

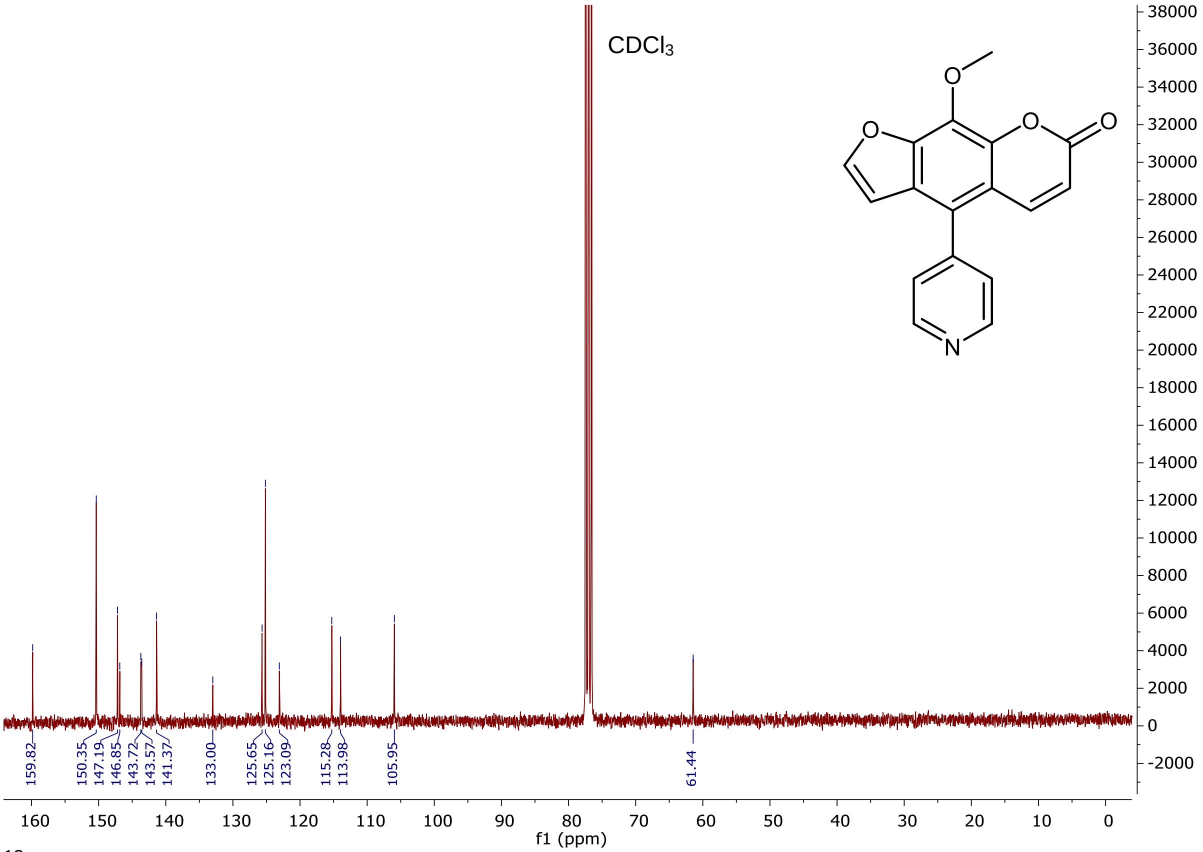

^{13}C NMR spectrum (75 MHz) of **11d**; recorded in $CDCl_3$ at 298 K.

3.2.7. 4-(4-(Dimethylamino)phenyl)-9-methoxy-7*H*-furo[3,2-*g*]chromen-7-one (11e)

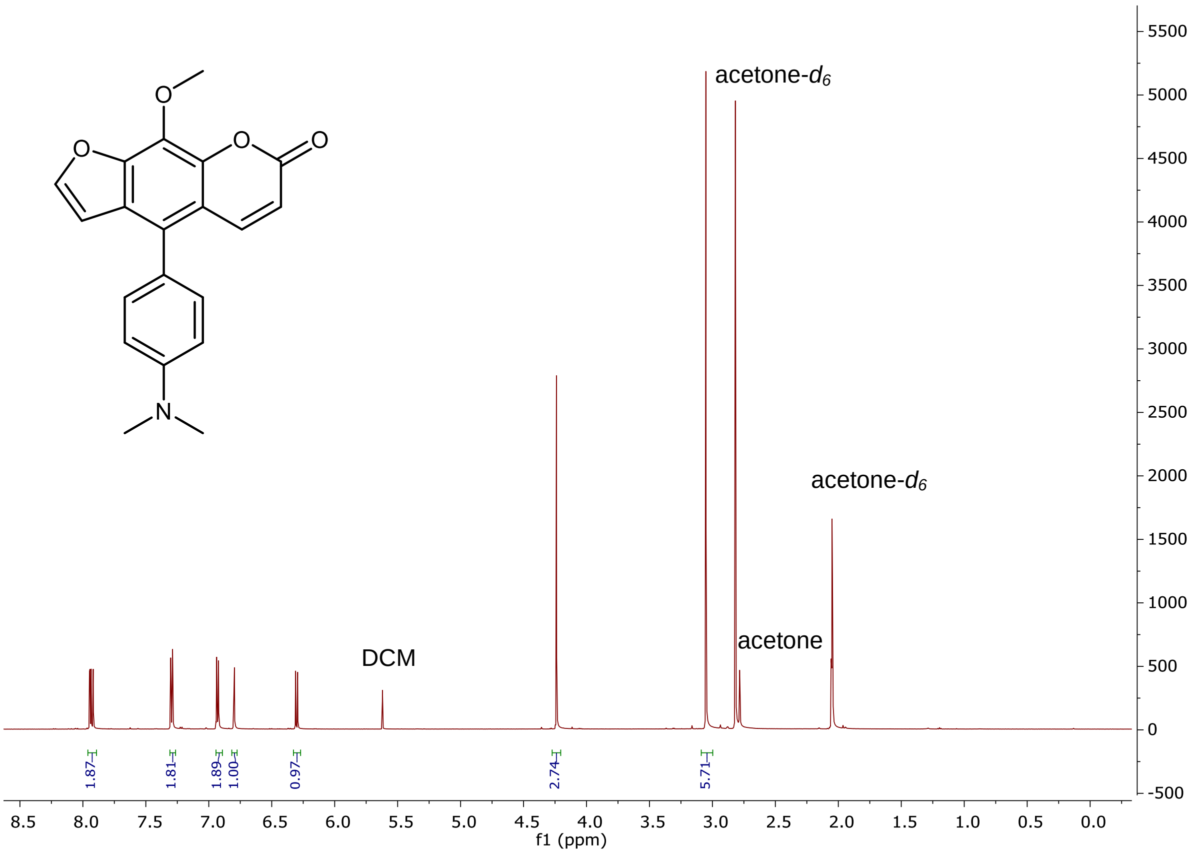

^{1}H NMR spectrum (600 MHz) of **11e**; recorded acetone-d_6 at 298 K.

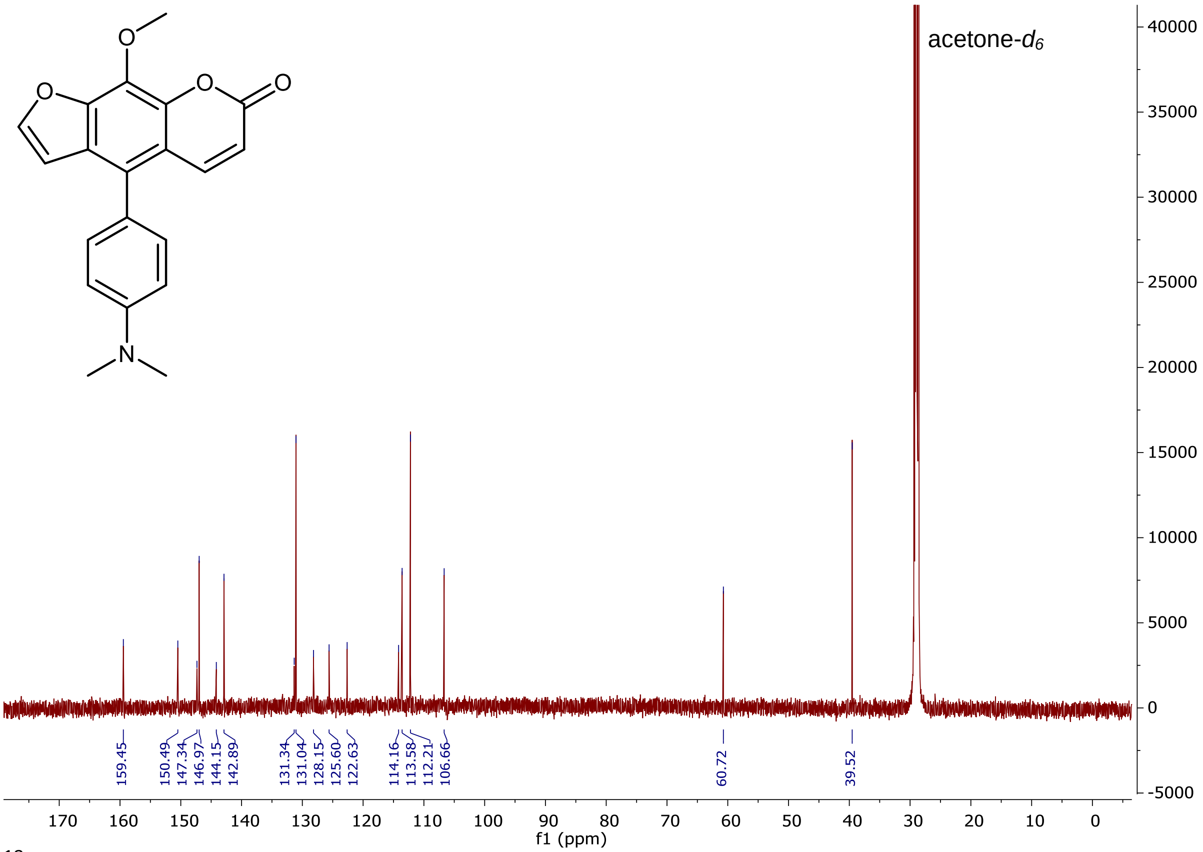

^{13}C NMR spectrum (150 MHz) of **11e**; recorded in acetone-d_6 at 298 K.

3.2.8 4-(4-((Dimethylamino)methyl)phenyl)-9-methoxy-7*H*-furo[3,2-*g*]chromen-7-one (11f)

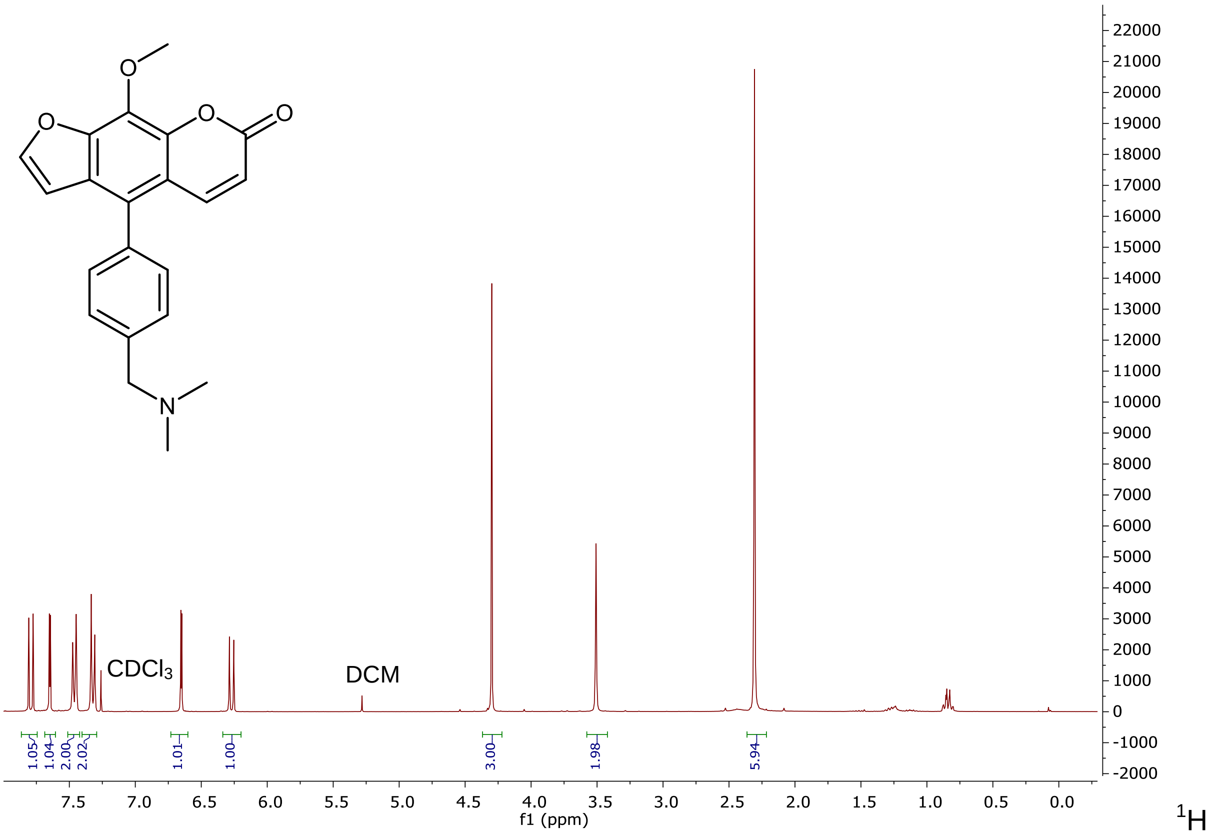

NMR spectrum (600 MHz) of **11f**; recorded CDCl3 at 298 K.

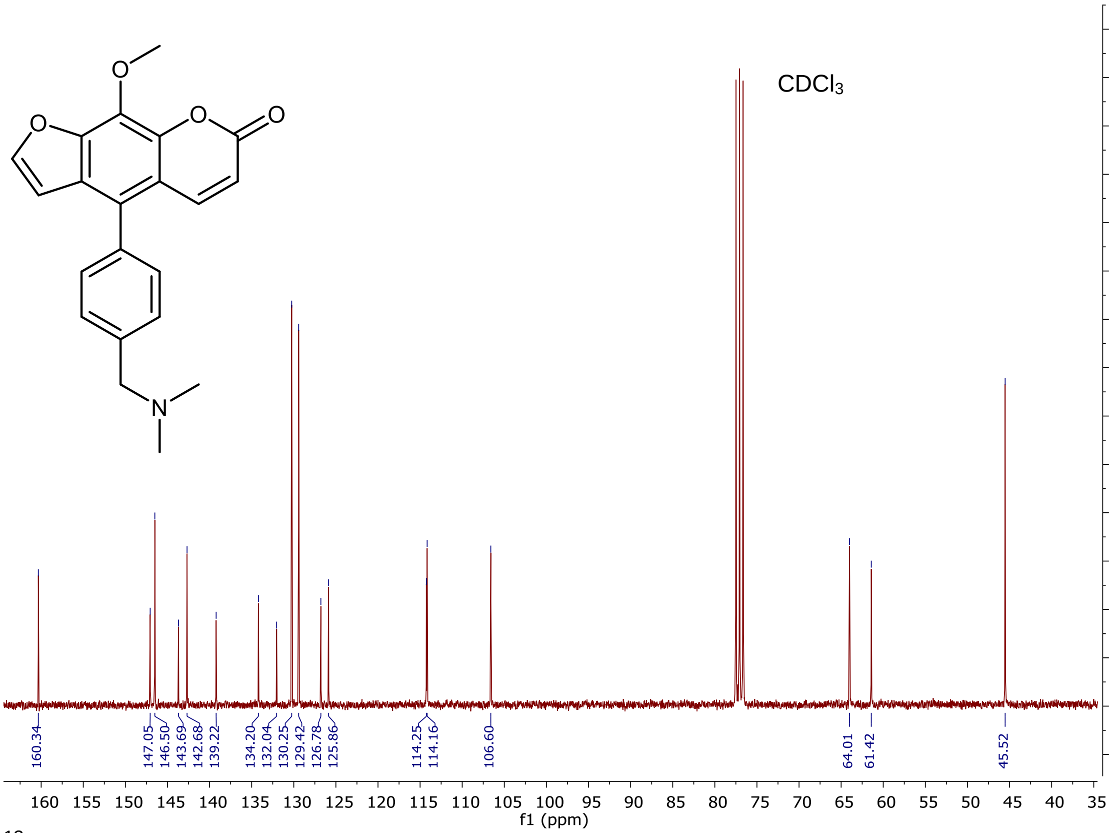

^{13}C NMR spectrum (150 MHz) of **11f**; recorded CDCl3 at 298 K.

3.2.9. 4-(9-Methoxy-7-oxo-7H-furo[3,2-*g*]chromen-4-yl)benzoic acid (11g)

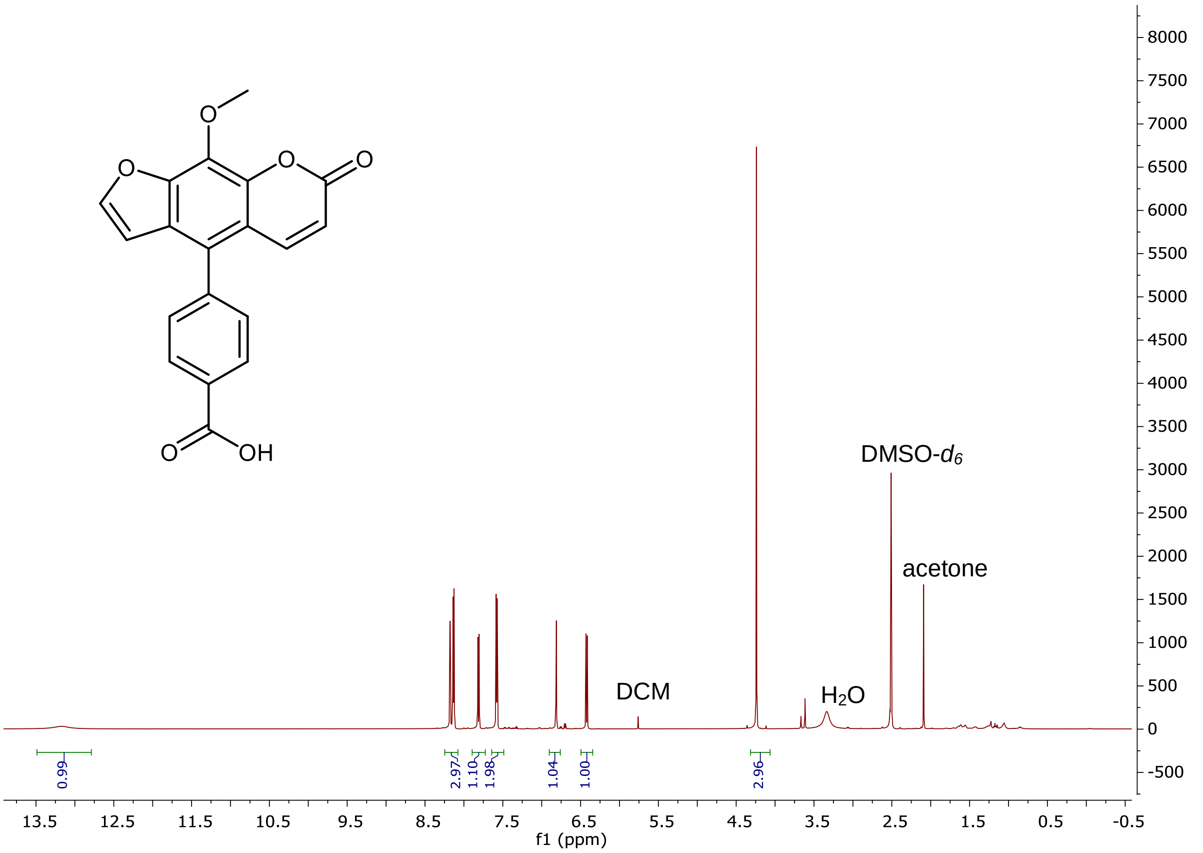

^{1}H NMR spectrum (600 MHz) of **11g**; recorded DMSO-d_6 at 298 K.

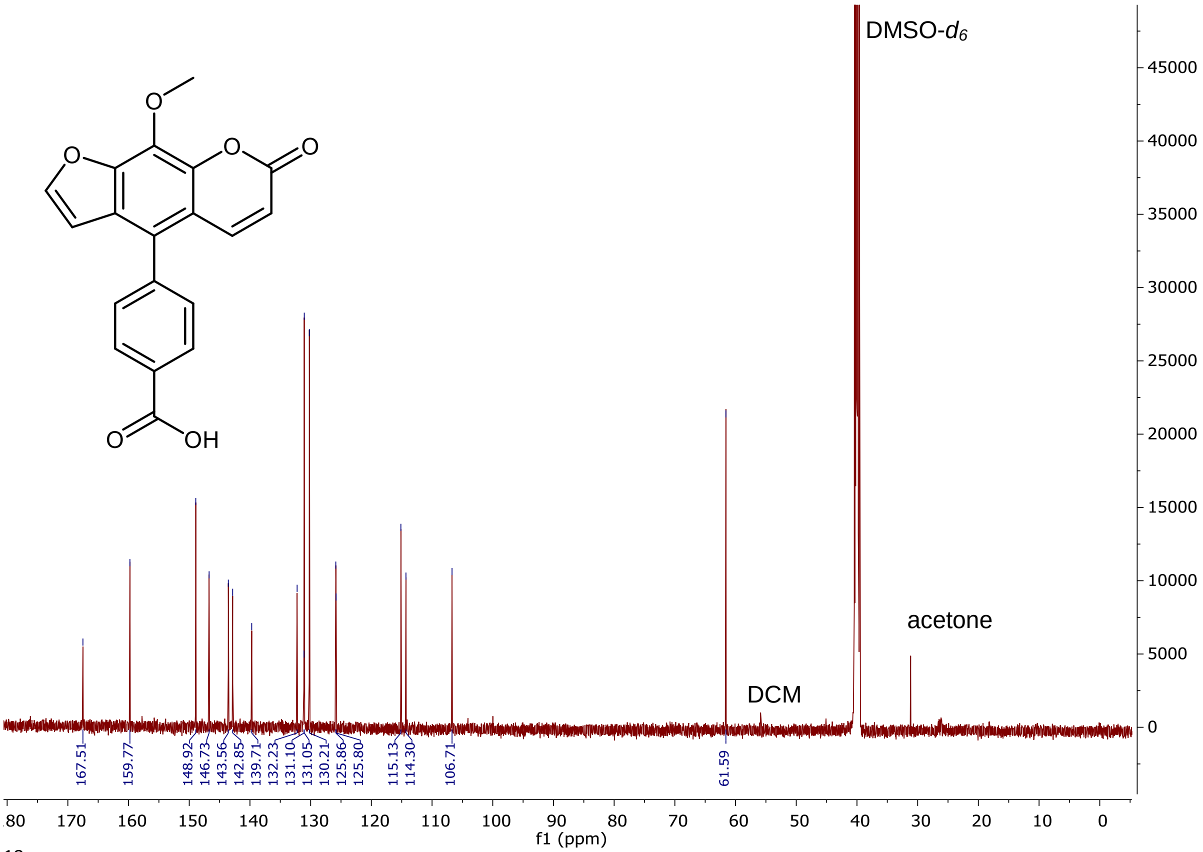

^{13}C NMR spectrum (150 MHz) of **11g**; recorded in DMSO-d_6 at 298 K.

3.2.10. 4-((9-Methoxy-7-oxo-7*H*-furo[3,2-*g*]chromen-4-yl)ethynyl)benzonitrile (13a)

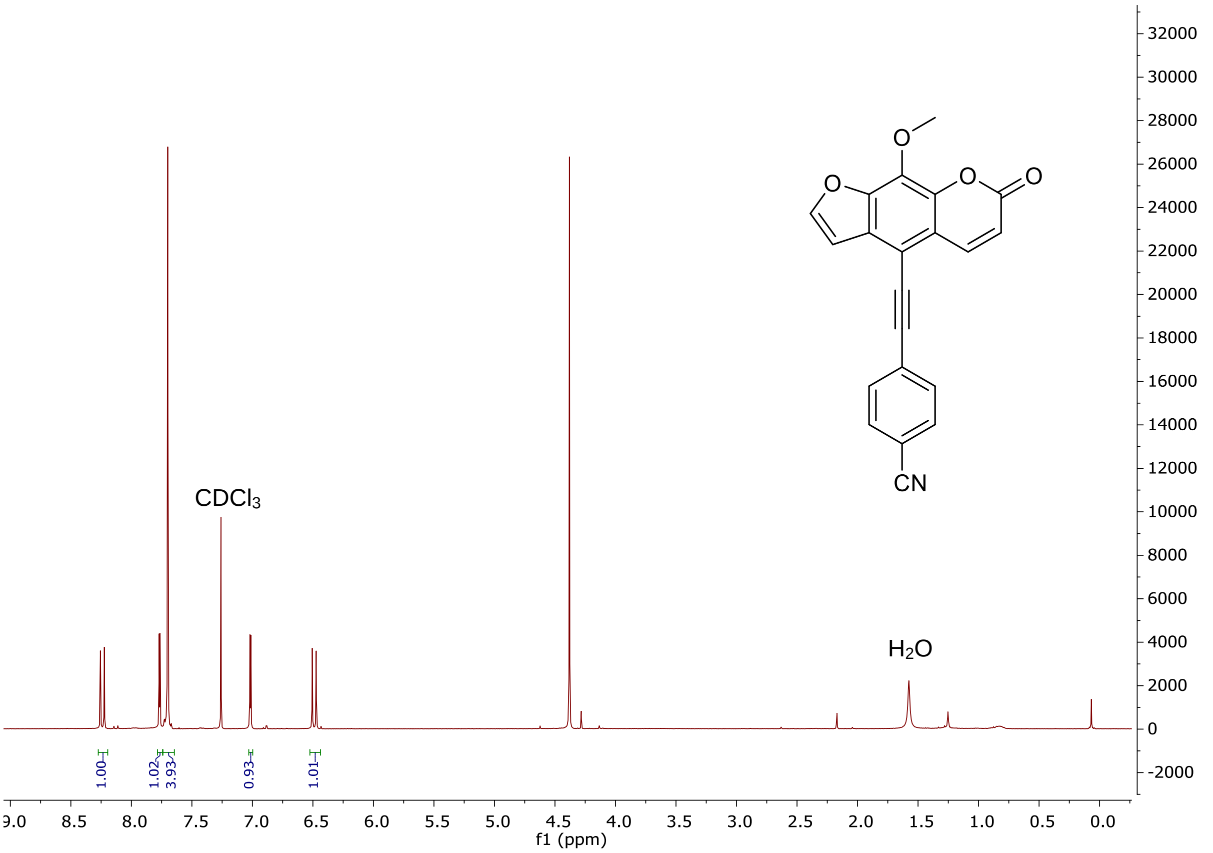

^{1}H NMR spectrum (300 MHz) of **13a**; recorded CDCl3 at 298 K.

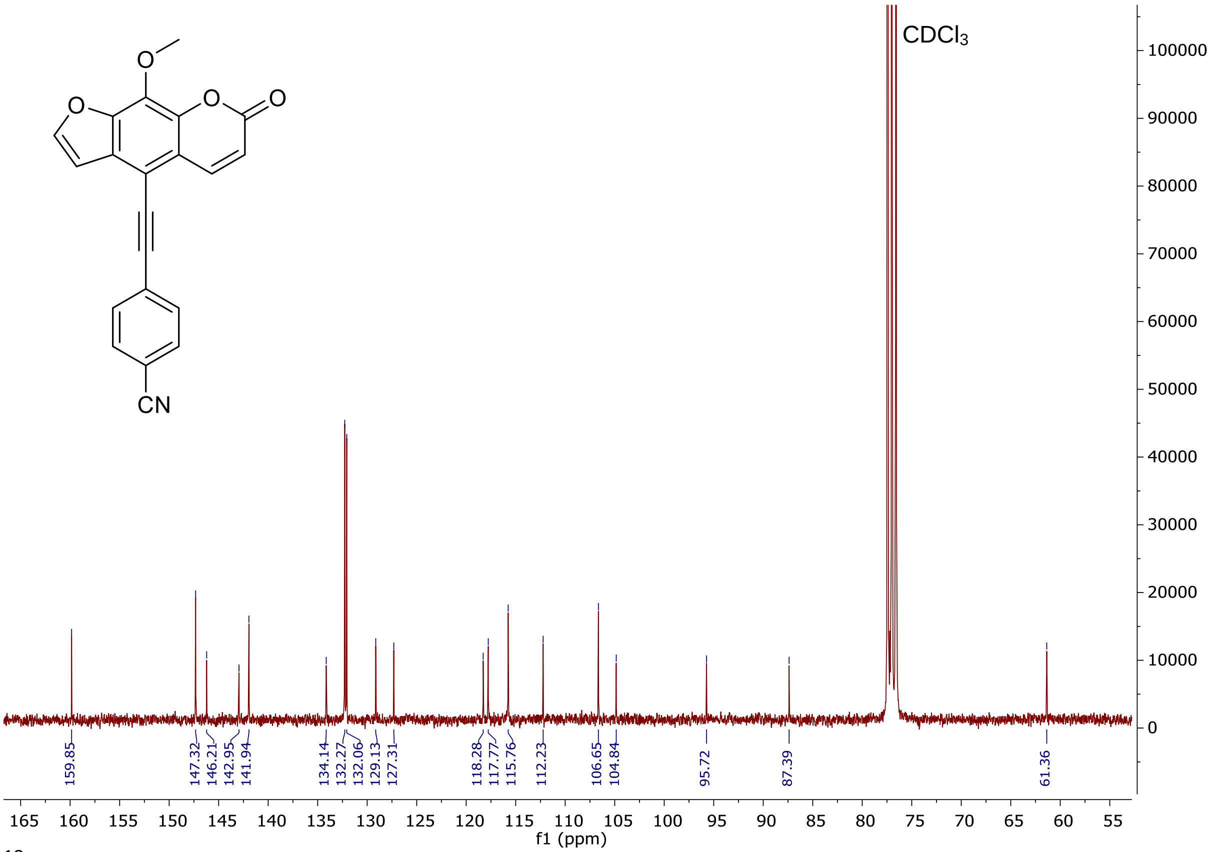

^{13}C NMR spectrum (75 MHz) of **13a**; recorded CDCl3 at 298 K.

3.2.11. 9-Methoxy-4-((4-nitrophenyl)ethynyl)-7*H*-furo[3,2-*g*]chromen-7-one (13b)

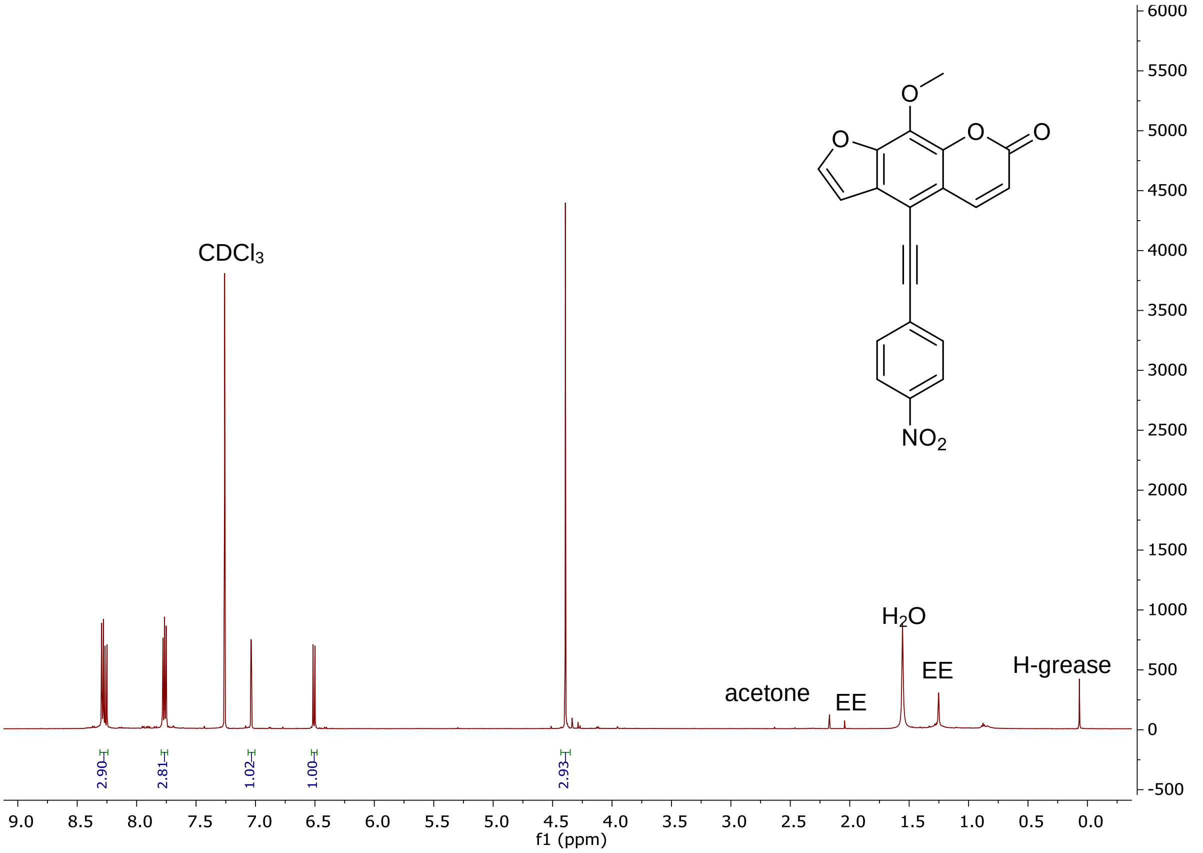

^{1}H NMR spectrum (600 MHz) of **13b**; recorded CDCl3 at 298 K.

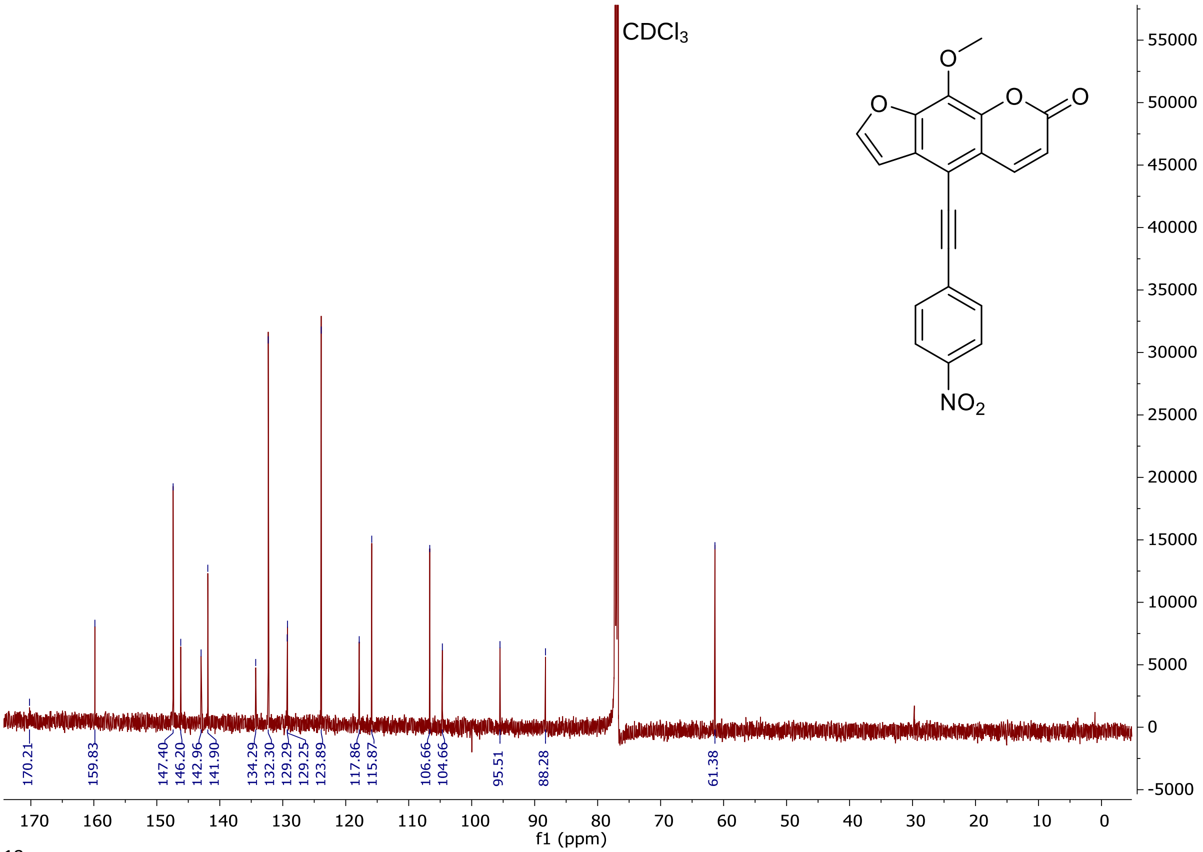

^{13}C NMR spectrum (150 MHz) of **13b**; recorded CDCl3 at 298 K.

3.2.12. 4-((9-Methoxy-7-oxo-7*H*-furo[3,2-*g*]chromen-4-yl)ethynyl)benzaldehyde (13c)

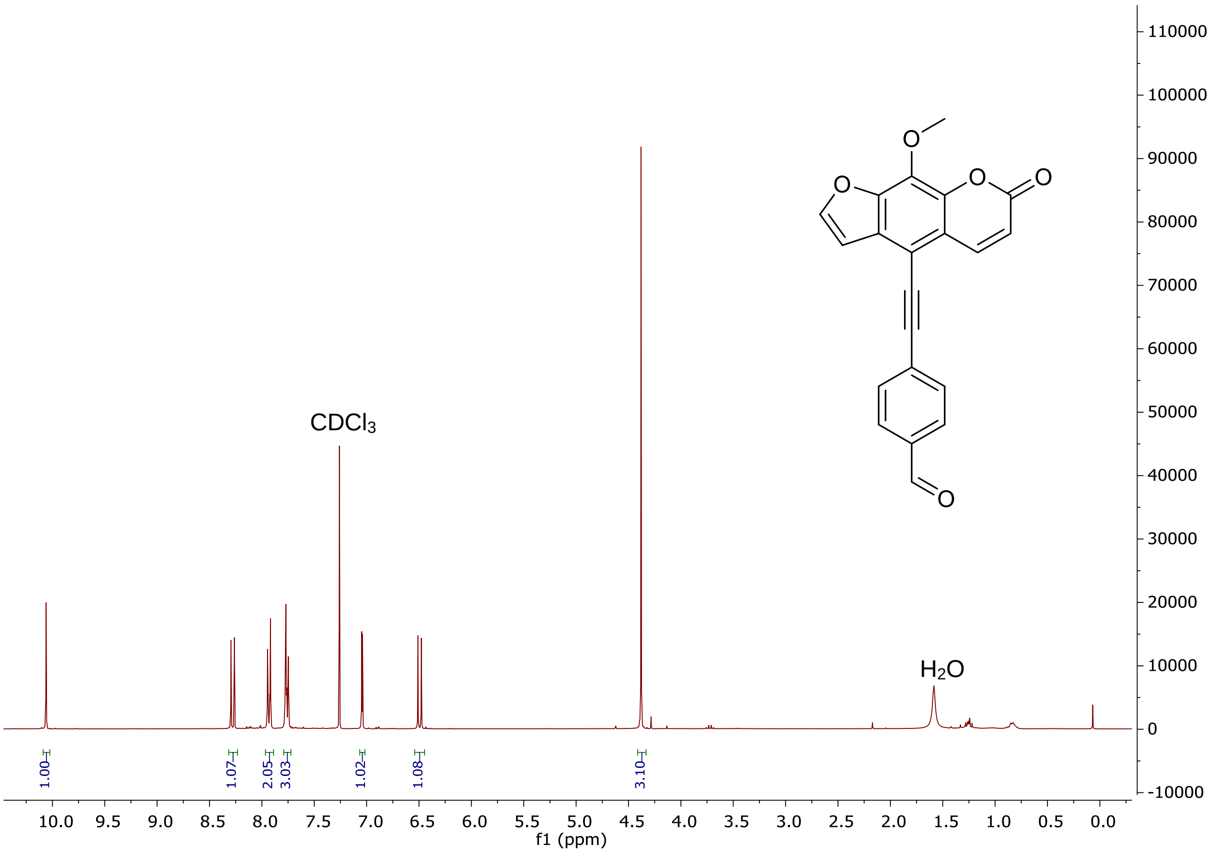

^{1}H NMR spectrum (300 MHz) of **13c**; recorded $CDCl_3$ at 298 K.

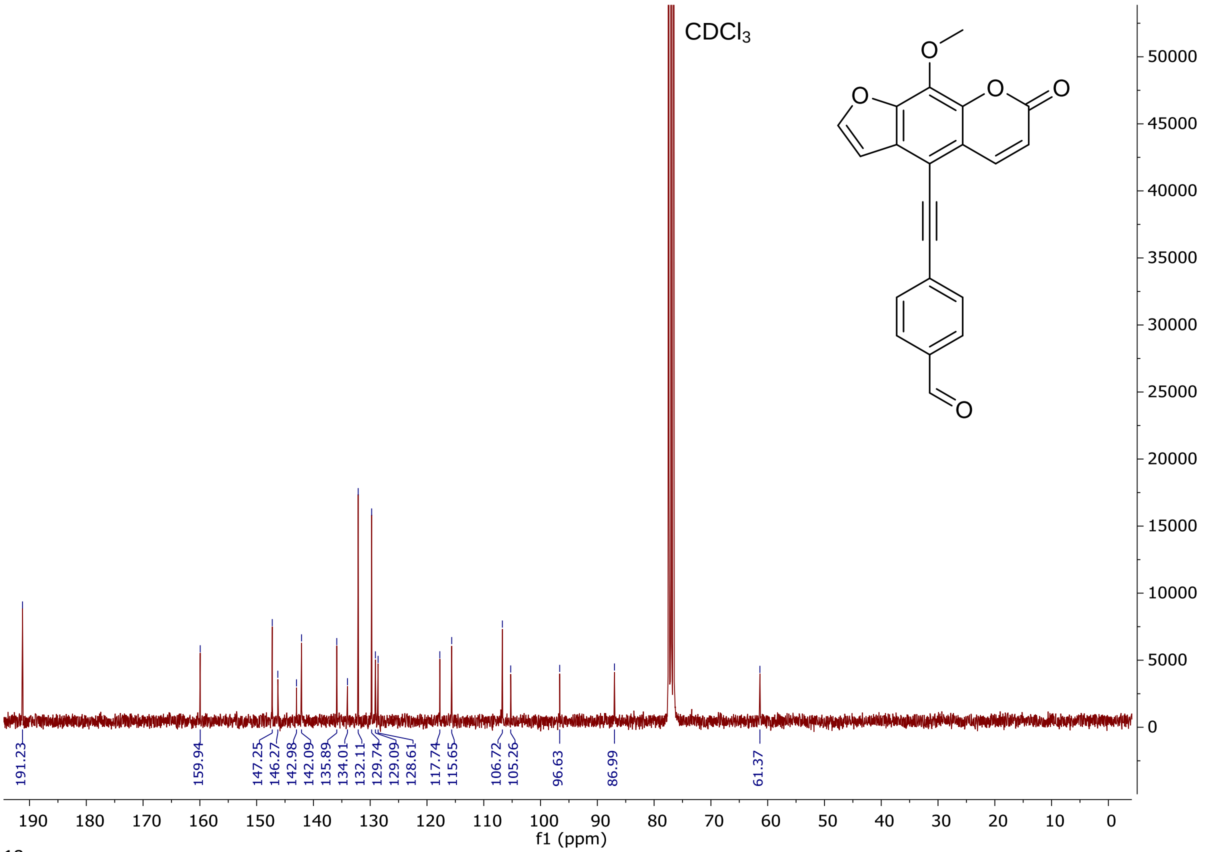

^{13}C NMR spectrum (75 MHz) of **13c**; recorded $CDCl_3$ at 298 K.

3.2.13. 9-Methoxy-4-(pyridin-4-ylethynyl)-7*H*-furo[3,2-*g*]chromen-7-one (13d)

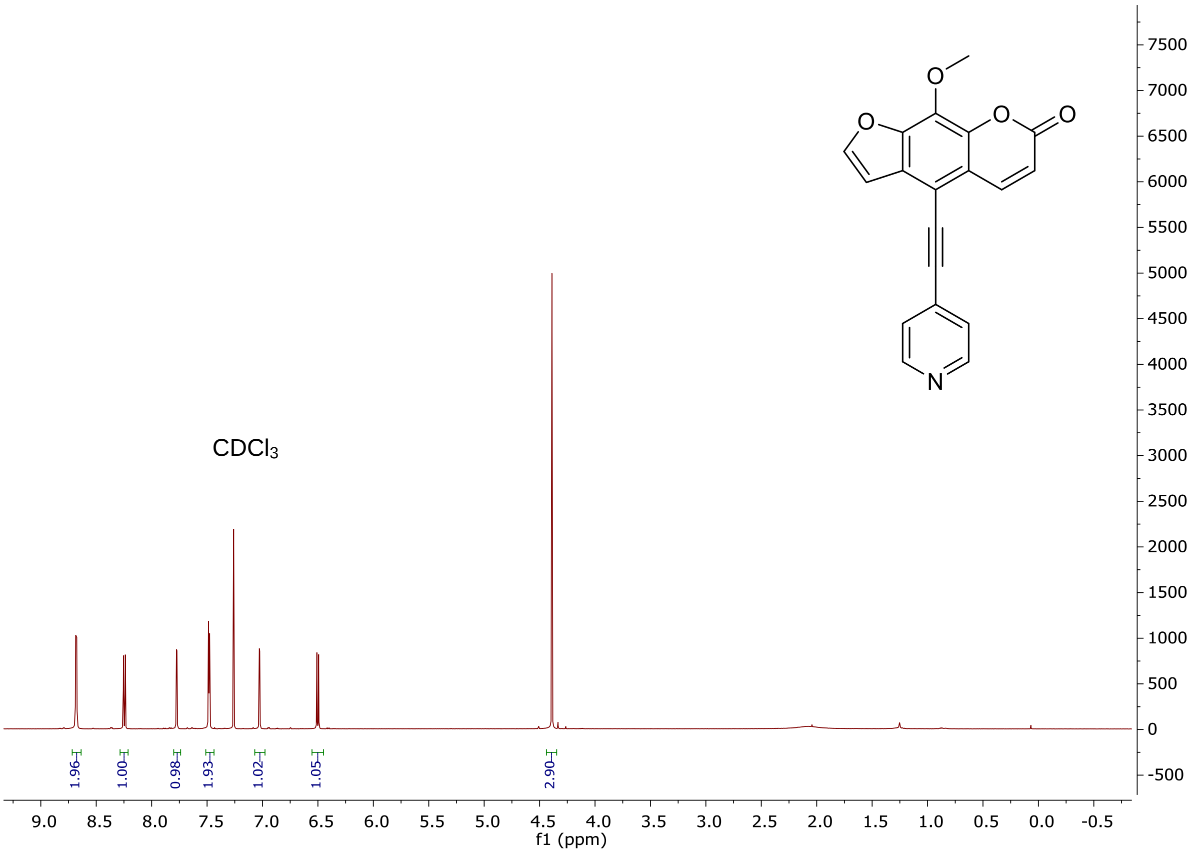

^{1}H NMR spectrum (600 MHz) of **13d**; recorded $CDCl_3$ at 298 K.

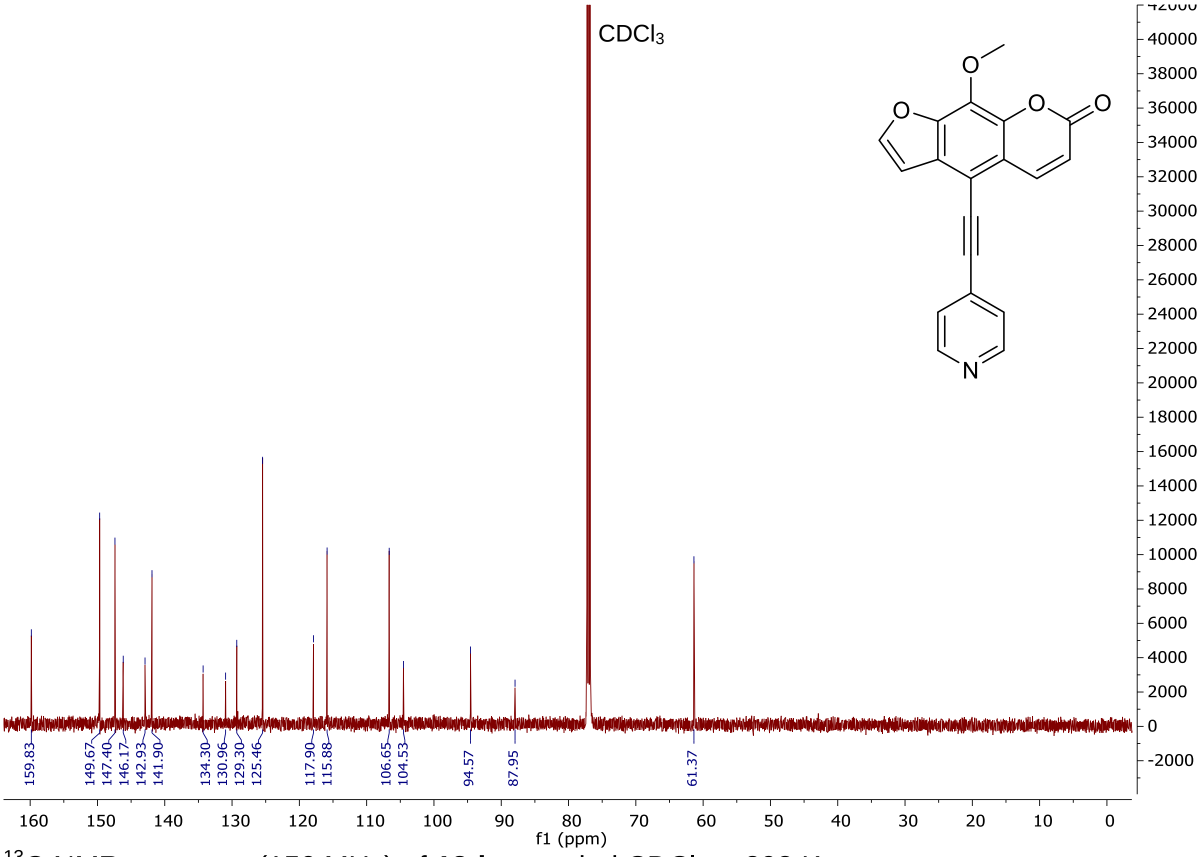

^{13}C NMR spectrum (150 MHz) of **13d**; recorded $CDCl_3$ at 298 K.

3.2.14. 4-((4-(Dimethylamino)phenyl)ethynyl)-9-methoxy-7*H*-furo[3,2-*g*]chromen-7-one (13e)

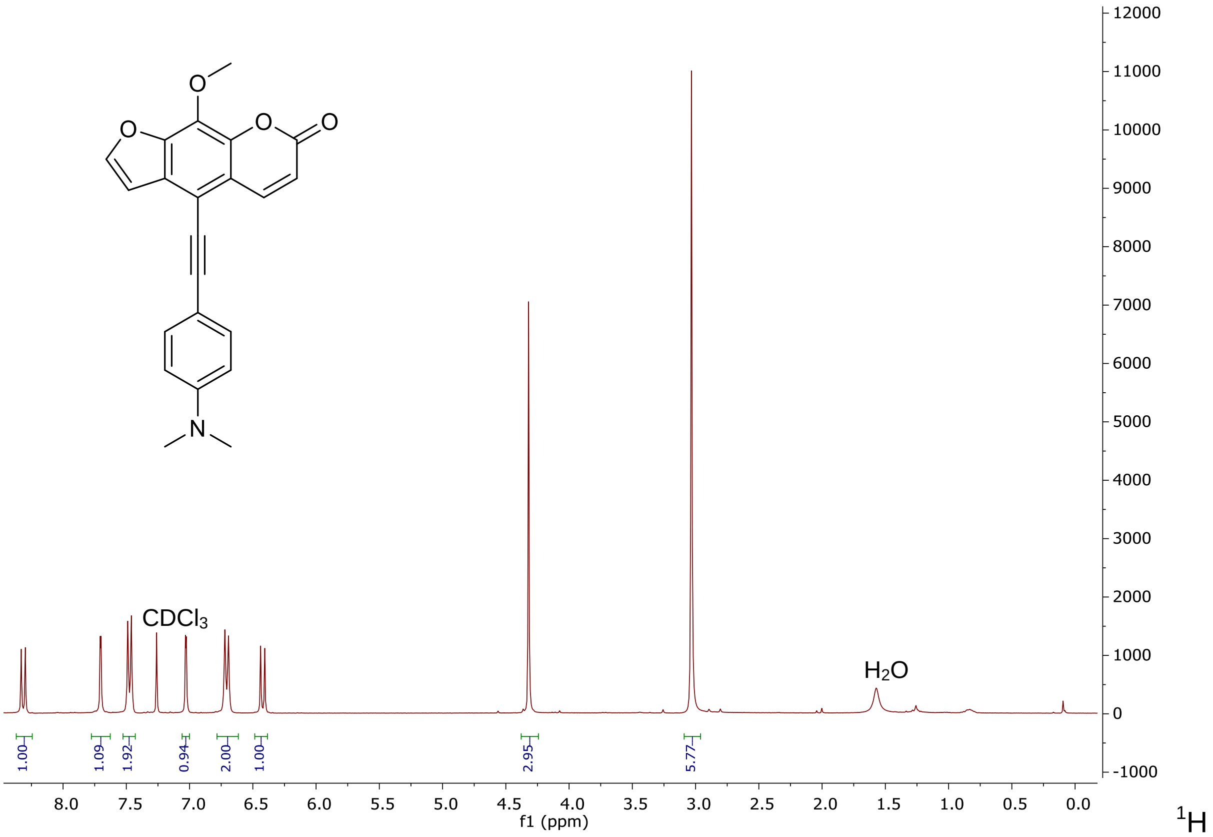

NMR spectrum (300 MHz) of **13e**; recorded $CDCl_3$ at 298 K.

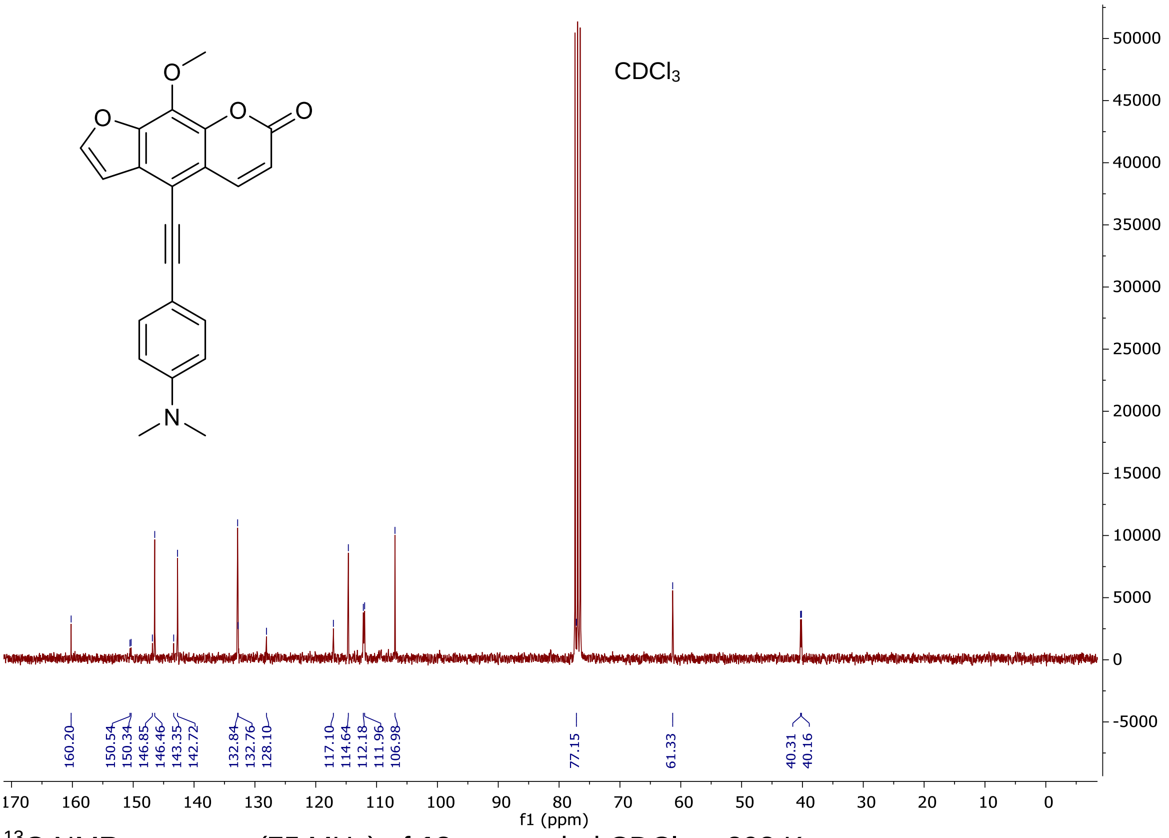

^{13}C NMR spectrum (75 MHz) of **13e**; recorded $CDCl_3$ at 298 K.

4-((4-(Dimethylamino)phenyl)ethynyl)-9-methoxy-7*H*-furo[3,2-*g*]chromen-7-one (13e+H⁺)

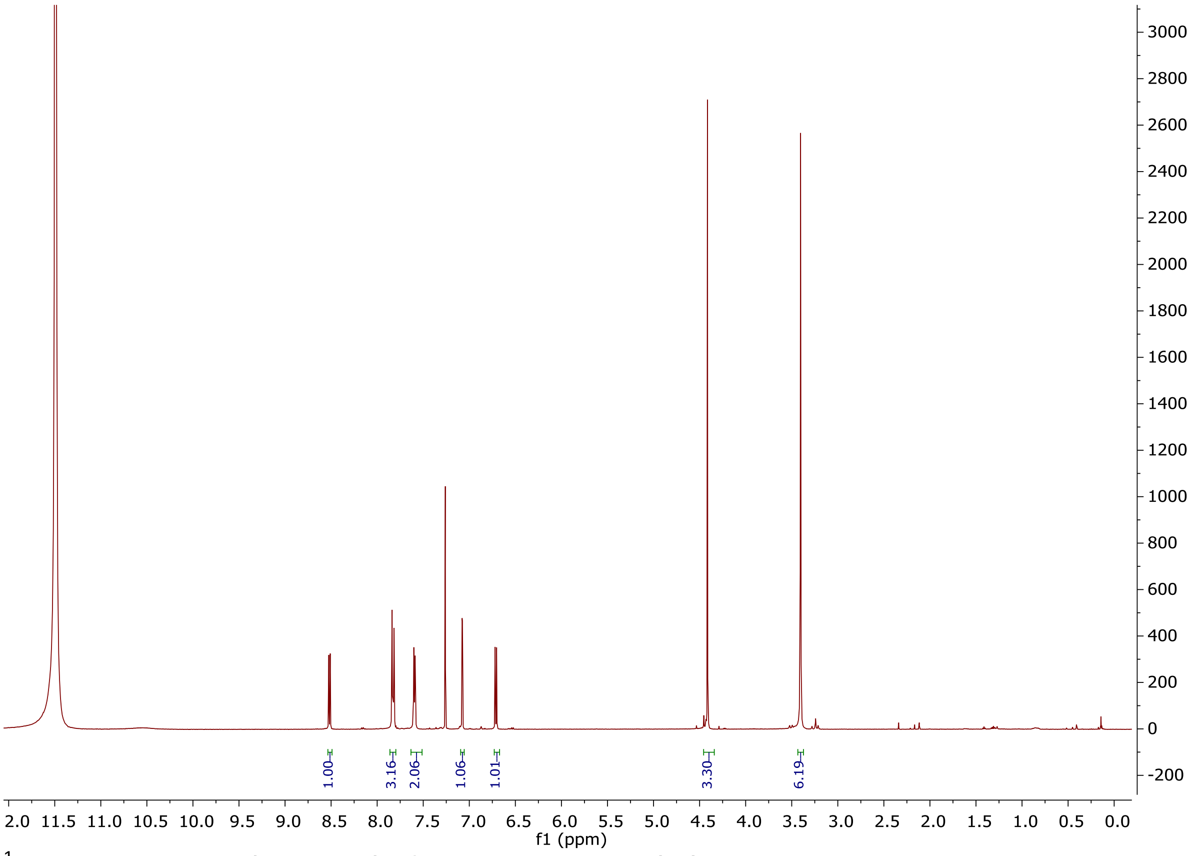

^{1}H NMR spectrum (300 MHz) of **13e + TFA**; recorded $CDCl_3$ at 298 K.

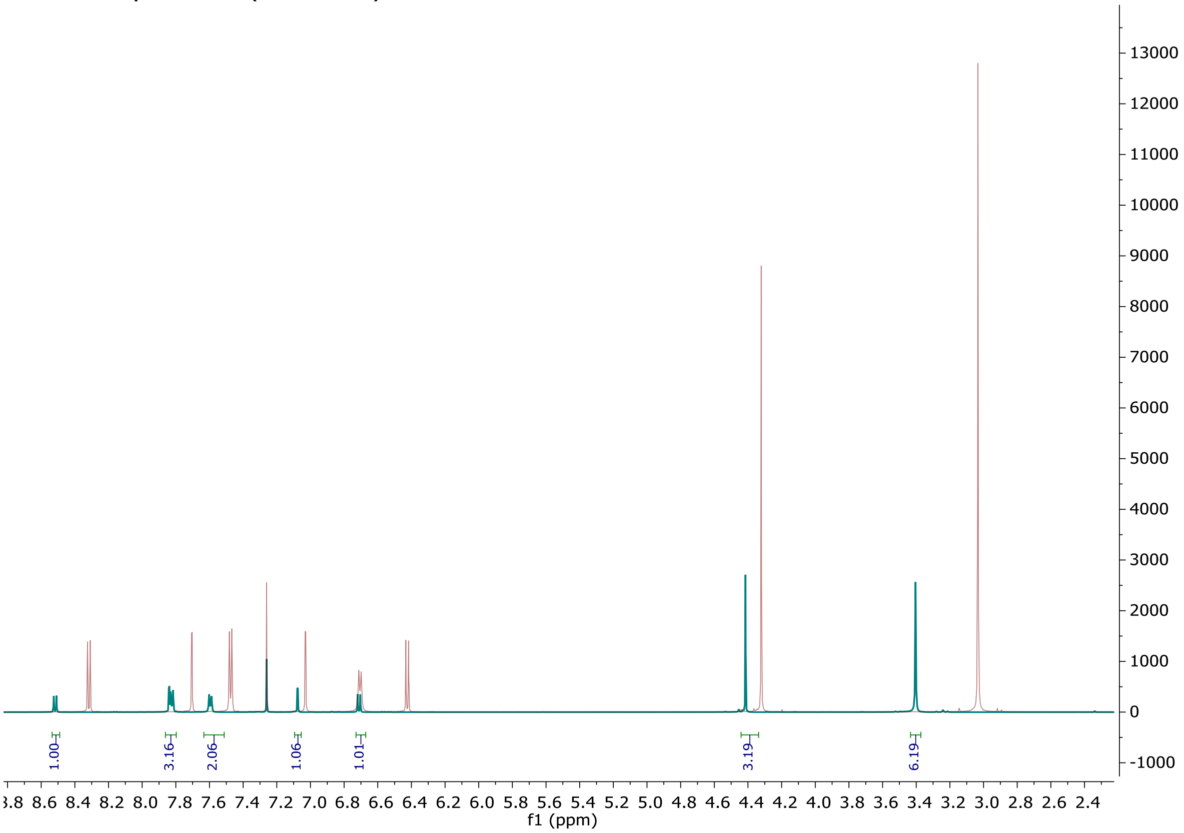

Stacked ^{1}H NMR spectrum (300 MHz) of **13e** (red) and **13e + TFA** (blue); recorded $CDCl_3$ at 298 K.

3.2.15. (*E*)-4-(2-(9-methoxy-7-oxo-7*H*-furo[3,2-*g*]chromen-4-yl)vinyl)benzonitrile (15a)

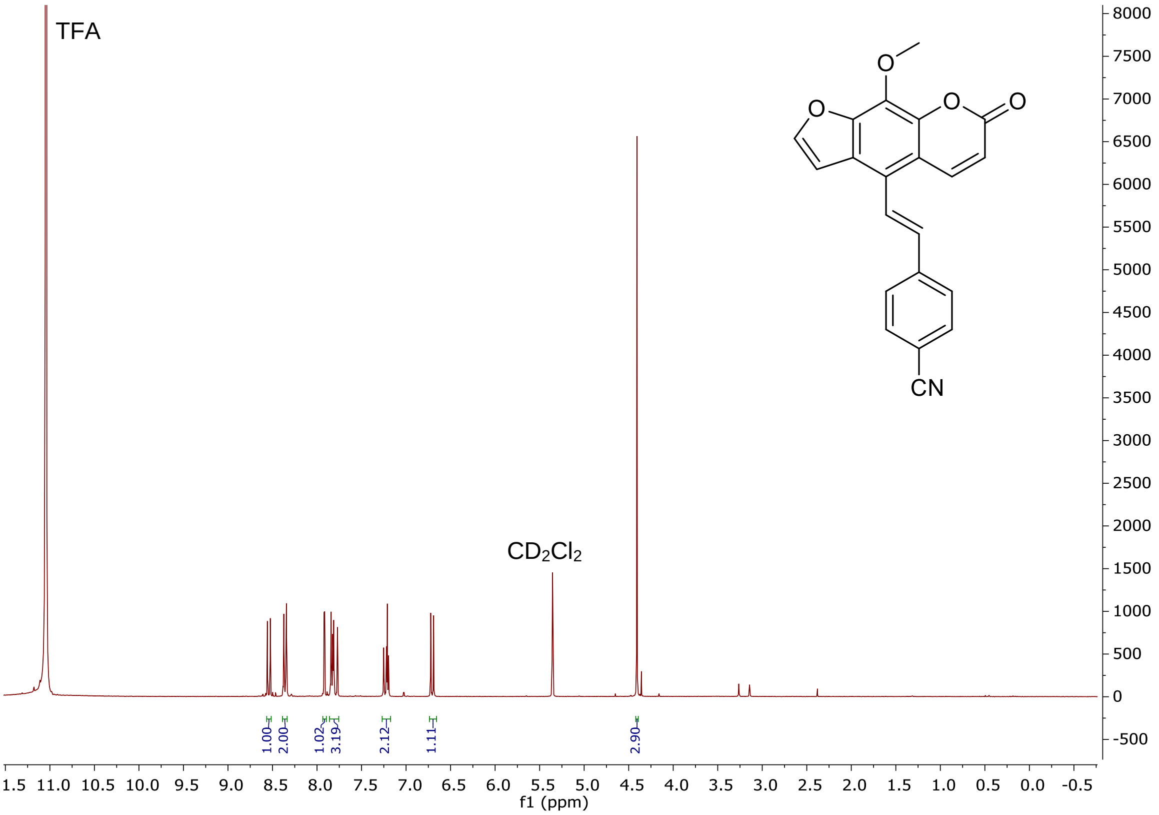

^1H NMR spectrum (300 MHz) of **15a**; recorded CD_2Cl_2/TFA at 298 K.

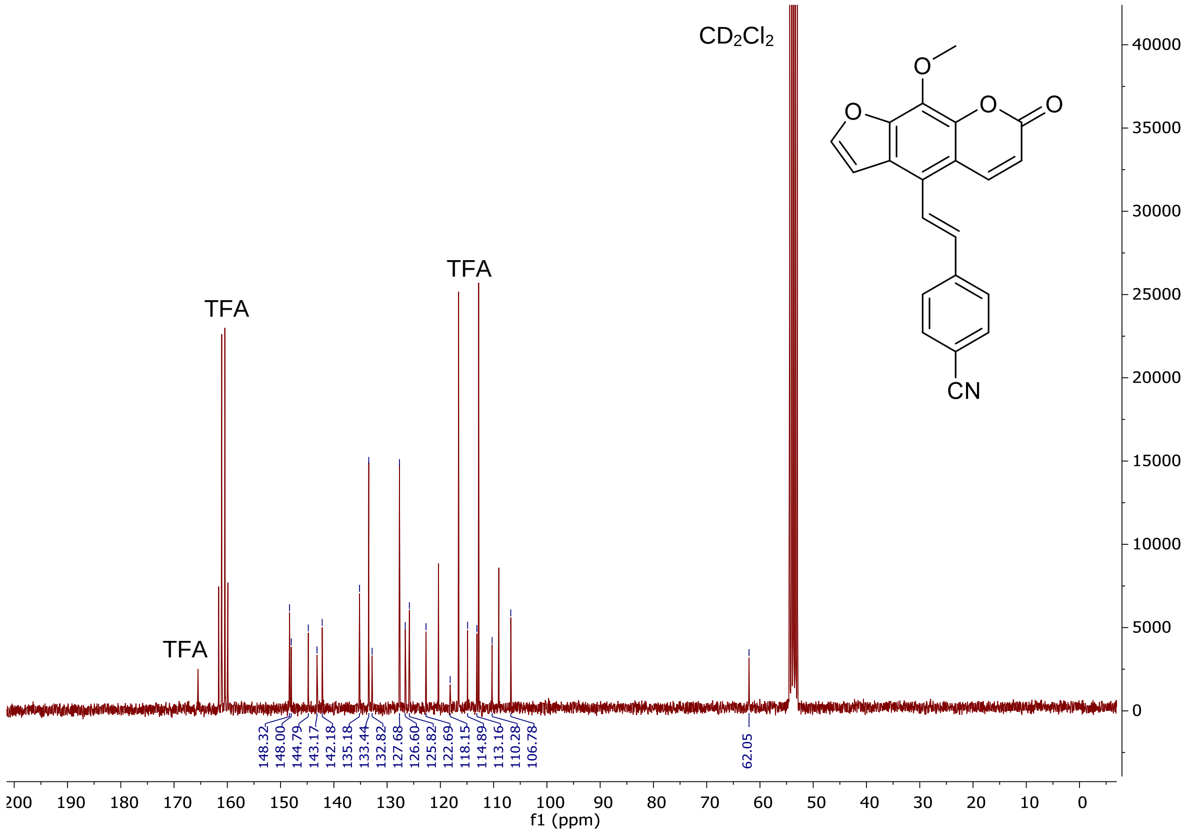

^{13}C NMR spectrum (75 MHz) of **15a**; recorded CD_2Cl_2/TFA at 298 K.

3.2.16. (*E*)-9-methoxy-4-(4-nitrostyryl)-7*H*-furo[3,2-*g*]chromen-7-one (15b)

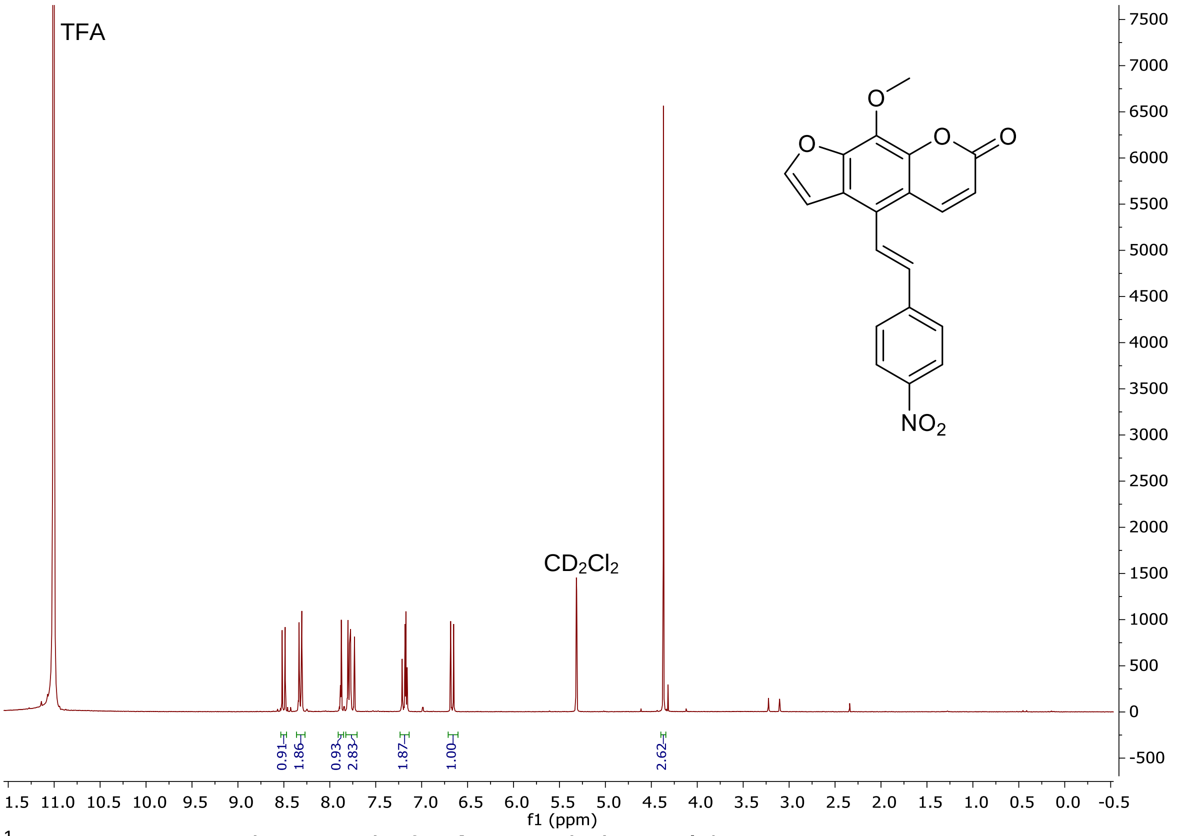

^{1}H NMR spectrum (300 MHz) of **15b**; recorded CD_2Cl_2/TFA at 298 K.

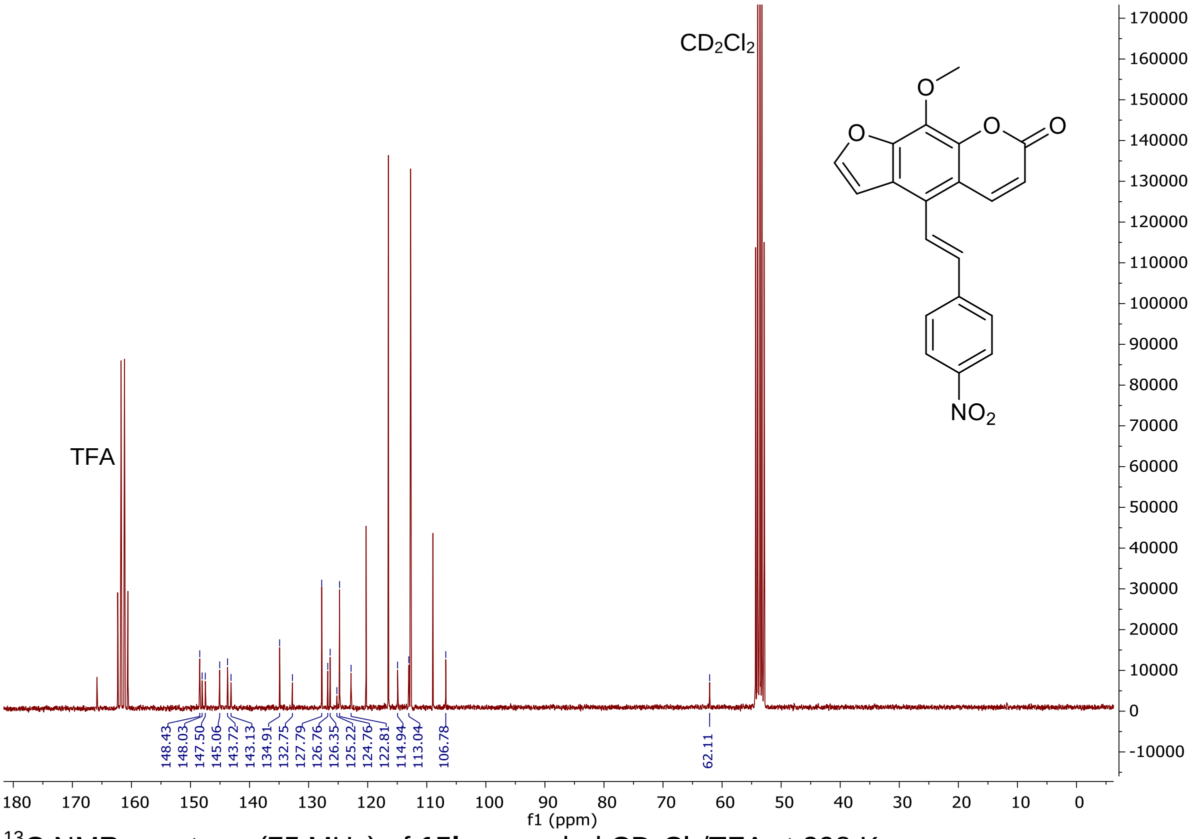

^{13}C NMR spectrum (75 MHz) of **15b**; recorded CD_2Cl_2/TFA at 298 K.

3.2.17. (*E*)-4-(2-(9-methoxy-7-oxo-7*H*-furo[3,2-*g*]chromen-4-yl)vinyl)benzaldehyde (15c)

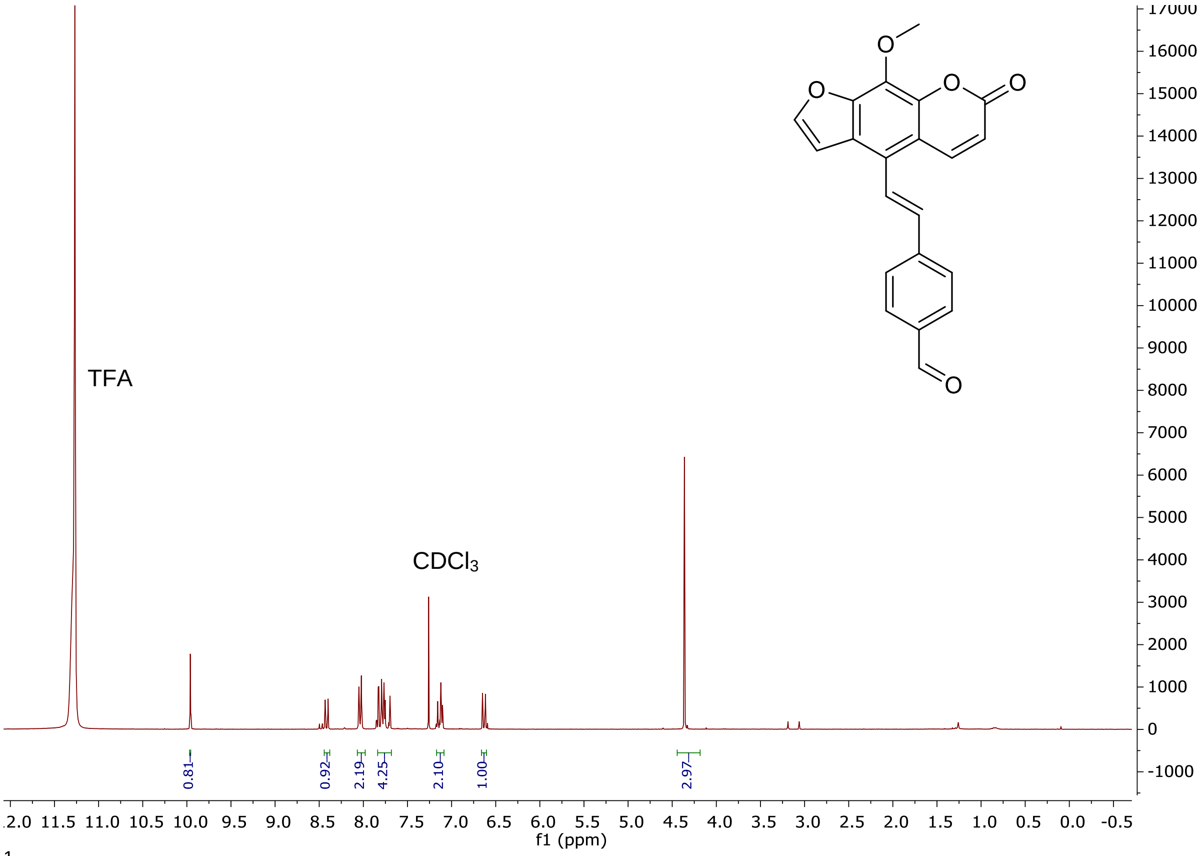

^{1}H NMR spectrum (300 MHz) of **15c**; recorded CDCl3/TFA at 298 K.

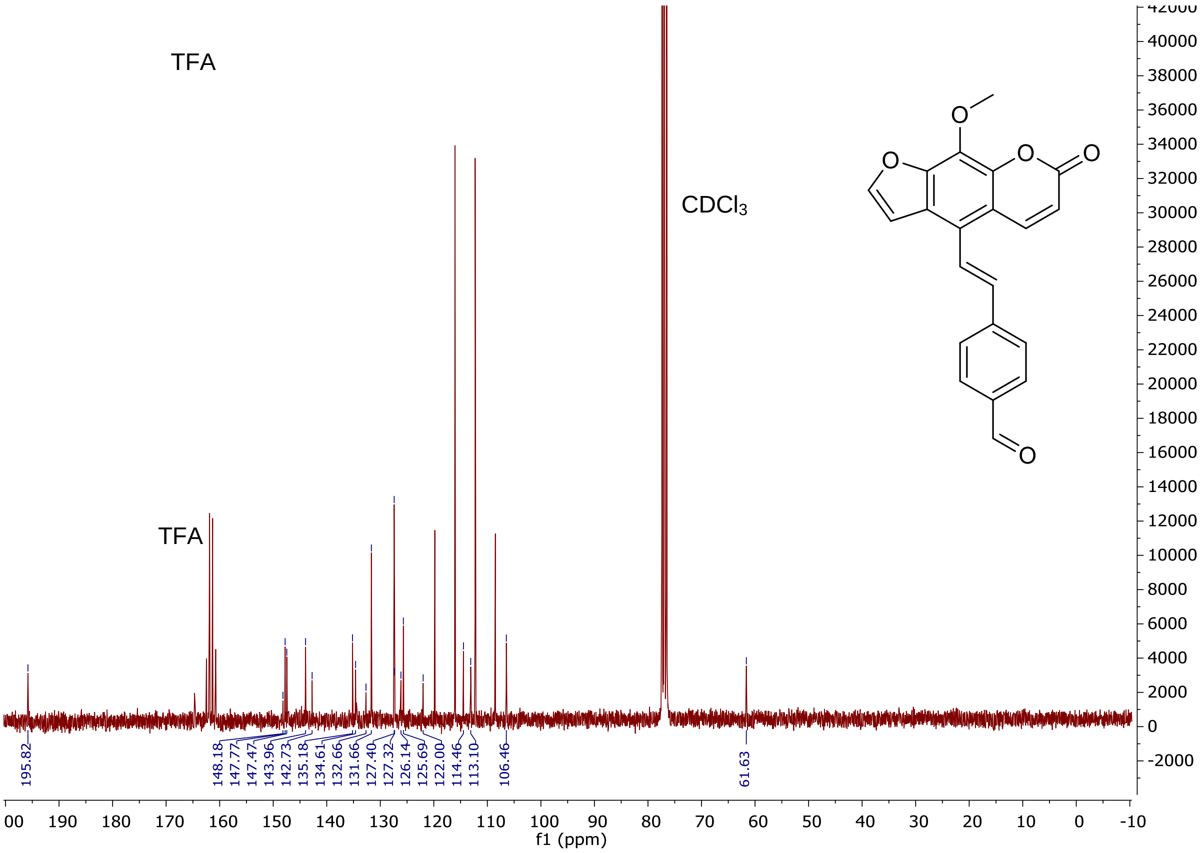

^{13}C NMR spectrum (75 MHz) of **15c**; recorded CDCl3/TFA at 298 K.

3.2.18. (*E*)-9-methoxy-4-(2-(pyridin-4-yl)vinyl)-7*H*-furo[3,2-*g*]chromen-7-one (15d)

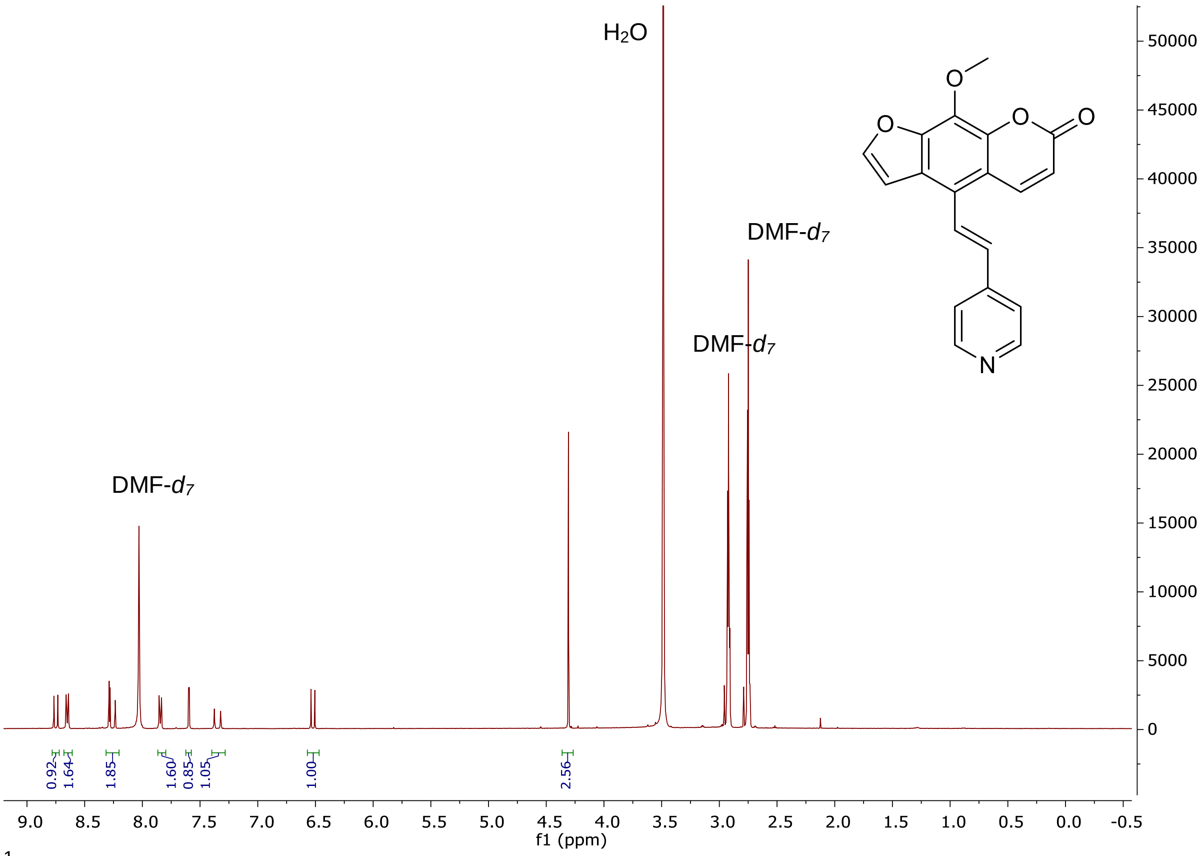

^{1}H NMR spectrum (300 MHz) of **15d**; recorded DMF-d_7 at 298 K.

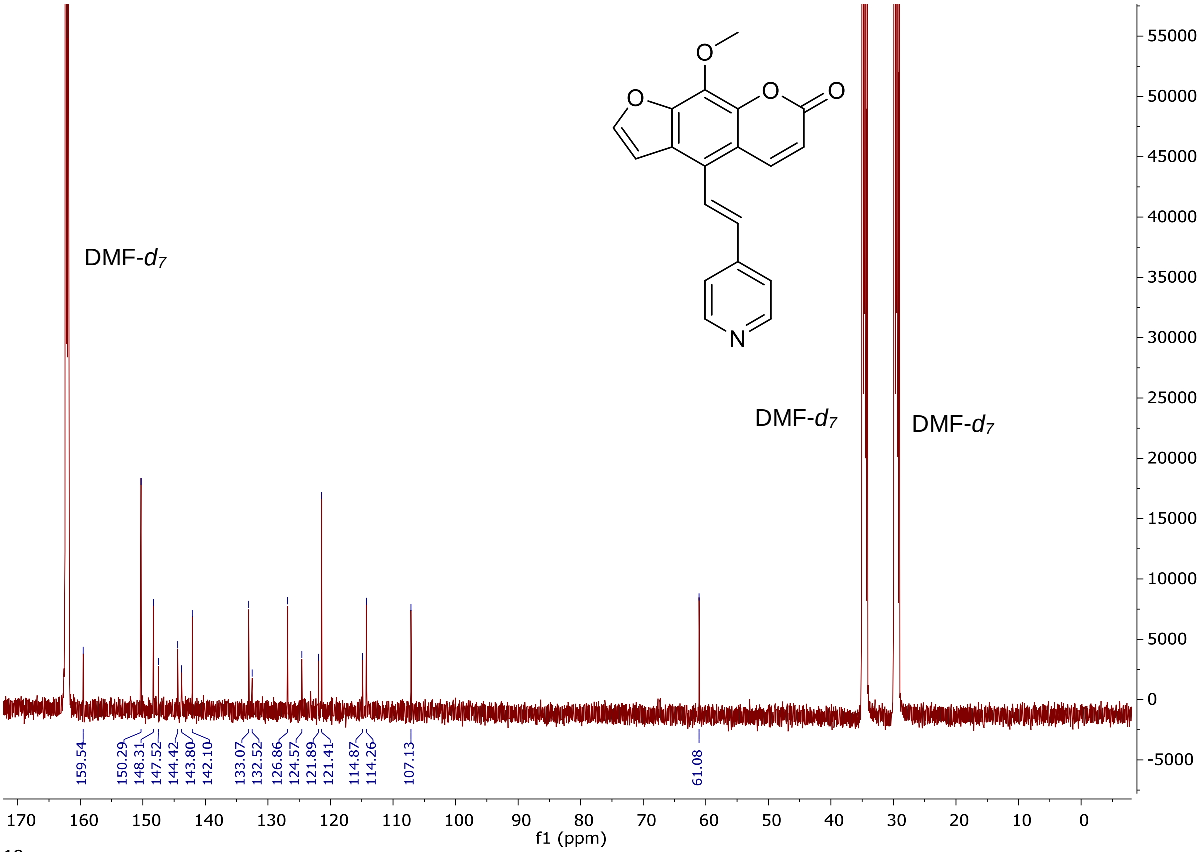

^{13}C NMR spectrum (75 MHz) of **15d**; recorded DMF-d_7 at 298 K.

3.2.19. (*E*)-4-(4-(Dimethylamino)styryl)-9-methoxy-7*H*-furo[3,2-*g*]chromen-7-one (15e)

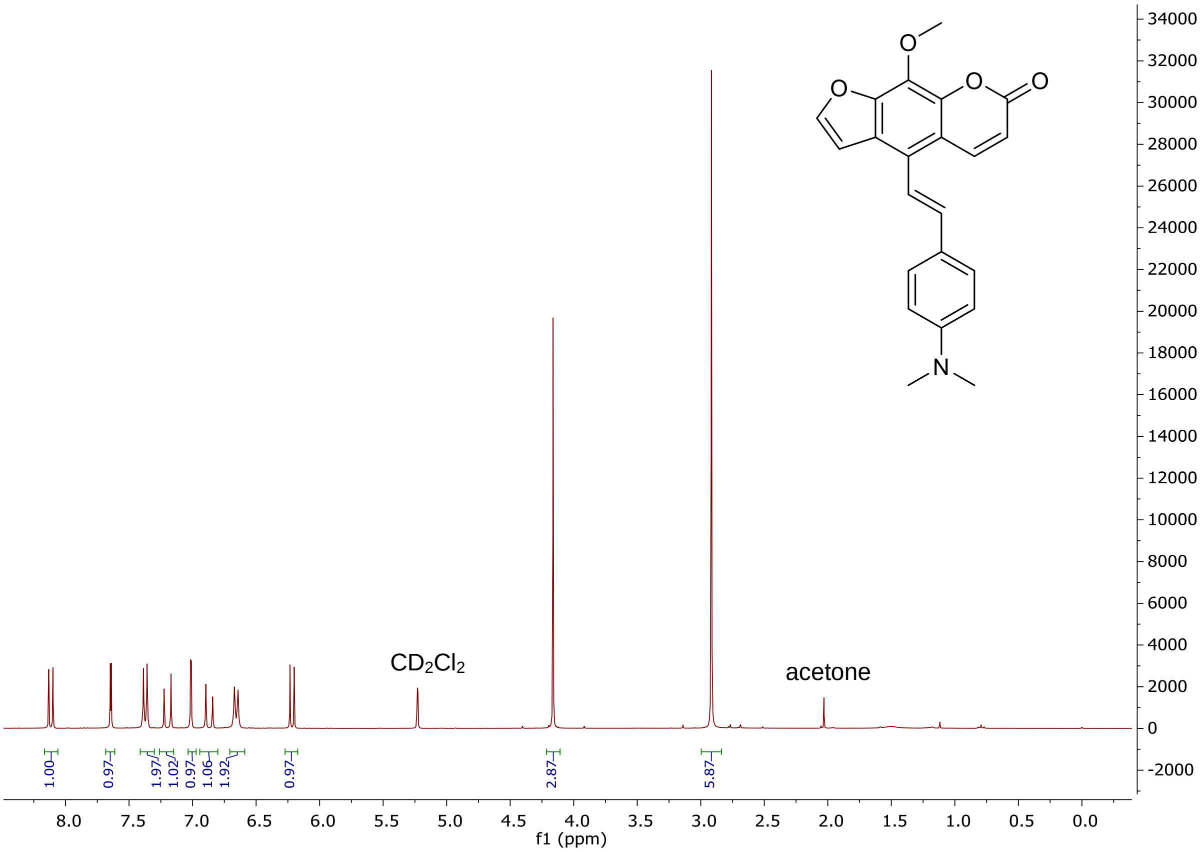

^{1}H NMR spectrum (300 MHz) of **15e**; recorded CD_2Cl_2 at 298 K.

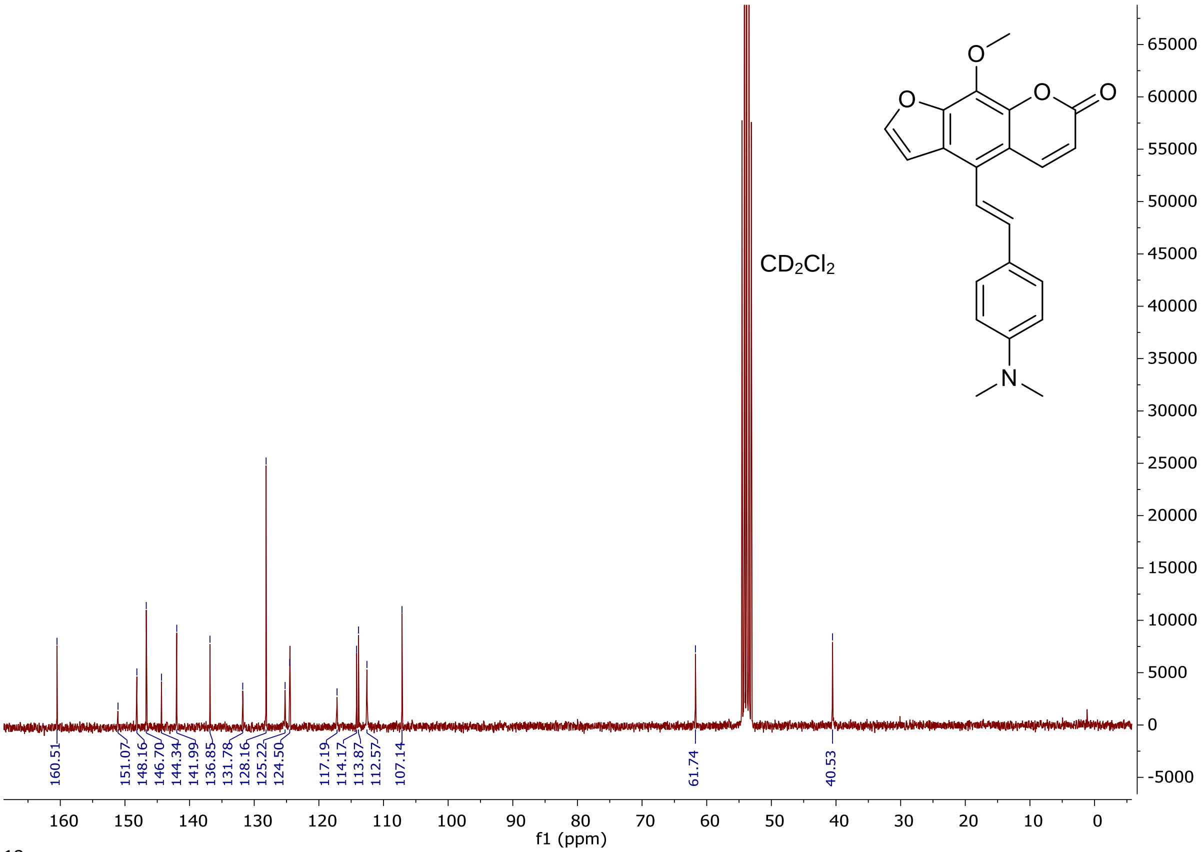

^{13}C NMR spectrum (75 MHz) of **15e**; recorded CD_2Cl_2 at 298 K.

4. Absorption and emission spectra

4.1. Absorption spectra of compound 8

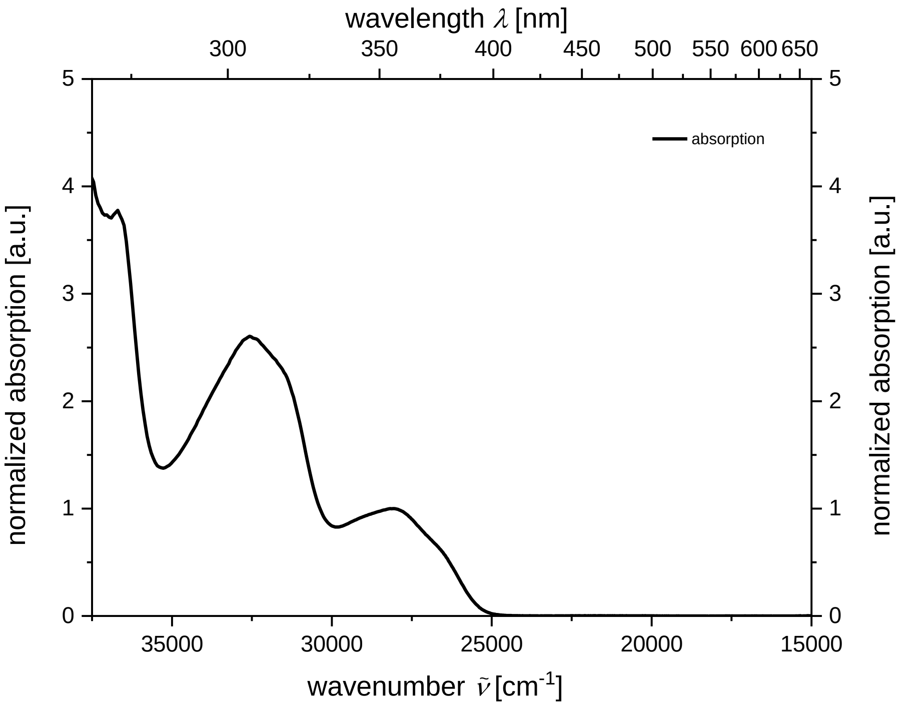

4.2. Absorption spectra of compound 9

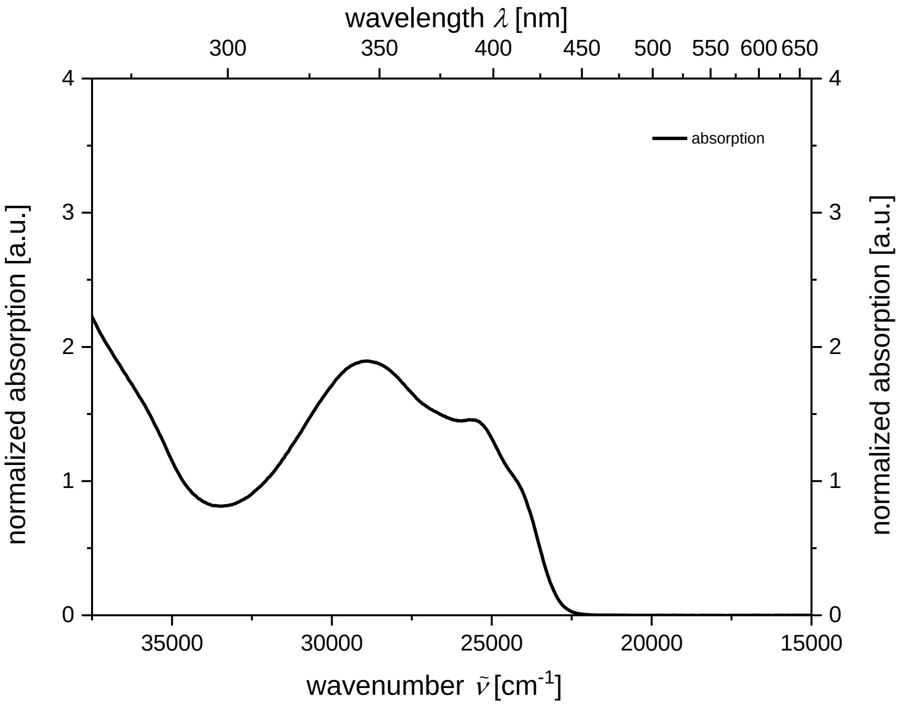

4.3. Absorption spectra of compound 11a

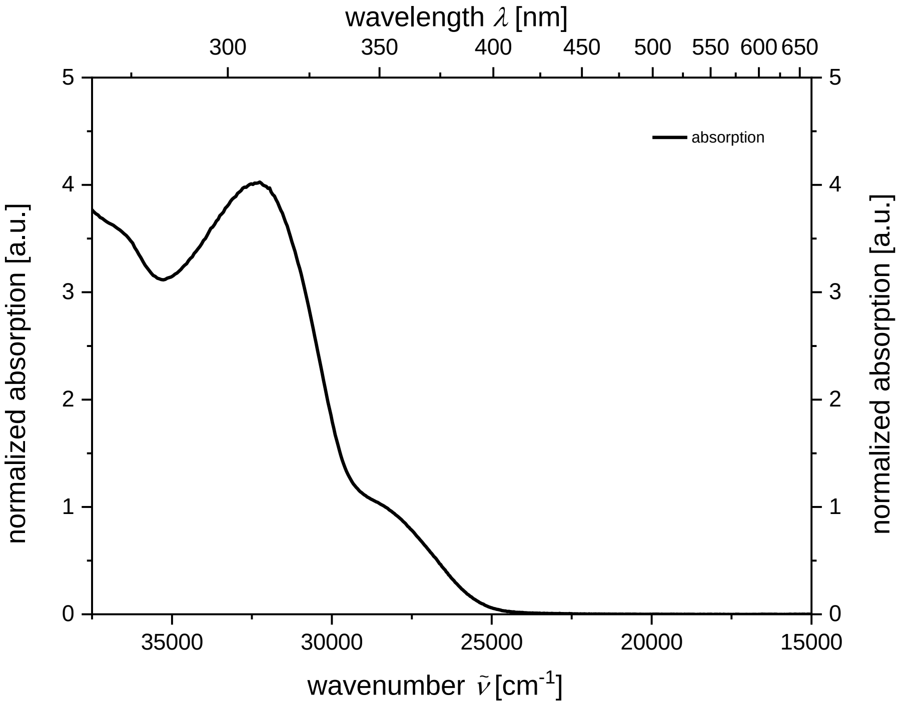

4.4. Absorption spectra of compound 11b

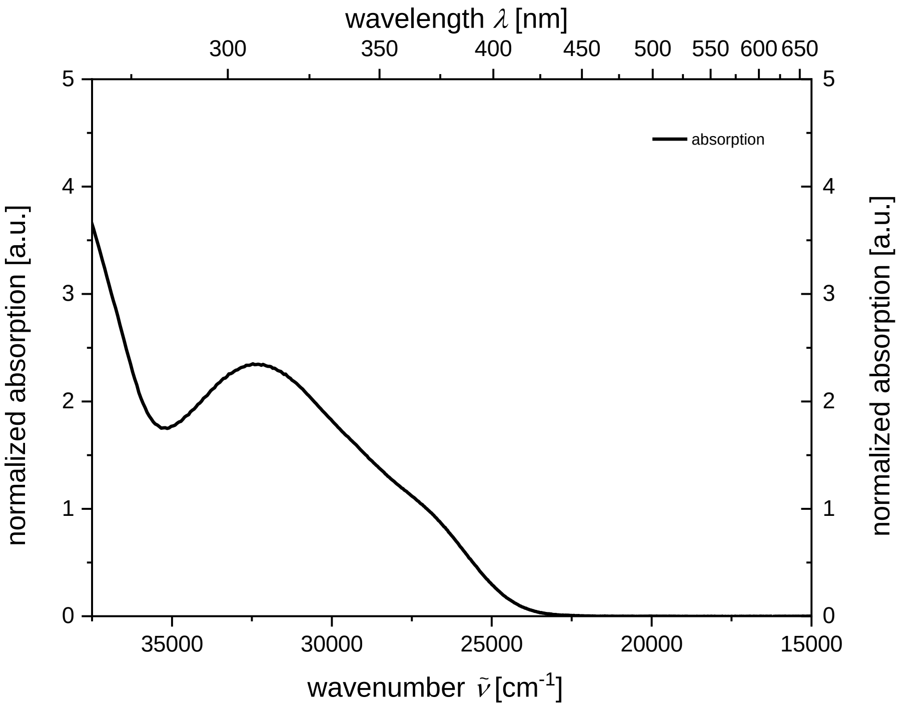

4.5. Absorption spectra of compound 11c

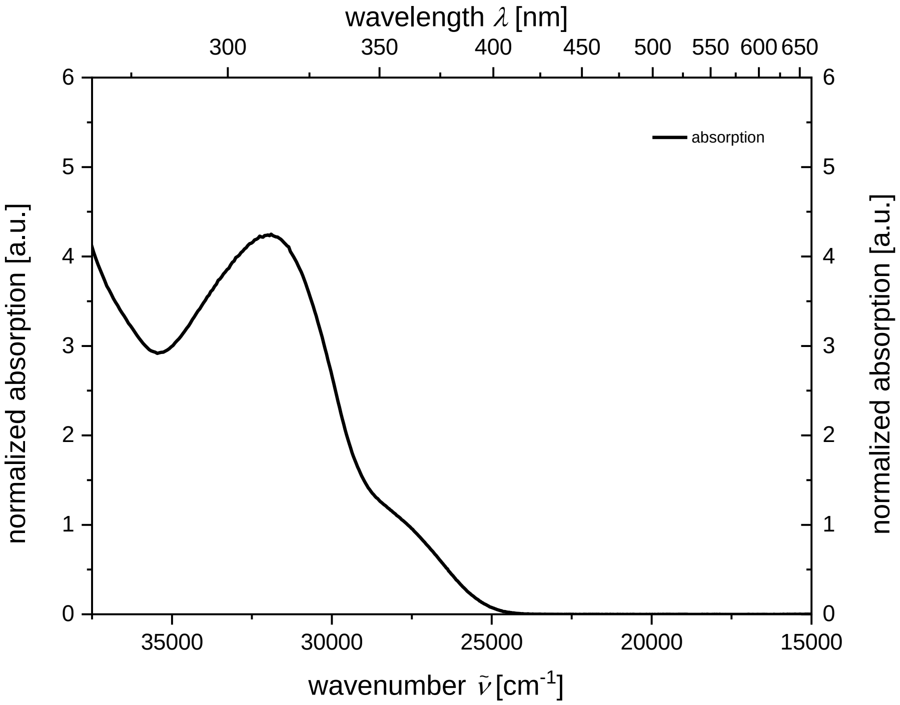

4.6. Absorption spectra of compound 11d

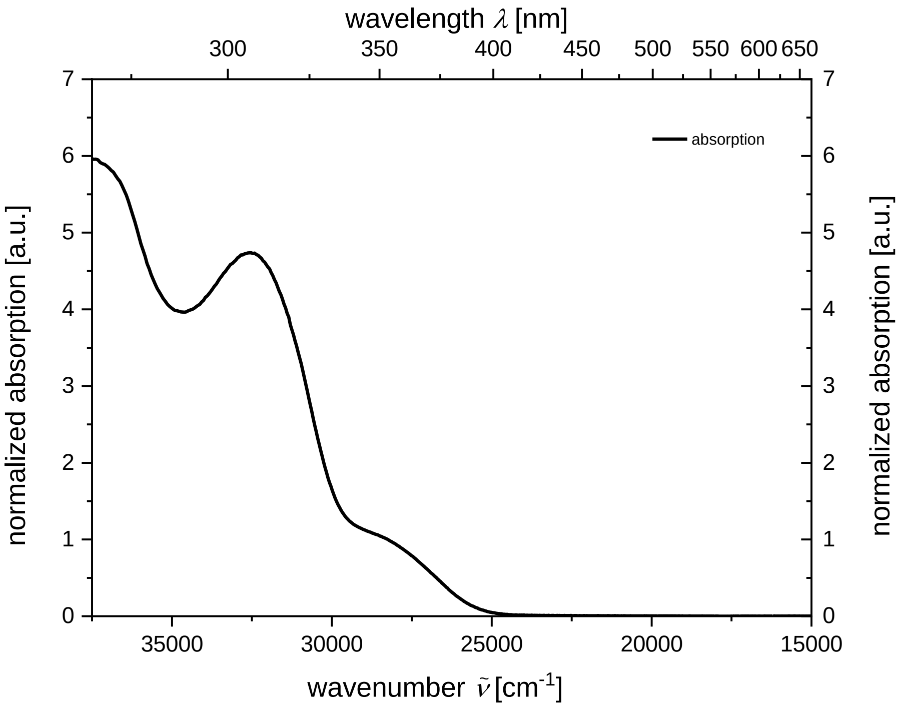

4.6. Absorption and emission spectra of compound 11e

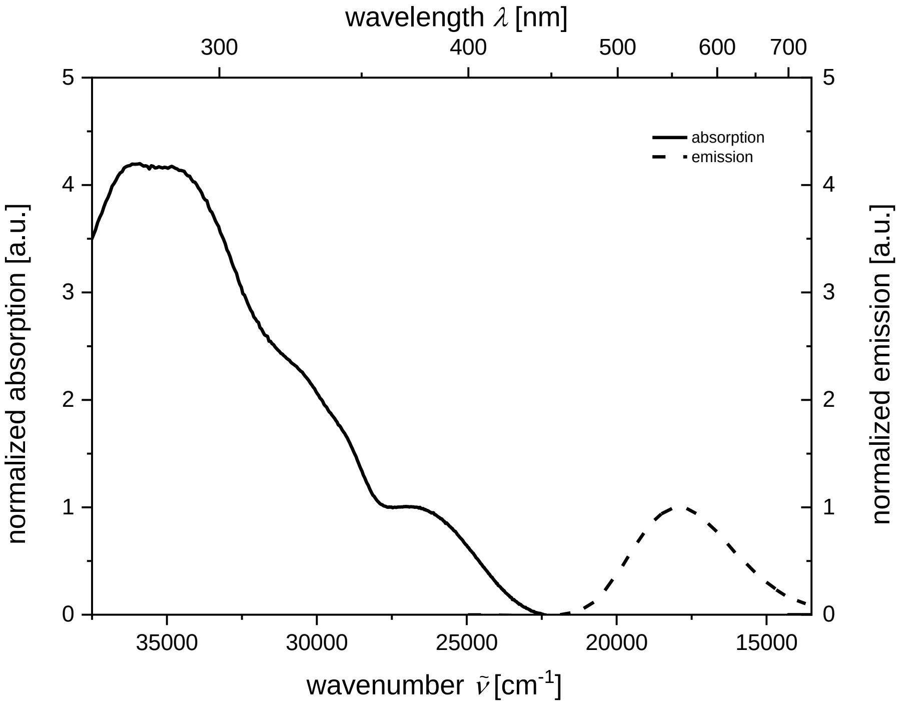

4.7. Absorption spectra of compound 11f

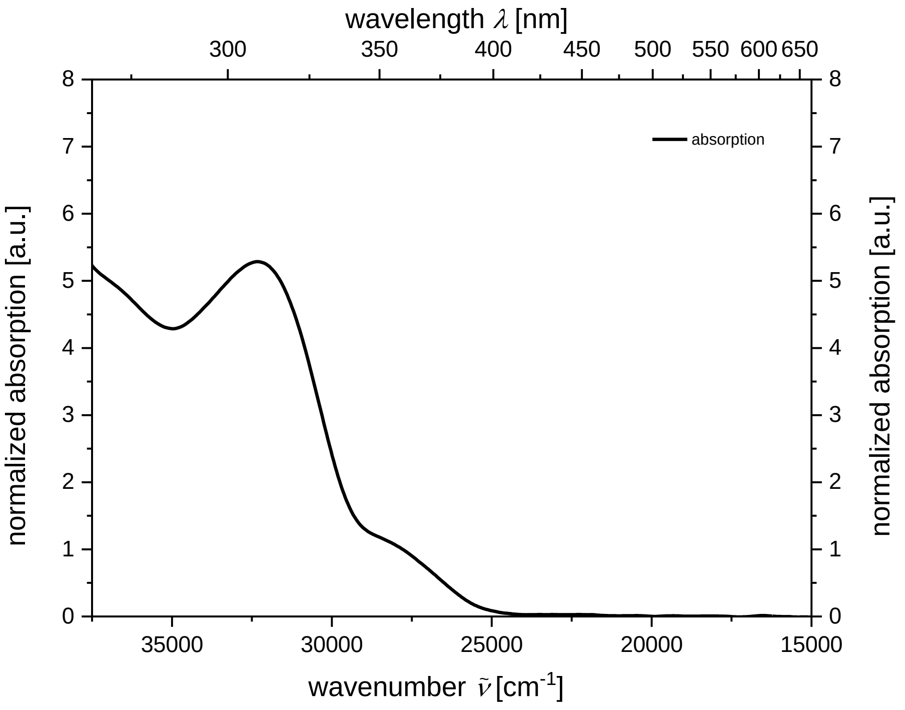

4.8. Absorption spectra of compound 11g

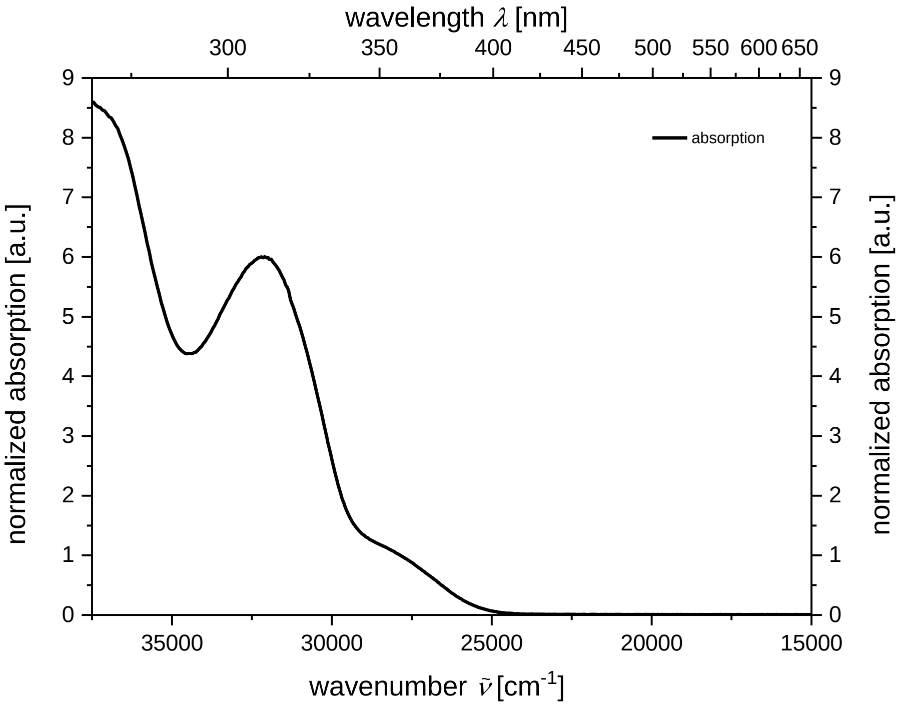

4.9. Absorption and emission spectra of compound 13a

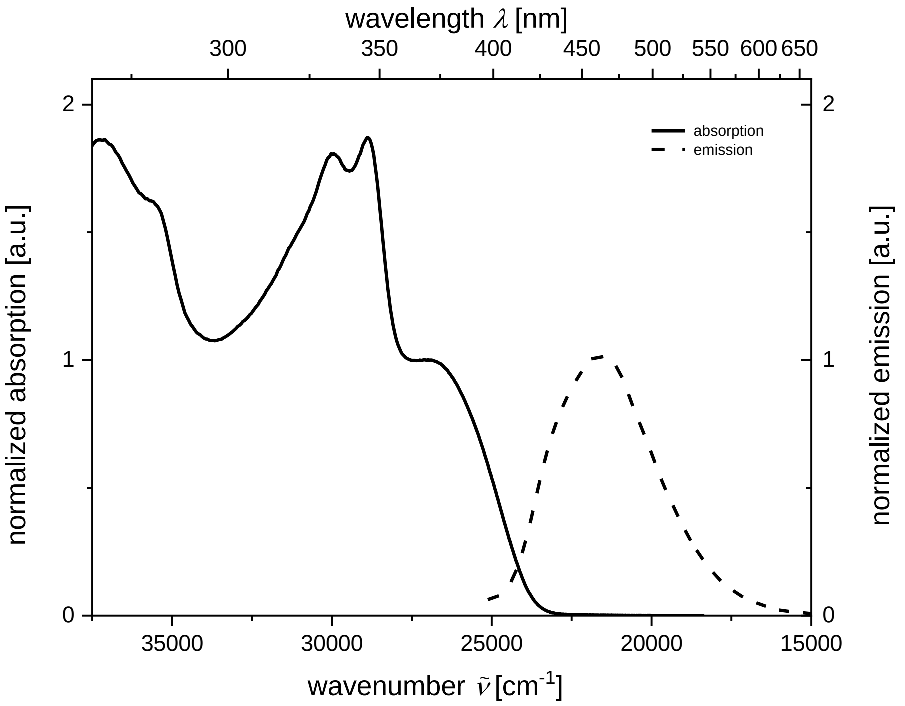

4.10. Absorption and emission spectra of compound 13b

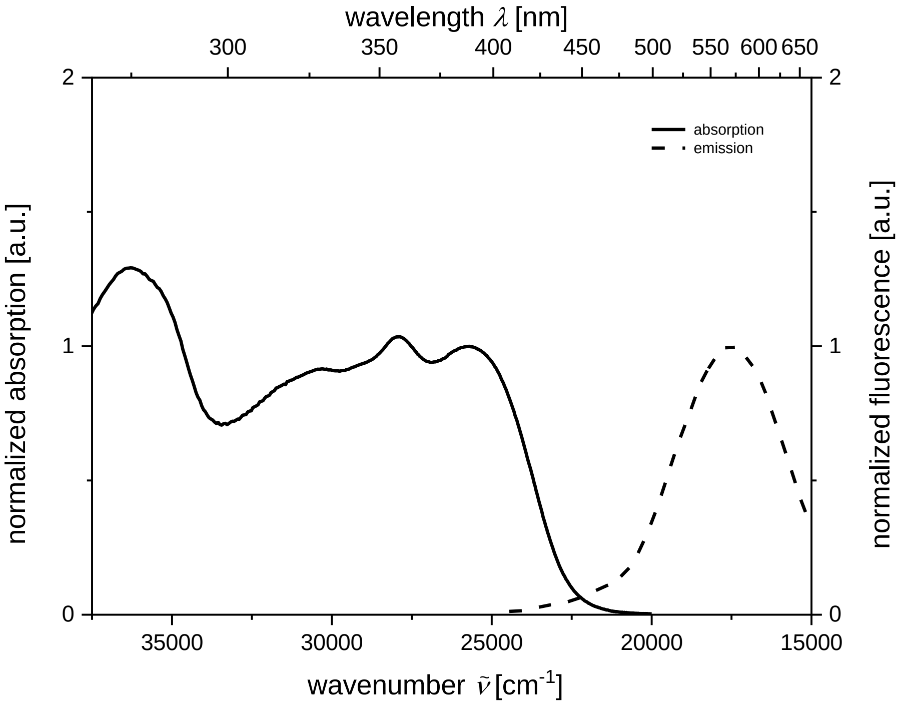

4.11. Absorption and emission spectra of compound 13c

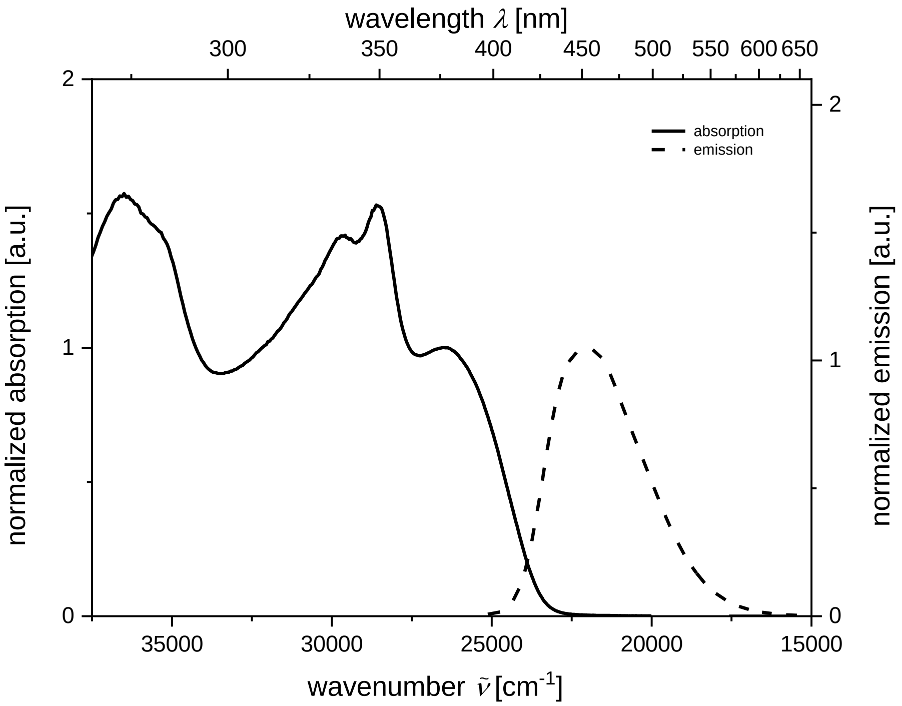

4.12. Absorption and emission spectra of compound 13d

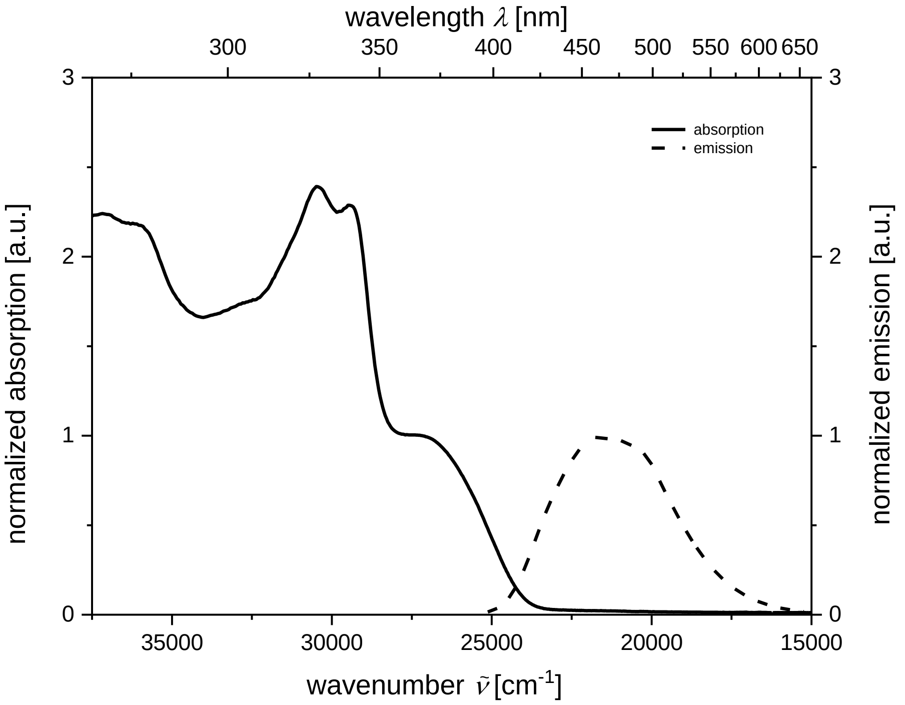

4.13. Absorption and emission spectra of compound 13e

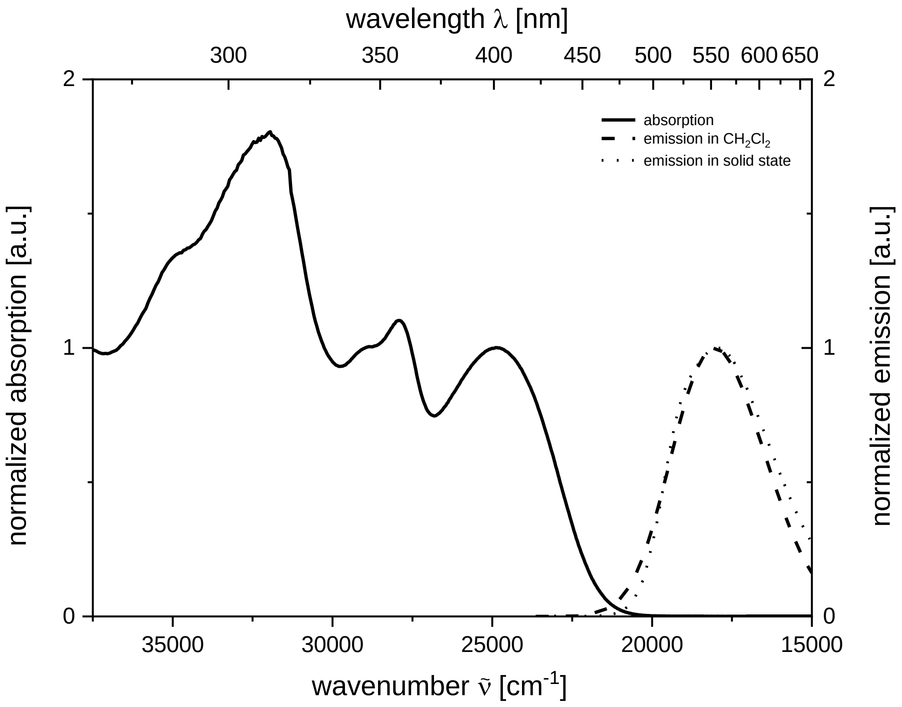

4.14. Absorption and emission spectra of compound 15a

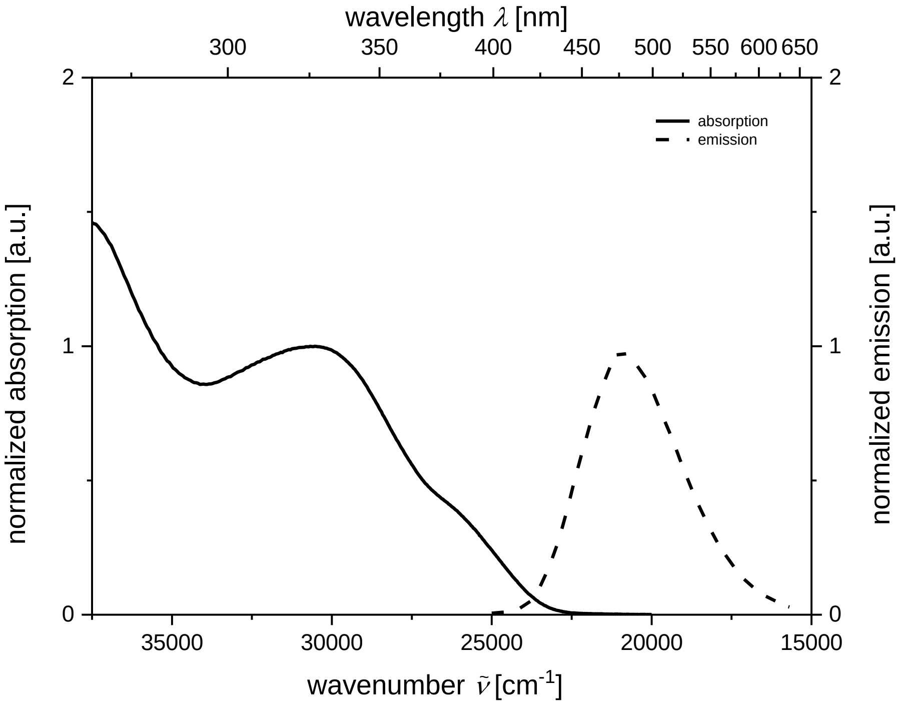

4.15. Absorption and emission spectra of compound 15b

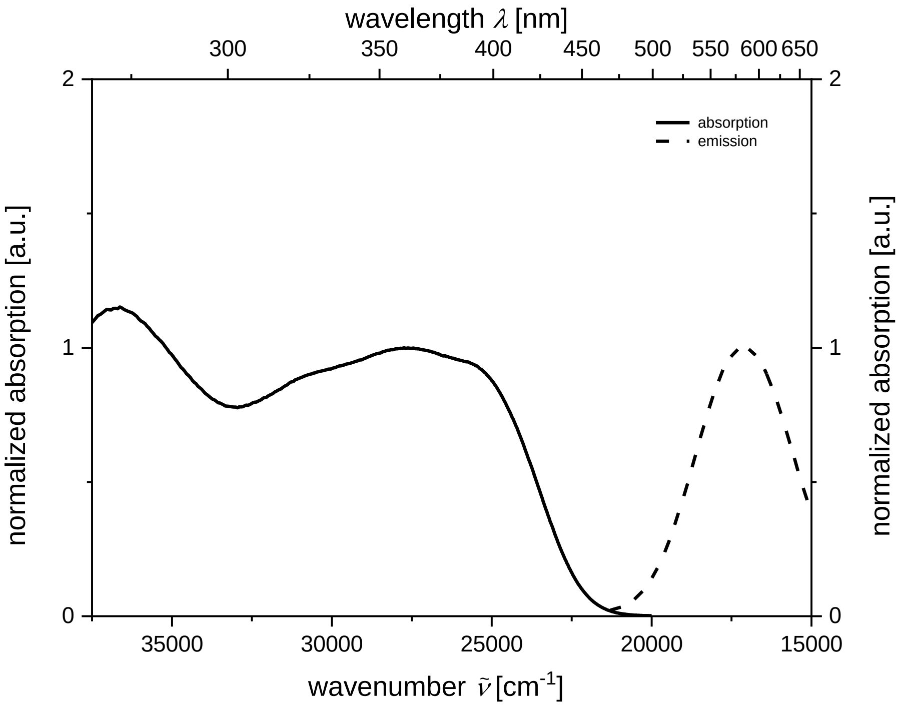

4.16. Absorption and emission spectra of compound 15c

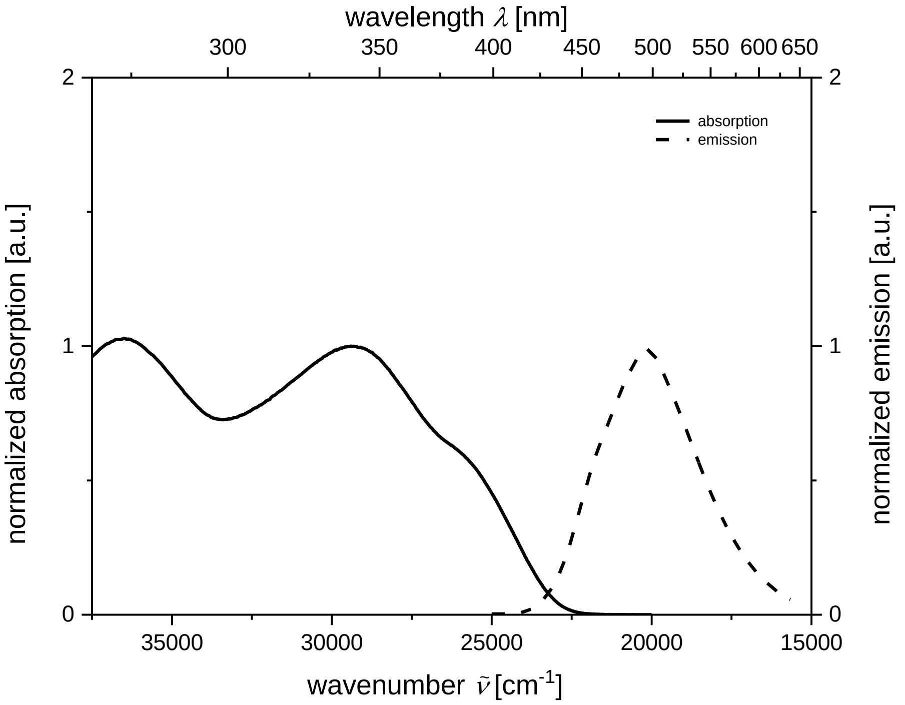

4.17. Absorption and emission spectra of compound 15d

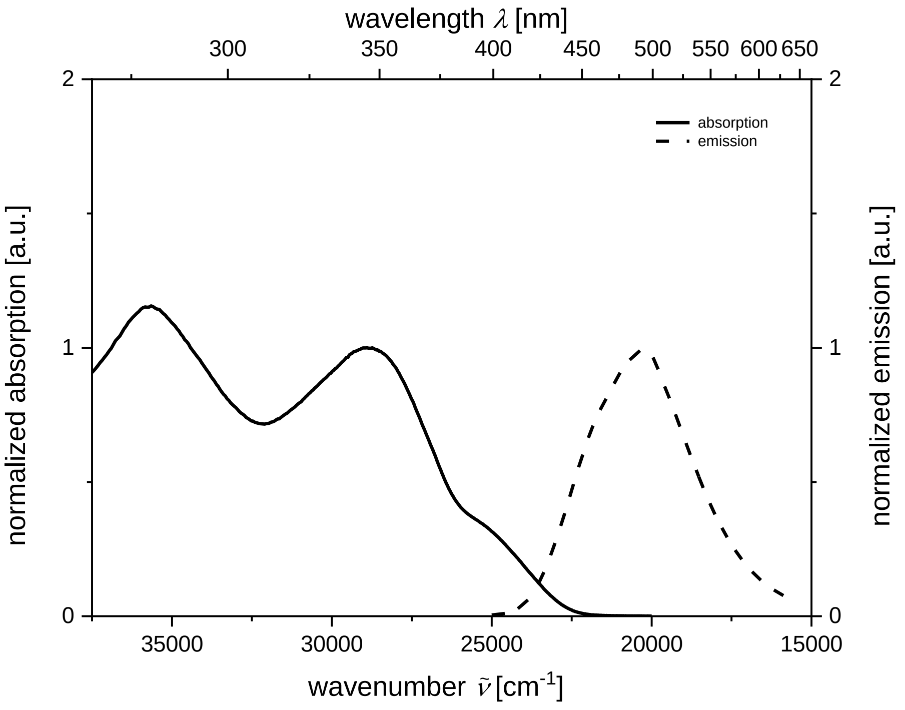

4.18. Absorption and emission spectra of compound 15e

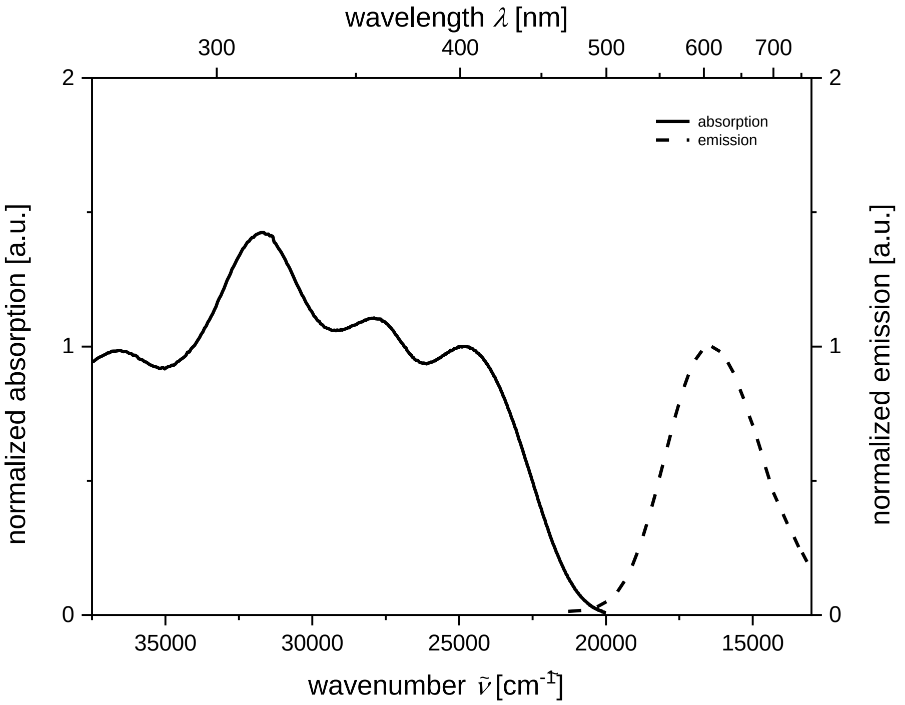

4.19. Emission spectra of PMMA-films of compounds 11e, 13e, 15e and solid state emission of compound 13e

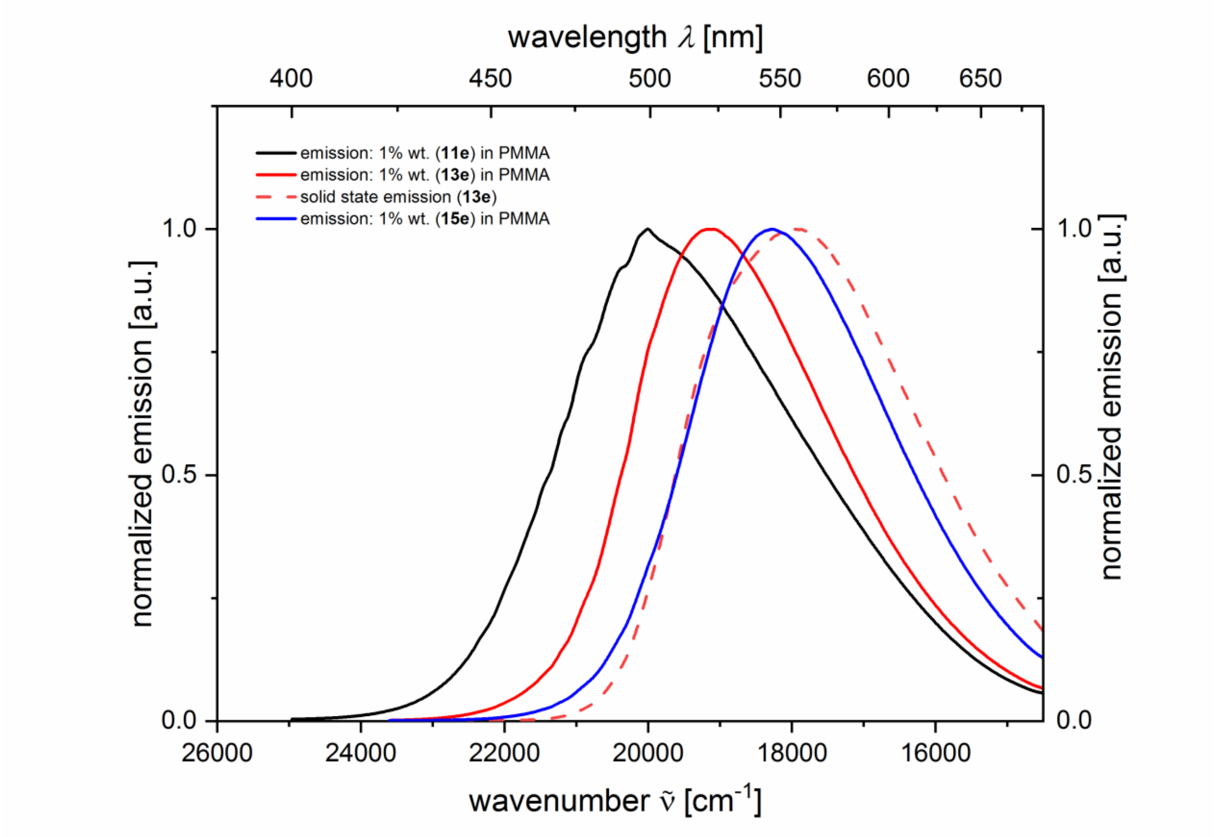

5. Solvatochromicity

5.1. Solvatochromicity of compound 11e

Table 6: Absorption and emission maxima of compound **11e** measured in different solvents.

solvent	refractive index[1, 4] $n_D{}^{20}$	permittivity[4] ε_r	orientation polarizability Δf	$\lambda_{max,Abs}$[a] [nm]	$\lambda_{max,Em}$[b] [nm]	$\Delta\tilde{\nu}$ [c] [cm^{-1}]
**cyclohexane**	1.4262	2.02	-0.0016497	362	471	6400
**toluene**	1.4969	2.38	0.013235	367	498	7200
**chloroform**	1.4459	4.81	0.14826	370	536	8400
**dichloromethane**	1.4242	8.93	0.21710	380	558	8400
**DMF**	1.4305	36.71	0.27286	375	626	10700
**acetonitrile**	1.3441	35.94	0.30457	370	630	11200

[a] Recorded in different solvents, T = 293 K, c = 10^{-5} M. [b] Recorded in different solvents, T = 293 K, c = 10^{-7} M. [c] $\Delta\tilde{\nu} = \frac{1}{\lambda_{max,Abs}} - \frac{1}{\lambda_{max,Em}}$.

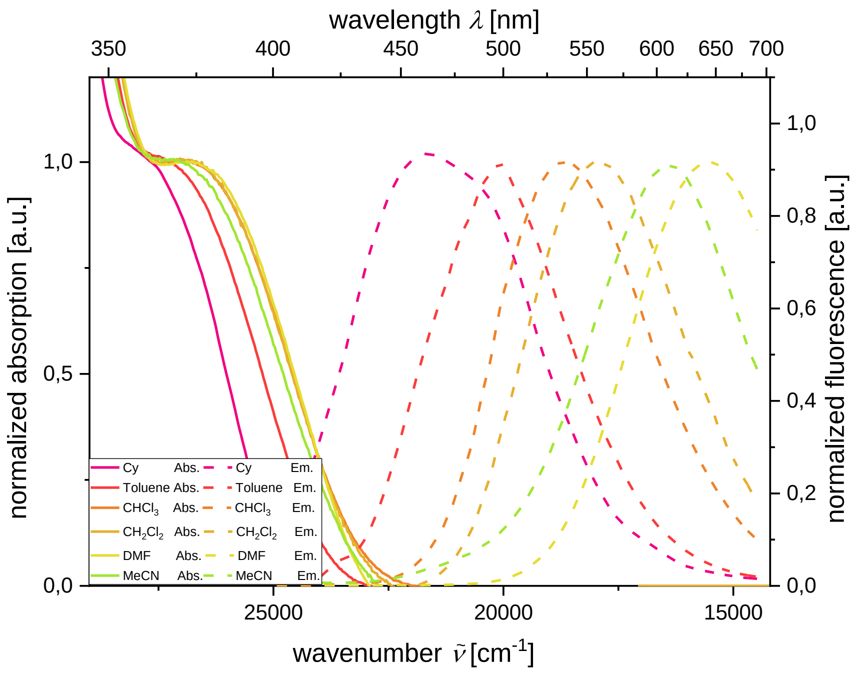

Figure 1. UV/Vis absorption (bold lines) and emission (dashed lines) spectra of compound **11e** measured in six solvents of different polarity recorded at T = 293 K).

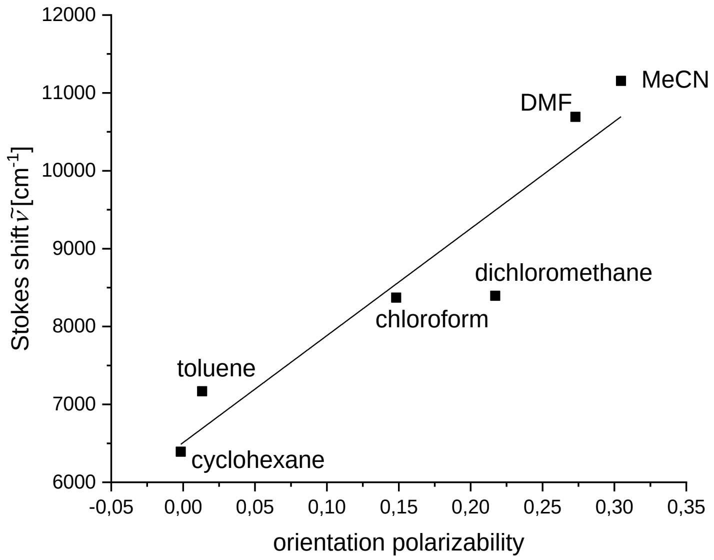

Figure 2. Lippert-Mataga plot for compound **11e** (n = 6, r^2 = 0.95).

5.2. Solvatochromicity of compound 13e

Table 7: Absorption and emission maxima of compound **13e** measured in different solvents.

solvent	refractive index[4] $n_D{}^{20}$	permittivity[4] ε_r	orientation polarizability Δf	$\lambda_{max,Abs}$[a] [nm]	$\lambda_{max,Em}$[b] [nm]	$\Delta\tilde{\nu}$ [c] [cm^{-1}]
**cyclohexane**	1.4262	2.02	-0.0016497	390	442	3000
**chloroform**	1.4459	4.81	0.14826	402	533	6100
**ethyl acetate**	1.3724	6.02	0.19964	394	530	6500
**tetrahydrofuran**	1.4072	7.58	0.20957	397	550	7000
**dichloromethane**	1.4242	8.93	0.21710	403	553	6700
**DMF**	1.4305	36.71	0,27286	403	616	8600
**DMSO**	1.4793	46.45	0.26301	408	633	8700
**acetonitrile**	1.3441	35.94	0.30457	398	620	9000

[a] Recorded in different solvents, T = 293 K, c = 10^{-5} M. [b] Recorded in different solvents, T = 293 K, c = 10^{-7} M. [c] $\Delta\tilde{\nu} = \frac{1}{\lambda_{max,Abs}} - \frac{1}{\lambda_{max,Em}}$.

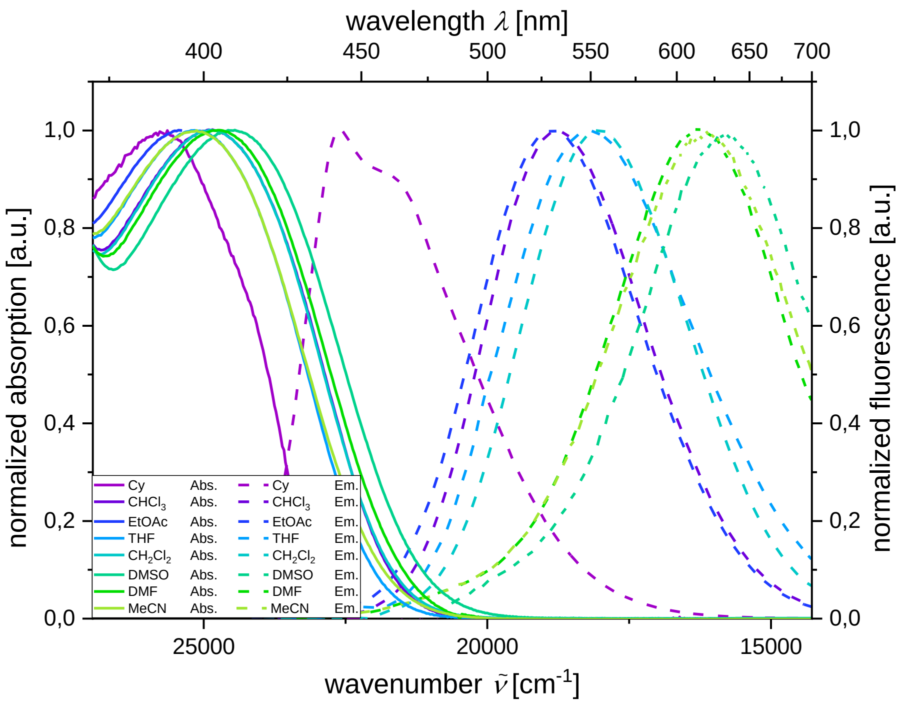

Figure 3. UV/Vis absorption (bold lines) and emission (dashed lines) spectra of compound **13e** measured in eight solvents of different polarity recorded at T = 293 K).

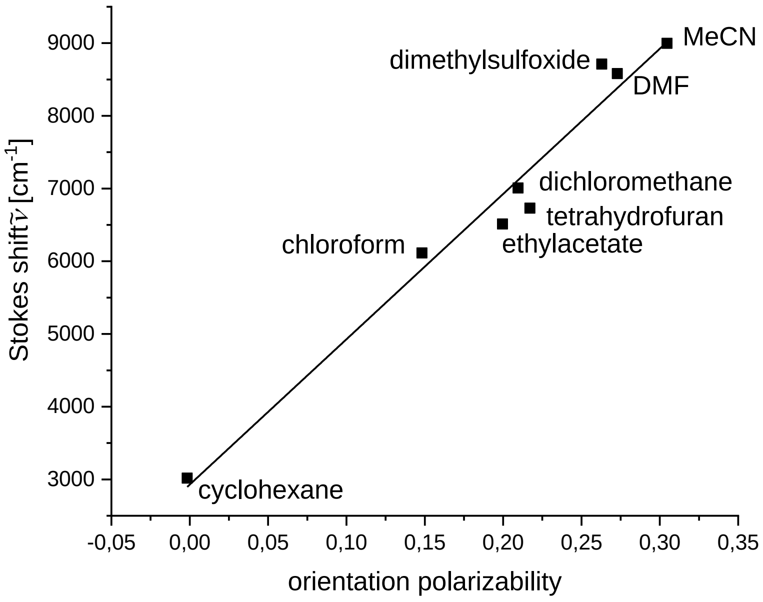

Figure 4. Lippert-Mataga plot for compound **13e** (n = 8, r^2 = 0.97).

5.3. Solvatochromicity of compound 15e

Table 8: Absorption and emission maxima of compound **15e** measured in different solvents.

solvent	refractive index[4] $n_D{}^{20}$	permittivity[4] ε_r	orientation polarizability Δf	$\lambda_{\text{max,Abs}}$[a] [nm]	$\lambda_{\text{max,Em}}$[b] [nm]	$\Delta\tilde{\nu}$ [c] [cm^{-1}]
**cyclohexane**	1.4262	2.02	-0.0016497	392	499	5500
**toluene**	1.4969	2.38	0.013235	396	525	6200
**chloroform**	1.4459	4.81	0.14826	402	577	7500
**ethyl acetate**	1.3724	6.02	0.19964	393	572	8000
**dichloromethane**	1.4242	8.93	0.21710	403	609	8400
**dmf**	1.4305	36.71	0,27286	407	662	9500
**dmso**	1.4793	46.45	0.26301	413	684	9600
**acetonitrile**	1.3441	35.94	0.30457	400	673	10100

[a] Recorded in different solvents, T = 293 K, c = 10^{-5} M. [b] Recorded in different solvents, T = 293 K, c = 10^{-7} M. [c] $\Delta\tilde{\nu} = \frac{1}{\lambda_{max,Abs}} - \frac{1}{\lambda_{max,Em}}$.

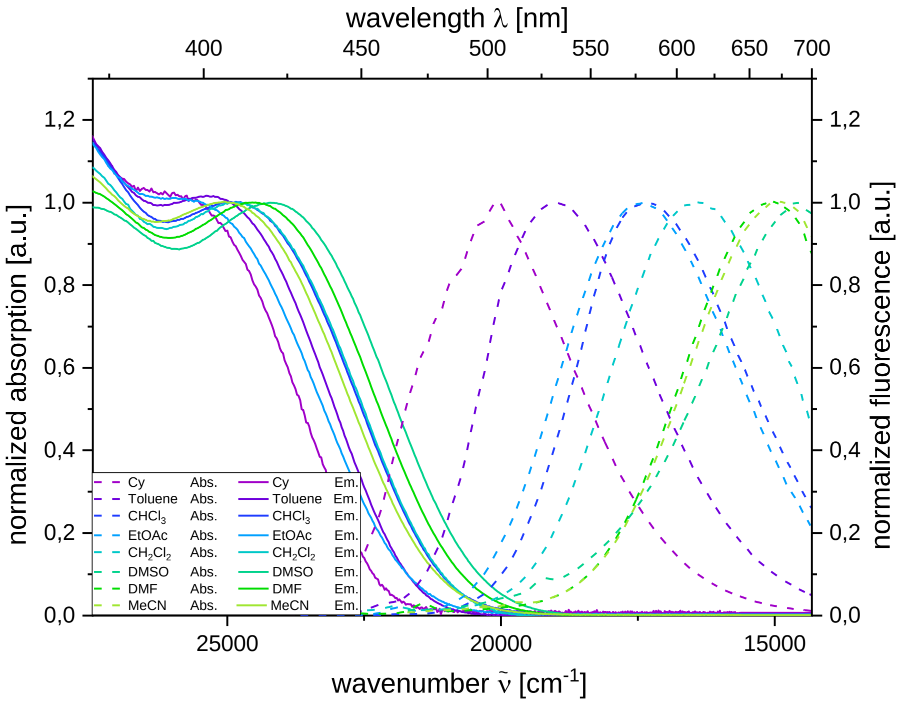

Figure 5. UV/Vis absorption (bold lines) and emission (dashed lines) spectra of compound **15e** measured in eight solvents of different polarity recorded at T = 293 K).

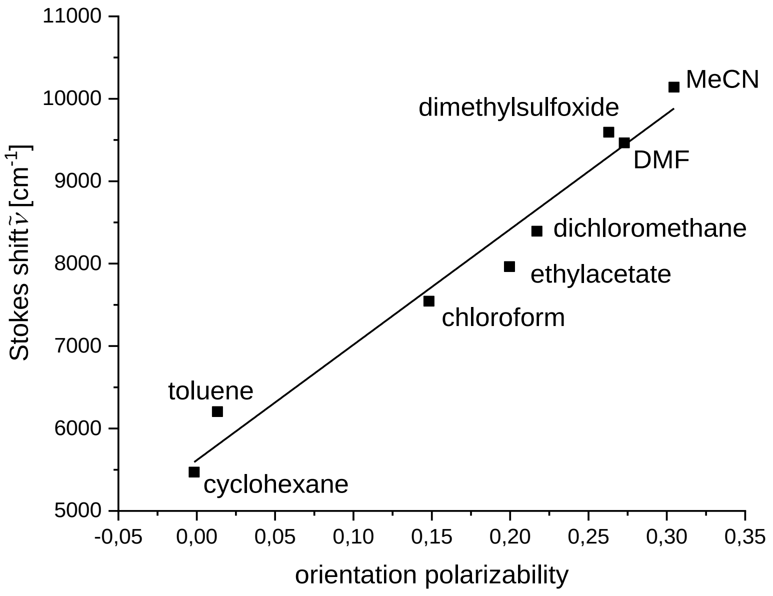

Figure 6. Lippert-Mataga plot for compound **15e** (n = 8, r^2 = 0.98).

6. Acidochromicity

6.1. Absorption spectra of non-protonated and protonated compounds

6.1.1. Absorption spectra of compound 11e

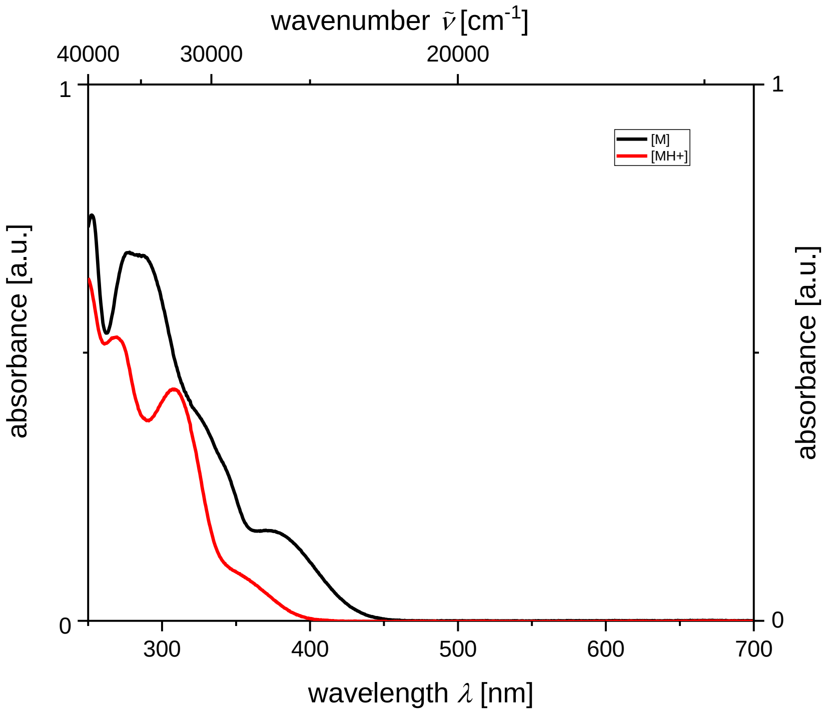

6.1.2. Absorption spectra of compound 13e

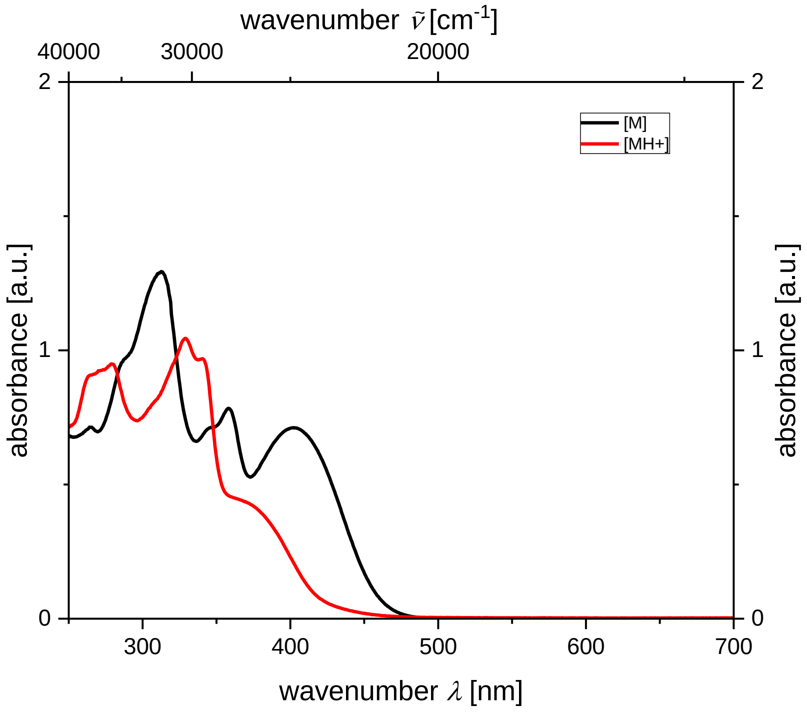

6.1.3. Absorption spectra of compound 15e

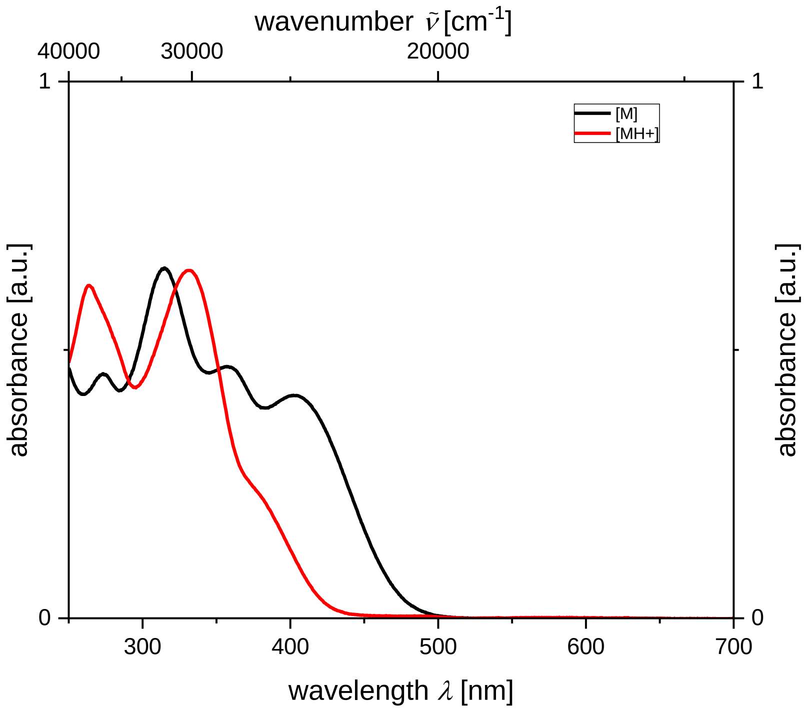

6.2. Determination of pK_a value

By absorption spectroscopy:

To determine the pK_a value by absorption spectroscopy, a stock solution with a suitable concentration is prepared first. Then ten test solutions are prepared in 10 mL volumetric flasks. For the blank sample, 1.5 mL is taken from the stock solution and made up to the volume of the 10 mL volumetric flask (in these examples the solvent is CH_2Cl_2). For the other test solutions, in addition to 1.5 mL of the stock solution, different amounts (0.5-8.5 mL) of a TFA stock solution (c(TFA) = 0.005192 M) are added.

By means of a difference spectrum ($\Delta E = E - E_0$) the absorption maxima of the non-protonated species M and the protonated species M+H$^+$ can be determined. From the obtained raw data, corrected measurement data are calculated. The corrected measurements of the protonated species are generated by subtracting the measurement value E_0 (no addition of TFA). In contrast, the measured values of the non-protonated species are corrected by the measured value of the fully protonated species. At the intersection of both titration curves is c(M) = c(M+H$^+$), which corresponds to the half equivalence point. At the half-equivalence point of a strong acid pH = pK_a applies.

6.2.1. Titration Experiment (Absorption) of compound 11e

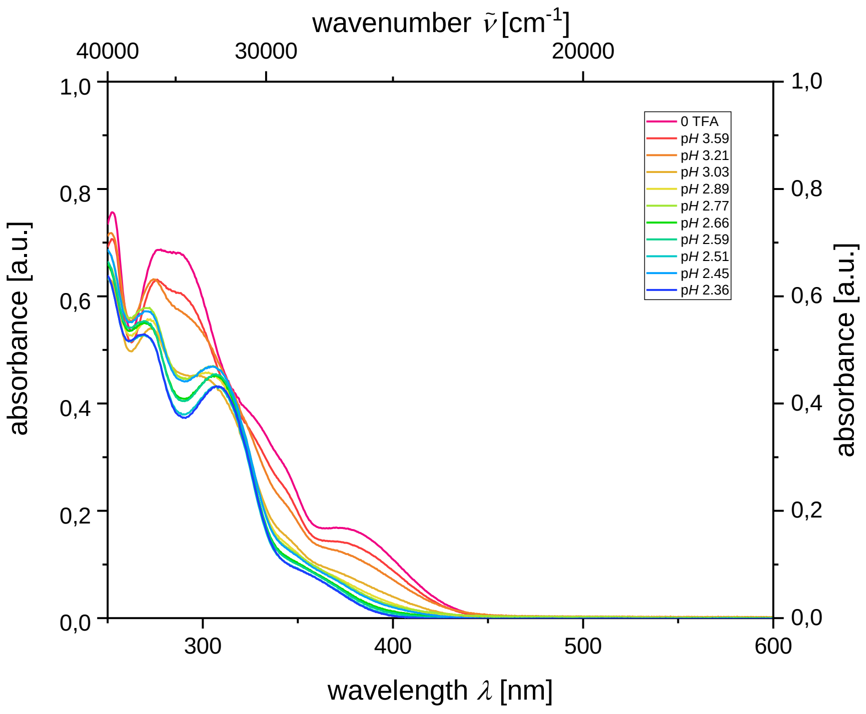

Figure 7. Absorption spectra of **11e** in the presence of increasing amounts of TFA (recorded in CH_2Cl_2, c(**11e**) = 0.000186 M, T = 293 K).

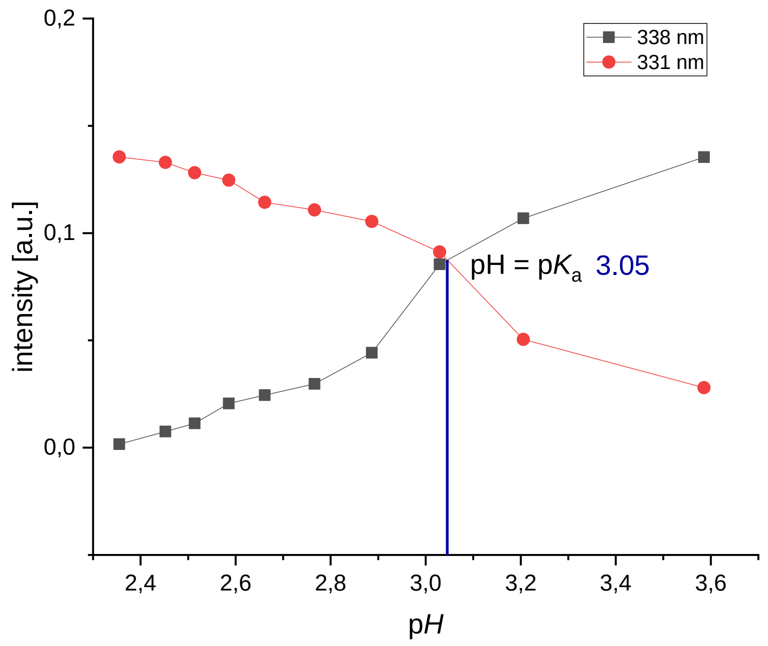

Figure 8. Determination of the p*K*a of **11e** by plotting the absorption intensity at 383 and 331 nm against p*H*-value.

6.2.2. Titration Experiment (Absorption) of compound 13e

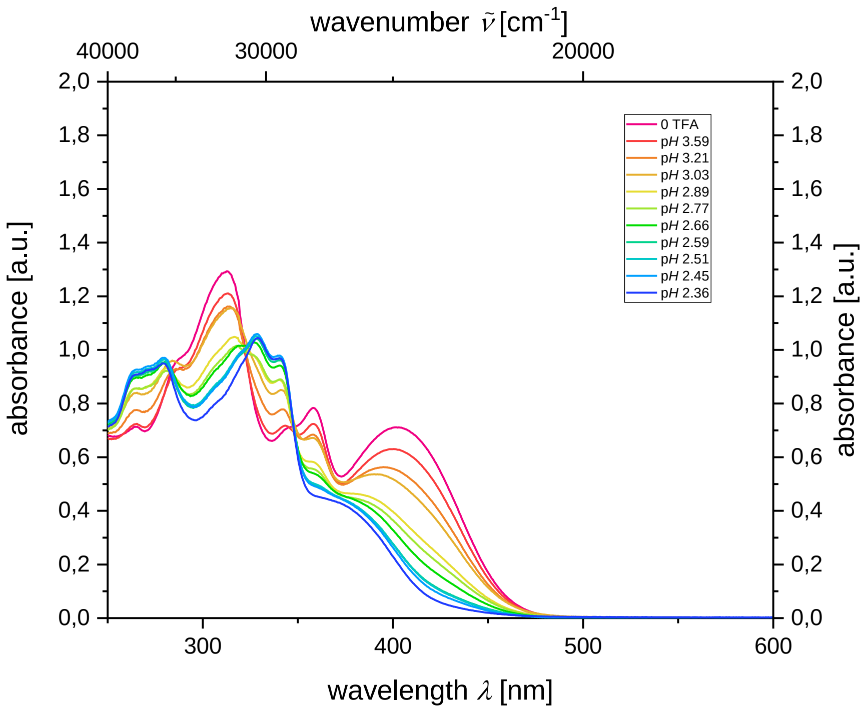

Figure 9. Absorption spectra of **13e** in the presence of increasing amounts of TFA (recorded in CH_2Cl_2, c(**13e**) = 0.000278 M, T = 293 K).

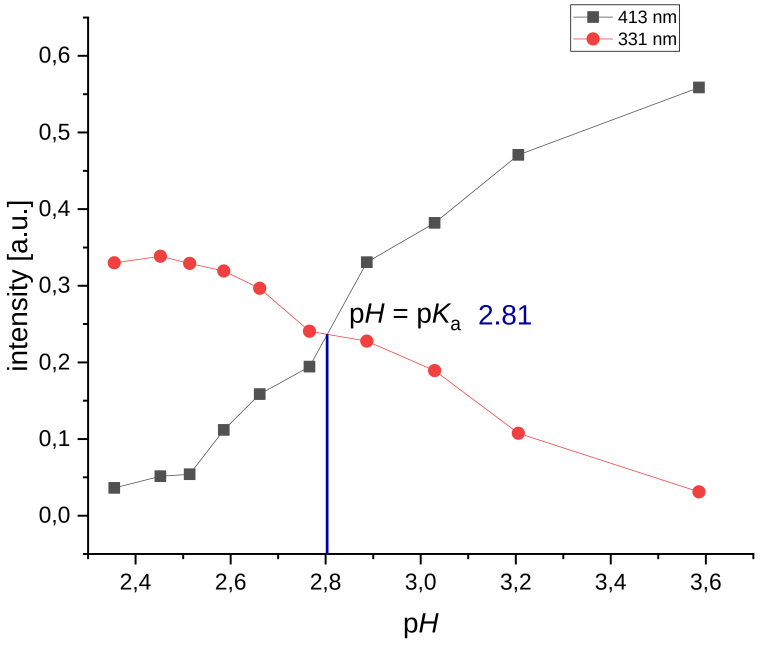

Figure 10. Determination of the p*K*a of **13e** by plotting the absorption intensity at 331 and 413 nm against p*H*-value.

6.2.3. Titration Experiment (Absorption) of compound 15e

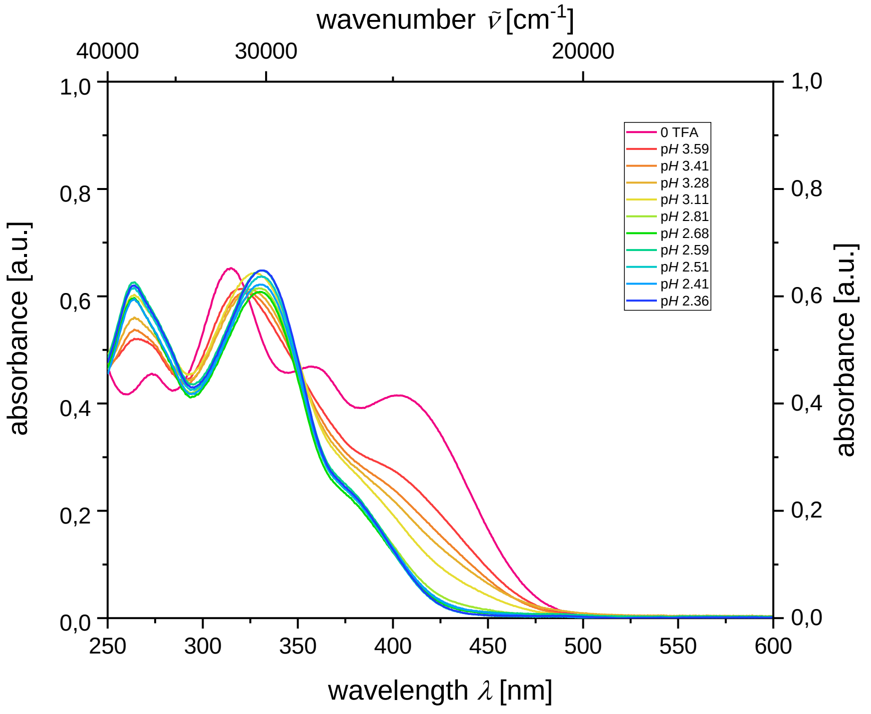

Figure 11. Absorption spectra of **15e** in the presence of increasing amounts of TFA (recorded in CH_2Cl_2, *c*(**15e**) = 0.000175 M, *T* = 293 K).

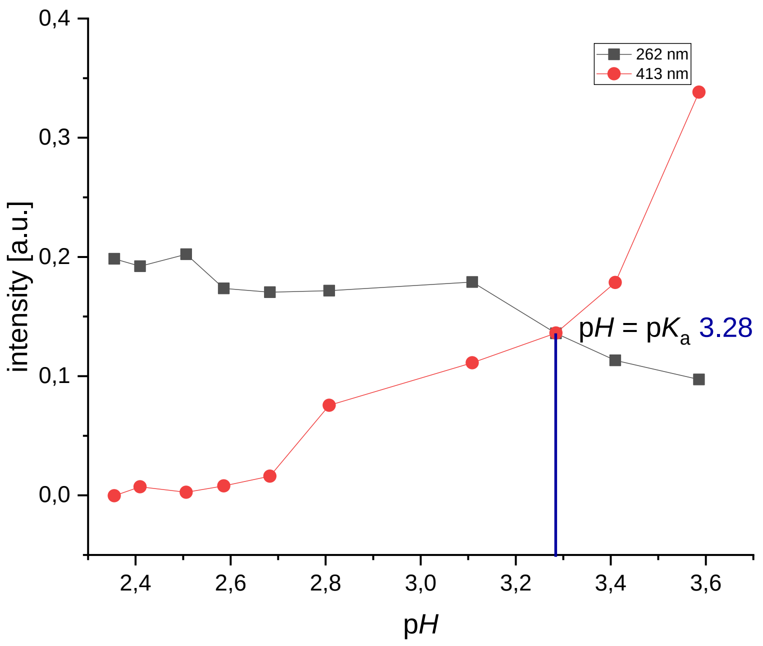

Figure 12. Determination of the p*K*a of **15e** by plotting the absorption intensity at 413 and 262 nm against p*H*-value.

6.2.4. Titration Experiment (Fluorescence Quenching) of compound 11e

Determination of the pK_a value by emission spectroscopy:

For quantification, 1.63 mg of **11e** are weighed and placed in a 50 mL volumetric flask dissolved in CH_2Cl_2 UVASOL®. For the measurement the sample must be diluted further and mixed with different amounts of TFA. For this purpose, 0.1 mL of the stock solution and varying volumes of a TFA solution (c(TFA) = 0.01754 M) are diluted in a 10 mL volumetric flask.

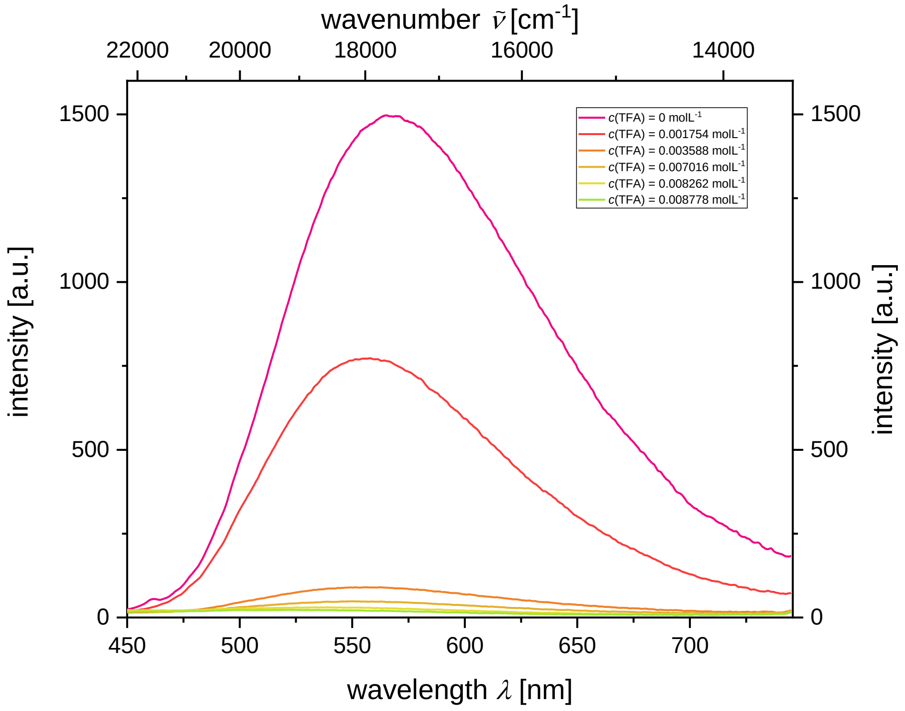

Figure 13. Emission spectra of the titration of compound **11e** (c = 9.07*10^{-5} M) with TFA in dichloromethane (recorded at T = 293 K, λ_{exc} = 380 nm).

Without the addition of the quencher (TFA), the measured substance has an area of emission maximum of 723. To determine the Stern-Volmer constant, $F_0 \cdot F^{-1}$(ordinate) has to be plotted in relation to the concentration of the quencher (abscissa). The result is a line with the line equation: F_0/F = 5351[H^+] + 1.9052. From the line equation the Stern-Volmer constant K_{SV} (slope) can be obtained. To determine the pK_a value of the substance, the concentration of protons [H^+] must be valid:

$$\frac{F_0}{F} = 0$$

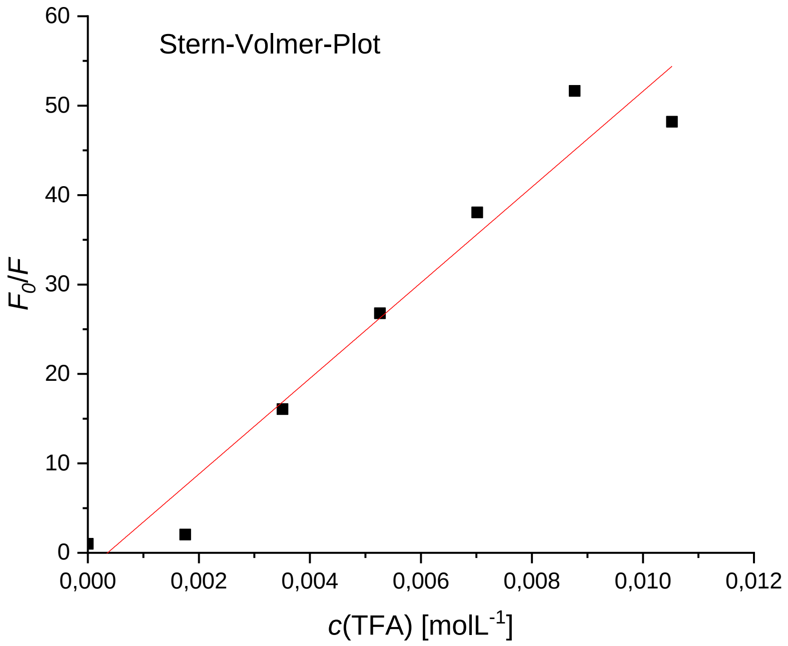

Figure 14: Stern-Volmer plot of compound **11e** (*c* = 9.07*10^{-5} M; $F_0/F = 5351[H^+] + 1.9052$ ($r^2 = 0.951$).

$$\frac{F_0}{F} = 0$$

$$[H]^+ = \frac{1.9052}{5351} = 0.000356046$$

$$pH = -\log[H]^+$$

$$= 3.45 = pKa$$

6.2.5. Titration Experiment (Fluorescence Quenching) of compound 13e

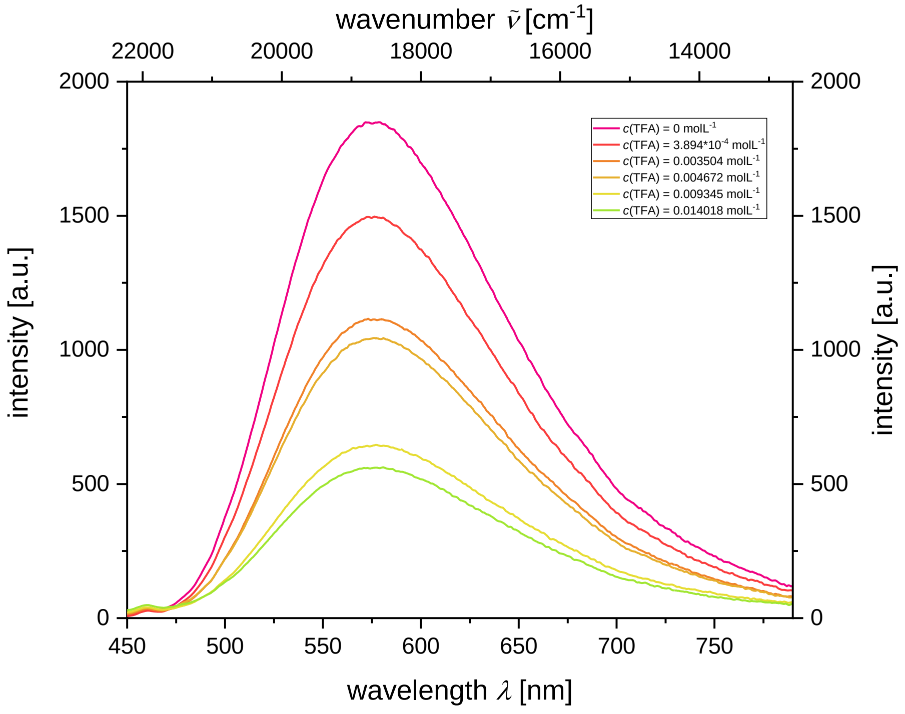

Figure 15. Emission spectra of the titration of compound **13e** (*c* = 0.000111 M) with TFA in dichloromethane (recorded at *T* = 293 K, λ_{exc} = 403 nm).

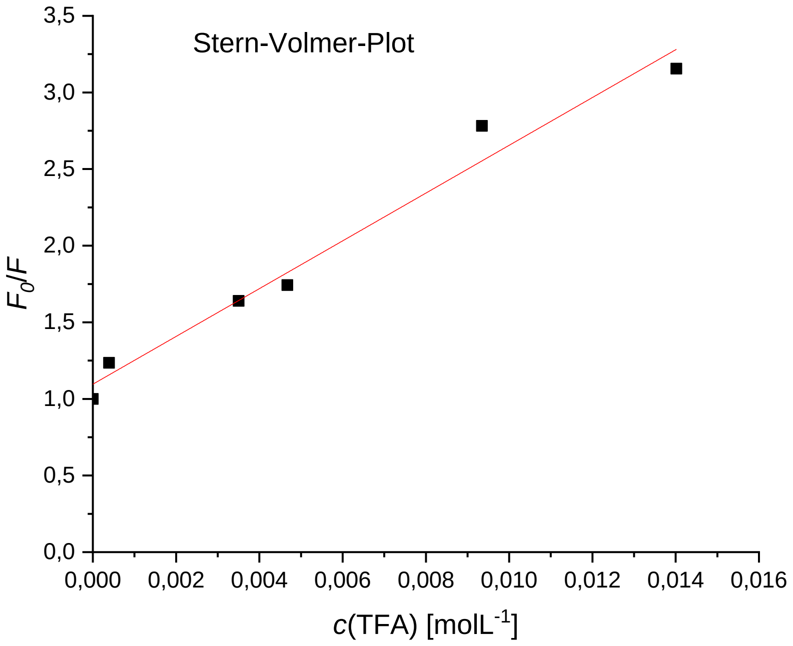

Figure 16. Stern-Volmer plot of compound **13e** (*c* = 0.000111 M; $F_0/F = 155.93[H^+] + 1.0959$ ($r^2 = 0.976$).

$$\frac{F_0}{F} = 0$$

$$[H]^{+} = \frac{1.0959}{155.93} = 0.00702815$$

$$pH = -\log[H]^{+}$$

$$= 2.15 = pKa$$

6.2.6. Titration Experiment (Fluorescence Quenching) of compound 15e

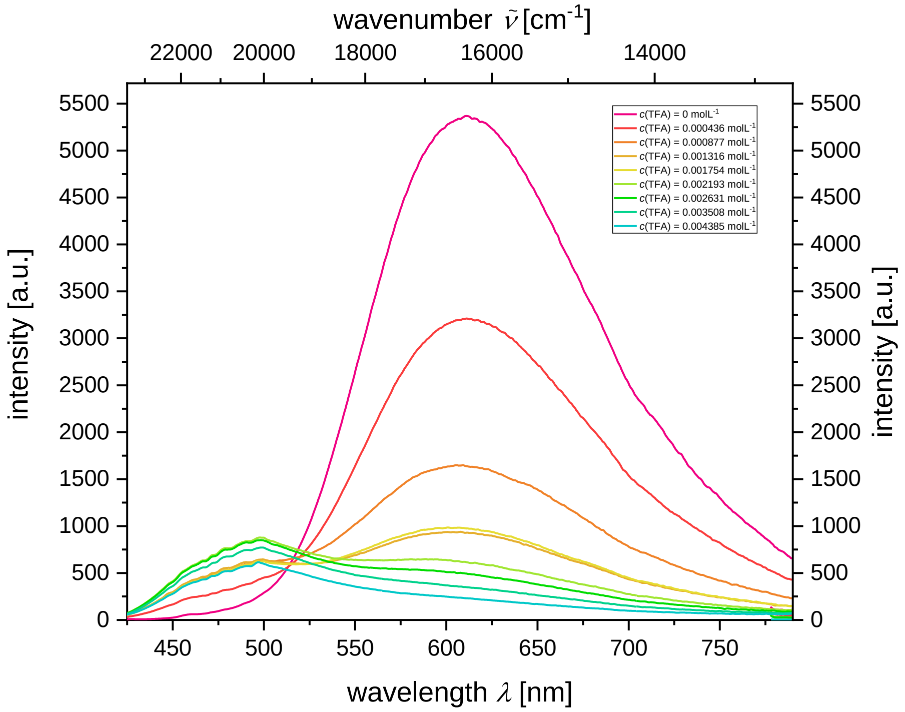

Figure 17. Emission spectra of the titration of compound **15e** (c = 0.000176 M) with TFA in dichloromethane (recorded at T = 293 K, λ_{exc} = 402 nm).

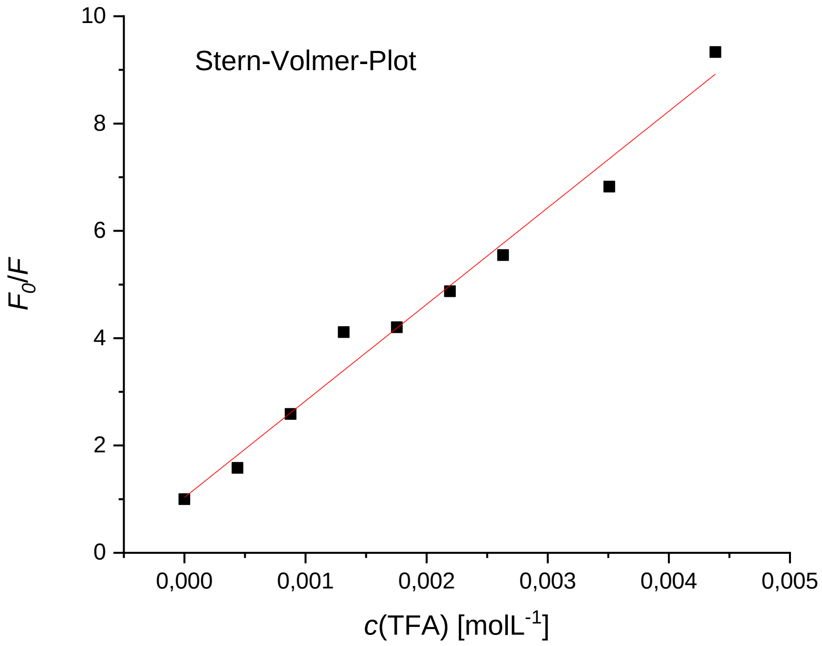

Figure 18. Stern-Volmer plot of compound **15e** (c = 0. 000176 M; $F_0/F = 1799.5[H^+] + 1.0316$ ($r^2 = 0.981$).

$$\frac{F_0}{F} = 0$$

$$[H]^+ = \frac{1.0316}{1799.5} = 0.00048529$$

$$pH = -\log[H]^+$$

$$= 3.31 = pKa$$

7. X-ray

7.1. X-ray structural data of compound 11a

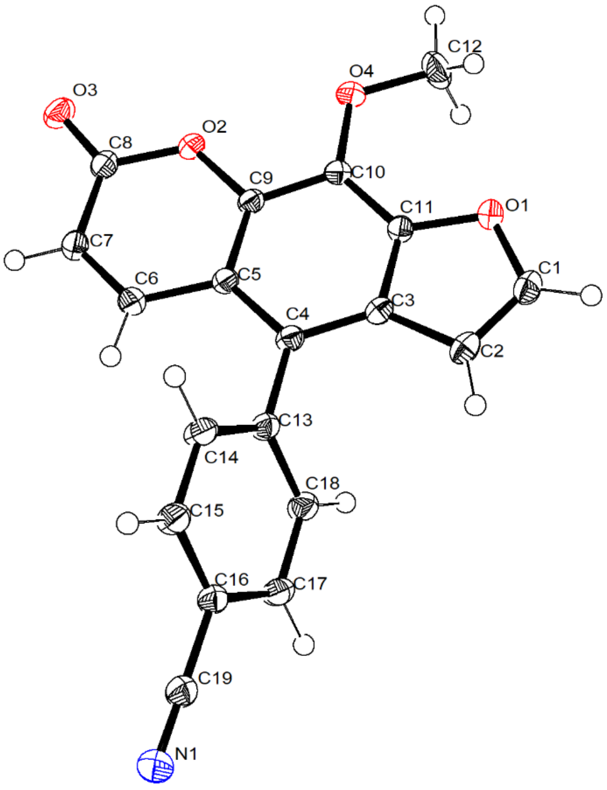

Figure 19. X-ray structure of compound **11a** in the crystal.

Molecular formula	$C_{19}H_{11}NO_4$	
Molecular weight [g/mol]	317.29	
Crystal description and color	Block, brown	
Crystal size [mm]	0.7x0.5x0.3	
Temperature [K]	140	
Crystal system	monoclinic	
Space group	$P2_1/c$	
Unit cell dimensions	a = 10.2786(5) Å	
	b = 11.3089(6) Å	β = 105.193(2)°
	c = 13.0480(7) Å	
Volume of the unit cell [Å]	1463.68(13)	
Formula unit's Z	4	
Calculated density [g/mL]	1.440	
Absorption coefficient [mm^{-1}]	0.102	
F (000)	656	
Range for data collection Θ [°]	2.4205 – 36.9275	
Index ranges	-14 ≤ h ≤ 14	

$-15 \le k \le 15$	
$-18 \le l \le 18$	
Reflections collected	33692
Independent reflections	4272
Observed reflections ($I>2\sigma(I)$)	3887
Final R indices ($I>2\sigma(I)$)[a]	$R_1 = 0.0405$
	$wR_2 = 0.1137$
R indices (all data)[a]	$R_1 = 0.0436$
	$wR_2 = 0.1172$
Completeness	99.9%
Data/restraints/parameters	4272/0/218
Goodness-of-fit-on F^2[b]	1.040

[a] $R1 = \Sigma||F_o|-|F_c||/\Sigma|F_o|$; $wR_2 = [\Sigma[w(F_o^2-F_c^2)^2/ \Sigma[(wF_o^2)^2]]^{1/2}$; $w = 1/[\sigma^2(F_o^2)+(aP)^2+bP]$ mit $P = (F_o^2+2F_c^2)/3$

[b] GooF = $S = [[\Sigma w(F_o^2-F_c^2)^2]/(m-n)]^{1/2}$, m = number of reflections, n = number of parameters

7.2. X-ray structural data of compound 13e

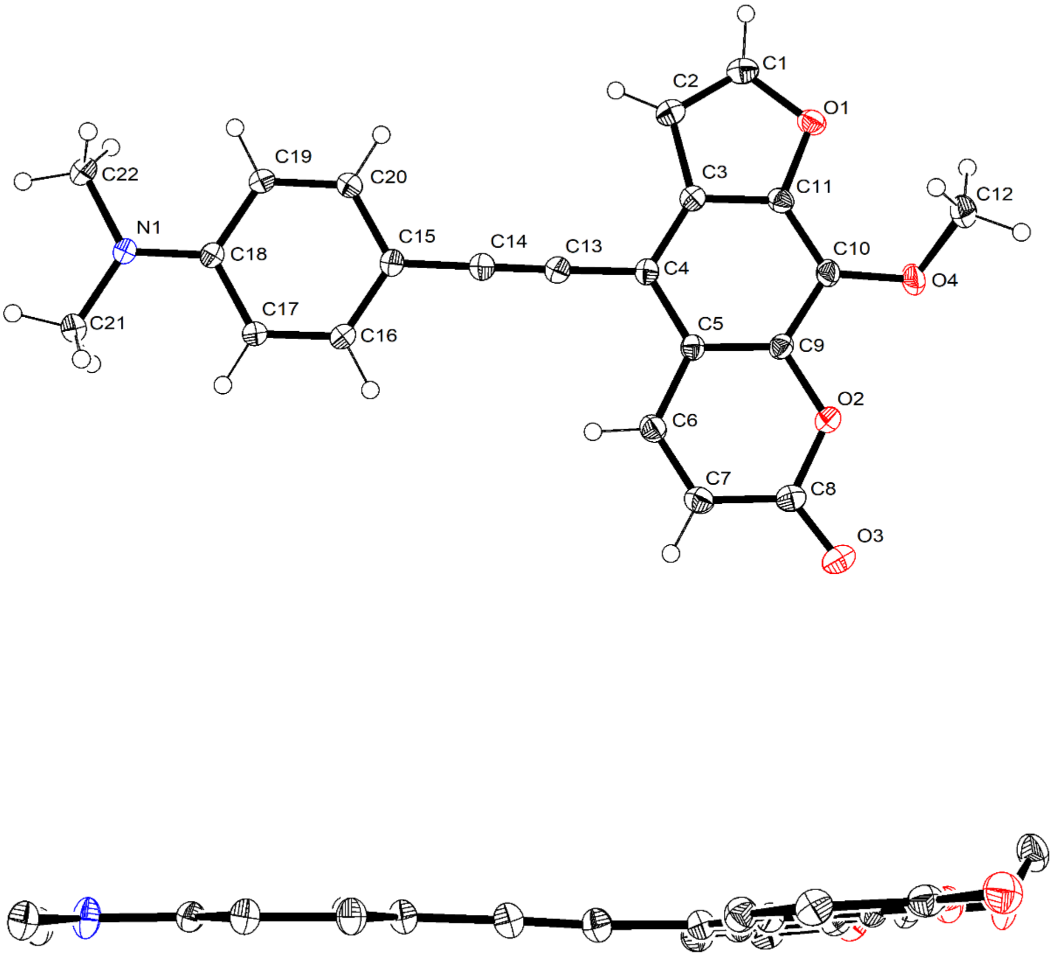

Figure 20. X-ray structure of compound in the crystal.

Molecular formula	$C_{22}H_{17}NO_4$	
Molecular weight [g/mol]	359.39	
Crystal description and color	Block, yellow	
Crystal size [mm]	0.8x0.5x0.3	
Temperature [K]	140	
Crystal system	monocline	
Space group	$P2_1/c$	
Unit cell dimensions	a = 14.6860(6) Å	
	b = 6.0575(3) Å	β = 95.485(2)°
	c = 19.9785(8) Å	
Volume of the unit cell [Å]	1769.16(13)	

Formula unit's Z	4
Calculated density [g/mL]	1.349
Absorption coefficient [mm^{-1}]	0.093
F (000)	752
Range for data collection Θ [°]	2.3645 – 32.742
Index ranges	-20 ≤ h ≤ 20
	-8≤ k ≤ 8
	-28 ≤ l ≤ 26
Reflections collected	33218
Independent reflections	5165
Observed reflections (I>2σ(I))	4393
Final R indices (I>2σ(I))[a]	R_1 = 0.0438
	wR_2 = 0.1202
R indices (all data)[a]	R_1 = 0.0513
	wR_2 = 0.1272
Completeness	99.9%
Ind. Refl./Limitat./Parameter	5165/0/247
Goodness-of-fit-on F^2[b]	1.049

[a] $R1 = \Sigma||F_o|-|F_c||/\Sigma|F_o|$; $wR_2 = [\Sigma[w(F_o^2-F_c^2)^2/\ \Sigma[(wF_o^2)^2]]^{1/2}$; $w = 1/[\sigma^2(F_o^2)+(aP)^2+bP]$ mit $P = (F_o^2+2F_c^2)/3$

[b] GooF = $S = [[\Sigma w(F_o^2-F_c^2)^2]/(m-n)]^{1/2}$, m = number of reflections, n = number of parameters

8. Computed xyz-coordinates and computed UV/Vis spectra of TD-DFT calculated structures

8.1. XYZ-coordinates of the S_0 state of compound 8 (PBE1PBE/6-311G(d,p)) and TD-DFT calculation (PBE1PBE/6-21G, SCRF(IEFPCM, DCM))

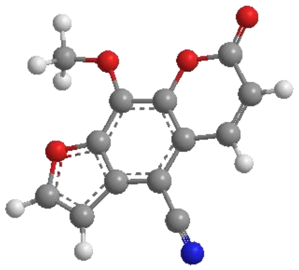

O -2.011985 -1.070811 0.000041
C -3.223224 -0.417003 -0.000014
O -4.219500 -1.091194 -0.000086
C -3.165653 1.031710 0.000015
C -1.991236 1.687890 0.000032
C -0.745760 0.971703 0.000025
C 0.512459 1.608726 0.000010
C 1.656445 0.807852 -0.000006
C 3.065116 1.071557 -0.000028
C 3.665300 -0.136495 -0.000020
O 2.768350 -1.161241 -0.000008
C 1.536032 -0.592562 0.000003
C 0.314499 -1.261998 0.000018
C -0.820149 -0.425255 0.000027
C 0.618041 3.021850 0.000001
N 0.712885 4.174496 -0.000014
O 0.084293 -2.571703 0.000023
C 1.175194 -3.494043 -0.000001
H -4.117116 1.547207 0.000009
H -1.967748 2.773069 0.000048
H 3.552847 2.033723 -0.000050
H 4.701364 -0.436072 -0.000025
H 0.712245 -4.478328 -0.000027
H 1.788284 -3.372512 -0.894438
H 1.788279 -3.372559 0.894448
SCF Done: E(RPBE1PBE) = -854.418601973 A.U. after 14 cycles
E(PBE1PBE) = -2243276.21036383 kJ/mol

Sum of electronic and zero-point Energies=   -854.287055

Sum of electronic and thermal Energies=   -854.272867

Sum of electronic and thermal Enthalpies=   -854.271923

Sum of electronic and thermal Free Energies=   -854.328755

HOMO -6.573 eV

LUMO -2.374 eV

Relevant excitation energies and oscillator strengths:

Excited State 1: Singlet-A 3.5743 eV 346.88 nm f=0.1227 <S**2>=0.000

62 -> 63   0.68754

62 -> 64   0.10827

This state for optimization and/or second-order correction.

Total Energy, E(TD-HF/TD-KS) = -853.128827252

Copying the excited state density for this state as the 1-particle RhoCI density.

Excited State 2: Singlet-A 4.3032 eV 288.12 nm f=0.3003 <S**2>=0.000

61 -> 63   0.65944

62 -> 64   0.16745

62 -> 65 -0.15745

Excited State 3: Singlet-A 4.5369 eV 273.28 nm f=0.0003 <S**2>=0.000

60 -> 63   0.67136

60 -> 64   0.14749

60 -> 65   0.14821

Excited State 4: Singlet-A 4.8396 eV 256.19 nm f=0.4567 <S**2>=0.000

61 -> 63 -0.16591

62 -> 64   0.66426

Excited State 5: Singlet-A 5.2357 eV 236.81 nm f=0.1135 <S**2>=0.000

59 -> 63   0.16661

61 -> 64   0.58471

62 -> 65   0.32801

Excited State 6: Singlet-A 5.2634 eV 235.56 nm f=0.0035 <S**2>=0.000

59 -> 63   0.66889

61 -> 64 -0.19656

Excited State 7: Singlet-A 5.6627 eV 218.95 nm f=0.5246 <S**2>=0.000

59 -> 63 -0.11030

61 -> 63   0.14831

61 -> 64 -0.31012

62 -> 65   0.58880

Excited State 8: Singlet-A 5.9651 eV 207.85 nm f=0.0000 <S**2>=0.000

61 -> 66 -0.12479

62 -> 66   0.67969

Excited State 9: Singlet-A 6.1351 eV 202.09 nm f=0.0272 <S**2>=0.000

58 -> 63   0.55383

59 -> 64   0.17989

61 -> 65 -0.36907

Excited State 10: Singlet-A 6.1530 eV 201.50 nm f=0.0003 <S**2>=0.000

60 -> 63 -0.18470

60 -> 64   0.65572

60 -> 65   0.16751

Excited State 11: Singlet-A 6.3603 eV 194.93 nm f=0.1629 <S**2>=0.000

58 -> 63 -0.11576

59 -> 64   0.63299

61 -> 65   0.17534

62 -> 67   0.16231

Excited State 12: Singlet-A 6.4411 eV 192.49 nm f=0.0012 <S**2>=0.000

52 -> 63 -0.11458

53 -> 63   0.19201

57 -> 63   0.49056

60 -> 64 -0.13669

60 -> 65   0.39554

60 -> 67   0.10280

Excited State 13: Singlet-A 6.4456 eV 192.35 nm f=0.0931 <S**2>=0.000

54 -> 63   0.15817

56 -> 63   0.21552

58 -> 63   0.36784

59 -> 64 -0.13794

61 -> 65   0.49278

Excited State 14: Singlet-A 6.5225 eV 190.09 nm f=0.0000 <S**2>=0.000

57 -> 63   0.48210

60 -> 64   0.14112

60 -> 65 -0.45114

60 -> 67 -0.11093

Excited State 15: Singlet-A 6.5777 eV 188.49 nm f=0.0000 <S**2>=0.000

55 -> 63   0.67799

55 -> 64 -0.11840

Excited State 16: Singlet-A 6.6491 eV 186.47 nm f=0.0698 <S**2>=0.000

59 -> 64 -0.16050

62 -> 67   0.65821

Excited State 17: Singlet-A 6.8678 eV 180.53 nm f=0.0013 <S**2>=0.000

52 -> 63 -0.25578

53 -> 63   0.55164

60 -> 65 -0.19687

62 -> 68   0.20563

Excited State 18: Singlet-A 6.9249 eV 179.04 nm f=0.0722 <S**2>=0.000

54 -> 63 -0.27339

56 -> 63   0.60800

61 -> 65 -0.12786

Excited State 19: Singlet-A 6.9250 eV 179.04 nm f=0.0005 <S**2>=0.000

53 -> 63 -0.10848

61 -> 66   0.67066

62 -> 66   0.13137

Excited State 20: Singlet-A 6.9909 eV 177.35 nm f=0.0047 <S**2>=0.000

53 -> 63 -0.15972

61 -> 66 -0.11699

62 -> 68   0.66356

Excited State 21: Singlet-A 7.0448 eV 175.99 nm f=0.0348 <S**2>=0.000

54 -> 63   0.57138

56 -> 63   0.17893

59 -> 65   0.22053

61 -> 65 -0.19896

61 -> 67   0.16573

Excited State 22: Singlet-A 7.2192 eV 171.74 nm f=0.0805 <S**2>=0.000

58 -> 64 -0.38946

59 -> 65 -0.21810
61 -> 67   0.51937

Excited State 23: Singlet-A 7.3432 eV 168.84 nm f=0.0039 <S**2>=0.000
52 -> 63   0.59967
53 -> 63   0.32125
53 -> 64 -0.11111
57 -> 64 -0.12476

Excited State 24: Singlet-A 7.3716 eV 168.19 nm f=0.0487 <S**2>=0.000
54 -> 63 -0.13850
58 -> 64 -0.38553
59 -> 65   0.55143

8.2. XYZ-coordinates of the S_0 state of compound 9 (PBE1PBE/6-311G(d,p)) and TD-DFT calculation (PBE1PBE/6-21G, SCRF(IEFPCM, DCM)

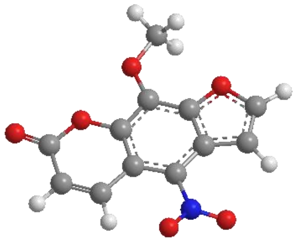

O -2.247356 0.943957 0.000280
C -3.329928 0.094508 -0.000219
O -4.425921 0.589701 -0.000594
C -3.030961 -1.325047 -0.000153
C -1.765047 -1.777798 0.000121
C -0.658155 -0.859879 0.000213
C 0.680561 -1.245660 0.000139
C 1.686617 -0.299631 0.000077
C 3.122558 -0.326742 -0.000134
C 3.510345 0.963660 -0.000256
O 2.453997 1.825299 -0.000147
C 1.331587 1.063571 0.000043
C 0.015536 1.521699 0.000095
C -0.962443 0.511826 0.000185
N 1.024323 -2.672806 0.000055
O 1.155207 -3.210870 -1.078012

O 1.153888 -3.211329 1.078053
O -0.428068 2.780790 0.000036
C 0.500093 3.864901 0.000278
H -3.883210 -1.991718 -0.000344
H -1.572912 -2.845886 0.000253
H 3.767991 -1.191053 -0.000166
H 4.481898 1.431578 -0.000416
H -0.114040 4.762927 0.000413
H 1.125688 3.845315 0.894533
H 1.125775 3.845639 -0.893922
SCF Done: E(RPBE1PBE) = -966.601202192 A.U. after 15 cycles
E(PBE1PBE) = -2537811.649675336 kJ/mol
Sum of electronic and zero-point Energies= -966.459693
Sum of electronic and thermal Energies= -966.445248
Sum of electronic and thermal Enthalpies= -966.444303
Sum of electronic and thermal Free Energies= -966.502000
HOMO -6.537 eV
LUMO -2.290 eV

Relevant excitation energies and oscillator strengths:
Excited State 1: Singlet-A 3.3031 eV 375.36 nm f=0.0000 <S**2>=0.000
63 -> 69 -0.20610
64 -> 69 -0.10203
66 -> 69 -0.12974
67 -> 69 0.65470
This state for optimization and/or second-order correction.
Total Energy, E(TD-HF/TD-KS) = -965.054540999
Copying the excited state density for this state as the 1-particle RhoCI density.

Excited State 2: Singlet-A 3.6236 eV 342.16 nm f=0.0572 <S**2>=0.000
67 -> 68 0.69647

Excited State 3: Singlet-A 4.2398 eV 292.43 nm f=0.0000 <S**2>=0.000
63 -> 69 0.31690
66 -> 69 0.56987
67 -> 69 0.23260

Excited State 4: Singlet-A 4.3609 eV 284.31 nm f=0.0015 <S**2>=0.000

62 -> 69   0.69689

Excited State 5: Singlet-A 4.4440 eV 278.99 nm f=0.2189 <S**2>=0.000

66 -> 68   0.64300

67 -> 70 -0.23710

67 -> 71   0.14533

Excited State 6: Singlet-A 4.5868 eV 270.30 nm f=0.0000 <S**2>=0.000

61 -> 69 -0.10777

63 -> 69   0.52968

64 -> 69   0.15223

66 -> 69 -0.39607

67 -> 69   0.11866

Excited State 7: Singlet-A 4.6096 eV 268.97 nm f=0.0004 <S**2>=0.000

65 -> 68   0.68333

65 -> 71 -0.16077

Excited State 8: Singlet-A 5.0445 eV 245.78 nm f=0.6449 <S**2>=0.000

66 -> 68   0.24699

66 -> 70 -0.14537

67 -> 70   0.61870

Excited State 9: Singlet-A 5.3904 eV 230.01 nm f=0.0030 <S**2>=0.000

64 -> 68 -0.20020

66 -> 70   0.49520

67 -> 70   0.18221

67 -> 71   0.41122

Excited State 10: Singlet-A 5.4555 eV 227.26 nm f=0.0027 <S**2>=0.000

64 -> 68   0.66191

66 -> 70   0.17772

67 -> 71   0.11994

Excited State 11: Singlet-A 5.5429 eV 223.68 nm f=0.0000 <S**2>=0.000

63 -> 69 -0.20322

64 -> 69 0.67244

Excited State 12: Singlet-A 5.5861 eV 221.95 nm f=0.0009 <S**2>=0.000
61 -> 69 0.13696
62 -> 68 0.68425

Excited State 13: Singlet-A 5.6613 eV 219.00 nm f=0.0005 <S**2>=0.000
65 -> 69 0.70112

Excited State 14: Singlet-A 5.7345 eV 216.21 nm f=0.0405 <S**2>=0.000
63 -> 68 0.68631
67 -> 71 -0.10338

Excited State 15: Singlet-A 5.8479 eV 212.01 nm f=0.3257 <S**2>=0.000
61 -> 68 0.37172
63 -> 68 0.12195
66 -> 70 -0.35260
67 -> 71 0.43714

Excited State 16: Singlet-A 5.9624 eV 207.94 nm f=0.2308 <S**2>=0.000
60 -> 68 -0.10707
61 -> 68 0.59215
66 -> 70 0.21575
67 -> 71 -0.26817

Excited State 17: Singlet-A 6.3756 eV 194.47 nm f=0.0001 <S**2>=0.000
65 -> 70 0.64445
65 -> 71 -0.22386

Excited State 18: Singlet-A 6.4695 eV 191.64 nm f=0.1286 <S**2>=0.000
58 -> 68 0.10624
60 -> 68 0.38027
64 -> 70 -0.29551
66 -> 71 0.46429
67 -> 72 -0.15093

Excited State 19: Singlet-A 6.4951 eV 190.89 nm f=0.0700 <S**2>=0.000

60 -> 68   0.11457

64 -> 70   0.54515

66 -> 71   0.32907

67 -> 72   0.24485

Excited State 20: Singlet-A 6.5282 eV 189.92 nm f=0.0015 <S**2>=0.000

60 -> 69   0.64379

61 -> 69   0.21356

63 -> 69   0.15056

Excited State 21: Singlet-A 6.7040 eV 184.94 nm f=0.0011 <S**2>=0.000

57 -> 68   0.14598

59 -> 68   0.60292

60 -> 69 -0.10850

61 -> 69   0.11362

62 -> 70   0.23960

65 -> 70   0.10097

Excited State 22: Singlet-A 6.7465 eV 183.78 nm f=0.0012 <S**2>=0.000

57 -> 68   0.16247

62 -> 70 -0.10515

65 -> 68   0.16271

65 -> 70   0.24149

65 -> 71   0.58224

65 -> 74 -0.12521

67 -> 73 -0.10076

Excited State 23: Singlet-A 6.7627 eV 183.34 nm f=0.0398 <S**2>=0.000

63 -> 70 -0.24809

64 -> 70 -0.29531

67 -> 72   0.56994

Excited State 24: Singlet-A 6.7667 eV 183.23 nm f=0.0053 <S**2>=0.000

59 -> 68 -0.31599

60 -> 69 -0.12120

61 -> 69   0.15723

62 -> 70   0.57420

8.3. XYZ-coordinates of the S_0 state of compound 11a (PBE1PBE/6-311G(d,p)) and TD-DFT calculation (PBE1PBE/6-21G, SCRF(IEFPCM, DCM)

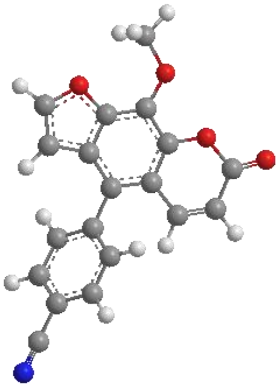

O -2.880136 1.740064 -0.032874

C -2.470100 3.048498 -0.111919

O -3.322006 3.900750 -0.109875

C -1.041471 3.261500 -0.201809

C -0.175014 2.231288 -0.168149

C -0.626718 0.871266 -0.041235

C 0.243266 -0.237028 -0.014998

C -0.348267 -1.500067 0.023169

C 0.168895 -2.842856 0.087947

C -0.903671 -3.655585 0.137954

O -2.078864 -2.962156 0.118425

C -1.748232 -1.647056 0.051963

C -2.635189 -0.574186 0.040427

C -2.021453 0.686521 -0.008800

C 1.714276 -0.097120 -0.008705

C 2.363587 0.637054 0.990668

C 3.743428 0.750073 1.007448

C 4.501788 0.120471 0.016061

C 3.867580 -0.621018 -0.985612

C 2.487233 -0.725802 -0.991496

C 5.923689 0.231877 0.027822

N 7.075074 0.322038 0.037281

O -3.973334 -0.592308 0.071449

C -4.667371 -1.837011 0.056493
H -0.715766 4.288401 -0.305042
H 0.889204 2.420774 -0.252667
H 1.202828 -3.149892 0.098186
H -1.007260 -4.727744 0.191149
H 1.781097 1.107903 1.775109
H 4.237098 1.315800 1.788828
H 4.457292 -1.104145 -1.755773
H 1.997269 -1.289408 -1.777808
H -5.723320 -1.573646 0.054620
H -4.429764 -2.409551 -0.842558
H -4.437003 -2.428488 0.944612
SCF Done: E(RPBE1PBE) = -1085.22773530 A.U. after 14 cycles

E(PBE1PBE) = -2849265.636075697 kJ/mol

Sum of electronic and zero-point Energies= -1085.037406

Sum of electronic and thermal Energies= -1085.018442

Sum of electronic and thermal Enthalpies= -1085.017498

Sum of electronic and thermal Free Energies= -1085.086071

HOMO -6.223 eV

LUMO -2.097 eV

Relevant excitation energies and oscillator strengths:

Excited State 1: Singlet-A 3.4892 eV 355.33 nm f=0.2066 <S**2>=0.000
82 -> 83 0.69434
This state for optimization and/or second-order correction.
Total Energy, E(TD-HF/TD-KS) = -1083.69683484
Copying the excited state density for this state as the 1-particle RhoCI density.

Excited State 2: Singlet-A 3.9645 eV 312.73 nm f=0.1376 <S**2>=0.000
81 -> 83 0.14720
82 -> 84 0.68119

Excited State 3: Singlet-A 4.2867 eV 289.23 nm f=0.2378 <S**2>=0.000
81 -> 83 0.65093
82 -> 84 -0.14522

82 -> 85 -0.14551
82 -> 86 -0.12290
82 -> 87   0.11523

Excited State 4: Singlet-A 4.6304 eV 267.76 nm f=0.0003 <S**2>=0.000
78 -> 83 -0.19619
78 -> 84 -0.11318
79 -> 83   0.48931
79 -> 84   0.22334
79 -> 87 -0.11404
80 -> 83   0.32711
80 -> 84   0.14512

Excited State 5: Singlet-A 4.7375 eV 261.71 nm f=0.0236 <S**2>=0.000
78 -> 83   0.12043
81 -> 84 -0.13481
82 -> 85   0.58624
82 -> 86 -0.30632

Excited State 6: Singlet-A 4.8059 eV 257.98 nm f=0.0434 <S**2>=0.000
81 -> 84   0.66981
82 -> 86 -0.18119

Excited State 7: Singlet-A 5.0431 eV 245.85 nm f=0.5175 <S**2>=0.000
81 -> 83   0.18702
81 -> 84   0.13513
81 -> 85 -0.11533
81 -> 86 -0.10615
82 -> 85   0.28833
82 -> 86   0.53887

Excited State 8: Singlet-A 5.2016 eV 238.36 nm f=0.1844 <S**2>=0.000
78 -> 83   0.24735
79 -> 83 -0.23720
79 -> 84 -0.10728
80 -> 83   0.56761
82 -> 85 -0.10824

Excited State 9: Singlet-A 5.2658 eV 235.45 nm f=0.0284 <S**2>=0.000
77 -> 83   0.13413
78 -> 83   0.48372
79 -> 83   0.31728
80 -> 83 -0.14821
80 -> 85 -0.15367
80 -> 86   0.11038
81 -> 85 -0.15267
82 -> 85 -0.10182
82 -> 86   0.14519

Excited State 10: Singlet-A 5.4091 eV 229.21 nm f=0.0186 <S**2>=0.000
77 -> 83   0.62934
78 -> 83 -0.18195
81 -> 85 -0.15792

Excited State 11: Singlet-A 5.4497 eV 227.51 nm f=0.0338 <S**2>=0.000
77 -> 83   0.21824
81 -> 85   0.45705
81 -> 86   0.19804
82 -> 86   0.14673
82 -> 87   0.39925

Excited State 12: Singlet-A 5.6176 eV 220.71 nm f=0.0313 <S**2>=0.000
77 -> 84 -0.10552
78 -> 83 -0.19646
78 -> 84 -0.20286
79 -> 84 -0.12464
81 -> 85 -0.34630
81 -> 86   0.45161
82 -> 87   0.21006

Excited State 13: Singlet-A 5.7164 eV 216.89 nm f=0.0137 <S**2>=0.000
77 -> 84   0.19550
78 -> 83   0.17971
78 -> 84   0.39547

79 -> 84   0.24820
80 -> 84   0.15003
80 -> 85   0.17887
80 -> 86 -0.14014
81 -> 85 -0.23742
81 -> 86   0.17955
82 -> 87   0.13230

Excited State 14: Singlet-A 5.8003 eV 213.75 nm f=0.3088 <S**2>=0.000
78 -> 84 -0.15108
79 -> 83 -0.12884
80 -> 84   0.54331
81 -> 86 -0.27954
82 -> 87   0.21681

Excited State 15: Singlet-A 5.8732 eV 211.10 nm f=0.1091 <S**2>=0.000
77 -> 84 -0.11929
78 -> 83   0.10545
78 -> 84 -0.21521
79 -> 83 -0.19454
79 -> 84   0.43191
80 -> 83 -0.13729
80 -> 84   0.14581
81 -> 85   0.12094
81 -> 86   0.22180
82 -> 87 -0.26778

Excited State 16: Singlet-A 5.9810 eV 207.30 nm f=0.1489 <S**2>=0.000
76 -> 83 -0.16431
77 -> 84 -0.17006
78 -> 84 -0.10123
79 -> 83 -0.12214
79 -> 84   0.34393
80 -> 84 -0.29995
81 -> 85 -0.12851
81 -> 86 -0.24186
82 -> 87   0.30581

Excited State 17: Singlet-A 6.0222 eV 205.88 nm f=0.1677 <S**2>=0.000
77 -> 84 0.60489
78 -> 84 -0.30819
80 -> 84 -0.10803

Excited State 18: Singlet-A 6.3611 eV 194.91 nm f=0.0878 <S**2>=0.000
76 -> 83 0.52721
80 -> 85 0.13165
80 -> 86 0.11357
81 -> 87 -0.31379
82 -> 89 0.13200

Excited State 19: Singlet-A 6.3982 eV 193.78 nm f=0.0052 <S**2>=0.000
72 -> 83 0.11168
75 -> 83 0.11561
76 -> 83 -0.19650
78 -> 85 -0.12150
79 -> 85 0.30965
79 -> 86 0.24909
79 -> 87 -0.18982
80 -> 85 0.32186
80 -> 86 0.22785
80 -> 87 -0.10329

Excited State 20: Singlet-A 6.4966 eV 190.84 nm f=0.0471 <S**2>=0.000
77 -> 85 0.19493
77 -> 86 0.12726
79 -> 85 -0.17216
79 -> 86 -0.10076
80 -> 85 0.16886
80 -> 86 0.15154
81 -> 87 0.24331
82 -> 88 0.35536
82 -> 89 0.36254

Excited State 21: Singlet-A 6.5360 eV 189.69 nm f=0.0453 <S**2>=0.000

72 -> 83   0.16869

75 -> 83   0.43407

79 -> 85   0.13715

80 -> 85 -0.32495

80 -> 86 -0.22414

81 -> 87   0.12876

82 -> 88   0.13048

82 -> 89   0.11646

Excited State 22: Singlet-A 6.5815 eV 188.38 nm f=0.0155 <S**2>=0.000

73 -> 83   0.12461

73 -> 84 -0.10794

75 -> 83 -0.26819

77 -> 85 -0.10656

79 -> 85   0.21925

79 -> 86   0.12327

80 -> 85 -0.12789

80 -> 86 -0.11063

80 -> 88 -0.12000

81 -> 87 -0.16605

82 -> 88   0.46101

Excited State 23: Singlet-A 6.6030 eV 187.77 nm f=0.0033 <S**2>=0.000

75 -> 83   0.36789

77 -> 85 -0.15098

77 -> 86 -0.10933

78 -> 85   0.11943

78 -> 86   0.12370

79 -> 85 -0.22344

79 -> 86 -0.14840

80 -> 85   0.13217

80 -> 86   0.14463

81 -> 87 -0.15245

82 -> 88   0.22533

82 -> 89 -0.21698

Excited State 24: Singlet-A 6.6883 eV 185.37 nm f=0.1758 <S**2>=0.000

76 -> 83   0.16396
77 -> 84 -0.10662
77 -> 85   0.16693
78 -> 84 -0.15550
78 -> 85   0.34655
78 -> 86   0.20626
79 -> 86   0.11200
80 -> 85   0.19108
80 -> 86 -0.20953
81 -> 87   0.23983
82 -> 89 -0.18265

8.4. XYZ-coordinates of the S_0 state of compound 11b (PBE1PBE/6-311G(d,p)) and TD-DFT calculation (PBE1PBE/6-21G, SCRF(IEFPCM, DCM)

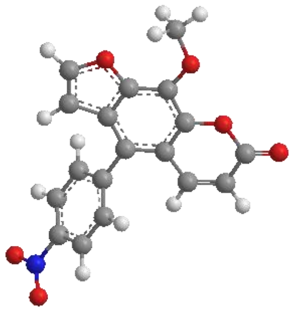

O -3.209240 1.756621 -0.057707
C -2.787092 3.059893 -0.158120
O -3.630705 3.920075 -0.167161
C -1.356769 3.257362 -0.255676
C -0.500162 2.219655 -0.206682
C -0.964471 0.866228 -0.055044
C -0.105517 -0.250576 -0.012833
C -0.709296 -1.507413 0.044552
C -0.205948 -2.854089 0.130688
C -1.286791 -3.654889 0.194831
O -2.454633 -2.949950 0.165451
C -2.110574 -1.639678 0.077432
C -2.986716 -0.558304 0.050579
C -2.360639 0.695748 -0.018219
C 1.366043 -0.125493 -0.008643
C 2.020932 0.634446 0.968890

C 3.401989 0.735862 0.983508
C 4.129025 0.064355 0.011369
C 3.514536 -0.703307 -0.967671
C 2.132881 -0.794142 -0.971002
N 5.588649 0.166582 0.020549
O 6.206385 -0.451511 -0.824858
O 6.103609 0.864340 0.872738
O -4.324473 -0.562733 0.083480
C -5.031401 -1.800565 0.075770
H -1.021325 4.279047 -0.377697
H 0.565250 2.398356 -0.298541
H 0.824565 -3.172087 0.145638
H -1.401038 -4.724958 0.265359
H 1.442527 1.133480 1.738419
H 3.918318 1.315219 1.737637
H 4.116409 -1.207969 -1.712038
H 1.637837 -1.376837 -1.739675
H -6.084503 -1.526270 0.068811
H -4.796865 -2.381730 -0.818526
H -4.809434 -2.387616 0.968849
SCF Done: E(RPBE1PBE) = -1197.38618557 A.U. after 15 cycles
E(PBE1PBE) = -3143737.6696912716 kJ/mol
Sum of electronic and zero-point Energies= -1197.225732
Sum of electronic and thermal Energies= -1197.206007
Sum of electronic and thermal Enthalpies= -1197.205063
Sum of electronic and thermal Free Energies= -1197.276059
HOMO -6.248 eV
LUMO -2.661 eV

Relevant excitation energies and oscillator strengths:

Excited State 1: Singlet-A 3.1663 eV 391.58 nm f=0.2374 <S**2>=0.000
87 -> 88 0.70247
This state for optimization and/or second-order correction.
Total Energy, E(TD-HF/TD-KS) = -1195.64638905
Copying the excited state density for this state as the 1-particle RhoCI density.

Excited State 2: Singlet-A 3.6428 eV 340.35 nm f=0.0074 <S**2>=0.000

86 -> 88 -0.12110
87 -> 89 0.68313

Excited State 3: Singlet-A 3.8940 eV 318.40 nm f=0.0102 <S**2>=0.000
81 -> 88 0.60716
81 -> 89 0.17968
81 -> 92 0.11471
86 -> 88 0.25127

Excited State 4: Singlet-A 3.9351 eV 315.08 nm f=0.0998 <S**2>=0.000
81 -> 88 -0.24568
86 -> 88 0.64127
87 -> 89 0.10427

Excited State 5: Singlet-A 4.3346 eV 286.03 nm f=0.0003 <S**2>=0.000
79 -> 88 0.46487
79 -> 89 0.13407
80 -> 88 0.46801
80 -> 89 0.13564

Excited State 6: Singlet-A 4.4793 eV 276.79 nm f=0.1511 <S**2>=0.000
86 -> 89 0.63310
87 -> 90 0.23193
87 -> 92 0.11138

Excited State 7: Singlet-A 4.6005 eV 269.50 nm f=0.0002 <S**2>=0.000
83 -> 89 0.13012
84 -> 88 0.12007
84 -> 89 -0.12765
85 -> 88 -0.38363
85 -> 89 0.47873
87 -> 90 -0.16753
87 -> 91 0.10401

Excited State 8: Singlet-A 4.6520 eV 266.52 nm f=0.0267 <S**2>=0.000
83 -> 88 -0.37057
85 -> 89 0.20803

86 -> 89 -0.12377

87 -> 90   0.38110

87 -> 91 -0.35554

Excited State 9: Singlet-A 4.8634 eV 254.93 nm f=0.1239 <S**2>=0.000

83 -> 88   0.49816

85 -> 88 -0.16905

87 -> 90   0.37261

87 -> 91 -0.17545

Excited State 10: Singlet-A 4.9833 eV 248.80 nm f=0.4745 <S**2>=0.000

84 -> 88 -0.16237

86 -> 89 -0.20085

86 -> 90 -0.10557

87 -> 90   0.31153

87 -> 91   0.53298

Excited State 11: Singlet-A 5.0175 eV 247.10 nm f=0.1061 <S**2>=0.000

82 -> 88   0.12865

84 -> 88   0.58281

84 -> 89 -0.11616

85 -> 88   0.28288

87 -> 91   0.12751

Excited State 12: Singlet-A 5.1588 eV 240.33 nm f=0.1530 <S**2>=0.000

82 -> 88   0.51606

83 -> 88   0.16709

84 -> 88 -0.25619

85 -> 88   0.27181

85 -> 89   0.19476

Excited State 13: Singlet-A 5.2326 eV 236.95 nm f=0.0683 <S**2>=0.000

82 -> 88   0.23245

85 -> 88 -0.18416

85 -> 89 -0.12388

86 -> 90 -0.33868

86 -> 91 -0.14041

87 -> 92   0.46627
87 -> 93   0.13716

Excited State 14: Singlet-A 5.2534 eV 236.01 nm f=0.0615 <S**2>=0.000
82 -> 88   0.37012
83 -> 88 -0.17512
84 -> 88   0.15163
85 -> 88 -0.35133
85 -> 89 -0.26868
86 -> 90   0.17871
87 -> 92 -0.22440

Excited State 15: Singlet-A 5.5095 eV 225.04 nm f=0.1617 <S**2>=0.000
84 -> 89 -0.16503
86 -> 90   0.53425
86 -> 91 -0.20557
87 -> 92   0.32662

Excited State 16: Singlet-A 5.5547 eV 223.21 nm f=0.0100 <S**2>=0.000
82 -> 89   0.13503
84 -> 89   0.61203
85 -> 89   0.20512
86 -> 90   0.10320
87 -> 92   0.11561

Excited State 17: Singlet-A 5.5831 eV 222.07 nm f=0.0082 <S**2>=0.000
83 -> 89   0.66215
84 -> 89   0.13397
85 -> 89 -0.12609

Excited State 18: Singlet-A 5.6307 eV 220.19 nm f=0.0824 <S**2>=0.000
82 -> 89 -0.13429
86 -> 91   0.60209
87 -> 92   0.25348
87 -> 93 -0.18578

Excited State 19: Singlet-A 5.7173 eV 216.86 nm f=0.0431 <S**2>=0.000

82 -> 89 0.65371

83 -> 89 0.12060

84 -> 89 -0.13833

Excited State 20: Singlet-A 5.9619 eV 207.96 nm f=0.2122 <S**2>=0.000

78 -> 88 0.22877

86 -> 90 0.14634

86 -> 91 0.17347

87 -> 93 0.59322

Excited State 21: Singlet-A 6.0481 eV 205.00 nm f=0.0082 <S**2>=0.000

78 -> 88 0.19082

81 -> 88 -0.20987

81 -> 89 0.60648

Excited State 22: Singlet-A 6.0630 eV 204.49 nm f=0.0195 <S**2>=0.000

79 -> 88 0.45499

79 -> 89 -0.13306

80 -> 88 -0.45253

80 -> 89 0.12266

Excited State 23: Singlet-A 6.0853 eV 203.74 nm f=0.0146 <S**2>=0.000

78 -> 88 0.53699

81 -> 89 -0.23969

86 -> 92 0.29599

87 -> 93 -0.15612

Excited State 24: Singlet-A 6.2402 eV 198.69 nm f=0.0377 <S**2>=0.000

78 -> 88 -0.30011

78 -> 89 -0.18062

86 -> 92 0.58667

8.5. XYZ-coordinates of the S_0 state of compound 11c (PBE1PBE/6-311G(d,p)) and TD-DFT calculation (PBE1PBE/6-21G, SCRF(IEFPCM, DCM)

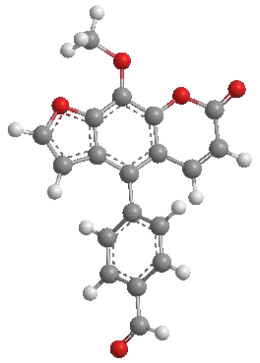

O -2.985608 1.691818 -0.071224

C -2.604749 3.009269 -0.144258

O -3.476485 3.841235 -0.164126

C -1.180056 3.255593 -0.202353

C -0.290553 2.246064 -0.145539

C -0.712439 0.876212 -0.023730

C 0.183197 -0.211022 0.025660

C -0.380472 -1.487321 0.053300

C 0.166312 -2.817577 0.133719

C -0.887650 -3.655211 0.162468

O -2.078201 -2.989423 0.114924

C -1.776855 -1.667130 0.051415

C -2.688330 -0.615509 0.016440

C -2.103094 0.658717 -0.023297

O -4.026474 -0.665012 0.018825

C -4.689929 -1.925583 -0.020923

C 1.650023 -0.037138 0.064767

C 2.257628 0.723499 1.069997

C 3.636703 0.863509 1.108202

C 4.432908 0.244189 0.145661

C 3.834254 -0.521346 -0.858190

C 2.459441 -0.661288 -0.896426

C 5.896340 0.400861 0.197330

O 6.668554 -0.094683 -0.591222

H -0.876293 4.289546 -0.302175

H 0.770675 2.459463 -0.206782

H 1.206849 -3.099491 0.167226

H -0.967448 -4.729359 0.216471

H -5.751491 -1.687401 -0.049716

H -4.415012 -2.487363 -0.916225

H -4.469306 -2.516631 0.869962

H 1.644661 1.188004 1.835080

H 4.102047 1.450709 1.894752

H 4.465468 -0.993566 -1.603364

H 1.994655 -1.243985 -1.684682

H 6.264850 1.025866 1.036581

SCF Done: E(RPBE1PBE) = -1106.29836889 A.U. after 14 cycles

E(PBE1PBE) = -2904586.5887987474 kJ/mol

Sum of electronic and zero-point Energies= -1106.099630

Sum of electronic and thermal Energies= -1106.080426

Sum of electronic and thermal Enthalpies= -1106.079482

Sum of electronic and thermal Free Energies= -1106.148776

HOMO -6.189 eV

LUMO -2.187 eV

Relevant excitation energies and oscillator strengths:

Excited State 1: Singlet-A 3.4209 eV 362.43 nm f=0.2659 <S**2>=0.000

83 -> 84 0.69425

This state for optimization and/or second-order correction.

Total Energy, E(TD-HF/TD-KS) = -1104.71454439

Copying the excited state density for this state as the 1-particle RhoCI density.

Excited State 2: Singlet-A 3.6993 eV 335.15 nm f=0.0001 <S**2>=0.000

81 -> 84 0.56083

81 -> 85 0.38117

81 -> 89 -0.11200

Excited State 3: Singlet-A 3.7793 eV 328.06 nm f=0.0435 <S**2>=0.000

82 -> 84 -0.10761

83 -> 84 0.10534

83 -> 85 0.68036

Excited State 4: Singlet-A 4.2005 eV 295.17 nm f=0.2124 <S**2>=0.000

82 -> 84 0.67136

83 -> 85 0.11547

83 -> 86 0.14185

Excited State 5: Singlet-A 4.6154 eV 268.63 nm f=0.0279 <S**2>=0.000
78 -> 84 -0.27595
78 -> 85 0.25163
80 -> 84 0.24218
80 -> 85 -0.14574
82 -> 85 0.48392
83 -> 86 -0.11788

Excited State 6: Singlet-A 4.6420 eV 267.09 nm f=0.0238 <S**2>=0.000
78 -> 84 0.29635
78 -> 85 -0.25337
79 -> 84 -0.10788
80 -> 84 -0.20888
80 -> 85 0.17175
82 -> 85 0.44840
83 -> 86 -0.17348

Excited State 7: Singlet-A 4.7996 eV 258.32 nm f=0.0279 <S**2>=0.000
78 -> 84 0.21827
79 -> 84 0.30223
79 -> 85 0.14067
80 -> 84 0.17741
83 -> 86 0.29124
83 -> 87 0.43113

Excited State 8: Singlet-A 5.0072 eV 247.61 nm f=0.5684 <S**2>=0.000
79 -> 84 -0.23606
80 -> 84 -0.10907
82 -> 84 -0.13436
82 -> 85 0.19852
82 -> 86 -0.12303
83 -> 86 0.54309
83 -> 87 -0.14169

Excited State 9: Singlet-A 5.0823 eV 243.95 nm f=0.0442 <S**2>=0.000
78 -> 84 -0.24051
79 -> 84 -0.17441
80 -> 84 -0.35663
83 -> 87 0.48473

Excited State 10: Singlet-A 5.1562 eV 240.45 nm f=0.2322 <S**2>=0.000
78 -> 84 -0.12013
79 -> 84   0.49354
80 -> 84 -0.39046
83 -> 87 -0.18827
83 -> 88   0.10778

Excited State 11: Singlet-A 5.2066 eV 238.13 nm f=0.0011 <S**2>=0.000
81 -> 84 -0.41069
81 -> 85   0.56435

Excited State 12: Singlet-A 5.3492 eV 231.78 nm f=0.0321 <S**2>=0.000
77 -> 84   0.66113
77 -> 85 -0.11027

Excited State 13: Singlet-A 5.3968 eV 229.74 nm f=0.0202 <S**2>=0.000
77 -> 84 -0.11861
82 -> 86   0.47672
83 -> 86   0.14747
83 -> 88   0.43985

Excited State 14: Singlet-A 5.4643 eV 226.90 nm f=0.0028 <S**2>=0.000
78 -> 84 -0.16172
78 -> 85   0.19246
79 -> 84 -0.12210
79 -> 85   0.48817
80 -> 85   0.38121

Excited State 15: Singlet-A 5.5897 eV 221.81 nm f=0.1332 <S**2>=0.000
78 -> 84 -0.21966
78 -> 85 -0.20682
79 -> 85 -0.33092
80 -> 84   0.22356
80 -> 85   0.46268

Excited State 16: Singlet-A 5.6833 eV 218.15 nm f=0.1316 <S**2>=0.000
77 -> 84   0.10012
78 -> 84   0.32722
78 -> 85   0.47715
79 -> 85 -0.24997
80 -> 85   0.17239

82 -> 86   0.15103
83 -> 88 -0.11753

Excited State 17: Singlet-A 5.7650 eV 215.06 nm f=0.0513 <S**2>=0.000
79 -> 85   0.12608
82 -> 86   0.30971
82 -> 87   0.54143
83 -> 88 -0.24672

Excited State 18: Singlet-A 5.8172 eV 213.13 nm f=0.0219 <S**2>=0.000
77 -> 84   0.10827
77 -> 85   0.63871
82 -> 87   0.16555
83 -> 88   0.16300

Excited State 19: Singlet-A 5.8759 eV 211.01 nm f=0.3663 <S**2>=0.000
76 -> 84   0.11555
77 -> 85 -0.24469
82 -> 86 -0.28150
82 -> 87   0.39227
83 -> 88   0.37264

Excited State 20: Singlet-A 5.9691 eV 207.71 nm f=0.0004 <S**2>=0.000
81 -> 86   0.38478
81 -> 87   0.58730

Excited State 21: Singlet-A 6.2907 eV 197.09 nm f=0.0424 <S**2>=0.000
76 -> 84   0.59532
82 -> 88   0.30375

Excited State 22: Singlet-A 6.3378 eV 195.63 nm f=0.0044 <S**2>=0.000
81 -> 85 -0.13810
81 -> 86   0.52994
81 -> 87 -0.36789
81 -> 88   0.12343
81 -> 89 -0.13627

Excited State 23: Singlet-A 6.3782 eV 194.39 nm f=0.0142 <S**2>=0.000
78 -> 86 -0.28562
78 -> 88   0.17023
80 -> 86   0.52159

80 -> 87 -0.10450

83 -> 89 0.14343

Excited State 24: Singlet-A 6.3991 eV 193.75 nm f=0.0788 <S**2>=0.000

77 -> 86 0.18735

80 -> 86 -0.12418

83 -> 89 0.63029

83 -> 90 0.12094

8.6. XYZ-coordinates of the S_0 state of compound 11d (PBE1PBE/6-311G(d,p)) and TD-DFT calculation (PBE1PBE/6-21G, SCRF(IEFPCM, DCM)

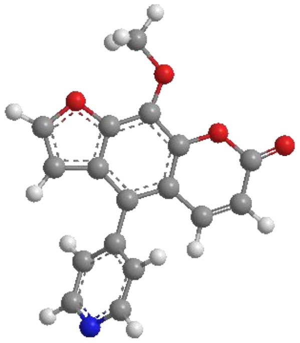

O -2.454131 1.621435 -0.043027

C -2.121180 2.952190 -0.110137

O -3.021986 3.752697 -0.111007

C -0.706944 3.249461 -0.184880

C 0.218272 2.271697 -0.150610

C -0.153593 0.886687 -0.037354

C 0.779385 -0.168521 -0.010720

C 0.263542 -1.464109 0.019978

C 0.858644 -2.774580 0.079505

C -0.164407 -3.649240 0.119743

O -1.378393 -3.026096 0.097723

C -1.125601 -1.693360 0.040718

C -2.074286 -0.674627 0.027857

C -1.535336 0.619929 -0.015662

O -3.409474 -0.771129 0.049124

C -4.028446 -2.054417 0.080934

C 2.239299 0.057528 0.001755

C 3.065788 -0.515696 -0.966501

C 4.434949 -0.295345 -0.903806

N 5.027996 0.439907 0.037514

C 4.236311 0.982413 0.962852
C 2.857250 0.824064 0.992133
H -0.441357 4.294565 -0.277077
H 1.270382 2.523627 -0.222639
H 1.908943 -3.019622 0.092487
H -0.205136 -4.725938 0.166356
H -5.098109 -1.853851 0.085575
H -3.767952 -2.639899 -0.803176
H -3.753143 -2.603284 0.983697
H 2.646922 -1.113329 -1.768523
H 5.088199 -0.731488 -1.655593
H 4.729073 1.574221 1.730342
H 2.276200 1.275946 1.788610
SCF Done: E(RPBE1PBE) = -1009.10550850 A.U. after 14 cycles
E(PBE1PBE) = -2649406.714387852 kJ/mol
Sum of electronic and zero-point Energies= -1008.914138
Sum of electronic and thermal Energies= -1008.897093
Sum of electronic and thermal Enthalpies= -1008.896149
Sum of electronic and thermal Free Energies= -1008.960755
HOMO -6.224 eV
LUMO -2.003 eV
Relevant excitation energies and oscillator strengths:

Excited State 1: Singlet-A 3.5514 eV 349.12 nm f=0.1126 <S**2>=0.000
76 -> 77 0.69442
This state for optimization and/or second-order correction.
Total Energy, E(TD-HF/TD-KS) = -1007.64106071
Copying the excited state density for this state as the 1-particle RhoCI density.

Excited State 2: Singlet-A 4.2947 eV 288.69 nm f=0.2318 <S**2>=0.000
75 -> 77 0.51604
76 -> 78 -0.45603

Excited State 3: Singlet-A 4.4467 eV 278.82 nm f=0.2216 <S**2>=0.000
75 -> 77 0.40883
76 -> 78 0.50337
76 -> 79 -0.19997
76 -> 80 -0.16417

Excited State 4: Singlet-A 4.5819 eV 270.60 nm f=0.0029 <S**2>=0.000

72 -> 77 0.10800
74 -> 77 0.58034
74 -> 78 0.32124
74 -> 79 -0.14823

Excited State 5: Singlet-A 4.6434 eV 267.01 nm f=0.0004 <S**2>=0.000
71 -> 77 -0.17623
72 -> 77 0.45993
72 -> 78 -0.11318
73 -> 77 0.42078
74 -> 77 -0.11848
74 -> 78 -0.10550

Excited State 6: Singlet-A 4.9027 eV 252.89 nm f=0.0543 <S**2>=0.000
76 -> 79 0.59848
76 -> 80 -0.31337

Excited State 7: Singlet-A 5.0742 eV 244.34 nm f=0.3182 <S**2>=0.000
74 -> 78 -0.11594
75 -> 77 -0.16452
75 -> 78 0.46637
76 -> 79 -0.15598
76 -> 80 -0.39782

Excited State 8: Singlet-A 5.1332 eV 241.53 nm f=0.0593 <S**2>=0.000
74 -> 77 -0.26999
74 -> 78 0.46836
74 -> 79 0.18716
74 -> 80 -0.35604
76 -> 80 -0.14661

Excited State 9: Singlet-A 5.1621 eV 240.18 nm f=0.1602 <S**2>=0.000
72 -> 77 0.10106
75 -> 77 0.10703
75 -> 78 0.50424
76 -> 78 0.11886
76 -> 79 0.19494

76 -> 80 0.36379

Excited State 10: Singlet-A 5.2714 eV 235.20 nm f=0.0135 <S**2>=0.000
72 -> 77 -0.36781
73 -> 77 0.44609
74 -> 77 0.11324
74 -> 79 0.26690
74 -> 80 -0.14539
76 -> 80 0.13688

Excited State 11: Singlet-A 5.2982 eV 234.01 nm f=0.0046 <S**2>=0.000
72 -> 77 0.17964
73 -> 77 -0.26446
74 -> 77 0.23231
74 -> 78 -0.24183
74 -> 79 0.46240
74 -> 80 -0.22853

Excited State 12: Singlet-A 5.4236 eV 228.60 nm f=0.0063 <S**2>=0.000
71 -> 77 0.64608
72 -> 77 0.21723

Excited State 13: Singlet-A 5.6101 eV 221.00 nm f=0.0207 <S**2>=0.000
75 -> 79 0.48456
75 -> 80 0.22045
76 -> 80 0.10359
76 -> 81 0.41902

Excited State 14: Singlet-A 5.7494 eV 215.65 nm f=0.0386 <S**2>=0.000
73 -> 78 0.12689
75 -> 79 -0.41671
75 -> 80 0.46890
76 -> 81 0.23081

Excited State 15: Singlet-A 5.8022 eV 213.68 nm f=0.1097 <S**2>=0.000
70 -> 77 0.65845
73 -> 78 -0.11941

75 -> 80 -0.12636

Excited State 16: Singlet-A 5.9338 eV 208.95 nm f=0.1057 <S**2>=0.000
69 -> 77   0.11737
70 -> 77   0.20590
70 -> 79   0.15107
70 -> 80 -0.11673
71 -> 78   0.13351
72 -> 78 -0.16706
73 -> 78   0.38749
73 -> 79 -0.11446
75 -> 79   0.22518
75 -> 80   0.14706
76 -> 81 -0.30895

Excited State 17: Singlet-A 5.9621 eV 207.96 nm f=0.2633 <S**2>=0.000
69 -> 77   0.12086
70 -> 79 -0.12785
70 -> 80   0.11288
72 -> 78   0.22364
73 -> 78 -0.31101
75 -> 80   0.39861
76 -> 81 -0.32334

Excited State 18: Singlet-A 6.2026 eV 199.89 nm f=0.0041 <S**2>=0.000
72 -> 77   0.10604
72 -> 78   0.49438
73 -> 78   0.30127
74 -> 78   0.13862
74 -> 79   0.18162
74 -> 80   0.25329

Excited State 19: Singlet-A 6.2338 eV 198.89 nm f=0.0056 <S**2>=0.000
72 -> 78 -0.27056
73 -> 78 -0.18028
74 -> 78   0.22979
74 -> 79   0.32940

74 -> 80   0.45777

Excited State 20: Singlet-A 6.2846 eV 197.28 nm f=0.1398 <S**2>=0.000
71 -> 78   0.65824
76 -> 82   0.13159

Excited State 21: Singlet-A 6.4418 eV 192.47 nm f=0.0038 <S**2>=0.000
66 -> 77   0.17767
68 -> 77   0.16081
72 -> 79   0.27109
72 -> 80   0.21437
72 -> 81 -0.22940
73 -> 79   0.31728
73 -> 80   0.23925
73 -> 81 -0.18012

Excited State 22: Singlet-A 6.4754 eV 191.47 nm f=0.0904 <S**2>=0.000
69 -> 77   0.53743
75 -> 81 -0.36346
76 -> 82   0.14341

Excited State 23: Singlet-A 6.6069 eV 187.66 nm f=0.0131 <S**2>=0.000
66 -> 77   0.12001
68 -> 77   0.28493
70 -> 78   0.17598
71 -> 78 -0.12174
72 -> 79   0.19037
72 -> 80   0.13655
73 -> 78 -0.12205
73 -> 79 -0.28745
75 -> 81   0.24390
76 -> 82   0.31413

Excited State 24: Singlet-A 6.6318 eV 186.95 nm f=0.0081 <S**2>=0.000
66 -> 77   0.13303
68 -> 77   0.50929
71 -> 79 -0.11660

72 -> 79 -0.13577

73 -> 80 -0.10799

75 -> 81 -0.18521

76 -> 82 -0.30151

8.7. XYZ-coordinates of the S_0 state of compound 11e (PBE1PBE/6-311G(d,p)) and TD-DFT calculation (PBE1PBE/6-21G, SCRF(IEFPCM, DCM)

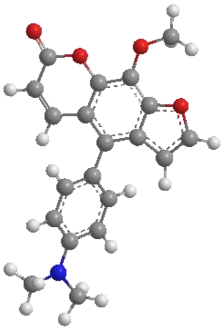

N 5.475733 0.237943 0.088968

C 4.112986 0.112117 0.052974

C 3.332599 0.813664 -0.890629

C 1.953432 0.699317 -0.903358

C 1.270859 -0.117487 0.004707

C 2.045840 -0.829857 0.926104

C 3.425163 -0.724598 0.957935

C -2.917402 3.019014 0.148043

C -1.494211 3.221461 0.300180

C -0.628616 2.189627 0.242386

C -1.074846 0.841193 0.027965

C -0.196672 -0.262511 -0.027916

C -0.788525 -1.524929 -0.136162

C -2.184208 -1.661674 -0.209469

C -3.073598 -0.593390 -0.177299

C -2.474481 0.660945 -0.051545

O -3.330083 1.716848 -0.005812

O -2.528123 -2.961448 -0.364693

C -0.278834 -2.867149 -0.267448

C -1.354752 -3.667308 -0.393488

O -3.768735 3.873104 0.156894

O -4.414067 -0.683712 -0.308340
C -5.075923 -1.606086 0.557847
C 6.253382 -0.607739 0.965682
C 6.155074 0.993698 -0.938728
H 3.807183 1.445950 -1.629983
H 1.393680 1.240429 -1.660567
H 1.554863 -1.473583 1.649731
H 3.972105 -1.295208 1.697560
H -1.170810 4.239935 0.471830
H 0.432871 2.365797 0.375769
H 0.753284 -3.180306 -0.269356
H -1.466397 -4.733188 -0.512882
H -6.141928 -1.443792 0.405748
H -4.822981 -2.639131 0.310669
H -4.821562 -1.401648 1.602249
H 5.965663 -0.467219 2.013104
H 6.147967 -1.674447 0.723255
H 7.305354 -0.341039 0.875078
H 5.809517 2.032779 -0.963016
H 7.222401 1.006804 -0.722786
H 6.013118 0.564072 -1.940260
SCF Done: E(RPBE1PBE) = -1126.88164698 A.U. after 14 cycles
E(PBE1PBE) = -2958627.9895223193 kJ/mol
Sum of electronic and zero-point Energies= -1126.646414
Sum of electronic and thermal Energies= -1126.624656
Sum of electronic and thermal Enthalpies= -1126.623712
Sum of electronic and thermal Free Energies= -1126.698920
HOMO -5.550 eV
LUMO -1.841 eV
Relevant excitation energies and oscillator strengths:

Excited State 1: Singlet-A 3.0960 eV 400.46 nm f=0.2105 <S**2>=0.000
88 -> 89 0.70370
This state for optimization and/or second-order correction.
Total Energy, E(TD-HF/TD-KS) = -1125.33810330
Copying the excited state density for this state as the 1-particle RhoCI density.

Excited State 2: Singlet-A 3.8824 eV 319.35 nm f=0.0147 <S**2>=0.000

87 -> 89 0.67522

88 -> 90 0.14819

Excited State 3: Singlet-A 4.2134 eV 294.26 nm f=0.2763 <S**2>=0.000

86 -> 89 0.25174

87 -> 89 -0.16362

88 -> 90 0.63279

Excited State 4: Singlet-A 4.4682 eV 277.48 nm f=0.3374 <S**2>=0.000

86 -> 89 0.61547

87 -> 90 -0.13499

88 -> 90 -0.25559

Excited State 5: Singlet-A 4.5946 eV 269.85 nm f=0.0384 <S**2>=0.000

85 -> 89 0.16204

88 -> 91 0.65991

Excited State 6: Singlet-A 4.6778 eV 265.05 nm f=0.0068 <S**2>=0.000

83 -> 89 0.48168

84 -> 89 0.42910

85 -> 89 -0.18447

Excited State 7: Singlet-A 4.9286 eV 251.56 nm f=0.3261 <S**2>=0.000

87 -> 90 -0.17899

87 -> 92 -0.10698

88 -> 92 0.64832

Excited State 8: Singlet-A 4.9839 eV 248.77 nm f=0.0256 <S**2>=0.000

83 -> 89 0.13666

84 -> 89 0.12889

85 -> 89 0.65320

88 -> 91 -0.13621

88 -> 92 0.10900

Excited State 9: Singlet-A 5.1961 eV 238.61 nm f=0.3952 <S**2>=0.000

86 -> 89 0.15126

86 -> 90 -0.26001

87 -> 90   0.59345

Excited State 10: Singlet-A 5.3530 eV 231.62 nm f=0.1541 <S**2>=0.000
84 -> 89 -0.11288
86 -> 90   0.54131
87 -> 90   0.25077
87 -> 92   0.21471
88 -> 92   0.19862
88 -> 93 -0.12604

Excited State 11: Singlet-A 5.3618 eV 231.24 nm f=0.0155 <S**2>=0.000
83 -> 89 -0.44044
84 -> 89   0.50446
86 -> 90   0.14877

Excited State 12: Singlet-A 5.5896 eV 221.81 nm f=0.0055 <S**2>=0.000
87 -> 91   0.67717
88 -> 93   0.12484

Excited State 13: Singlet-A 5.6383 eV 219.90 nm f=0.0882 <S**2>=0.000
87 -> 91 -0.14653
87 -> 92   0.27718
88 -> 93   0.61053

Excited State 14: Singlet-A 5.7933 eV 214.01 nm f=0.2536 <S**2>=0.000
81 -> 89   0.13018
86 -> 89 -0.11454
86 -> 90 -0.25948
87 -> 92   0.55646
88 -> 93 -0.22422

Excited State 15: Singlet-A 5.9213 eV 209.39 nm f=0.0137 <S**2>=0.000
82 -> 89   0.68794

Excited State 16: Singlet-A 6.0666 eV 204.37 nm f=0.0034 <S**2>=0.000
85 -> 90   0.18589
86 -> 91   0.66745

Excited State 17: Singlet-A 6.1101 eV 202.92 nm f=0.0196 <S**2>=0.000
85 -> 90   0.65904
86 -> 91 -0.18145

Excited State 18: Singlet-A 6.1321 eV 202.19 nm f=0.0234 <S**2>=0.000
80 -> 89 -0.14364
81 -> 89 -0.43563
86 -> 92   0.48129
87 -> 92   0.10321

Excited State 19: Singlet-A 6.2047 eV 199.82 nm f=0.0153 <S**2>=0.000
80 -> 89 -0.26438
81 -> 89   0.45365
86 -> 92   0.40322
88 -> 94   0.16070

Excited State 20: Singlet-A 6.2404 eV 198.68 nm f=0.0964 <S**2>=0.000
81 -> 89 -0.10322
83 -> 90   0.19383
84 -> 90 -0.29525
87 -> 93 -0.16427
88 -> 94   0.55012

Excited State 21: Singlet-A 6.3405 eV 195.54 nm f=0.0195 <S**2>=0.000
80 -> 89 -0.15450
83 -> 90   0.10264
84 -> 90 -0.41023
87 -> 93   0.48781
88 -> 94 -0.14548

Excited State 22: Singlet-A 6.3623 eV 194.87 nm f=0.1329 <S**2>=0.000
81 -> 89 -0.18936
84 -> 90   0.40435
87 -> 93   0.41215
88 -> 94   0.29494

Excited State 23: Singlet-A 6.4401 eV 192.52 nm f=0.0218 <S**2>=0.000

83 -> 90 0.61913

83 -> 92 -0.12229

84 -> 90 0.11133

88 -> 94 -0.17212

Excited State 24: Singlet-A 6.6097 eV 187.58 nm f=0.0214 <S**2>=0.000

77 -> 89 -0.18379

78 -> 89 0.31221

79 -> 89 -0.10735

80 -> 89 -0.20256

83 -> 89 -0.11240

83 -> 90 -0.18746

83 -> 92 -0.25055

83 -> 93 -0.14395

84 -> 92 -0.21356

84 -> 93 -0.12098

85 -> 92 0.14946

86 -> 93 0.20449

8.8. XYZ-coordinates of the S_0 state of compound 11f (PBE1PBE/6-311G(d,p)) and TD-DFT calculation (PBE1PBE/6-21G, SCRF(IEFPCM, DCM)

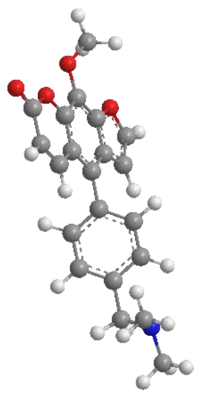

O -3.844837 1.530411 -0.019990

C -3.566101 2.873973 -0.079027

O -4.500078 3.636606 -0.075783

C -2.166125 3.230070 -0.151658

C -1.201063 2.290572 -0.121177

C -1.514085 0.891455 -0.014581

C -0.533118 -0.119915 0.010660
C -0.995704 -1.436017 0.036635
C -0.345596 -2.720059 0.106828
C -1.329789 -3.637603 0.152564
O -2.570519 -3.065783 0.124459
C -2.373179 -1.724647 0.054523
C -3.365995 -0.748943 0.036691
C -2.885197 0.566458 0.002436
C 0.917327 0.170615 0.026681
C 1.491393 0.943205 1.039643
C 2.860097 1.182743 1.060630
C 3.693833 0.665712 0.071922
C 3.122963 -0.109851 -0.937903
C 1.758319 -0.356960 -0.959410
O -4.697014 -0.915903 0.074339
C -5.248667 -2.192235 -0.237246
C 5.171555 0.968587 0.066719
N 5.986067 -0.165615 -0.329284
C 7.342262 0.242412 -0.635774
C 5.973352 -1.208975 0.677596
H -1.944476 4.285784 -0.239259
H -0.159059 2.582719 -0.189818
H 0.714594 -2.917748 0.122825
H -1.325549 -4.714610 0.208369
H 0.864626 1.337457 1.833535
H 3.286708 1.773155 1.866577
H 3.768167 -0.525643 -1.704847
H 1.331795 -0.954903 -1.758832
H -6.324874 -2.035847 -0.285420
H -4.886824 -2.549363 -1.204824
H -5.017170 -2.925605 0.536963
H 5.357584 1.776379 -0.651857
H 5.465723 1.361460 1.058898
H 7.916464 -0.621220 -0.981707
H 7.336508 0.989639 -1.433864
H 7.869558 0.673394 0.235804
H 6.542583 -2.071911 0.322287

H 4.947857 -1.533620 0.869299

H 6.413993 -0.876449 1.636158

SCF Done: E(RPBE1PBE) = -1166.15606356 A.U. after 14 cycles

E(PBE1PBE) = -3061742.9781079926 kJ/mol

Sum of electronic and zero-point Energies= -1165.885679

Sum of electronic and thermal Energies= -1165.863090

Sum of electronic and thermal Enthalpies= -1165.862146

Sum of electronic and thermal Free Energies= -1165.939075

HOMO -6.076 eV

LUMO -1.872 eV

Relevant excitation energies and oscillator strengths:

Excited State 1: Singlet-A 3.5316 eV 351.07 nm f=0.1247 <S**2>=0.000

91 -> 93 -0.39263

92 -> 93 0.57528

This state for optimization and/or second-order correction.

Total Energy, E(TD-HF/TD-KS) = -1164.53813503

Copying the excited state density for this state as the 1-particle RhoCI density.

Excited State 2: Singlet-A 3.8037 eV 325.96 nm f=0.0005 <S**2>=0.000

91 -> 93 0.57669

92 -> 93 0.39827

Excited State 3: Singlet-A 4.3263 eV 286.58 nm f=0.2796 <S**2>=0.000

90 -> 93 0.62978

91 -> 94 0.16091

92 -> 94 -0.20651

Excited State 4: Singlet-A 4.6082 eV 269.05 nm f=0.0672 <S**2>=0.000

87 -> 93 0.14780

89 -> 93 0.13857

91 -> 94 0.24904

92 -> 94 0.55152

92 -> 95 0.19337

92 -> 96 -0.11874

Excited State 5: Singlet-A 4.6551 eV 266.34 nm f=0.0052 <S**2>=0.000

87 -> 93 0.54072

87 -> 97 -0.10927

88 -> 93 -0.11254

89 -> 93   0.25841

91 -> 94 -0.27377

Excited State 6: Singlet-A 4.7219 eV 262.57 nm f=0.3095 <S**2>=0.000

87 -> 93   0.24939

90 -> 93 -0.19448

91 -> 94   0.50403

91 -> 95   0.18854

92 -> 94 -0.26582

92 -> 95 -0.10259

Excited State 7: Singlet-A 4.8913 eV 253.48 nm f=0.0986 <S**2>=0.000

87 -> 93 -0.24123

89 -> 93   0.61329

91 -> 95   0.11624

92 -> 95 -0.14223

Excited State 8: Singlet-A 4.9542 eV 250.26 nm f=0.0096 <S**2>=0.000

88 -> 93   0.40729

92 -> 95   0.28942

92 -> 96   0.44926

Excited State 9: Singlet-A 5.0121 eV 247.37 nm f=0.0151 <S**2>=0.000

88 -> 93   0.48163

91 -> 95 -0.17638

91 -> 96 -0.33132

92 -> 95 -0.26037

92 -> 96 -0.18707

Excited State 10: Singlet-A 5.0636 eV 244.85 nm f=0.2881 <S**2>=0.000

89 -> 93   0.11993

90 -> 93 -0.18048

90 -> 94   0.29150

91 -> 95 -0.35905

91 -> 96 -0.13457

92 -> 94 -0.13588

92 -> 95 0.37667

92 -> 96 -0.18019

Excited State 11: Singlet-A 5.1671 eV 239.95 nm f=0.0161 <S**2>=0.000

88 -> 93 0.26587

90 -> 94 0.13393

91 -> 95 0.22915

91 -> 96 0.40814

92 -> 96 -0.39268

Excited State 12: Singlet-A 5.2320 eV 236.97 nm f=0.0151 <S**2>=0.000

90 -> 94 -0.15229

91 -> 94 -0.22190

91 -> 95 0.37574

91 -> 96 -0.37157

92 -> 94 -0.17246

92 -> 95 0.27753

92 -> 96 -0.15937

Excited State 13: Singlet-A 5.3732 eV 230.74 nm f=0.2156 <S**2>=0.000

86 -> 93 0.11007

90 -> 94 0.55656

91 -> 94 -0.11524

91 -> 95 0.21209

91 -> 96 -0.15084

91 -> 97 -0.12120

92 -> 95 -0.16034

92 -> 97 0.12763

Excited State 14: Singlet-A 5.4879 eV 225.92 nm f=0.0014 <S**2>=0.000

86 -> 93 0.67712

Excited State 15: Singlet-A 5.7377 eV 216.09 nm f=0.0889 <S**2>=0.000

88 -> 94 0.32737

88 -> 95 0.16013

89 -> 94 0.20478

89 -> 95 -0.14932

89 -> 96 -0.22295

90 -> 94   0.11378

90 -> 96 -0.24124

91 -> 97   0.21776

92 -> 96 -0.10617

92 -> 97 -0.29240

Excited State 16: Singlet-A 5.7791 eV 214.54 nm f=0.0551 <S**2>=0.000

88 -> 94 -0.25777

88 -> 95 -0.13531

89 -> 94   0.12713

89 -> 96   0.20986

90 -> 94   0.14389

90 -> 95   0.34830

90 -> 96   0.10298

91 -> 97   0.23737

92 -> 97 -0.32177

Excited State 17: Singlet-A 5.9178 eV 209.51 nm f=0.1456 <S**2>=0.000

85 -> 93 -0.11482

89 -> 94 -0.40446

90 -> 95   0.49846

90 -> 96 -0.15684

Excited State 18: Singlet-A 6.0089 eV 206.33 nm f=0.0158 <S**2>=0.000

88 -> 94   0.16098

89 -> 94   0.29821

89 -> 95 -0.12377

89 -> 96 -0.13431

90 -> 95   0.25779

90 -> 96   0.47670

92 -> 97   0.16274

Excited State 19: Singlet-A 6.0748 eV 204.10 nm f=0.0905 <S**2>=0.000

85 -> 93   0.16373

88 -> 94   0.16016

89 -> 94 -0.38559
89 -> 96 -0.13209
90 -> 95 -0.12198
90 -> 96 0.39646
92 -> 97 -0.27342

Excited State 20: Singlet-A 6.1332 eV 202.15 nm f=0.0072 <S**2>=0.000
91 -> 97 0.57007
92 -> 97 0.38166

Excited State 21: Singlet-A 6.3140 eV 196.36 nm f=0.0176 <S**2>=0.000
87 -> 94 -0.15401
88 -> 94 0.45388
88 -> 95 -0.36228
88 -> 96 0.18657
89 -> 95 0.11806
89 -> 96 0.24036

Excited State 22: Singlet-A 6.4004 eV 193.71 nm f=0.2143 <S**2>=0.000
85 -> 93 0.28420
86 -> 94 0.51568
91 -> 98 -0.18192
92 -> 98 0.22628

Excited State 23: Singlet-A 6.4103 eV 193.41 nm f=0.0032 <S**2>=0.000
84 -> 93 0.11687
87 -> 94 0.58806
87 -> 95 -0.20520
88 -> 94 0.10530
88 -> 95 -0.12782
89 -> 96 0.10664

Excited State 24: Singlet-A 6.4807 eV 191.31 nm f=0.0451 <S**2>=0.000
85 -> 93 -0.27006
86 -> 94 0.24204
87 -> 94 0.21721
89 -> 95 0.36932

89 -> 96 -0.15862

90 -> 97 0.29783

8.9. XYZ-coordinates of the S_0 state of compound 11g (PBE1PBE/6-311G(d,p)) and TD-DFT calculation (PBE1PBE/6-21G, SCRF(IEFPCM, DCM)

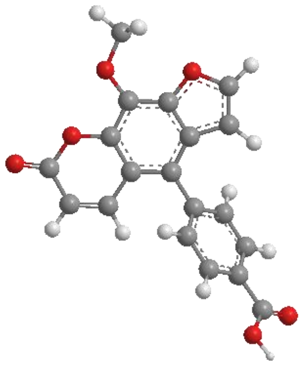

C -5.621242 0.100770 -0.051760

C -4.141336 0.036341 -0.012519

C -3.395174 0.690858 0.967545

C -2.011917 0.600116 0.961972

C -1.346655 -0.141276 -0.021210

C -2.106018 -0.798670 -0.996580

C -3.487702 -0.709501 -0.993155

O -6.307387 -0.452398 -0.878243

O -6.138481 0.844446 0.934545

C 2.785539 3.070576 -0.158084

C 1.354356 3.257400 -0.260051

C 0.505470 2.212959 -0.212776

C 0.979370 0.863473 -0.059542

C 0.126839 -0.257840 -0.018182

C 0.739957 -1.509495 0.045150

C 2.141977 -1.632750 0.084505

C 3.011376 -0.545705 0.058488

C 2.377124 0.703097 -0.016108

O 3.217732 1.771154 -0.053502

O 2.493889 -2.940843 0.177478

C 0.244585 -2.859376 0.131413

C 1.329781 -3.653324 0.202654

O 3.623006 3.937354 -0.166120

O 4.350205 -0.541685 0.099632
C 5.063527 -1.775032 0.076299
H -3.900104 1.262526 1.736685
H -1.438725 1.092615 1.740407
H -1.603262 -1.368751 -1.770491
H -4.078890 -1.209564 -1.751858
H -7.099110 0.827128 0.830067
H 1.011817 4.276614 -0.383266
H -0.561613 2.381241 -0.305550
H -0.784523 -3.182141 0.140516
H 1.450837 -4.722472 0.275917
H 6.115341 -1.495454 0.070491
H 4.846720 -2.374222 0.962632
H 4.830653 -2.347478 -0.824232
SCF Done: E(RPBE1PBE) = -1181.50156186 A.U. after 14 cycles
E(PBE1PBE) = -3102032.5869637425 kJ/mol
Sum of electronic and zero-point Energies= -1181.298532
Sum of electronic and thermal Energies= -1181.278502
Sum of electronic and thermal Enthalpies= -1181.277557
Sum of electronic and thermal Free Energies= -1181.348908
HOMO -2.040 eV
LUMO -6.174 eV
Relevant excitation energies and oscillator strengths:
Excited State 1: Singlet-A 3.4956 eV 354.68 nm f=0.1905 <S**2>=0.000
87 -> 88 0.69343
This state for optimization and/or second-order correction.
Total Energy, E(TD-HF/TD-KS) = -1179.77700095
Copying the excited state density for this state as the 1-particle RhoCI density.

Excited State 2: Singlet-A 3.9909 eV 310.67 nm f=0.1482 <S**2>=0.000
86 -> 88 0.14863
87 -> 89 0.67976

Excited State 3: Singlet-A 4.3032 eV 288.12 nm f=0.2372 <S**2>=0.000
86 -> 88 0.64694
87 -> 89 -0.14758
87 -> 90 -0.18926

87 -> 92   0.11991

Excited State 4: Singlet-A 4.6405 eV 267.18 nm f=0.0003 <S**2>=0.000

83 -> 88   0.50749

83 -> 89   0.23197

83 -> 92 -0.12522

85 -> 88 -0.35133

85 -> 89 -0.12920

Excited State 5: Singlet-A 4.8244 eV 256.99 nm f=0.0064 <S**2>=0.000

82 -> 88 -0.10954

82 -> 89   0.10486

86 -> 89   0.63062

87 -> 90 -0.23375

Excited State 6: Singlet-A 4.8675 eV 254.72 nm f=0.0826 <S**2>=0.000

82 -> 88   0.11331

82 -> 89 -0.10268

83 -> 88   0.13603

84 -> 88 -0.15332

84 -> 89   0.11838

85 -> 88   0.19356

85 -> 89 -0.10432

86 -> 89 -0.18832

87 -> 90 -0.36935

87 -> 91   0.41389

Excited State 7: Singlet-A 4.8834 eV 253.89 nm f=0.0172 <S**2>=0.000

81 -> 88   0.11279

81 -> 89 -0.11288

82 -> 88 -0.43323

82 -> 89   0.44323

82 -> 90   0.10201

82 -> 94 -0.10528

86 -> 89 -0.19651

Excited State 8: Singlet-A 5.0373 eV 246.13 nm f=0.4866 <S**2>=0.000

84 -> 88 -0.22567
85 -> 88   0.11833
86 -> 88   0.18104
86 -> 89   0.12002
86 -> 90 -0.12702
87 -> 90   0.45841
87 -> 91   0.34319

Excited State 9: Singlet-A 5.1515 eV 240.68 nm f=0.0506 <S**2>=0.000
83 -> 88   0.27536
84 -> 88 -0.10888
85 -> 88   0.49525
87 -> 91 -0.34977

Excited State 10: Singlet-A 5.2135 eV 237.81 nm f=0.1403 <S**2>=0.000
83 -> 88   0.15062
84 -> 88   0.60939
85 -> 88   0.16840
87 -> 91   0.19534

Excited State 11: Singlet-A 5.4240 eV 228.58 nm f=0.0062 <S**2>=0.000
81 -> 88   0.57022
82 -> 88   0.14799
83 -> 88 -0.11445
86 -> 90 -0.25064
87 -> 90 -0.11775
87 -> 92 -0.19175

Excited State 12: Singlet-A 5.4515 eV 227.43 nm f=0.0338 <S**2>=0.000
81 -> 88   0.31560
83 -> 88 -0.11361
86 -> 90   0.42608
87 -> 90   0.11849
87 -> 92   0.38755

Excited State 13: Singlet-A 5.5364 eV 223.94 nm f=0.0157 <S**2>=0.000
83 -> 89   0.11697

84 -> 88 -0.11594

84 -> 89 -0.41283

84 -> 91 -0.12832

85 -> 88   0.11170

85 -> 89   0.42086

85 -> 90   0.10306

85 -> 91 -0.11208

86 -> 91   0.13465

87 -> 91   0.14120

Excited State 14: Singlet-A 5.7704 eV 214.86 nm f=0.3538 <S**2>=0.000

84 -> 89   0.23264

85 -> 89   0.32358

86 -> 90   0.36997

86 -> 91 -0.24565

87 -> 92 -0.33609

Excited State 15: Singlet-A 5.8404 eV 212.29 nm f=0.0596 <S**2>=0.000

82 -> 88   0.10788

84 -> 89   0.35723

85 -> 89   0.24252

86 -> 91   0.50026

Excited State 16: Singlet-A 5.8442 eV 212.15 nm f=0.0031 <S**2>=0.000

81 -> 88 -0.14516

82 -> 88   0.47793

82 -> 89   0.44813

84 -> 89 -0.10775

Excited State 17: Singlet-A 5.9079 eV 209.86 nm f=0.0972 <S**2>=0.000

83 -> 88 -0.16991

83 -> 89   0.38735

85 -> 88   0.11563

85 -> 89 -0.22065

86 -> 90   0.16949

86 -> 91   0.33296

87 -> 92 -0.27388

Excited State 18: Singlet-A 5.9836 eV 207.21 nm f=0.0777 <S**2>=0.000
79 -> 88 -0.11831
83 -> 88 -0.19036
83 -> 89   0.47381
84 -> 89   0.21052
85 -> 89   0.12063
86 -> 90 -0.14358
86 -> 91 -0.19278
87 -> 92   0.23320

Excited State 19: Singlet-A 6.0454 eV 205.09 nm f=0.1714 <S**2>=0.000
81 -> 89   0.66600
82 -> 89   0.14676

Excited State 20: Singlet-A 6.3653 eV 194.78 nm f=0.0873 <S**2>=0.000
79 -> 88   0.43156
80 -> 88   0.22470
85 -> 90 -0.25328
86 -> 92 -0.31902
87 -> 93   0.11505

Excited State 21: Singlet-A 6.3976 eV 193.80 nm f=0.0112 <S**2>=0.000
79 -> 88   0.25120
83 -> 90 -0.31097
83 -> 92   0.17953
85 -> 90   0.43457
85 -> 91   0.12831
86 -> 92 -0.13618

Excited State 22: Singlet-A 6.4929 eV 190.95 nm f=0.0500 <S**2>=0.000
81 -> 90   0.20250
83 -> 90 -0.17360
84 -> 90 -0.30660
86 -> 92   0.25022
87 -> 93   0.45464

Excited State 23: Singlet-A 6.5171 eV 190.24 nm f=0.0556 <S**2>=0.000

76 -> 88 -0.16351

78 -> 88 0.32503

81 -> 90 0.15550

83 -> 90 0.29308

83 -> 92 -0.10594

85 -> 90 0.34664

86 -> 92 0.10369

87 -> 93 0.18172

Excited State 24: Singlet-A 6.5443 eV 189.46 nm f=0.0077 <S**2>=0.000

80 -> 88 0.17052

83 -> 90 -0.12663

84 -> 90 0.54750

84 -> 91 0.19902

85 -> 90 -0.17433

86 -> 92 0.15045

87 -> 93 0.14240

8.10. XYZ-coordinates of the S_0 state of compound 13a (PBE1PBE/6-311G(d,p)) and TD-DFT calculation (PBE1PBE/6-21G, SCRF(IEFPCM, DCM)

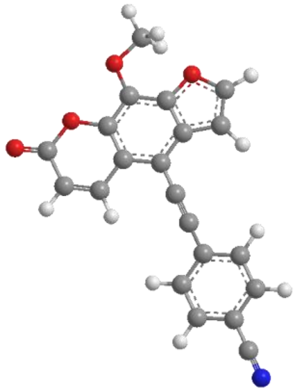

O -3.714014 1.824324 -0.000117

C -3.234613 3.113827 -0.000211

O -4.042258 4.007181 -0.000480

C -1.793625 3.258484 0.000080

C -0.981962 2.184109 0.000216

C -1.513571 0.851412 0.000098

C -0.693800 -0.301435 0.000168

C -1.342445 -1.542522 0.000099
C -0.884743 -2.901699 0.000075
C -1.994959 -3.666989 0.000199
O -3.136145 -2.921127 0.000007
C -2.745300 -1.620602 0.000015
C -3.581380 -0.505617 -0.000083
C -2.909382 0.730765 -0.000024
O -4.914934 -0.461477 -0.000222
C -5.669170 -1.672518 0.000078
C 0.713311 -0.213779 0.000184
C 1.924482 -0.152058 0.000236
C 3.338280 -0.076523 0.000149
C 4.113708 -1.247891 -0.000467
C 5.493154 -1.174915 -0.000575
C 6.125544 0.073753 -0.000071
C 5.362681 1.247325 0.000542
C 3.983376 1.171408 0.000660
C 7.548701 0.149645 -0.000196
N 8.702298 0.210833 -0.000294
H -1.415267 4.272420 0.000148
H 0.096315 2.306510 0.000422
H 0.136812 -3.248109 0.000044
H -2.147485 -4.734627 0.000281
H -6.710700 -1.357920 0.000011
H -5.461499 -2.263581 -0.893794
H -5.461482 -2.263134 0.894248
H 3.622138 -2.213416 -0.000847
H 6.087369 -2.080989 -0.001077
H 5.855889 2.212155 0.000962
H 3.390902 2.078584 0.001124
SCF Done: E(RPBE1PBE) = -1161.31334046 A.U. after 15 cycles
E(PBE1PBE) = -3049028.407640398 kJ/mol
Sum of electronic and zero-point Energies= -1161.111504
Sum of electronic and thermal Energies= -1161.090509
Sum of electronic and thermal Enthalpies= -1161.089564
Sum of electronic and thermal Free Energies= -1161.164090HOMO -6.085 eV
LUMO -2.499 eV

Relevant excitation energies and oscillator strengths:

Excited State 1: Singlet-A 3.1198 eV 397.41 nm f=0.8154 <S**2>=0.000

88 -> 89 0.69737

This state for optimization and/or second-order correction.

Total Energy, E(TD-HF/TD-KS) = -1159.70734156

Copying the excited state density for this state as the 1-particle RhoCI density.

Excited State 2: Singlet-A 3.7736 eV 328.55 nm f=0.3676 <S**2>=0.000

87 -> 89 -0.32389

88 -> 90 0.60918

Excited State 3: Singlet-A 3.9739 eV 312.00 nm f=0.1949 <S**2>=0.000

87 -> 89 0.60258

88 -> 90 0.33128

88 -> 91 0.10493

Excited State 4: Singlet-A 4.5130 eV 274.73 nm f=0.0003 <S**2>=0.000

85 -> 89 0.60342

85 -> 90 0.33651

85 -> 93 -0.12073

Excited State 5: Singlet-A 4.6890 eV 264.42 nm f=0.1359 <S**2>=0.000

86 -> 89 0.61393

87 -> 90 -0.24629

88 -> 91 -0.17181

88 -> 93 -0.11270

Excited State 6: Singlet-A 4.6968 eV 263.98 nm f=0.0006 <S**2>=0.000

84 -> 89 0.36867

84 -> 90 -0.13224

86 -> 92 -0.13654

88 -> 92 0.56136

Excited State 7: Singlet-A 4.7607 eV 260.43 nm f=0.0783 <S**2>=0.000

86 -> 89 0.27141

87 -> 90 0.50215

88 -> 91 0.34245

88 -> 93 -0.13767

Excited State 8: Singlet-A 4.8438 eV 255.97 nm f=0.4530 <S**2>=0.000

87 -> 89 -0.10093

87 -> 90 -0.40848

87 -> 91   0.10633

88 -> 91   0.54060

Excited State 9: Singlet-A 4.9180 eV 252.10 nm f=0.0000 <S**2>=0.000

82 -> 89   0.68322

85 -> 90   0.11212

Excited State 10: Singlet-A 5.1104 eV 242.61 nm f=0.0338 <S**2>=0.000

83 -> 89   0.68675

Excited State 11: Singlet-A 5.1664 eV 239.98 nm f=0.0520 <S**2>=0.000

84 -> 89   0.54612

86 -> 92 -0.13384

88 -> 92 -0.41374

Excited State 12: Singlet-A 5.3417 eV 232.10 nm f=0.0289 <S**2>=0.000

86 -> 90   0.12217

87 -> 91 -0.40179

88 -> 91   0.10261

88 -> 93   0.53461

Excited State 13: Singlet-A 5.5529 eV 223.28 nm f=0.0000 <S**2>=0.000

88 -> 94   0.64841

88 -> 95 -0.21660

Excited State 14: Singlet-A 5.5846 eV 222.01 nm f=0.0001 <S**2>=0.000

82 -> 89 -0.12631

85 -> 89 -0.35967

85 -> 90   0.56414

85 -> 93 -0.16223

Excited State 15: Singlet-A 5.5972 eV 221.51 nm f=0.0641 <S**2>=0.000

86 -> 90   0.67226

Excited State 16: Singlet-A 5.7244 eV 216.59 nm f=0.3383 <S**2>=0.000

81 -> 89   0.14744

83 -> 90   0.10006

86 -> 89   0.10419

87 -> 91   0.53226

88 -> 93   0.37690

Excited State 17: Singlet-A 5.8056 eV 213.56 nm f=0.0004 <S**2>=0.000
84 -> 89   0.20801
84 -> 90   0.46415
86 -> 92   0.18113
87 -> 92 -0.43412

Excited State 18: Singlet-A 5.9666 eV 207.80 nm f=0.0143 <S**2>=0.000
81 -> 89   0.11981
84 -> 90   0.38648
86 -> 92   0.18572
87 -> 92   0.53677

Excited State 19: Singlet-A 5.9688 eV 207.72 nm f=0.0114 <S**2>=0.000
81 -> 89   0.61415
83 -> 90 -0.20641
87 -> 92 -0.10102

Excited State 20: Singlet-A 5.9935 eV 206.86 nm f=0.1098 <S**2>=0.000
81 -> 89   0.16402
83 -> 90   0.64832
87 -> 91 -0.11933

Excited State 21: Singlet-A 5.9965 eV 206.76 nm f=0.0002 <S**2>=0.000
82 -> 90   0.69130

Excited State 22: Singlet-A 6.2603 eV 198.05 nm f=0.0043 <S**2>=0.000
80 -> 89   0.49063
86 -> 91 -0.17176
87 -> 93 -0.43484

Excited State 23: Singlet-A 6.3104 eV 196.48 nm f=0.0329 <S**2>=0.000
80 -> 89   0.22516
86 -> 91   0.62264
88 -> 96 -0.18748

Excited State 24: Singlet-A 6.3686 eV 194.68 nm f=0.0879 <S**2>=0.000
83 -> 91   0.17079
86 -> 91   0.22122
88 -> 96   0.61373

8.11. XYZ-coordinates of the S_0 state of compound 13b (PBE1PBE/6-311G(d,p)) and TD-DFT calculation (PBE1PBE/6-21G, SCRF(IEFPCM, DCM)

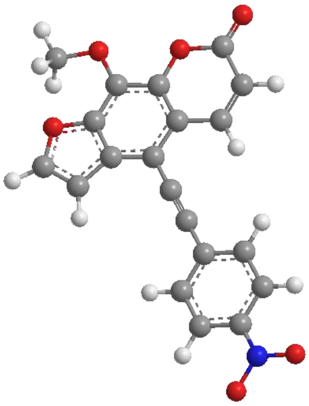

O 4.117358 1.831300 0.000034

C 3.632999 3.119152 -0.000098

O 4.437097 4.015504 -0.000130

C 2.191364 3.258034 -0.000173

C 1.383877 2.180595 -0.000134

C 1.921035 0.850033 -0.000035

C 1.106072 -0.306564 -0.000028

C 1.759484 -1.545396 0.000008

C 1.307365 -2.906166 -0.000024

C 2.420893 -3.666888 -0.000033

O 3.558841 -2.916479 -0.000027

C 3.162556 -1.617667 0.000001

C 3.993924 -0.499180 0.000018

C 3.316899 0.735034 0.000003

O 5.326626 -0.449485 0.000010

C 6.086551 -1.657371 0.000336

C -0.300617 -0.224481 -0.000020

C -1.512196 -0.167438 -0.000010

C -2.925166 -0.096696 -0.000008

C -3.695883 -1.272355 -0.000051

C -5.075758 -1.204475 -0.000049

C -5.686268 0.043350 0.000020

C -4.952789 1.223060 0.000064

C -3.573094 1.150880 0.000038

N -7.144856 0.116661 0.000060

O -7.659059 1.219284 0.000829

O -7.766721 -0.929081 -0.000724

H 1.809007 4.270469 -0.000269

H 0.305156 2.298868 -0.000187

H 0.287290 -3.256899 -0.000030

H 2.577720 -4.733906 -0.000052

H 7.126513 -1.337791 0.000452

H 5.881128 -2.248667 0.894505

H 5.881416 -2.248968 -0.893699

H -3.200648 -2.235775 -0.000096

H -5.683986 -2.099402 -0.000104

H -5.467235 2.174987 0.000116

H -2.982988 2.059360 0.000069

SCF Done: E(RPBE1PBE) = -1273.47355079 A.U. after 15 cycles

E(PBE1PBE) = -3343505.062293855 kJ/mol

Sum of electronic and zero-point Energies= -1273.299950

Sum of electronic and thermal Energies= -1273.278222

Sum of electronic and thermal Enthalpies= -1273.277278

Sum of electronic and thermal Free Energies= -1273.354057

HOMO -6.126 eV

LUMO -2.865 eV

Relevant excitation energies and oscillator strengths:

Excited State 1: Singlet-A 2.9393 eV 421.82 nm f=0.8636 <S**2>=0.000

93 -> 94 0.70107

This state for optimization and/or second-order correction.

Total Energy, E(TD-HF/TD-KS) = -1271.65185486

Copying the excited state density for this state as the 1-particle RhoCI density.

Excited State 2: Singlet-A 3.5410 eV 350.14 nm f=0.0151 <S**2>=0.000

92 -> 94 -0.16822

93 -> 95 0.66279

93 -> 96 -0.13471

Excited State 3: Singlet-A 3.7652 eV 329.29 nm f=0.2278 <S**2>=0.000

92 -> 94 0.66799

93 -> 95 0.18330

Excited State 4: Singlet-A 3.8354 eV 323.26 nm f=0.0000 <S**2>=0.000

86 -> 94 -0.14457

87 -> 94 0.58546

87 -> 95 -0.29403

87 -> 96 -0.14456

87 -> 98 -0.10559

Excited State 5: Singlet-A 4.2879 eV 289.15 nm f=0.0000 <S**2>=0.000
84 -> 94   0.60909
84 -> 95 -0.30298
84 -> 96 -0.14688
84 -> 98 -0.10534

Excited State 6: Singlet-A 4.4082 eV 281.26 nm f=0.0892 <S**2>=0.000
91 -> 94 -0.12741
92 -> 94 -0.13453
92 -> 95   0.51735
93 -> 96   0.42173

Excited State 7: Singlet-A 4.4758 eV 277.01 nm f=0.0002 <S**2>=0.000
90 -> 94   0.52784
90 -> 95   0.43063
90 -> 96 -0.11431

Excited State 8: Singlet-A 4.5620 eV 271.77 nm f=0.0024 <S**2>=0.000
89 -> 94   0.54663
91 -> 97 -0.13195
93 -> 97   0.39654

Excited State 9: Singlet-A 4.5896 eV 270.14 nm f=0.5052 <S**2>=0.000
91 -> 94 -0.23213
92 -> 95 -0.42589
93 -> 96   0.46831
93 -> 98   0.14583

Excited State 10: Singlet-A 4.6251 eV 268.07 nm f=0.0833 <S**2>=0.000
91 -> 94   0.62699
93 -> 96   0.25007

Excited State 11: Singlet-A 4.7944 eV 258.60 nm f=0.0000 <S**2>=0.000
86 -> 94   0.63417
87 -> 94   0.19227
90 -> 95   0.12618

Excited State 12: Singlet-A 4.9473 eV 250.61 nm f=0.0515 <S**2>=0.000
88 -> 94   0.67635
88 -> 95   0.12295

93 -> 98 -0.10484

Excited State 13: Singlet-A 4.9542 eV 250.26 nm f=0.0342 <S**2>=0.000
89 -> 94 -0.39050
93 -> 97   0.57605

Excited State 14: Singlet-A 5.0771 eV 244.20 nm f=0.1973 <S**2>=0.000
91 -> 95 -0.13066
92 -> 95   0.10703
92 -> 96 -0.33124
93 -> 98   0.55785
93 -> 99   0.12509

Excited State 15: Singlet-A 5.2193 eV 237.55 nm f=0.0001 <S**2>=0.000
86 -> 94 -0.12831
90 -> 94 -0.46320
90 -> 95   0.46972
90 -> 96 -0.15145

Excited State 16: Singlet-A 5.2595 eV 235.73 nm f=0.0131 <S**2>=0.000
91 -> 95   0.55437
92 -> 96 -0.39666

Excited State 17: Singlet-A 5.3912 eV 229.98 nm f=0.2406 <S**2>=0.000
91 -> 95   0.38256
92 -> 95   0.10668
92 -> 96   0.43776
93 -> 98   0.34136
93 -> 99 -0.11013

Excited State 18: Singlet-A 5.5748 eV 222.40 nm f=0.0000 <S**2>=0.000
91 ->100 -0.10679
92 ->100   0.10326
93 ->100   0.67987

Excited State 19: Singlet-A 5.6196 eV 220.63 nm f=0.0029 <S**2>=0.000
85 -> 94 -0.29016
89 -> 94   0.14618
89 -> 95   0.61689

Excited State 20: Singlet-A 5.6647 eV 218.87 nm f=0.0102 <S**2>=0.000

83 -> 94   0.11448
88 -> 94 -0.13134
88 -> 95   0.67019

Excited State 21: Singlet-A 5.7048 eV 217.33 nm f=0.0000 <S**2>=0.000
86 -> 94 -0.17819
86 -> 95   0.34453
87 -> 94   0.21237
87 -> 95   0.52290

Excited State 22: Singlet-A 5.7110 eV 217.10 nm f=0.0142 <S**2>=0.000
85 -> 94   0.60355
85 -> 95   0.12028
89 -> 95   0.29840

Excited State 23: Singlet-A 5.8176 eV 213.12 nm f=0.0771 <S**2>=0.000
83 -> 94 -0.11817
91 -> 96 -0.14024
92 -> 96   0.14601
92 -> 98   0.10962
93 -> 99   0.62638

Excited State 24: Singlet-A 5.8263 eV 212.80 nm f=0.0000 <S**2>=0.000
86 -> 95   0.57687
87 -> 94 -0.25507
87 -> 95 -0.22951
87 -> 96 -0.14078
87 -> 98 -0.11193

8.12. XYZ-coordinates of the S_0 state of compound 13c (PBE1PBE/6-311G(d,p)) and TD-DFT calculation (PBE1PBE/6-21G, SCRF(IEFPCM, DCM)

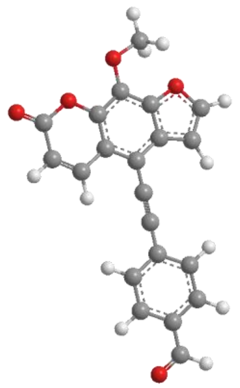

O 3.739838 1.879966 0.000028
C 3.225930 3.156160 -0.000100
O 4.009550 4.070766 -0.000028
C 1.781698 3.261980 -0.000293
C 0.999305 2.166035 -0.000317
C 1.566352 0.848185 -0.000136
C 0.777252 -0.325928 -0.000140
C 1.459178 -1.549038 -0.000024
C 1.037895 -2.920010 0.000035
C 2.168056 -3.655474 0.000173
O 3.288961 -2.879345 0.000221
C 2.863612 -1.589614 0.000096
C 3.669887 -0.452806 0.000129
C 2.965029 0.764983 -0.000002
O 5.002048 -0.373373 0.000283
C 5.787399 -1.564417 0.000151
C -0.631688 -0.275331 -0.000196
C -1.844296 -0.244903 -0.000225
C -3.259596 -0.202746 -0.000175
C -4.006462 -1.392088 -0.000681
C -5.388997 -1.340458 -0.000594
C -6.050876 -0.111477 -0.000003
C -5.310787 1.075176 0.000491
C -3.931923 1.034789 0.000410
C -7.521872 -0.075064 0.000133
O -8.184139 0.937983 0.000582
H 1.376238 4.265386 -0.000424
H -0.081932 2.258829 -0.000479

H 0.025834 -3.293259 -0.000016
H 2.349175 -4.718645 0.000244
H 6.820473 -1.223029 0.000183
H 5.594953 -2.160520 0.894083
H 5.594945 -2.160324 -0.893912
H -3.490614 -2.345155 -0.001146
H -5.965601 -2.260986 -0.000994
H -5.840697 2.021561 0.000948
H -3.354456 1.952167 0.000813
H -8.008948 -1.071713 -0.000375
SCF Done: E(RPBE1PBE) = -1182.38416193 A.U. after 15 cycles
E(PBE1PBE) = -3104349.8536240472
HOMO -6.057 eV
LUMO -2.556 eV

Relevant excitation energies and oscillator strengths:

Excited State 1: Singlet-A 3.0674 eV 404.19 nm f=0.8747 <S**2>=0.000
89 -> 90   0.69786
This state for optimization and/or second-order correction.
Total Energy, E(TD-HF/TD-KS) = -1180.72466742
Copying the excited state density for this state as the 1-particle RhoCI density.

Excited State 2: Singlet-A 3.5773 eV 346.58 nm f=0.0000 <S**2>=0.000
87 -> 90   0.57723
87 -> 91 -0.34219
87 -> 92 -0.15343
87 -> 94 -0.10585
87 -> 95   0.10584

Excited State 3: Singlet-A 3.6808 eV 336.84 nm f=0.2112 <S**2>=0.000
88 -> 90 -0.22409
89 -> 91   0.64814
89 -> 92 -0.10941

Excited State 4: Singlet-A 3.9099 eV 317.10 nm f=0.2169 <S**2>=0.000
88 -> 90   0.64560
89 -> 91   0.23657
89 -> 92   0.10196

Excited State 5: Singlet-A 4.5092 eV 274.96 nm f=0.0003 <S**2>=0.000
84 -> 90   0.57830
84 -> 91   0.37237
84 -> 94 -0.11498

Excited State 6: Singlet-A 4.5935 eV 269.91 nm f=0.0769 <S**2>=0.000
85 -> 90   0.14421
86 -> 90   0.40986
88 -> 90   0.11540
88 -> 91 -0.39590
89 -> 92 -0.34572

Excited State 7: Singlet-A 4.6351 eV 267.49 nm f=0.0065 <S**2>=0.000
85 -> 90   0.53957
85 -> 91 -0.13546
86 -> 93 -0.14287
88 -> 91   0.17080
89 -> 93   0.34543

Excited State 8: Singlet-A 4.6811 eV 264.86 nm f=0.1592 <S**2>=0.000
86 -> 90   0.52064
88 -> 91   0.35038
89 -> 92   0.19091
89 -> 94 -0.17413

Excited State 9: Singlet-A 4.7615 eV 260.39 nm f=0.4537 <S**2>=0.000
88 -> 91 -0.40634
89 -> 92   0.53908

Excited State 10: Singlet-A 4.8581 eV 255.21 nm f=0.0000 <S**2>=0.000
82 -> 90   0.68068
84 -> 91   0.11881

Excited State 11: Singlet-A 5.0630 eV 244.88 nm f=0.0001 <S**2>=0.000
87 -> 90   0.40054
87 -> 91   0.54535

87 -> 92 0.14097

Excited State 12: Singlet-A 5.0758 eV 244.27 nm f=0.0267 <S**2>=0.000
83 -> 90 0.67608

Excited State 13: Singlet-A 5.1299 eV 241.69 nm f=0.0233 <S**2>=0.000
83 -> 90 -0.11133
85 -> 90 -0.35965
89 -> 93 0.57994

Excited State 14: Singlet-A 5.2325 eV 236.95 nm f=0.0791 <S**2>=0.000
88 -> 92 -0.39805
89 -> 94 0.53110

Excited State 15: Singlet-A 5.4374 eV 228.02 nm f=0.0229 <S**2>=0.000
86 -> 90 -0.11514
86 -> 91 0.66650
89 -> 94 -0.10913

Excited State 16: Singlet-A 5.4804 eV 226.23 nm f=0.0001 <S**2>=0.000
82 -> 90 -0.13314
84 -> 90 -0.39901
84 -> 91 0.53266
84 -> 92 -0.11061
84 -> 94 -0.14394

Excited State 17: Singlet-A 5.5858 eV 221.96 nm f=0.0000 <S**2>=0.000
88 -> 96 0.10141
89 -> 96 0.68304

Excited State 18: Singlet-A 5.6036 eV 221.26 nm f=0.3693 <S**2>=0.000
85 -> 91 -0.10284
86 -> 91 0.15024
88 -> 92 0.53601
89 -> 94 0.36207

Excited State 19: Singlet-A 5.6261 eV 220.37 nm f=0.0240 <S**2>=0.000

85 -> 90 0.14404

85 -> 91 0.64721

86 -> 93 0.12489

89 -> 93 0.11514

Excited State 20: Singlet-A 5.8785 eV 210.91 nm f=0.0001 <S**2>=0.000

82 -> 91 0.69337

Excited State 21: Singlet-A 5.8828 eV 210.76 nm f=0.0523 <S**2>=0.000

81 -> 90 -0.11263

83 -> 90 -0.10588

83 -> 91 0.67451

Excited State 22: Singlet-A 5.9513 eV 208.33 nm f=0.0054 <S**2>=0.000

81 -> 90 0.64158

88 -> 93 0.10337

88 -> 94 -0.16189

Excited State 23: Singlet-A 5.9800 eV 207.33 nm f=0.0001 <S**2>=0.000

87 -> 91 0.12988

87 -> 92 -0.27094

87 -> 93 0.63129

Excited State 24: Singlet-A 6.0593 eV 204.62 nm f=0.0297 <S**2>=0.000

81 -> 90 -0.10135

85 -> 91 0.10627

88 -> 93 0.68473

8.13. XYZ-coordinates of the S_0 state of compound 13d (PBE1PBE/6-311G(d,p)) and TD-DFT calculation (PBE1PBE/6-21G, SCRF(IEFPCM, DCM)

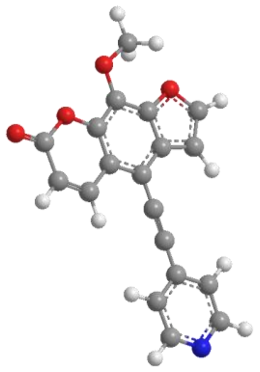

O -3.141768 1.787450 0.000337

C -2.685887 3.085303 -0.000285

O -3.509592 3.963918 -0.001088

C -1.247697 3.256136 0.000219

C -0.416477 2.196849 0.000449

C -0.923789 0.854605 0.000298

C -0.083697 -0.283193 0.000193

C -0.709660 -1.535652 0.000037

C -0.227342 -2.886349 -0.000091

C -1.323399 -3.671665 -0.000244

O -2.478168 -2.946657 -0.000133

C -2.110907 -1.639296 0.000027

C -2.966791 -0.539603 0.000104

C -2.317287 0.708689 0.000222

O -4.300994 -0.519397 0.000071

C -5.033718 -1.743533 0.000130

C 1.322325 -0.171077 0.000153

C 2.531972 -0.091924 0.000128

C 3.945952 0.001138 0.000038

C 4.749191 -1.146714 -0.000289

C 6.126806 -0.995687 -0.000369

N 6.749717 0.184397 -0.000149

C 5.978572 1.273381 0.000162

C 4.592903 1.243808 0.000270

H -0.887740 4.276752 0.000229

H 0.659365 2.338912 0.000723

H 0.800431 -3.213844 -0.000113

H -1.456576 -4.741880 -0.000383

H -6.080595 -1.447193 0.000154

H -4.815903 -2.330749 -0.893875

H -4.815846 -2.330688 0.894163

H 4.302534 -2.133729 -0.000488

H 6.765284 -1.875570 -0.000621

H 6.496939 2.228932 0.000335

H 4.021838 2.164487 0.000523

SCF Done: E(RPBE1PBE) = -1085.18870656 A.U. after 15 cycles

E(PBE1PBE) = -2849163.166111021

Sum of electronic and zero-point Energies= -1084.987522

Sum of electronic and thermal Energies= -1084.968447

Sum of electronic and thermal Enthalpies= -1084.967502

Sum of electronic and thermal Free Energies= -1085.037537

HOMO -6.120 eV

LUMO -2.379 eV

Relevant excitation energies and oscillator strengths:

Excited State 1: Singlet-A 3.2310 eV 383.74 nm f=0.5352 <S**2>=0.000

82 -> 83 0.69431

This state for optimization and/or second-order correction.

Total Energy, E(TD-HF/TD-KS) = -1083.64914880

Copying the excited state density for this state as the 1-particle RhoCI density.

Excited State 2: Singlet-A 3.9328 eV 315.25 nm f=0.4399 <S**2>=0.000

81 -> 83 0.50298

82 -> 84 0.47363

Excited State 3: Singlet-A 4.1364 eV 299.74 nm f=0.2577 <S**2>=0.000

81 -> 83 -0.46074

82 -> 84 0.50666

82 -> 85   0.12162

Excited State 4: Singlet-A 4.2025 eV 295.02 nm f=0.0025 <S**2>=0.000
80 -> 83   0.63906
80 -> 84   0.25794
80 -> 85   0.13563

Excited State 5: Singlet-A 4.5249 eV 274.00 nm f=0.0003 <S**2>=0.000
77 -> 83   0.63372
77 -> 84 -0.27726
77 -> 87   0.11586

Excited State 6: Singlet-A 4.8604 eV 255.09 nm f=0.0022 <S**2>=0.000
78 -> 83   0.47245
78 -> 84   0.13343
79 -> 86 -0.11437
82 -> 86   0.48005

Excited State 7: Singlet-A 4.9186 eV 252.07 nm f=0.1661 <S**2>=0.000
79 -> 83   0.53392
82 -> 85 -0.40139
82 -> 87   0.16568

Excited State 8: Singlet-A 4.9393 eV 251.02 nm f=0.0747 <S**2>=0.000
81 -> 84   0.68850

Excited State 9: Singlet-A 4.9683 eV 249.55 nm f=0.0000 <S**2>=0.000
75 -> 83   0.67868
75 -> 84   0.12965
77 -> 84 -0.10308

Excited State 10: Singlet-A 4.9926 eV 248.34 nm f=0.3310 <S**2>=0.000
76 -> 83 -0.12305
79 -> 83   0.41000
81 -> 83   0.13343
81 -> 85   0.10600
82 -> 85   0.51860

Excited State 11: Singlet-A 5.1974 eV 238.55 nm f=0.0561 <S**2>=0.000
76 -> 83   0.59818
78 -> 83 -0.23270
82 -> 86   0.23253

Excited State 12: Singlet-A 5.2018 eV 238.35 nm f=0.0452 <S**2>=0.000
76 -> 83 -0.31398
78 -> 83 -0.43907
82 -> 86   0.43957

Excited State 13: Singlet-A 5.2803 eV 234.80 nm f=0.0002 <S**2>=0.000
80 -> 83 -0.12479
80 -> 84   0.25749
80 -> 86   0.64086

Excited State 14: Singlet-A 5.2836 eV 234.66 nm f=0.0011 <S**2>=0.000
80 -> 83 -0.26879
80 -> 84   0.55109
80 -> 85   0.16415
80 -> 86 -0.29715

Excited State 15: Singlet-A 5.5430 eV 223.68 nm f=0.0063 <S**2>=0.000
81 -> 85 -0.38050
82 -> 85   0.12738
82 -> 87   0.54932

Excited State 16: Singlet-A 5.6265 eV 220.36 nm f=0.0000 <S**2>=0.000
79 -> 88 -0.11888
81 -> 88   0.10568
82 -> 88   0.68104

Excited State 17: Singlet-A 5.7626 eV 215.15 nm f=0.0002 <S**2>=0.000
75 -> 83   0.11834
77 -> 83   0.29935
77 -> 84   0.60249
77 -> 87 -0.16810

Excited State 18: Singlet-A 5.8516 eV 211.88 nm f=0.2643 <S**2>=0.000
74 -> 83 0.16382
79 -> 84 0.29975
81 -> 85 0.50746
82 -> 87 0.29431

Excited State 19: Singlet-A 5.9072 eV 209.89 nm f=0.0174 <S**2>=0.000
78 -> 83 -0.14002
78 -> 84 0.55113
78 -> 85 0.11643
79 -> 86 -0.15959
81 -> 86 0.31561
82 -> 86 -0.13494

Excited State 20: Singlet-A 5.9600 eV 208.03 nm f=0.0346 <S**2>=0.000
74 -> 83 -0.21286
79 -> 84 0.59944
81 -> 85 -0.15913
82 -> 87 -0.19951

Excited State 21: Singlet-A 6.0933 eV 203.48 nm f=0.0193 <S**2>=0.000
78 -> 84 -0.31049
81 -> 86 0.62638

Excited State 22: Singlet-A 6.1175 eV 202.67 nm f=0.0001 <S**2>=0.000
75 -> 83 -0.12856
75 -> 84 0.67704
75 -> 85 0.10283

Excited State 23: Singlet-A 6.1989 eV 200.01 nm f=0.1747 <S**2>=0.000
76 -> 84 0.67039
81 -> 85 -0.11802

Excited State 24: Singlet-A 6.2602 eV 198.05 nm f=0.0008 <S**2>=0.000
80 -> 84 -0.24085
80 -> 85 0.64770

80 -> 87 0.12033

8.14. XYZ-coordinates of the S_0 state of compound 13e (PBE1PBE/6-311G(d,p)) and TD-DFT calculation (PBE1PBE/6-21G, SCRF(IEFPCM, DCM)

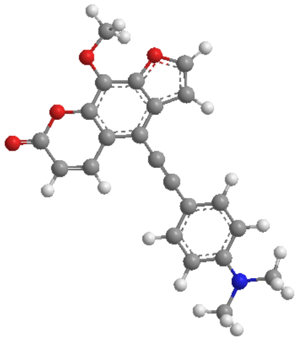

N -7.024871 0.107416 -0.007409

C -5.663664 0.033415 -0.004087

C -4.863476 1.199212 -0.008323

C -3.486511 1.118743 -0.007517

C -2.822931 -0.119470 -0.001665

C -3.615287 -1.279348 0.004091

C -4.993279 -1.211502 0.003420

C -7.818723 -1.100816 0.011191

C -7.682251 1.395035 0.000120

C -0.196957 -0.245092 -0.000487

C -1.410379 -0.191702 -0.000922

C 3.715045 3.127859 0.002177

C 2.273715 3.254660 0.002196

C 1.476522 2.168156 0.001448

C 2.022103 0.842918 0.000579

C 1.212062 -0.316923 -0.000208

C 1.880218 -1.547918 -0.000810

C 3.283898 -1.609865 -0.000739

C 4.109018 -0.485769 -0.000030

C 3.422203 0.738490 0.000623

O 4.211521 1.845510 0.001334

O 3.689216 -2.906372 -0.001574

C 1.438112 -2.913619 -0.001781

C 2.555545 -3.666934 -0.002193

O 4.512787 4.031705 0.002850
O 5.447142 -0.427183 -0.000004
C 6.208530 -1.631412 0.000342
H -5.326288 2.177599 -0.012228
H -2.902486 2.033079 -0.011154
H -3.132536 -2.250908 0.009699
H -5.558887 -2.134343 0.008968
H -7.631205 -1.699251 0.911316
H -8.873914 -0.833601 -0.000701
H -7.620523 -1.730463 -0.864520
H -7.419306 1.991029 -0.882192
H -8.760228 1.244102 -0.007045
H -7.428106 1.978742 0.893626
H 1.881995 4.263521 0.002891
H 0.396415 2.275208 0.001536
H 0.419882 -3.269959 -0.002147
H 2.721438 -4.732527 -0.002915
H 7.248615 -1.310960 0.000818
H 6.006001 -2.225583 0.893796
H 6.006820 -2.225591 -0.893287
SCF Done: E(RPBE1PBE) = -1202.96882846 A.U. after 14 cycles
E(PBE1PBE) = -3158394.8997154953
Sum of electronic and zero-point Energies= -1202.722476
Sum of electronic and thermal Energies= -1202.698628
Sum of electronic and thermal Enthalpies= -1202.697684
Sum of electronic and thermal Free Energies= -1202.779874
HOMO -5.337 eV
LUMO -2.051 eV
Relevant excitation energies and oscillator strengths:
Excited State 1: Singlet-A 2.8029 eV 442.35 nm f=0.6549 <S**2>=0.000

94 -> 95 0.70093

This state for optimization and/or second-order correction.

Total Energy, E(TD-HF/TD-KS) = -1201.34907256

Copying the excited state density for this state as the 1-particle RhoCI density.

Excited State 2: Singlet-A 3.6808 eV 336.84 nm f=0.1754 <S**2>=0.000

92 -> 95 -0.14549

93 -> 95   0.57590

94 -> 96 -0.35212

Excited State 3: Singlet-A 3.8452 eV 322.43 nm f=0.6012 <S**2>=0.000

92 -> 95   0.10403

93 -> 95   0.37805

94 -> 96   0.58123

Excited State 4: Singlet-A 4.2868 eV 289.22 nm f=0.2774 <S**2>=0.000

92 -> 95   0.62648

93 -> 97 -0.10482

94 -> 96 -0.15797

94 -> 97   0.19598

94 -> 99   0.11213

Excited State 5: Singlet-A 4.4768 eV 276.95 nm f=0.0346 <S**2>=0.000

91 -> 95   0.17344

91 -> 96 -0.10171

93 -> 98   0.10296

94 -> 98   0.65869

Excited State 6: Singlet-A 4.5794 eV 270.74 nm f=0.2707 <S**2>=0.000

92 -> 95 -0.17058

93 -> 96   0.27349

94 -> 97   0.61082

Excited State 7: Singlet-A 4.6037 eV 269.32 nm f=0.0003 <S**2>=0.000

89 -> 95   0.65609

89 -> 96   0.17489

89 -> 97 -0.10261

Excited State 8: Singlet-A 4.8574 eV 255.25 nm f=0.2261 <S**2>=0.000

90 -> 95   0.13169

92 -> 95   0.15090

93 -> 96   0.62384

94 -> 97 -0.21841

Excited State 9: Singlet-A 5.0463 eV 245.69 nm f=0.0028 <S**2>=0.000

87 -> 95   0.62915

87 -> 96 -0.10278

89 -> 95 -0.10993

91 -> 95   0.21656

94 ->100   0.11569

Excited State 10: Singlet-A 5.0474 eV 245.64 nm f=0.0256 <S**2>=0.000

87 -> 95 -0.21064

91 -> 95   0.64595

94 -> 98 -0.16716

Excited State 11: Singlet-A 5.1717 eV 239.74 nm f=0.0898 <S**2>=0.000

90 -> 95 -0.10628

92 -> 96   0.61920

94 -> 99 -0.26892

Excited State 12: Singlet-A 5.2360 eV 236.79 nm f=0.0001 <S**2>=0.000

87 -> 95 -0.12828

90 ->100 -0.10182

94 ->100   0.66975

Excited State 13: Singlet-A 5.2435 eV 236.45 nm f=0.0083 <S**2>=0.000

90 -> 95   0.65403

94 -> 97   0.12518

94 -> 99 -0.10816

Excited State 14: Singlet-A 5.3957 eV 229.78 nm f=0.0187 <S**2>=0.000

88 -> 95   0.63428

92 -> 96   0.12236

94 -> 99   0.22547

Excited State 15: Singlet-A 5.4349 eV 228.13 nm f=0.0196 <S**2>=0.000

88 -> 95 -0.26513

92 -> 96   0.23574

93 -> 97   0.14409

94 -> 99   0.56768

Excited State 16: Singlet-A 5.5702 eV 222.58 nm f=0.2367 <S**2>=0.000

90 -> 95 -0.11210

92 -> 95   0.10754

92 -> 96 -0.14816

93 -> 97   0.64997

Excited State 17: Singlet-A 5.5886 eV 221.85 nm f=0.0039 <S**2>=0.000

93 -> 98   0.68398

94 -> 98 -0.11734

Excited State 18: Singlet-A 6.0215 eV 205.90 nm f=0.0065 <S**2>=0.000

86 -> 95   0.20028

88 -> 96 -0.13339

90 -> 96 -0.14616

92 -> 97 -0.39700

93 -> 99 -0.11614

94 ->101   0.46724

Excited State 19: Singlet-A 6.0547 eV 204.77 nm f=0.1132 <S**2>=0.000

86 -> 95 -0.14886

92 -> 97   0.48963

94 ->101   0.44902

Excited State 20: Singlet-A 6.0709 eV 204.23 nm f=0.0502 <S**2>=0.000

91 -> 96   0.67071

94 -> 98   0.10114

Excited State 21: Singlet-A 6.1005 eV 203.24 nm f=0.0000 <S**2>=0.000

87 -> 95 -0.16194

87 -> 96 -0.29013

89 -> 95 -0.12899

89 -> 96   0.59375

Excited State 22: Singlet-A 6.2098 eV 199.66 nm f=0.0110 <S**2>=0.000

86 -> 95   0.13166

90 -> 96 -0.39677

93 -> 99   0.53876

Excited State 23: Singlet-A 6.2404 eV 198.68 nm f=0.0002 <S**2>=0.000

87 -> 96   0.60648

87 -> 97 -0.11076

89 -> 95 -0.16426

89 -> 96   0.26570

89 -> 99 -0.11576

Excited State 24: Singlet-A 6.2858 eV 197.24 nm f=0.0632 <S**2>=0.000

86 -> 95   0.25644

90 -> 96   0.52616

93 -> 99   0.33279

94 ->101   0.15397

8.15. XYZ-coordinates of the S_0 state of compound 15a (PBE1PBE/6-311G(d,p)) and TD-DFT calculation (PBE1PBE/6-21G, SCRF(IEFPCM, DCM)

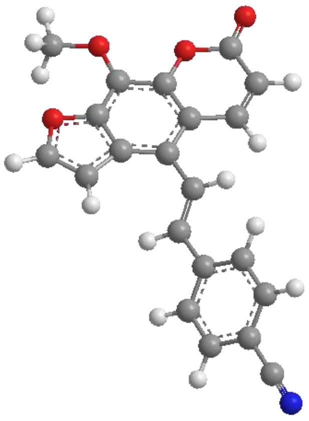

O 3.887795 1.652610 0.096501

C 3.582383 2.991003 0.131140

O 4.498045 3.770885 0.205475

C 2.174698 3.318700 0.080508

C 1.233858 2.359099 -0.012663

C 1.578169 0.964691 -0.076015

C 0.615409 -0.070848 -0.160404

C 1.115154 -1.381138 -0.189905

C 0.519885 -2.682243 -0.360054

C 1.535444 -3.567498 -0.359043

O 2.746077 -2.960820 -0.208683

C 2.498694 -1.629778 -0.114511

C 3.464296 -0.632646 -0.008225

C 2.951140 0.672697 0.000463

O 4.794235 -0.763219 0.059130

C 5.369295 -2.046011 0.297445

C -0.805629 0.232092 -0.245637

C -1.798666 -0.530716 0.247055

C -3.225855 -0.251127 0.145117

C -4.117680 -1.025038 0.903054

C -5.481875 -0.802764 0.859870

C -5.991577 0.210201 0.041910

C -5.117963 0.987205 -0.730217

C -3.758242 0.753440 -0.679505

C -7.395171 0.447195 -0.013054

N -8.533577 0.640130 -0.057571
H 1.924739 4.370560 0.128266
H 0.189351 2.647748 -0.029734
H -0.522743 -2.924428 -0.484266
H 1.569314 -4.641016 -0.456462
H 6.433997 -1.861167 0.426635
H 4.961218 -2.494588 1.206155
H 5.210561 -2.714657 -0.549973
H -1.067864 1.163672 -0.739313
H -1.545496 -1.420434 0.816897
H -3.727136 -1.811811 1.540210
H -6.155691 -1.408359 1.454569
H -5.514909 1.765078 -1.372008
H -3.104100 1.353744 -1.301340
SCF Done: E(RPBE1PBE) = -1162.51613189 A.U. after 15 cycles
E(PBE1PBE) = -3052186.3367804214
Sum of electronic and zero-point Energies= -1162.331464
Sum of electronic and thermal Energies= -1162.310267
Sum of electronic and thermal Enthalpies= -1162.30932
Sum of electronic and thermal Free Energies= -1162.384224
HOMO -5.994 eV
LUMO -2.412 eV
Relevant excitation energies and oscillator strengths:
Excited State 1: Singlet-A 3.0772 eV 402.91 nm f=0.7491 <S**2>=0.000
89 -> 90 0.69996
This state for optimization and/or second-order correction.
Total Energy, E(TD-HF/TD-KS) = -1160.94688875
Copying the excited state density for this state as the 1-particle RhoCI density.

Excited State 2: Singlet-A 3.6782 eV 337.08 nm f=0.2324 <S**2>=0.000
88 -> 90 0.20308
89 -> 91 0.66030

Excited State 3: Singlet-A 3.9161 eV 316.60 nm f=0.2546 <S**2>=0.000
88 -> 90 0.65474
89 -> 91 -0.20854
89 -> 92 -0.11739

Excited State 4: Singlet-A 4.5041 eV 275.27 nm f=0.1369 <S**2>=0.000
86 -> 90 -0.22763
86 -> 91   0.15110
87 -> 90   0.59134
88 -> 91   0.19213
89 -> 92   0.10611

Excited State 5: Singlet-A 4.5533 eV 272.29 nm f=0.0272 <S**2>=0.000
86 -> 90   0.52984
86 -> 91 -0.34197
86 -> 94   0.10453
87 -> 90   0.25070

Excited State 6: Singlet-A 4.6671 eV 265.66 nm f=0.0121 <S**2>=0.000
85 -> 90   0.37393
85 -> 91   0.12111
87 -> 93 -0.13657
88 -> 91 -0.25182
89 -> 93   0.49667

Excited State 7: Singlet-A 4.6781 eV 265.03 nm f=0.0935 <S**2>=0.000
85 -> 90   0.15617
87 -> 90 -0.21243
88 -> 91   0.52512
89 -> 92   0.28692
89 -> 93   0.15132
89 -> 94 -0.10989

Excited State 8: Singlet-A 4.7927 eV 258.69 nm f=0.3965 <S**2>=0.000
87 -> 91   0.10013
88 -> 90   0.11422
88 -> 91 -0.31514
89 -> 92   0.57827
89 -> 93 -0.11755

Excited State 9: Singlet-A 5.0820 eV 243.97 nm f=0.0175 <S**2>=0.000

84 -> 90   0.67281

Excited State 10: Singlet-A 5.0892 eV 243.62 nm f=0.0338 <S**2>=0.000
84 -> 90 -0.10727
85 -> 90   0.50548
87 -> 93 -0.11927
89 -> 93 -0.44554

Excited State 11: Singlet-A 5.2861 eV 234.55 nm f=0.1472 <S**2>=0.000
87 -> 91   0.37577
88 -> 92 -0.30263
89 -> 94   0.48017

Excited State 12: Singlet-A 5.3320 eV 232.53 nm f=0.0438 <S**2>=0.000
87 -> 90   0.11387
87 -> 91   0.56178
88 -> 92   0.12175
89 -> 92 -0.14062
89 -> 94 -0.33481

Excited State 13: Singlet-A 5.5359 eV 223.96 nm f=0.0010 <S**2>=0.000
86 -> 90   0.39159
86 -> 91   0.55077
86 -> 94 -0.14094

Excited State 14: Singlet-A 5.6671 eV 218.78 nm f=0.2740 <S**2>=0.000
83 -> 90 -0.24085
84 -> 91   0.11805
88 -> 92   0.53991
89 -> 94   0.28485
89 -> 95 -0.13594

Excited State 15: Singlet-A 5.7489 eV 215.67 nm f=0.0057 <S**2>=0.000
85 -> 90 -0.21780
85 -> 91   0.50881
87 -> 93 -0.21282
88 -> 93   0.34571

Excited State 16: Singlet-A 5.8682 eV 211.28 nm f=0.0065 <S**2>=0.000
83 -> 90   0.46333
84 -> 91   0.45086
88 -> 92   0.10722
89 -> 95   0.17668

Excited State 17: Singlet-A 5.9019 eV 210.08 nm f=0.1589 <S**2>=0.000
83 -> 90 -0.34751
84 -> 91   0.48350
88 -> 92 -0.23802
88 -> 93 -0.13558
89 -> 94 -0.10893
89 -> 95 -0.13646

Excited State 18: Singlet-A 5.9424 eV 208.64 nm f=0.0765 <S**2>=0.000
84 -> 91   0.14398
85 -> 91 -0.31269
87 -> 93   0.13912
88 -> 93   0.58670

Excited State 19: Singlet-A 6.0352 eV 205.43 nm f=0.0293 <S**2>=0.000
82 -> 90 -0.16208
83 -> 90 -0.20157
87 -> 92 -0.39275
88 -> 94 -0.11069
89 -> 95   0.48537

Excited State 20: Singlet-A 6.1304 eV 202.24 nm f=0.0349 <S**2>=0.000
82 -> 90 -0.35568
87 -> 92   0.51099
88 -> 94 -0.13861
89 -> 95   0.24523

Excited State 21: Singlet-A 6.2081 eV 199.71 nm f=0.0076 <S**2>=0.000
82 -> 90   0.37693
83 -> 90 -0.14272

87 -> 92 0.19780

88 -> 94 0.39689

89 -> 95 0.30861

89 -> 97 0.10942

Excited State 22: Singlet-A 6.2939 eV 196.99 nm f=0.0037 <S**2>=0.000

78 -> 90 -0.14288

81 -> 90 0.63833

81 -> 91 -0.12591

Excited State 23: Singlet-A 6.3649 eV 194.79 nm f=0.0025 <S**2>=0.000

80 -> 90 0.38918

80 -> 91 0.16992

87 -> 96 -0.16799

89 -> 96 0.49442

Excited State 24: Singlet-A 6.4047 eV 193.58 nm f=0.1164 <S**2>=0.000

85 -> 90 0.10653

85 -> 91 0.31092

85 -> 92 -0.19183

87 -> 93 0.56223

8.16. XYZ-coordinates of the S_0 state of compound 15b (PBE1PBE/6-311G(d,p)) and TD-DFT calculation (PBE1PBE/6-21G, SCRF(IEFPCM, DCM)

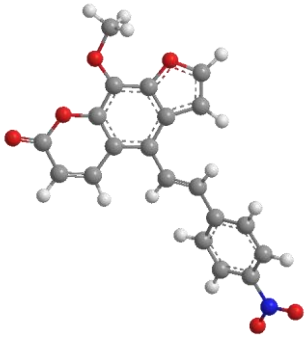

O 4.264899 1.676281 0.125818

C 3.943901 3.010519 0.176421

O 4.850016 3.800164 0.261640

C 2.532373 3.321912 0.127540

C 1.603096 2.352487 0.022097
C 1.964091 0.962928 -0.057198
C 1.014272 -0.084069 -0.154782
C 1.530813 -1.387802 -0.200577
C 0.952139 -2.694047 -0.385926
C 1.978756 -3.566649 -0.393231
O 3.181097 -2.946987 -0.234395
C 2.917278 -1.620072 -0.126460
C 3.870032 -0.612002 -0.007823
C 3.339968 0.686987 0.016783
O 5.200607 -0.723792 0.063094
C 5.799778 -2.007988 0.221979
C -0.409089 0.202750 -0.235134
C -1.395539 -0.583817 0.234918
C -2.823671 -0.314486 0.136972
C -3.709431 -1.121385 0.869490
C -5.075710 -0.911873 0.829640
C -5.568212 0.118675 0.040995
C -4.724751 0.933138 -0.707026
C -3.362993 0.710781 -0.659131
N -7.006895 0.348473 -0.011127
O -7.414697 1.254343 -0.714892
O -7.724993 -0.376772 0.652464
H 2.269698 4.370074 0.186973
H 0.555524 2.629595 0.006987
H -0.086872 -2.948340 -0.515353
H 2.025839 -4.638547 -0.502367
H 5.640443 -2.630135 -0.660160
H 5.411708 -2.513946 1.108712
H 6.862826 -1.811851 0.347463
H -0.681786 1.144177 -0.703492
H -1.135358 -1.485199 0.782294
H -3.312333 -1.923025 1.483240
H -5.760216 -1.529942 1.395692
H -5.145956 1.719967 -1.318992
H -2.713288 1.336049 -1.260221
SCF Done: E(RPBE1PBE) = -1274.68294160 A.U. after 14 cycles

E(PBE1PBE) = -3346680.3181073884 kJ/mol

Sum of electronic and zero-point Energies=   -1274.520101

Sum of electronic and thermal Energies=   -1274.498141

Sum of electronic and thermal Enthalpies=   -1274.497196

Sum of electronic and thermal Free Energies= -1274.574700

HOMO -6.025 eV

LUMO -2.790 eV

Relevant excitation energies and oscillator strengths:

Excited State 1: Singlet-A 2.8833 eV 430.01 nm f=0.7748 <S**2>=0.000

94 -> 95   0.70270

This state for optimization and/or second-order correction.

Total Energy, E(TD-HF/TD-KS) = -1272.89216076

Copying the excited state density for this state as the 1-particle RhoCI density.

Excited State 2: Singlet-A 3.4796 eV 356.31 nm f=0.0049 <S**2>=0.000

93 -> 95   0.14227

94 -> 96   0.66656

94 -> 97   0.14359

Excited State 3: Singlet-A 3.7294 eV 332.45 nm f=0.2550 <S**2>=0.000

93 -> 95   0.67524

94 -> 96 -0.15757

Excited State 4: Singlet-A 3.8453 eV 322.43 nm f=0.0000 <S**2>=0.000

88 -> 95   0.59969

88 -> 96   0.30294

88 -> 97 -0.18552

Excited State 5: Singlet-A 4.2996 eV 288.36 nm f=0.0295 <S**2>=0.000

86 -> 95   0.54058

86 -> 96   0.26566

86 -> 97 -0.15895

93 -> 96 -0.14782

94 -> 97   0.26380

Excited State 6: Singlet-A 4.3060 eV 287.93 nm f=0.1021 <S**2>=0.000

86 -> 95 -0.27194

86 -> 96 -0.13402
92 -> 95 -0.11226
93 -> 96 -0.28835
94 -> 97 0.52259

Excited State 7: Singlet-A 4.4165 eV 280.73 nm f=0.1129 <S**2>=0.000
92 -> 95 0.59851
93 -> 96 0.19305
94 -> 97 0.21032

Excited State 8: Singlet-A 4.4800 eV 276.75 nm f=0.2661 <S**2>=0.000
90 -> 95 0.14044
92 -> 95 -0.28999
93 -> 96 0.52685
94 -> 97 0.23299
94 -> 98 -0.14478
94 -> 99 -0.12782

Excited State 9: Singlet-A 4.5148 eV 274.62 nm f=0.0111 <S**2>=0.000
91 -> 95 0.48859
91 -> 96 -0.44400
91 -> 97 -0.13300
92 -> 95 0.12483

Excited State 10: Singlet-A 4.5327 eV 273.53 nm f=0.0663 <S**2>=0.000
90 -> 95 0.54309
92 -> 98 0.12831
93 -> 96 -0.15537
94 -> 97 -0.15517
94 -> 98 -0.30611

Excited State 11: Singlet-A 4.8904 eV 253.53 nm f=0.0839 <S**2>=0.000
89 -> 95 0.30836
90 -> 95 0.23650
92 -> 96 -0.11070
93 -> 96 0.11454
94 -> 98 0.49459

94 -> 99   0.22403

Excited State 12: Singlet-A 4.9152 eV 252.25 nm f=0.0218 <S**2>=0.000
89 -> 95   0.55476
89 -> 96 -0.10287
90 -> 95 -0.23490
94 -> 98 -0.31014
94 -> 99   0.11526

Excited State 13: Singlet-A 4.9914 eV 248.40 nm f=0.1498 <S**2>=0.000
89 -> 95 -0.23398
90 -> 95 -0.10249
92 -> 96 -0.29008
93 -> 97   0.23369
94 -> 98 -0.16153
94 -> 99   0.48299
94 ->100   0.10385

Excited State 14: Singlet-A 5.0654 eV 244.77 nm f=0.0903 <S**2>=0.000
92 -> 96   0.58309
93 -> 97   0.32431
94 -> 99   0.16542

Excited State 15: Singlet-A 5.2012 eV 238.38 nm f=0.0064 <S**2>=0.000
91 -> 95   0.48597
91 -> 96   0.44961
91 -> 97   0.17511

Excited State 16: Singlet-A 5.2550 eV 235.94 nm f=0.2235 <S**2>=0.000
92 -> 96 -0.19156
93 -> 96 -0.12350
93 -> 97   0.54621
94 -> 99 -0.33449

Excited State 17: Singlet-A 5.5625 eV 222.89 nm f=0.0024 <S**2>=0.000
87 -> 95 -0.15919
90 -> 95 -0.12291

90 -> 96   0.65955
90 -> 97 -0.13312

Excited State 18: Singlet-A 5.6316 eV 220.16 nm f=0.0022 <S**2>=0.000
85 -> 95 -0.11036
89 -> 95   0.11586
89 -> 96   0.66599

Excited State 19: Singlet-A 5.6753 eV 218.46 nm f=0.0302 <S**2>=0.000
87 -> 95   0.18669
92 -> 97   0.28011
93 -> 97 -0.10933
93 -> 98   0.21854
93 -> 99   0.17440
94 ->100   0.50855

Excited State 20: Singlet-A 5.7040 eV 217.36 nm f=0.0093 <S**2>=0.000
87 -> 95   0.62193
87 -> 96 -0.11050
87 -> 97   0.10352
90 -> 96   0.15920
94 ->100 -0.17740

Excited State 21: Singlet-A 5.7358 eV 216.16 nm f=0.0000 <S**2>=0.000
88 -> 95 -0.36659
88 -> 96   0.53597
88 -> 97 -0.22117
88 -> 99   0.10502

Excited State 22: Singlet-A 5.7943 eV 213.98 nm f=0.0234 <S**2>=0.000
85 -> 95   0.60809
93 -> 98 -0.20943
93 -> 99 -0.15453
94 ->100   0.11298

Excited State 23: Singlet-A 5.8441 eV 212.15 nm f=0.1137 <S**2>=0.000
85 -> 95   0.22624

92 -> 97 -0.18031

93 -> 98   0.60300

94 ->100 -0.11573

Excited State 24: Singlet-A 5.8815 eV 210.80 nm f=0.0098 <S**2>=0.000

92 -> 97   0.57951

93 -> 98   0.10194

93 -> 99 -0.24042

94 ->100 -0.25244

8.17. XYZ-coordinates of the S_0 state of compound 15c (PBE1PBE/6-311G(d,p)) and TD-DFT calculation (PBE1PBE/6-21G, SCRF(IEFPCM, DCM)

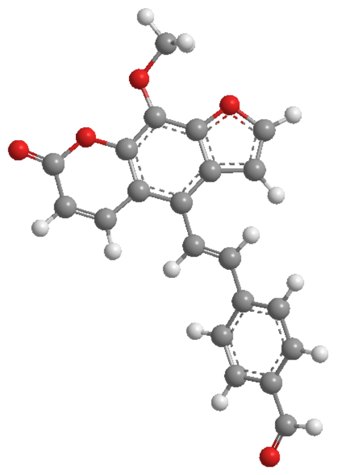

O 3.927635 1.693324 0.092236

C 3.598108 3.025463 0.141370

O 4.499971 3.821219 0.217191

C 2.184425 3.327952 0.102794

C 1.260490 2.352137 0.008008

C 1.629514 0.964856 -0.069535

C 0.684991 -0.087414 -0.154669

C 1.208911 -1.387915 -0.200133

C 0.636287 -2.698189 -0.377973

C 1.667843 -3.564562 -0.393319

O 2.868399 -2.936992 -0.246608

C 2.597267 -1.611743 -0.137840

C 3.545187 -0.598021 -0.029629

C 3.008279 0.697280 -0.005756

O 4.878099 -0.705303 0.025715
C 5.476068 -1.977155 0.266136
C -0.742233 0.189954 -0.222037
C -1.715248 -0.596493 0.274440
C -3.148643 -0.344167 0.196914
C -4.013710 -1.168470 0.931875
C -5.383563 -0.966763 0.905606
C -5.926109 0.063833 0.138490
C -5.073928 0.887406 -0.607216
C -3.710461 0.685600 -0.581969
C -7.380358 0.271467 0.115815
O -7.944905 1.133563 -0.520703
H 1.915712 4.374652 0.161114
H 0.210767 2.621472 0.000276
H -0.402684 -2.957947 -0.496936
H 1.720728 -4.636282 -0.501425
H 6.537750 -1.773613 0.392226
H 5.078054 -2.430146 1.177180
H 5.326945 -2.651093 -0.578823
H -1.027345 1.121266 -0.703063
H -1.436831 -1.488937 0.828217
H -3.596665 -1.971260 1.531687
H -6.040929 -1.611343 1.482347
H -5.509055 1.679470 -1.207263
H -3.071180 1.324861 -1.180698
H -7.957558 -0.437915 0.744281

SCF Done: E(RPBE1PBE) = -1183.58697126 A.U. after 14 cycles

E(PBE1PBE) = -3107507.829760524 kJ/mol

Sum of electronic and zero-point Energies=   -1183.393572

Sum of electronic and thermal Energies=   -1183.372176

Sum of electronic and thermal Enthalpies=   -1183.371231

Sum of electronic and thermal Free Energies= -1183.446274

HOMO -5.967 eV

LUMO -2.476 eV

Relevant excitation energies and oscillator strengths:

Excited State 1: Singlet-A 3.0258 eV 409.75 nm f=0.8001 <S**2>=0.000

90 -> 91   0.70036

This state for optimization and/or second-order correction.

Total Energy, E(TD-HF/TD-KS) = -1181.96389150

Copying the excited state density for this state as the 1-particle RhoCI density.

Excited State 2: Singlet-A 3.5948 eV 344.90 nm f=0.0442 <S**2>=0.000

87 -> 91 -0.23574

87 -> 92 -0.14961

87 -> 93 -0.11236

88 -> 91 -0.39574

88 -> 92 -0.20555

88 -> 93 -0.10399

90 -> 92 0.40811

Excited State 3: Singlet-A 3.6032 eV 344.10 nm f=0.0859 <S**2>=0.000

87 -> 91 0.26358

87 -> 92 0.12166

88 -> 91 0.24300

88 -> 92 0.15336

88 -> 93 0.11350

89 -> 91 0.11926

90 -> 92 0.52958

Excited State 4: Singlet-A 3.8663 eV 320.68 nm f=0.2705 <S**2>=0.000

89 -> 91 0.66788

90 -> 92 -0.16487

Excited State 5: Singlet-A 4.4443 eV 278.98 nm f=0.1637 <S**2>=0.000

87 -> 91 0.47350

88 -> 91 -0.35752

89 -> 92 0.26363

90 -> 93 0.21294

Excited State 6: Singlet-A 4.5434 eV 272.89 nm f=0.0005 <S**2>=0.000

85 -> 91 0.49973

85 -> 92 -0.37155

86 -> 91 0.16109

89 -> 92 0.10488

90 -> 93   0.18578

Excited State 7: Singlet-A 4.5631 eV 271.71 nm f=0.0180 <S**2>=0.000
85 -> 91 -0.20947
85 -> 92   0.15610
86 -> 91   0.44423
86 -> 92   0.10463
87 -> 91 -0.16840
88 -> 91   0.13279
89 -> 92   0.11058
90 -> 93   0.26998
90 -> 94 -0.21523

Excited State 8: Singlet-A 4.5943 eV 269.86 nm f=0.0780 <S**2>=0.000
86 -> 91 -0.28707
87 -> 91 -0.19599
88 -> 91   0.14864
89 -> 92   0.47591
90 -> 93   0.18413
90 -> 94   0.20869

Excited State 9: Singlet-A 4.6904 eV 264.34 nm f=0.3702 <S**2>=0.000
86 -> 91 -0.19337
89 -> 92 -0.39167
90 -> 93   0.51631

Excited State 10: Singlet-A 5.0136 eV 247.30 nm f=0.0473 <S**2>=0.000
84 -> 91 -0.36514
86 -> 91   0.22517
89 -> 93   0.11888
90 -> 94   0.45845
90 -> 95 -0.23924

Excited State 11: Singlet-A 5.0493 eV 245.55 nm f=0.0001 <S**2>=0.000
87 -> 91 -0.24679
87 -> 92   0.29571
87 -> 93   0.11071

88 -> 91 -0.30531
88 -> 92 0.42800
88 -> 93 0.15150

Excited State 12: Singlet-A 5.0613 eV 244.97 nm f=0.0132 <S**2>=0.000
84 -> 91 0.56184
86 -> 91 0.17069
87 -> 92 0.11297
90 -> 94 0.32884

Excited State 13: Singlet-A 5.1815 eV 239.28 nm f=0.0327 <S**2>=0.000
87 -> 92 0.49259
88 -> 92 -0.36819
90 -> 94 -0.14626
90 -> 95 -0.23324

Excited State 14: Singlet-A 5.1912 eV 238.83 nm f=0.1607 <S**2>=0.000
84 -> 91 -0.10955
86 -> 91 0.15477
87 -> 92 0.20917
88 -> 92 -0.14866
89 -> 93 -0.31297
90 -> 94 0.17744
90 -> 95 0.49071

Excited State 15: Singlet-A 5.4468 eV 227.63 nm f=0.0005 <S**2>=0.000
85 -> 91 0.43124
85 -> 92 0.51745
85 -> 93 -0.11187
85 -> 95 -0.11762

Excited State 16: Singlet-A 5.5246 eV 224.42 nm f=0.3081 <S**2>=0.000
83 -> 91 -0.11074
87 -> 92 0.10272
89 -> 93 0.56440
90 -> 95 0.29814
90 -> 96 -0.13560

Excited State 17: Singlet-A 5.5684 eV 222.66 nm f=0.0157 <S**2>=0.000
86 -> 91 -0.14505
86 -> 92   0.64243

Excited State 18: Singlet-A 5.8029 eV 213.66 nm f=0.0176 <S**2>=0.000
83 -> 91   0.12294
84 -> 92   0.66559

Excited State 19: Singlet-A 5.8400 eV 212.30 nm f=0.0223 <S**2>=0.000
83 -> 91   0.15015
87 -> 92 -0.12598
87 -> 94 -0.16557
88 -> 92 -0.18588
88 -> 93   0.44144
88 -> 94 -0.18545
89 -> 93   0.12265
90 -> 96   0.33543

Excited State 20: Singlet-A 5.8445 eV 212.14 nm f=0.0320 <S**2>=0.000
83 -> 91 -0.19072
87 -> 92 -0.13869
87 -> 93   0.39421
87 -> 94 -0.12316
88 -> 92 -0.15977
88 -> 93   0.13521
88 -> 94 -0.20827
89 -> 93 -0.13396
89 -> 94   0.10917
90 -> 96 -0.34203

Excited State 21: Singlet-A 5.8747 eV 211.05 nm f=0.0147 <S**2>=0.000
83 -> 91   0.57179
87 -> 93   0.27310
88 -> 93 -0.17419
90 -> 96 -0.12639

Excited State 22: Singlet-A 5.9944 eV 206.83 nm f=0.1763 <S**2>=0.000

82 -> 91 -0.17143

86 -> 92 0.10263

89 -> 94 0.60572

89 -> 95 -0.14965

90 -> 96 0.17042

Excited State 23: Singlet-A 6.0128 eV 206.20 nm f=0.0125 <S**2>=0.000

82 -> 91 -0.17442

83 -> 91 -0.18857

87 -> 93 0.38885

88 -> 93 -0.28974

89 -> 94 -0.17575

90 -> 96 0.37328

Excited State 24: Singlet-A 6.0923 eV 203.51 nm f=0.0001 <S**2>=0.000

87 -> 93 0.13949

87 -> 94 0.34360

87 -> 95 0.16520

88 -> 92 -0.10276

88 -> 93 0.18630

88 -> 94 0.46642

88 -> 95 0.22030

8.18. XYZ-coordinates of the S_0 state of compound 15d (PBE1PBE/6-311G(d,p)) and TD-DFT calculation (PBE1PBE/6-21G, SCRF(IEFPCM, DCM)

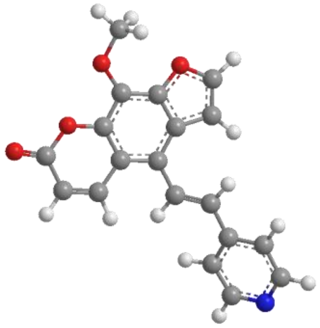

O 3.360159 1.561606 0.093630

C 3.103042 2.910319 0.124630

O 4.046423 3.656877 0.194902

C 1.708011 3.288604 0.075554
C 0.733046 2.363424 -0.014605
C 1.027165 0.957356 -0.075616
C 0.027522 -0.042076 -0.157725
C 0.477819 -1.369665 -0.182481
C -0.165736 -2.648364 -0.346199
C 0.816315 -3.570540 -0.343242
O 2.049136 -3.008649 -0.197334
C 1.851426 -1.668925 -0.107818
C 2.853082 -0.707581 -0.005600
C 2.388590 0.615875 0.000770
O 4.177517 -0.887232 0.060031
C 4.704943 -2.189238 0.303836
C -1.381894 0.312957 -0.248846
C -2.404114 -0.402294 0.252104
C -3.817481 -0.056357 0.138152
C -4.314900 0.937938 -0.714049
C -5.675735 1.194411 -0.735331
C -6.102985 -0.409254 0.812251
C -4.760161 -0.743601 0.910543
N -6.575050 0.544115 0.008658
H 1.496450 4.348918 0.121692
H -0.300633 2.688995 -0.030632
H -1.217142 -2.851720 -0.466562
H 0.810199 -4.644989 -0.436059
H 5.776148 -2.043597 0.429508
H 4.282869 -2.617577 1.215971
H 4.519242 -2.856101 -0.539524
H -1.609374 1.246180 -0.756988
H -2.192944 -1.294106 0.835867
H -3.659812 1.501905 -1.368247
H -6.068433 1.965083 -1.394286
H -6.838249 -0.939181 1.413142
H -4.446581 -1.531373 1.587713
SCF Done: E(RPBE1PBE) = -1086.39142411 A.U. after 14 cycles
E(PBE1PBE) = -2852320.9012790895 kJ/mol
Sum of electronic and zero-point Energies= -1086.207697

Sum of electronic and thermal Energies=   -1086.188447

Sum of electronic and thermal Enthalpies=   -1086.187502

Sum of electronic and thermal Free Energies= -1086.257770

HOMO -6.024 eV

LUMO -2.293 eV

Relevant excitation energies and oscillator strengths:

Excited State 1: Singlet-A 3.1921 eV 388.41 nm f=0.4906 <S**2>=0.000

83 -> 84   0.69653

This state for optimization and/or second-order correction.

Total Energy, E(TD-HF/TD-KS) = -1084.88890100

Copying the excited state density for this state as the 1-particle RhoCI density.

Excited State 2: Singlet-A 3.8358 eV 323.23 nm f=0.3356 <S**2>=0.000

82 -> 84   0.30408

83 -> 85   0.62305

Excited State 3: Singlet-A 4.0383 eV 307.02 nm f=0.2290 <S**2>=0.000

82 -> 84   0.60962

83 -> 85 -0.30388

83 -> 86 -0.14839

Excited State 4: Singlet-A 4.1772 eV 296.81 nm f=0.0041 <S**2>=0.000

81 -> 84   0.64325

81 -> 85   0.25368

81 -> 86   0.11034

Excited State 5: Singlet-A 4.5553 eV 272.17 nm f=0.0022 <S**2>=0.000

78 -> 84   0.60240

78 -> 85 -0.31137

78 -> 88   0.11605

80 -> 84 -0.11029

Excited State 6: Singlet-A 4.7530 eV 260.85 nm f=0.0571 <S**2>=0.000

80 -> 84   0.57058

82 -> 85   0.36307

Excited State 7: Singlet-A 4.8153 eV 257.48 nm f=0.0179 <S**2>=0.000

79 -> 84 0.53194

79 -> 85 0.14520

80 -> 87 0.13377

83 -> 87 -0.38988

Excited State 8: Singlet-A 4.8316 eV 256.61 nm f=0.1444 <S**2>=0.000

80 -> 84 -0.34368

82 -> 85 0.56406

83 -> 86 0.16252

Excited State 9: Singlet-A 4.9098 eV 252.53 nm f=0.4184 <S**2>=0.000

77 -> 84 -0.10265

82 -> 84 0.15745

82 -> 85 -0.18750

82 -> 86 0.10528

83 -> 86 0.62141

Excited State 10: Singlet-A 5.1464 eV 240.91 nm f=0.0243 <S**2>=0.000

77 -> 84 -0.27431

79 -> 84 0.38297

83 -> 87 0.50813

Excited State 11: Singlet-A 5.1744 eV 239.61 nm f=0.0490 <S**2>=0.000

77 -> 84 0.61873

79 -> 84 0.15356

83 -> 87 0.23647

Excited State 12: Singlet-A 5.2194 eV 237.55 nm f=0.0043 <S**2>=0.000

81 -> 84 -0.27845

81 -> 85 0.60960

81 -> 86 0.14238

81 -> 87 -0.11855

Excited State 13: Singlet-A 5.3036 eV 233.77 nm f=0.0017 <S**2>=0.000

81 -> 85 0.11841

81 -> 87 0.68695

Excited State 14: Singlet-A 5.5028 eV 225.31 nm f=0.0265 <S**2>=0.000
80 -> 85   0.22850
82 -> 86 -0.34927
83 -> 86   0.10176
83 -> 88   0.53826

Excited State 15: Singlet-A 5.6653 eV 218.85 nm f=0.1107 <S**2>=0.000
78 -> 84 -0.15488
78 -> 85 -0.22458
80 -> 85   0.59016
82 -> 86   0.15010
83 -> 88 -0.12943

Excited State 16: Singlet-A 5.7118 eV 217.07 nm f=0.0310 <S**2>=0.000
78 -> 84   0.29470
78 -> 85   0.54625
78 -> 88 -0.13128
80 -> 85   0.24938
82 -> 86   0.14432

Excited State 17: Singlet-A 5.7941 eV 213.98 nm f=0.0090 <S**2>=0.000
79 -> 84 -0.15696
79 -> 85   0.61282
80 -> 87   0.17508
82 -> 87 -0.16970
83 -> 87   0.13290

Excited State 18: Singlet-A 5.8428 eV 212.20 nm f=0.1991 <S**2>=0.000
76 -> 84   0.23838
77 -> 85   0.19141
82 -> 86   0.48876
83 -> 88   0.34911

Excited State 19: Singlet-A 6.0355 eV 205.42 nm f=0.1980 <S**2>=0.000
77 -> 85   0.66085
82 -> 86 -0.15954

Excited State 20: Singlet-A 6.0850 eV 203.76 nm f=0.0224 <S**2>=0.000
79 -> 85   0.19413
82 -> 87   0.66804

Excited State 21: Singlet-A 6.1794 eV 200.64 nm f=0.0785 <S**2>=0.000
76 -> 84   0.53921
81 -> 86   0.10886
82 -> 86 -0.17964
82 -> 87 -0.10470
82 -> 88 -0.13952
83 -> 89 -0.29207

Excited State 22: Singlet-A 6.2233 eV 199.23 nm f=0.0060 <S**2>=0.000
81 -> 85 -0.19672
81 -> 86   0.63008
81 -> 88   0.15751
83 -> 89   0.10095

Excited State 23: Singlet-A 6.2892 eV 197.14 nm f=0.0223 <S**2>=0.000
74 -> 84   0.15908
75 -> 84   0.11758
76 -> 84   0.21999
80 -> 86 -0.12012
82 -> 88 -0.22897
83 -> 89   0.55560

Excited State 24: Singlet-A 6.3388 eV 195.60 nm f=0.0033 <S**2>=0.000
72 -> 84 -0.15022
73 -> 84   0.11520
74 -> 84 -0.11487
75 -> 84   0.61800
78 -> 86 -0.14265

8.19. XYZ-coordinates of the S_0 state of compound 15e (PBE1PBE/6-311G(d,p)) and TD-DFT calculation (PBE1PBE/6-21G, SCRF(IEFPCM, DCM)

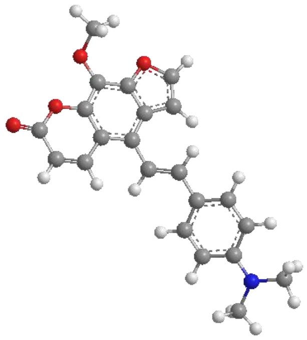

N -6.892375 0.340583 0.084656
C -5.542896 0.134510 0.095967
C -4.964382 -0.937891 0.808453
C -3.595168 -1.124337 0.818012
C -2.716101 -0.274504 0.131426
C -3.297591 0.782025 -0.587980
C -4.661375 0.986501 -0.610963
C -7.458880 1.401961 -0.716753
C -7.771557 -0.591060 0.753711
C -0.298088 0.245619 -0.297874
C -1.287615 -0.527002 0.193477
C 4.117247 2.949633 0.178953
C 2.712810 3.287834 0.154174
C 1.764782 2.337475 0.024792
C 2.097447 0.947269 -0.103517
C 1.121595 -0.073024 -0.226529
C 1.607294 -1.388622 -0.307873
C 2.986526 -1.644499 -0.246148
C 3.963637 -0.664462 -0.112057
C 3.474728 0.639922 -0.043159
O 4.416521 1.611373 0.086690
O 3.238153 -2.964956 -0.397792
C 1.008981 -2.681499 -0.535061
C 2.022957 -3.567681 -0.573036
O 5.039950 3.719418 0.279755
O 5.295268 -0.888467 -0.110512

C 5.774680 -1.818510 0.861734
H -5.590010 -1.630735 1.356549
H -3.187031 -1.960436 1.379738
H -2.670028 1.460596 -1.157383
H -5.053699 1.813232 -1.189523
H -7.071205 2.382666 -0.418224
H -7.255824 1.266588 -1.787170
H -8.538511 1.415277 -0.576765
H -7.708652 -1.600287 0.325713
H -7.543466 -0.660692 1.823434
H -8.798996 -0.244857 0.654607
H -0.558801 1.188020 -0.771394
H -1.011727 -1.431105 0.732484
H 2.470000 4.337777 0.253023
H 0.721077 2.630210 0.030245
H -0.034616 -2.915745 -0.668062
H 2.057361 -4.635881 -0.716242
H 6.861123 -1.785978 0.794889
H 5.461644 -1.518140 1.866188
H 5.424653 -2.831489 0.651682
SCF Done: E(RPBE1PBE) = -1204.16869910 A.U. after 14 cycles
E(PBE1PBE) = -3161545.1603207896 kJ/mol
Sum of electronic and zero-point Energies=   -1203.940325
Sum of electronic and thermal Energies=   -1203.916307
Sum of electronic and thermal Enthalpies=   -1203.915362
Sum of electronic and thermal Free Energies= -1203.996763
HOMO -5.319 eV
LUMO -2.033 eV
Relevant excitation energies and oscillator strengths:
Excited State 1: Singlet-A 2.7834 eV 445.45 nm f=0.6721 <S**2>=0.000
95 -> 96   0.70244
This state for optimization and/or second-order correction.
Total Energy, E(TD-HF/TD-KS) = -1202.58444447
Copying the excited state density for this state as the 1-particle RhoCI density.

Excited State 2: Singlet-A 3.6774 eV 337.16 nm f=0.2719 <S**2>=0.000
93 -> 96   0.16805

94 -> 96 0.44117
95 -> 97 0.50016

Excited State 3: Singlet-A 3.7647 eV 329.34 nm f=0.3892 <S**2>=0.000
94 -> 96 0.51830
95 -> 97 -0.46987

Excited State 4: Singlet-A 4.2197 eV 293.82 nm f=0.2481 <S**2>=0.000
93 -> 96 0.64254
94 -> 96 -0.12021
95 -> 97 -0.13964
95 -> 98 -0.13393

Excited State 5: Singlet-A 4.4501 eV 278.61 nm f=0.0254 <S**2>=0.000
92 -> 96 -0.18159
92 -> 97 0.10284
94 -> 97 -0.10750
95 -> 98 -0.44113
95 -> 99 0.47280

Excited State 6: Singlet-A 4.5437 eV 272.87 nm f=0.1772 <S**2>=0.000
92 -> 96 -0.15086
94 -> 97 0.30968
95 -> 98 0.43309
95 -> 99 0.40786

Excited State 7: Singlet-A 4.6098 eV 268.96 nm f=0.0008 <S**2>=0.000
89 -> 96 0.30926
89 -> 97 0.11233
90 -> 96 0.57474
90 -> 97 0.18808

Excited State 8: Singlet-A 4.7374 eV 261.72 nm f=0.1847 <S**2>=0.000
91 -> 96 -0.10344
92 -> 96 0.10021
93 -> 96 -0.14208
94 -> 97 0.59226

95 -> 98 -0.26853
95 -> 99 -0.10302

Excited State 9: Singlet-A 4.9449 eV 250.73 nm f=0.0593 <S**2>=0.000
92 -> 96   0.65114
95 -> 99   0.25450

Excited State 10: Singlet-A 5.0320 eV 246.39 nm f=0.0163 <S**2>=0.000
91 -> 96   0.44651
93 -> 97   0.48464
95 ->100 -0.16631

Excited State 11: Singlet-A 5.0579 eV 245.13 nm f=0.0554 <S**2>=0.000
89 -> 96 -0.12382
91 -> 96   0.50166
93 -> 97 -0.45641

Excited State 12: Singlet-A 5.2084 eV 238.05 nm f=0.0336 <S**2>=0.000
89 -> 96   0.57622
90 -> 96 -0.32728
91 -> 96   0.10012
95 ->100 -0.12259

Excited State 13: Singlet-A 5.3469 eV 231.88 nm f=0.0179 <S**2>=0.000
89 -> 96   0.11764
93 -> 97   0.15963
95 ->100   0.64242

Excited State 14: Singlet-A 5.4877 eV 225.93 nm f=0.2733 <S**2>=0.000
93 -> 96   0.10580
93 -> 98 -0.11642
94 -> 98   0.65706

Excited State 15: Singlet-A 5.7170 eV 216.87 nm f=0.0043 <S**2>=0.000
93 -> 99 -0.10956
94 -> 99   0.68124

Excited State 16: Singlet-A 5.8583 eV 211.64 nm f=0.0663 <S**2>=0.000
87 -> 96 -0.14569
88 -> 96 -0.27891
93 -> 98   0.55473
94 ->100   0.12095
95 ->101   0.14084

Excited State 17: Singlet-A 5.8691 eV 211.25 nm f=0.0291 <S**2>=0.000
92 -> 97   0.67156
95 -> 99 -0.11454

Excited State 18: Singlet-A 5.9265 eV 209.20 nm f=0.0604 <S**2>=0.000
88 -> 96   0.54925
90 -> 97 -0.10154
93 -> 98   0.26089
94 -> 98   0.10459
94 ->100   0.14458
95 ->101 -0.23562

Excited State 19: Singlet-A 5.9792 eV 207.36 nm f=0.0152 <S**2>=0.000
88 -> 96   0.17733
89 -> 97   0.13386
90 -> 97 -0.26821
91 -> 97 -0.36001
95 ->101   0.45353

Excited State 20: Singlet-A 6.0116 eV 206.24 nm f=0.0209 <S**2>=0.000
88 -> 96   0.17238
90 -> 97   0.16301
91 -> 97   0.46248
94 ->100   0.17262
95 ->101   0.39909

Excited State 21: Singlet-A 6.0649 eV 204.43 nm f=0.0357 <S**2>=0.000
89 -> 96 -0.13430
89 -> 97   0.25849
90 -> 96 -0.18110

90 -> 97   0.52337
90 ->100 -0.10144
91 -> 97 -0.25485

Excited State 22: Singlet-A 6.1937 eV 200.18 nm f=0.0498 <S**2>=0.000
86 -> 96   0.10643
87 -> 96   0.37561
88 -> 96 -0.11900
91 -> 97 -0.12212
93 ->100   0.10543
94 ->100   0.52065

Excited State 23: Singlet-A 6.2179 eV 199.40 nm f=0.1806 <S**2>=0.000
89 -> 97   0.58006
90 -> 97 -0.20308
91 -> 97   0.17912
93 -> 98 -0.11276
95 ->101 -0.16281

Excited State 24: Singlet-A 6.3333 eV 195.76 nm f=0.0236 <S**2>=0.000
87 -> 96 -0.15440
93 -> 98 -0.11960
93 -> 99   0.62525
94 -> 99   0.11368
94 ->100   0.18941

8.20. Kohn-Sham FMOs of the DFT calculated structures 11a, 11e, 13a, 13e, 15a, 15e, and 5-Ph-8-MOP

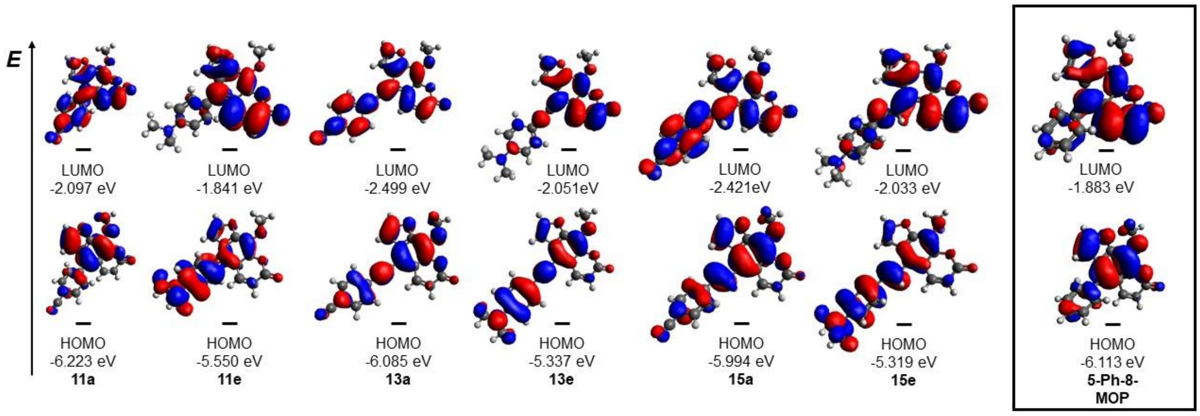

9. Literature

[1] V. S. Raju, G. V. Subbaraju, M. S. Manhas, Z. Kaluza, A. K. Bose, *Tetrahedron* **1992**, *48*, 8347-8352.

[2] J. I. Maki, P. J. Malkonen, H. E. Nupponen, United States Patent US4169840 A, **1979**.

[3] B.-L. Zhang, C.-Q. Fan, L. Dong, F.-D. Wang, J.-M. Yue, *Eur. J. Med. Chem.* **2010**, *45*, 5258-5264.

[4] C. Reichardt, T. Welton, P. Appendix A. Properties, and Use of Organic Solvents, *Solvents and Solvent Effects in Organic Chemistry*, Wiley-VCH, Weinheim, **2010**.
